# Supplementary figures and images for: SDR enzymes oxidize specific lipidic alkynylcarbinols into cytotoxic protein-reactive species (part 1 of 2)
Source: eLife. 2022 May 10;11:e73913. doi: 10.7554/eLife.73913 (PMC9090334; doi:10.7554/eLife.73913)

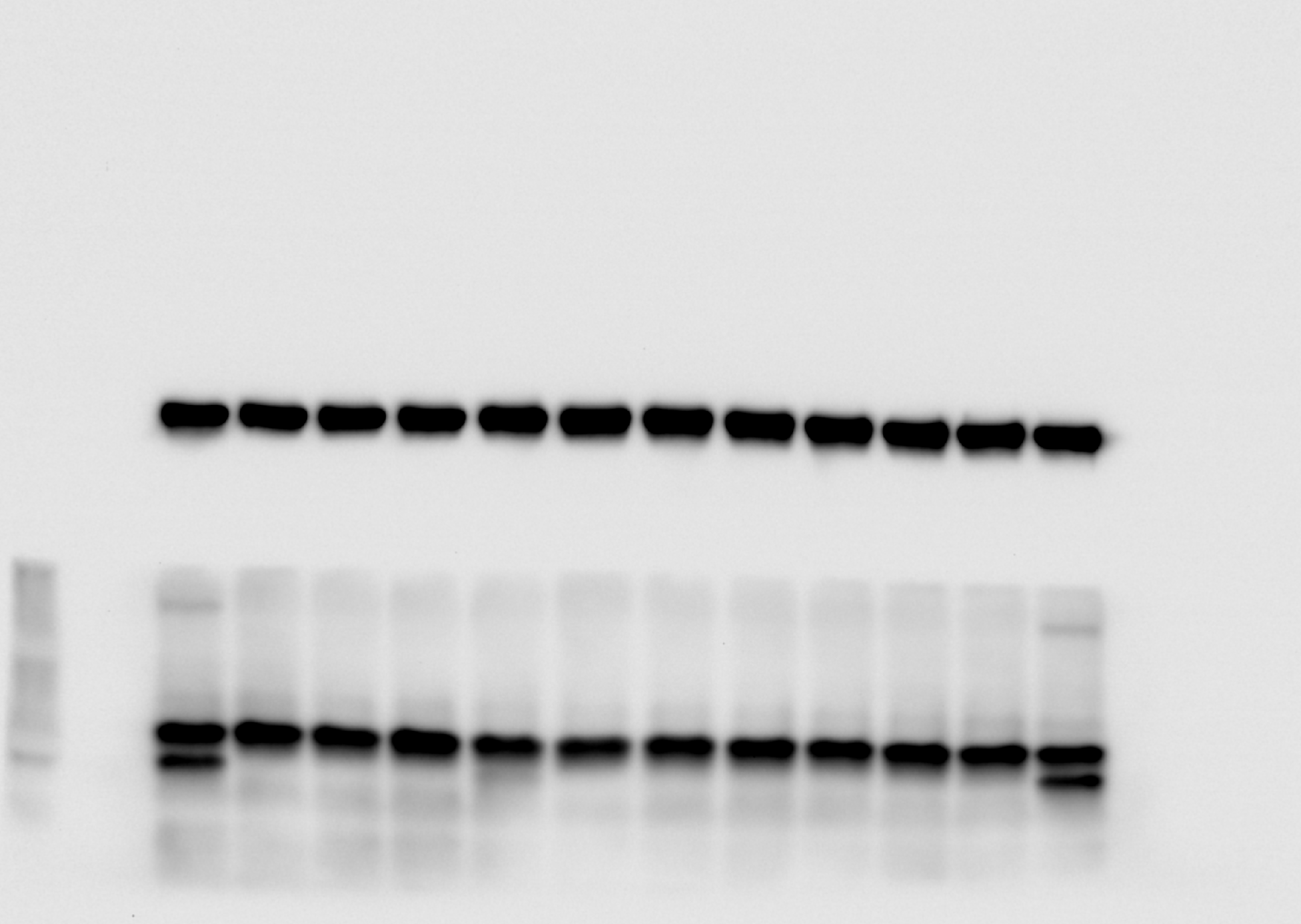

Supplement: Figure 1—source data 1. — The tiff files correspond to uncropped pictures of the chemiluminescent signal acquired on a BioRad Chemidoc. The regions used to generate the figure are highlighted by back squares in the jpg file, which also contains at the bottom an overlay with a picture of the membrane to locate the protein ladder positions. [file elife-73913-fig1-data1.zip › Figure 1-source data 1/Fig.1F-HSD17B11.tif]

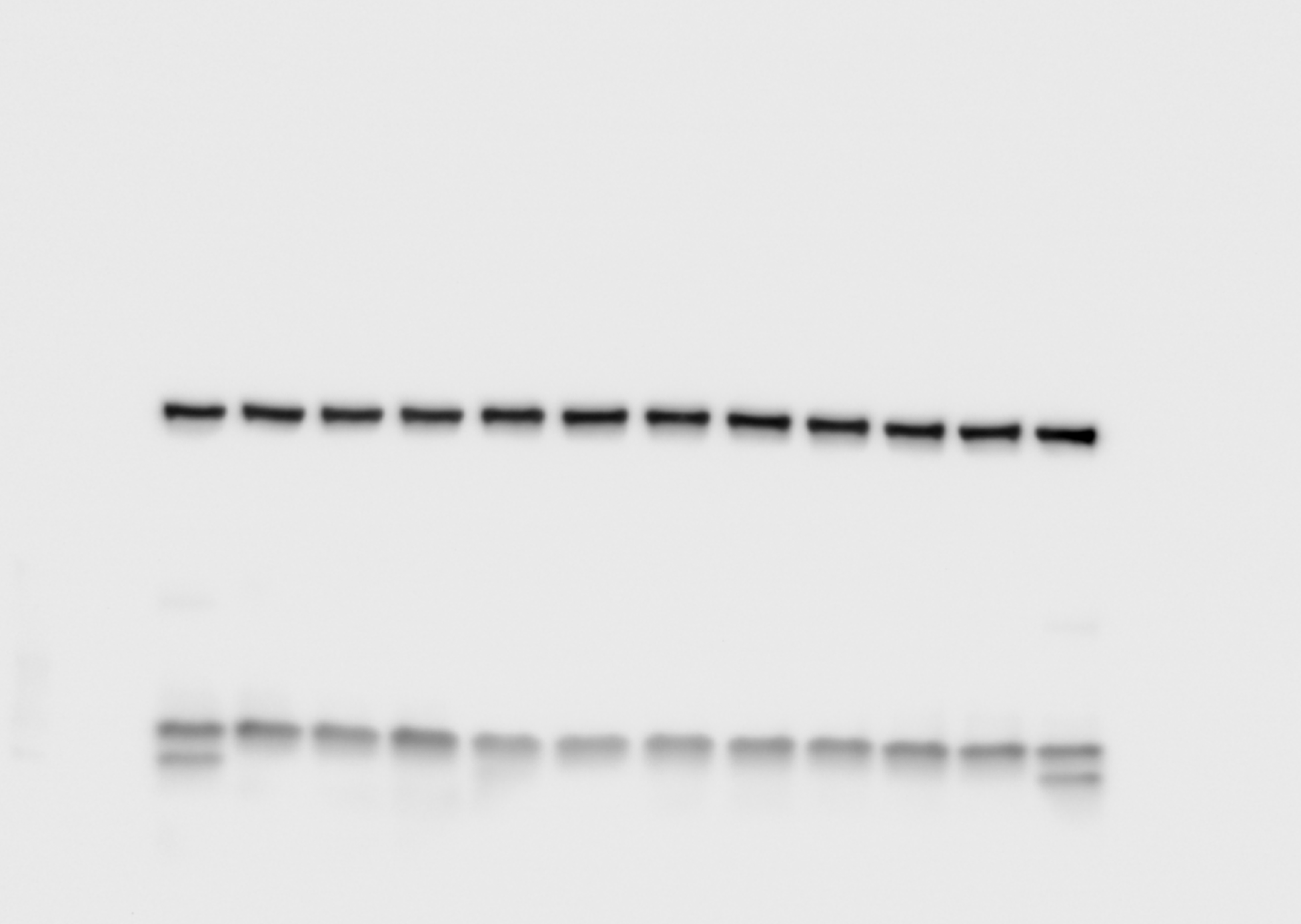

Supplement: Figure 1—source data 1. — The tiff files correspond to uncropped pictures of the chemiluminescent signal acquired on a BioRad Chemidoc. The regions used to generate the figure are highlighted by back squares in the jpg file, which also contains at the bottom an overlay with a picture of the membrane to locate the protein ladder positions. [file elife-73913-fig1-data1.zip › Figure 1-source data 1/Fig.1F-Ku80.tif]

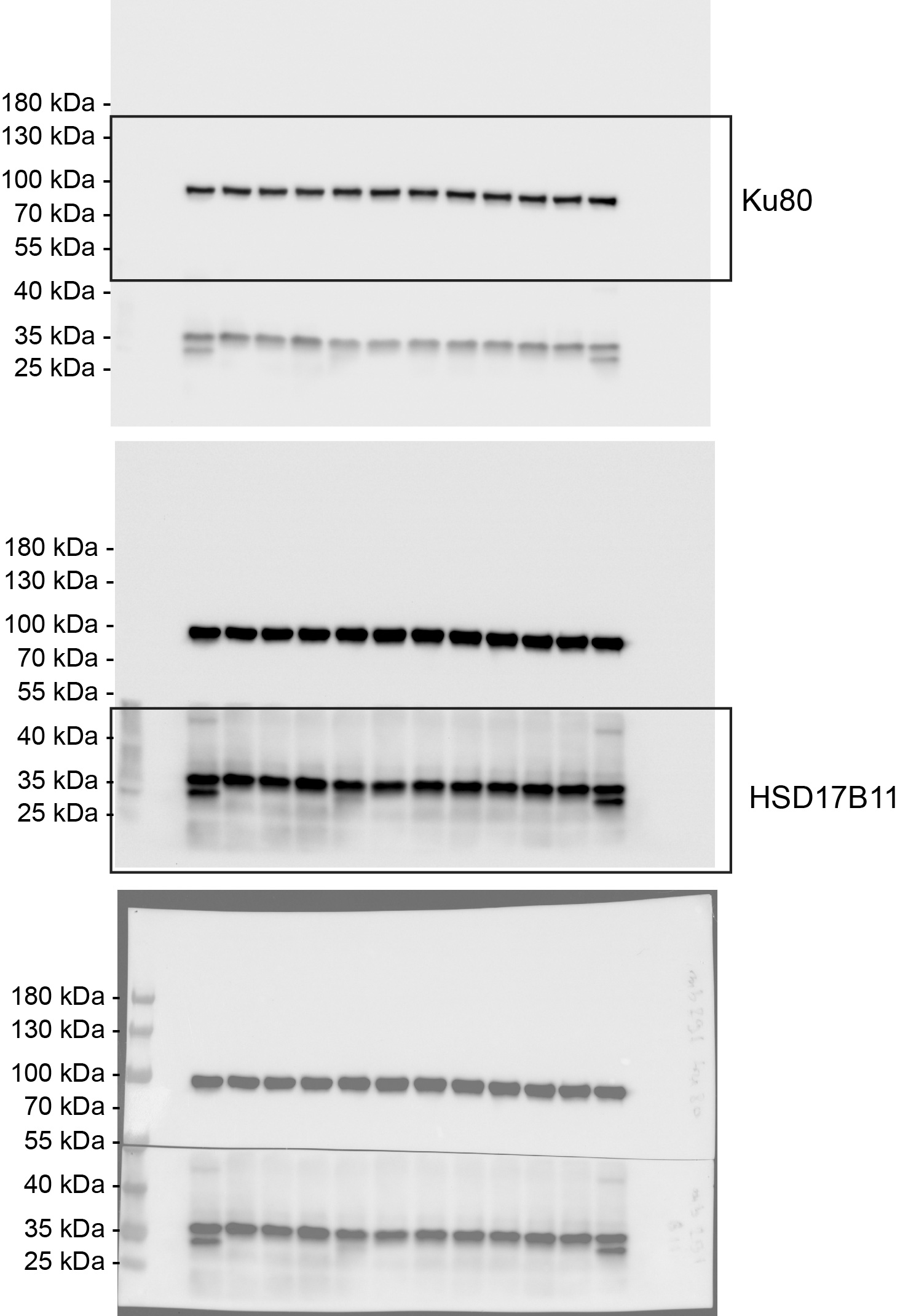

Supplement: Figure 1—source data 1. — The tiff files correspond to uncropped pictures of the chemiluminescent signal acquired on a BioRad Chemidoc. The regions used to generate the figure are highlighted by back squares in the jpg file, which also contains at the bottom an overlay with a picture of the membrane to locate the protein ladder positions. [file elife-73913-fig1-data1.zip › Figure 1-source data 1/Fig.1F.jpg]

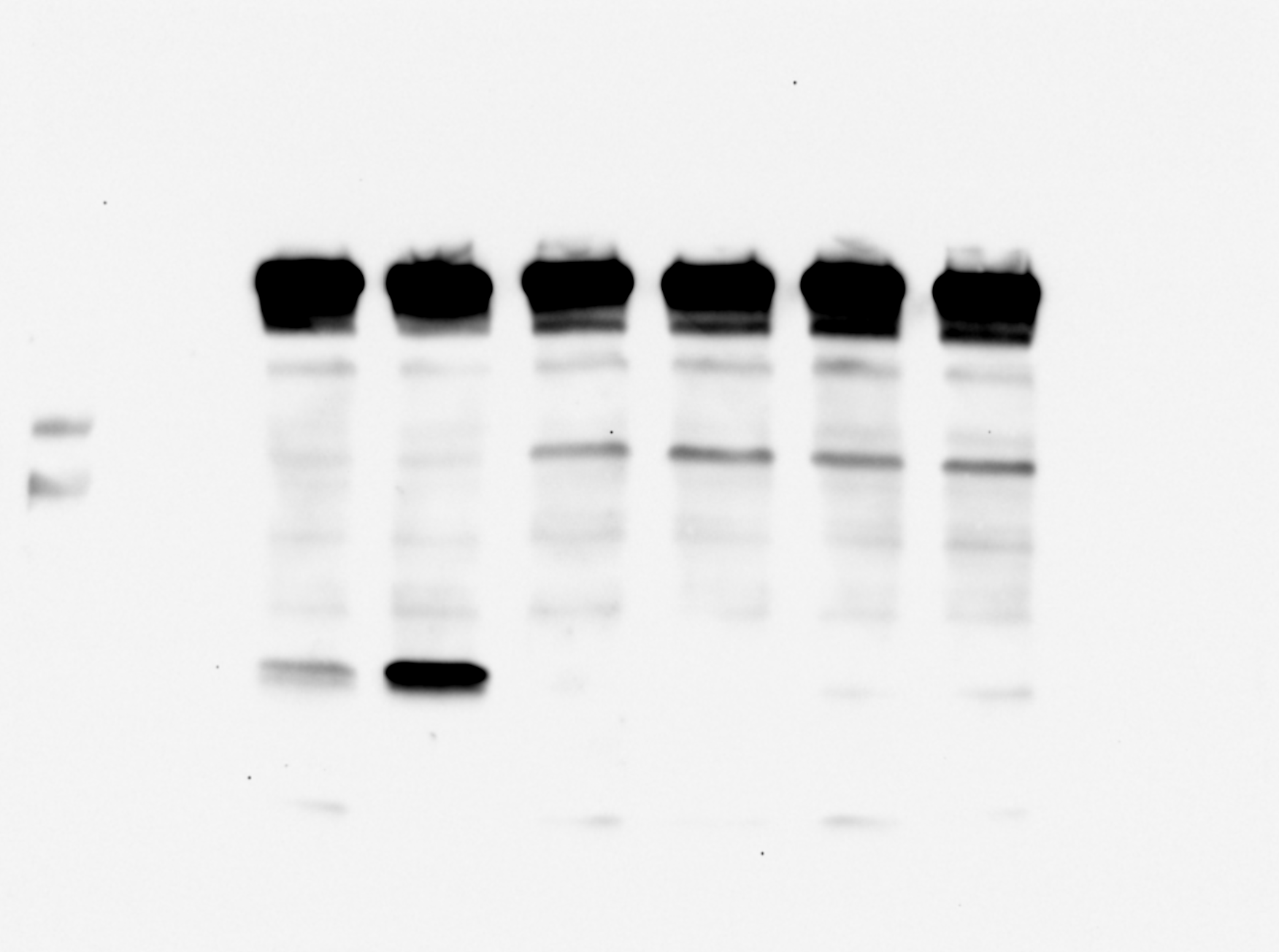

Supplement: Figure 1—source data 2. — The tiff files correspond to uncropped pictures of the chemiluminescent signal acquired on a BioRad Chemidoc. The regions used to generate the figure are highlighted by back squares in the jpg file, which also contains at the bottom an overlay with a picture of the membrane to locate the protein ladder positions. [file elife-73913-fig1-data2.zip › Figure 1-source data 2/Fig.1G-GFP.tif]

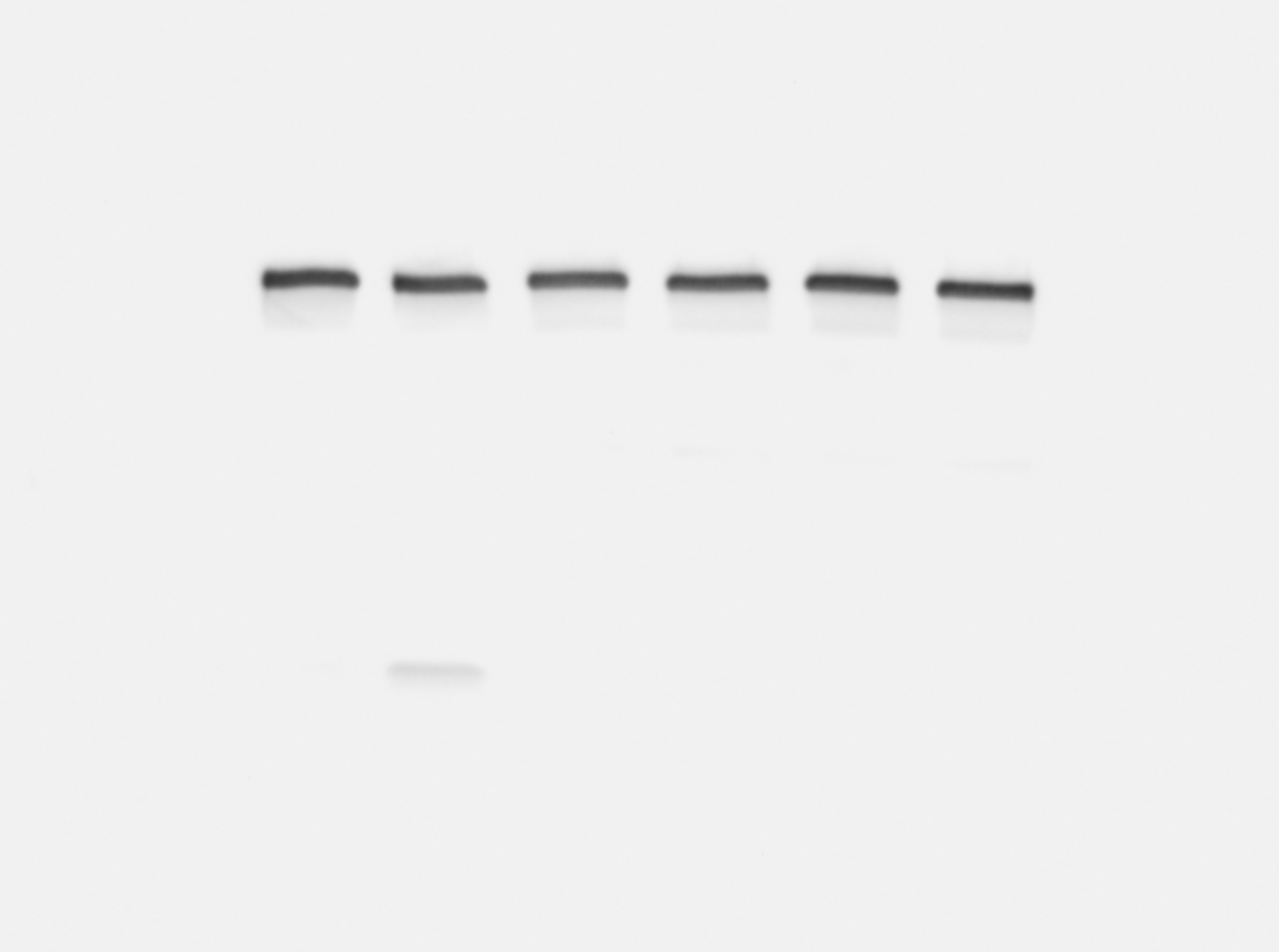

Supplement: Figure 1—source data 2. — The tiff files correspond to uncropped pictures of the chemiluminescent signal acquired on a BioRad Chemidoc. The regions used to generate the figure are highlighted by back squares in the jpg file, which also contains at the bottom an overlay with a picture of the membrane to locate the protein ladder positions. [file elife-73913-fig1-data2.zip › Figure 1-source data 2/Fig.1G-SAFA.tif]

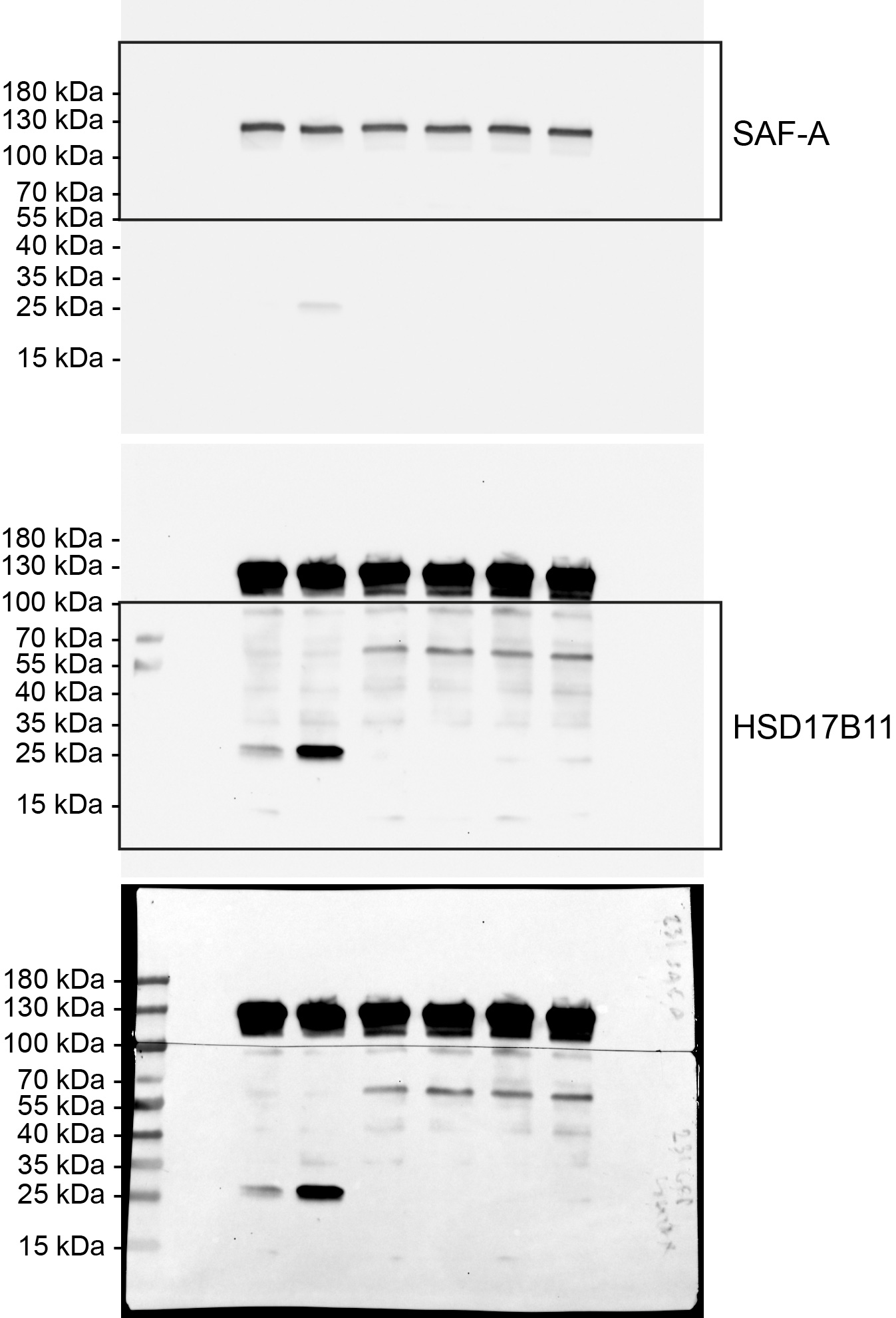

Supplement: Figure 1—source data 2. — The tiff files correspond to uncropped pictures of the chemiluminescent signal acquired on a BioRad Chemidoc. The regions used to generate the figure are highlighted by back squares in the jpg file, which also contains at the bottom an overlay with a picture of the membrane to locate the protein ladder positions. [file elife-73913-fig1-data2.zip › Figure 1-source data 2/Fig.1G.jpg]

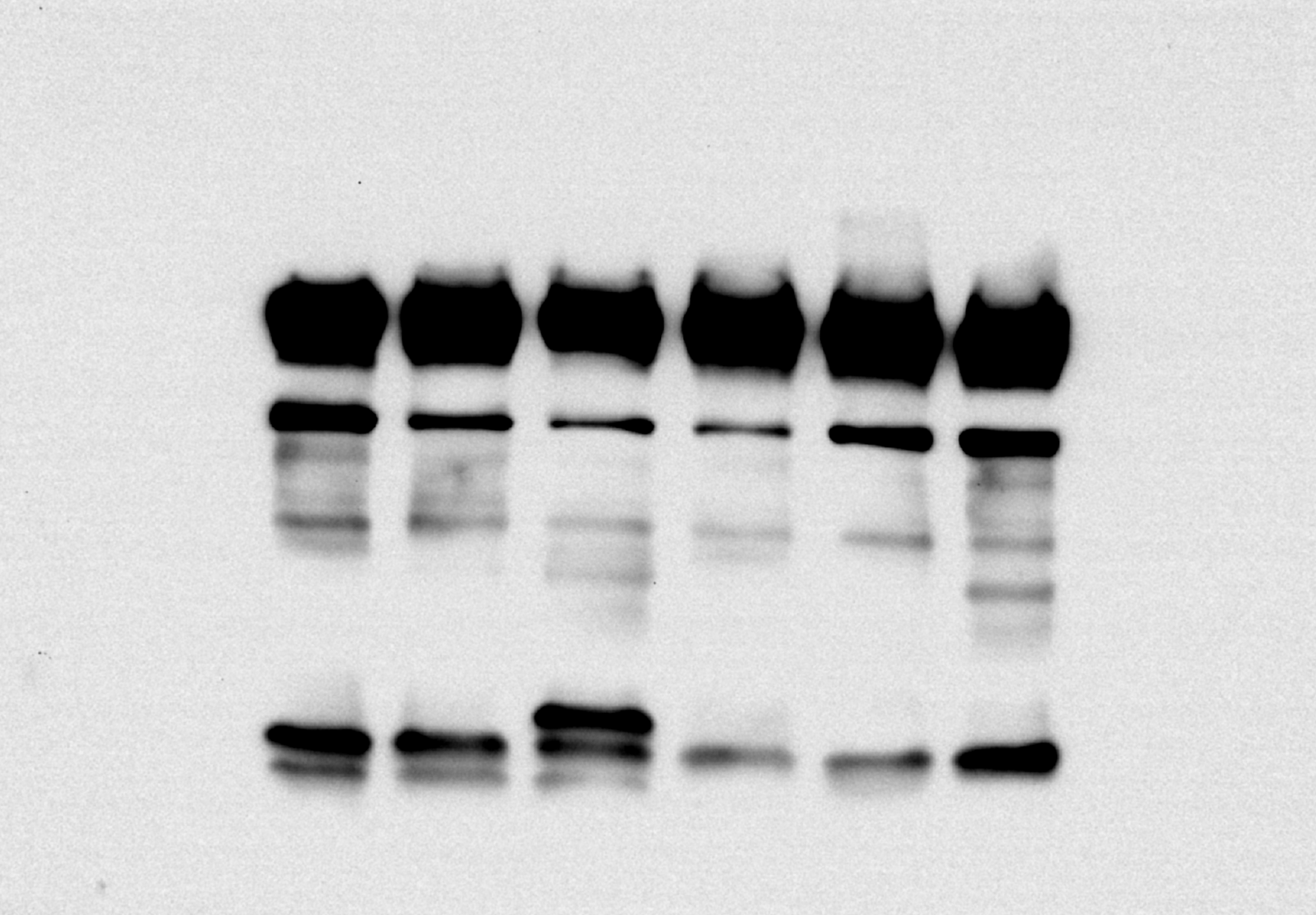

Supplement: Figure 1—figure supplement 4—source data 1. — The tiff files correspond to uncropped pictures of the chemiluminescent signal acquired on a BioRad Chemidoc. The regions used to generate the figure are highlighted by back squares in the jpg file, which also contains at the bottom an overlay with a picture of the membrane to locate the protein ladder positions. [file elife-73913-fig1-figsupp4-data1.zip › Figure 1-figure supplement 4-source data 1/Fig.1-S4B-HSD17B11.tif]

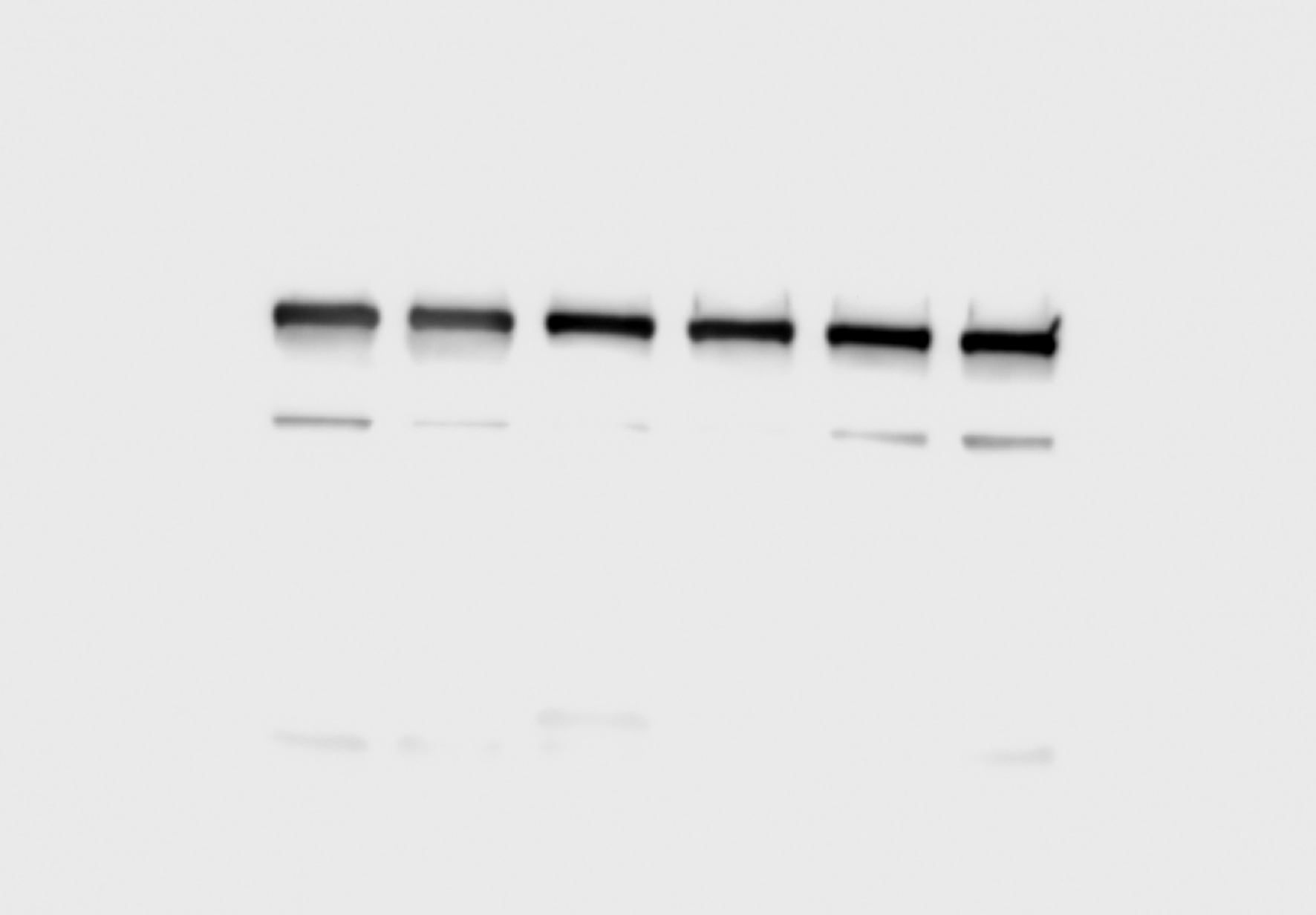

Supplement: Figure 1—figure supplement 4—source data 1. — The tiff files correspond to uncropped pictures of the chemiluminescent signal acquired on a BioRad Chemidoc. The regions used to generate the figure are highlighted by back squares in the jpg file, which also contains at the bottom an overlay with a picture of the membrane to locate the protein ladder positions. [file elife-73913-fig1-figsupp4-data1.zip › Figure 1-figure supplement 4-source data 1/Fig.1-S4B-SAFA.tif]

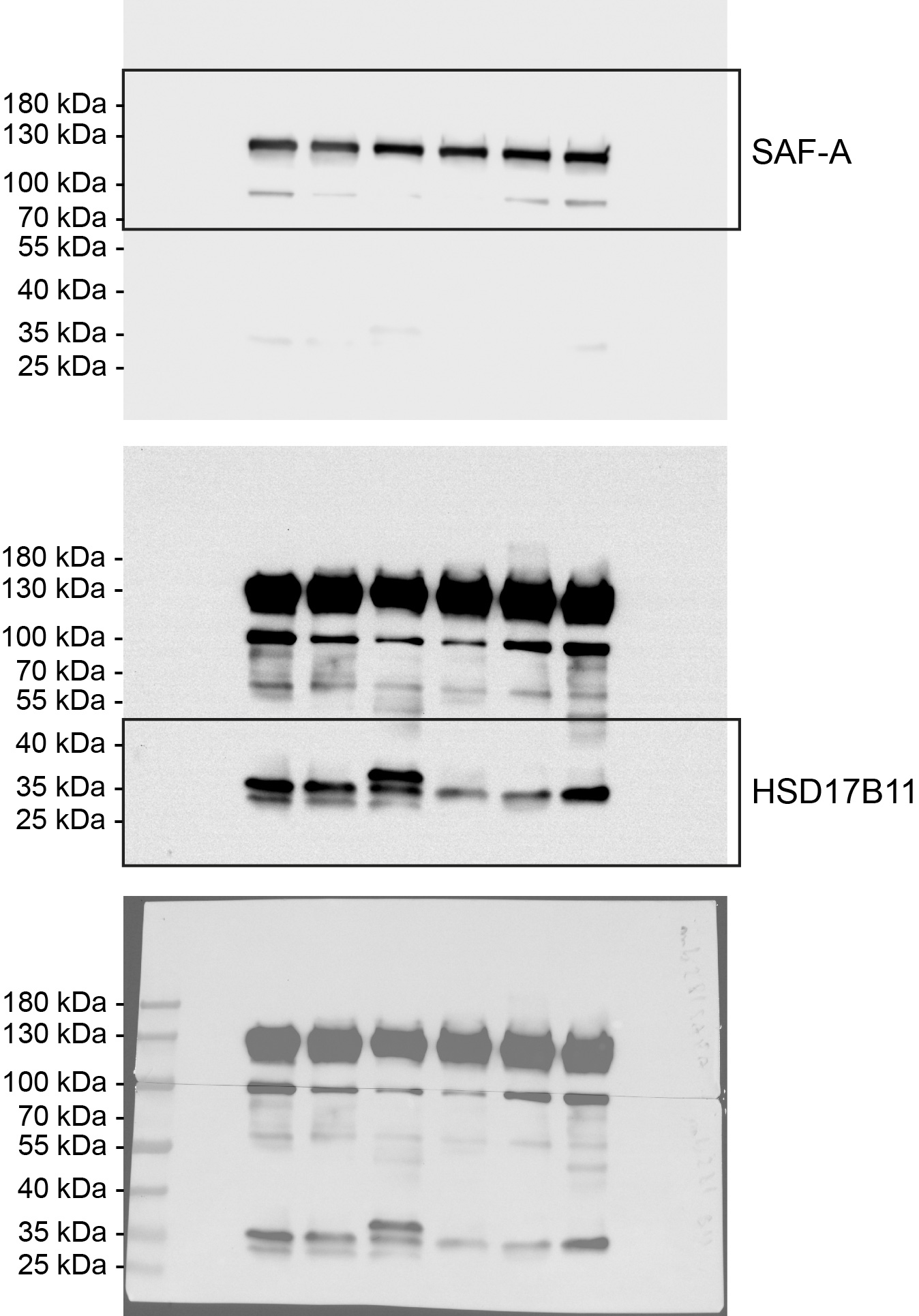

Supplement: Figure 1—figure supplement 4—source data 1. — The tiff files correspond to uncropped pictures of the chemiluminescent signal acquired on a BioRad Chemidoc. The regions used to generate the figure are highlighted by back squares in the jpg file, which also contains at the bottom an overlay with a picture of the membrane to locate the protein ladder positions. [file elife-73913-fig1-figsupp4-data1.zip › Figure 1-figure supplement 4-source data 1/Fig.1-S4B.jpg]

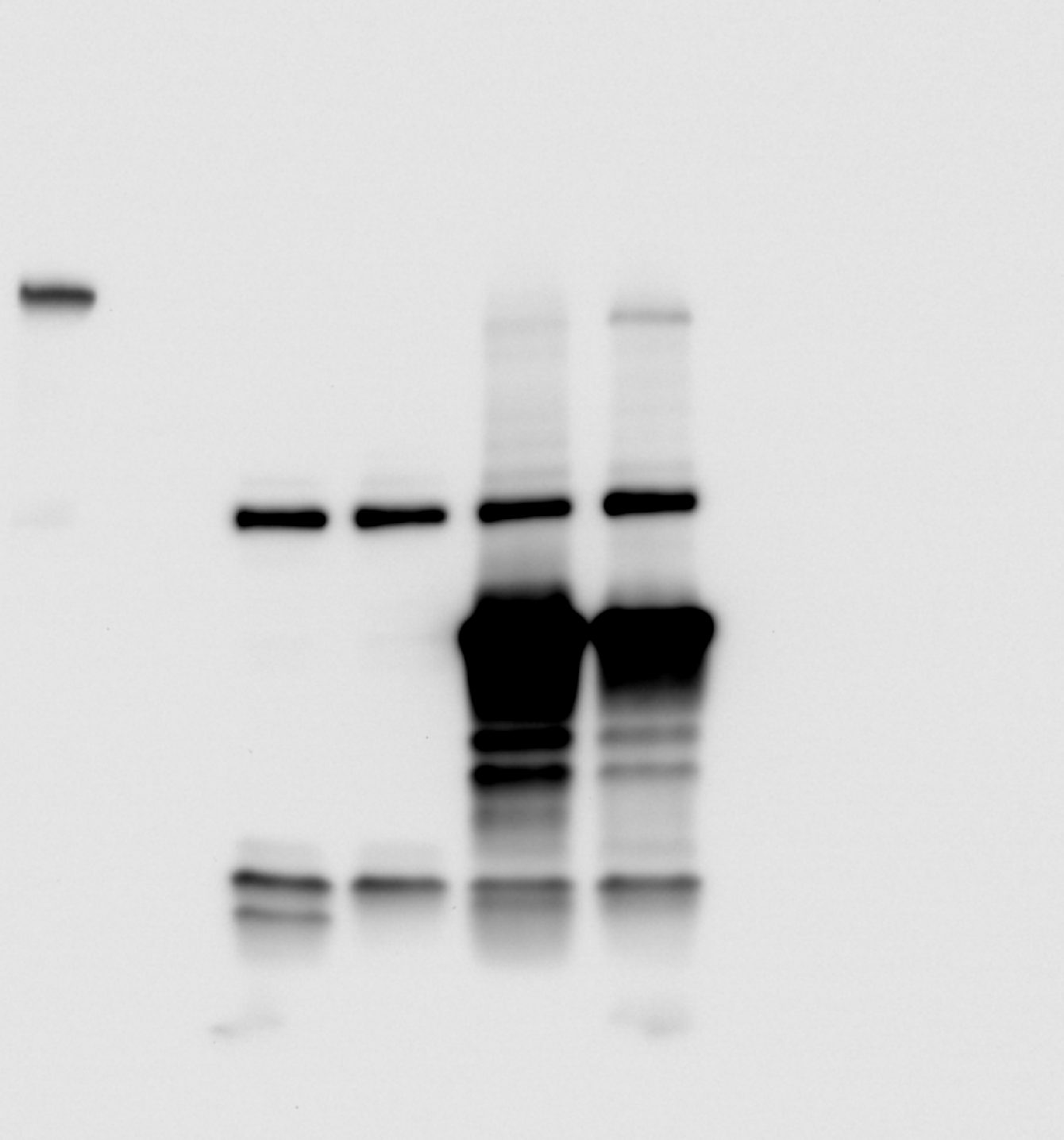

Supplement: Figure 1—figure supplement 5—source data 1. — The tiff files correspond to uncropped pictures of the chemiluminescent signal acquired on a BioRad Chemidoc. Two different immunoblotting of the same extracts were used for this figure (respectively labeled upper and lower). The regions used to generate the figure are highlighted for each immunoblot by back squares in the jpg files, which also contain at the bottom an overlay with a picture of the membrane to locate the protein ladder positions. [file elife-73913-fig1-figsupp5-data1.zip › Figure 1-figure supplement 5-source data 1/Fig.1-S5A-lower-HSD17B11.tif]

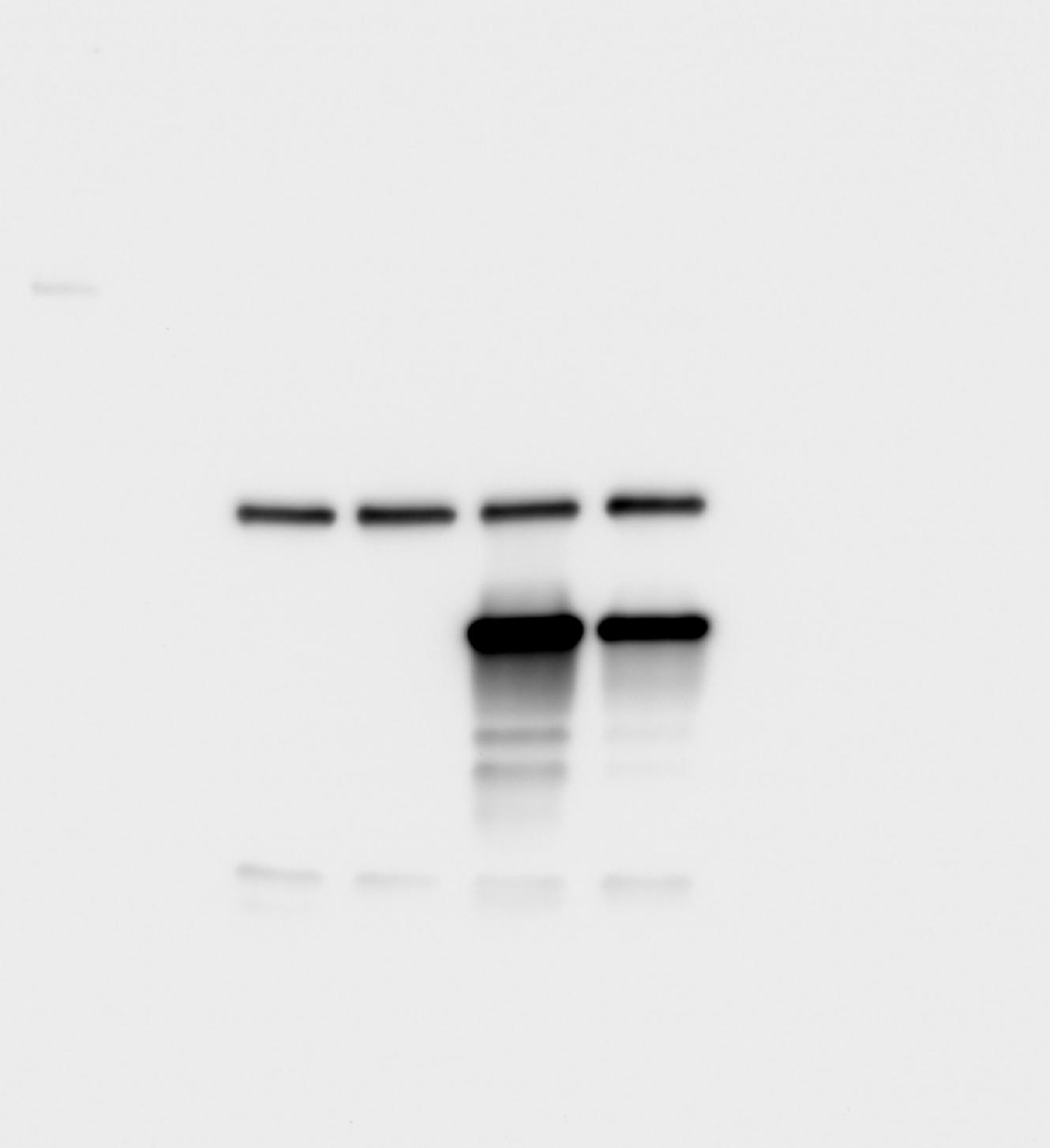

Supplement: Figure 1—figure supplement 5—source data 1. — The tiff files correspond to uncropped pictures of the chemiluminescent signal acquired on a BioRad Chemidoc. Two different immunoblotting of the same extracts were used for this figure (respectively labeled upper and lower). The regions used to generate the figure are highlighted for each immunoblot by back squares in the jpg files, which also contain at the bottom an overlay with a picture of the membrane to locate the protein ladder positions. [file elife-73913-fig1-figsupp5-data1.zip › Figure 1-figure supplement 5-source data 1/Fig.1-S5A-lower-SAFA.tif]

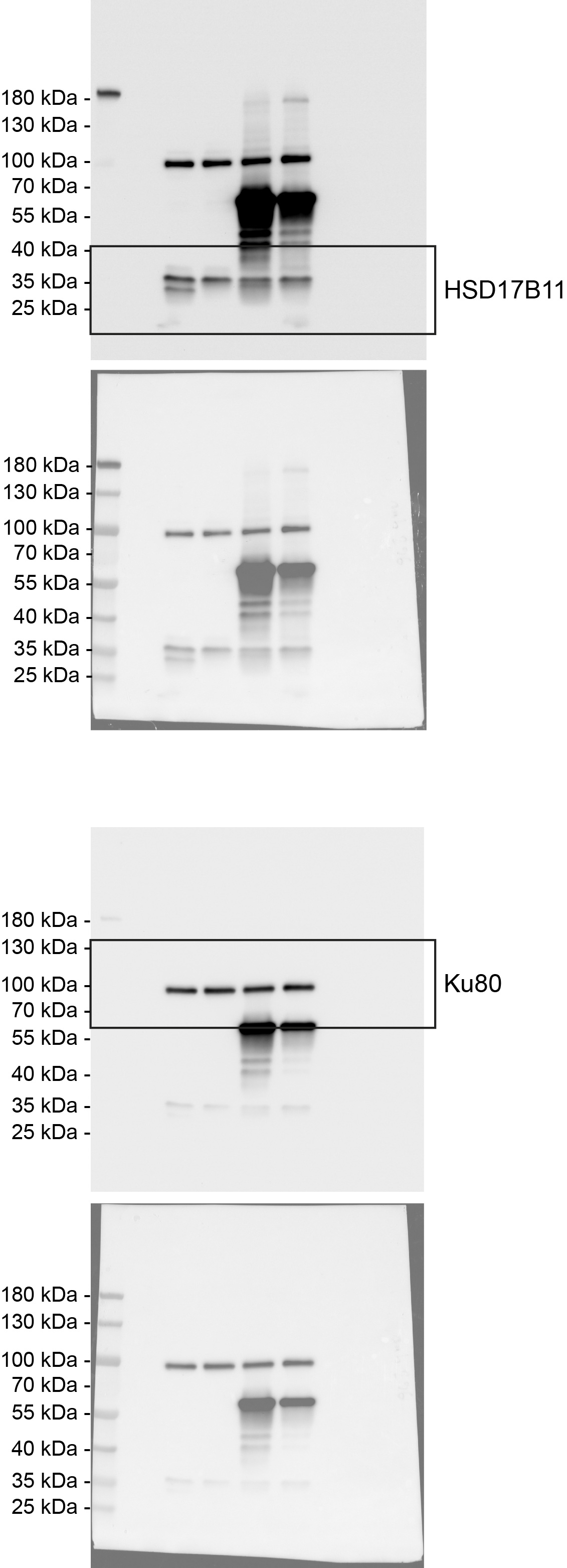

Supplement: Figure 1—figure supplement 5—source data 1. — The tiff files correspond to uncropped pictures of the chemiluminescent signal acquired on a BioRad Chemidoc. Two different immunoblotting of the same extracts were used for this figure (respectively labeled upper and lower). The regions used to generate the figure are highlighted for each immunoblot by back squares in the jpg files, which also contain at the bottom an overlay with a picture of the membrane to locate the protein ladder positions. [file elife-73913-fig1-figsupp5-data1.zip › Figure 1-figure supplement 5-source data 1/Fig.1-S5A-Lower.jpg]

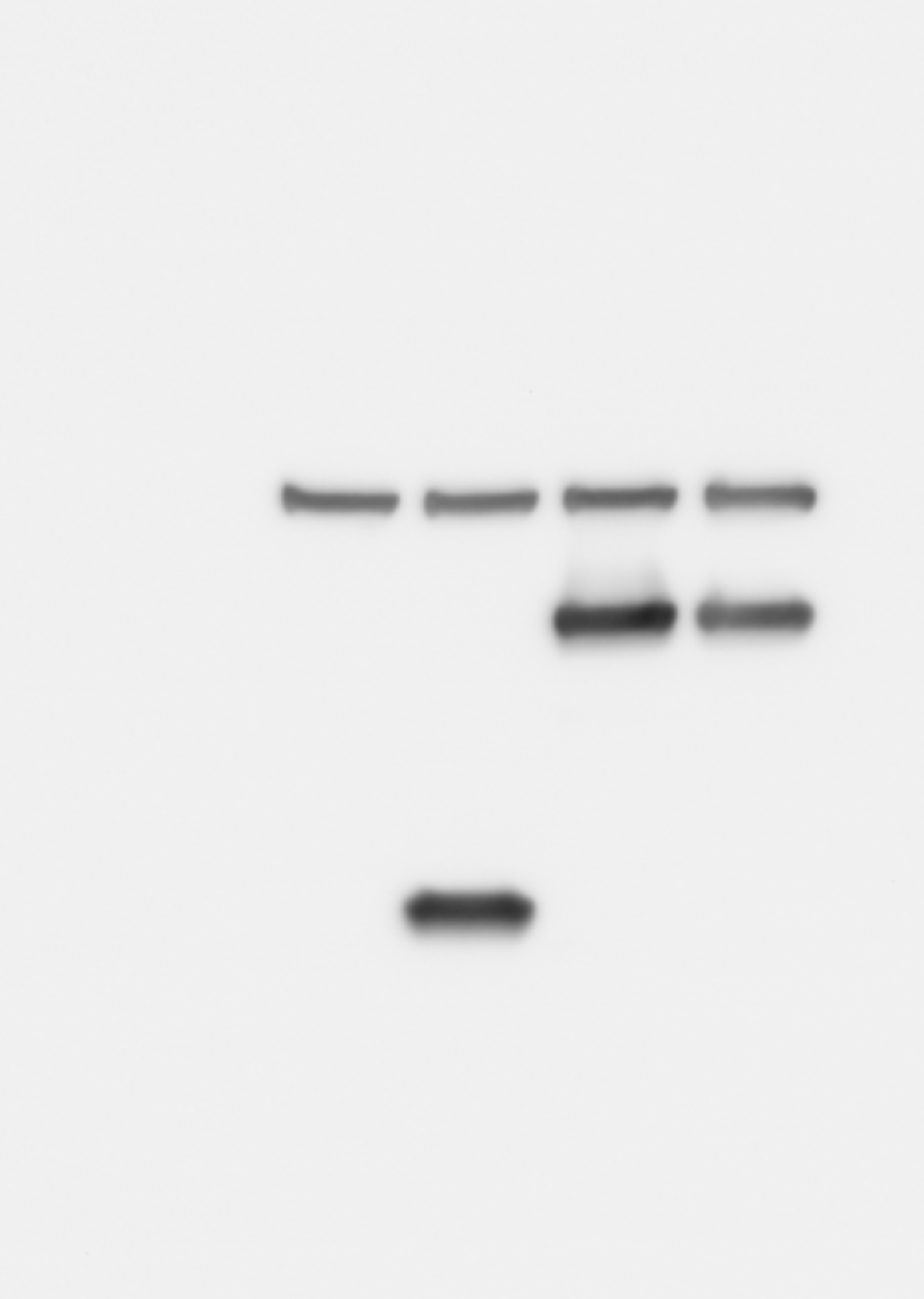

Supplement: Figure 1—figure supplement 5—source data 1. — The tiff files correspond to uncropped pictures of the chemiluminescent signal acquired on a BioRad Chemidoc. Two different immunoblotting of the same extracts were used for this figure (respectively labeled upper and lower). The regions used to generate the figure are highlighted for each immunoblot by back squares in the jpg files, which also contain at the bottom an overlay with a picture of the membrane to locate the protein ladder positions. [file elife-73913-fig1-figsupp5-data1.zip › Figure 1-figure supplement 5-source data 1/Fig.1-S5A-upper-GFP-Ku80.tif]

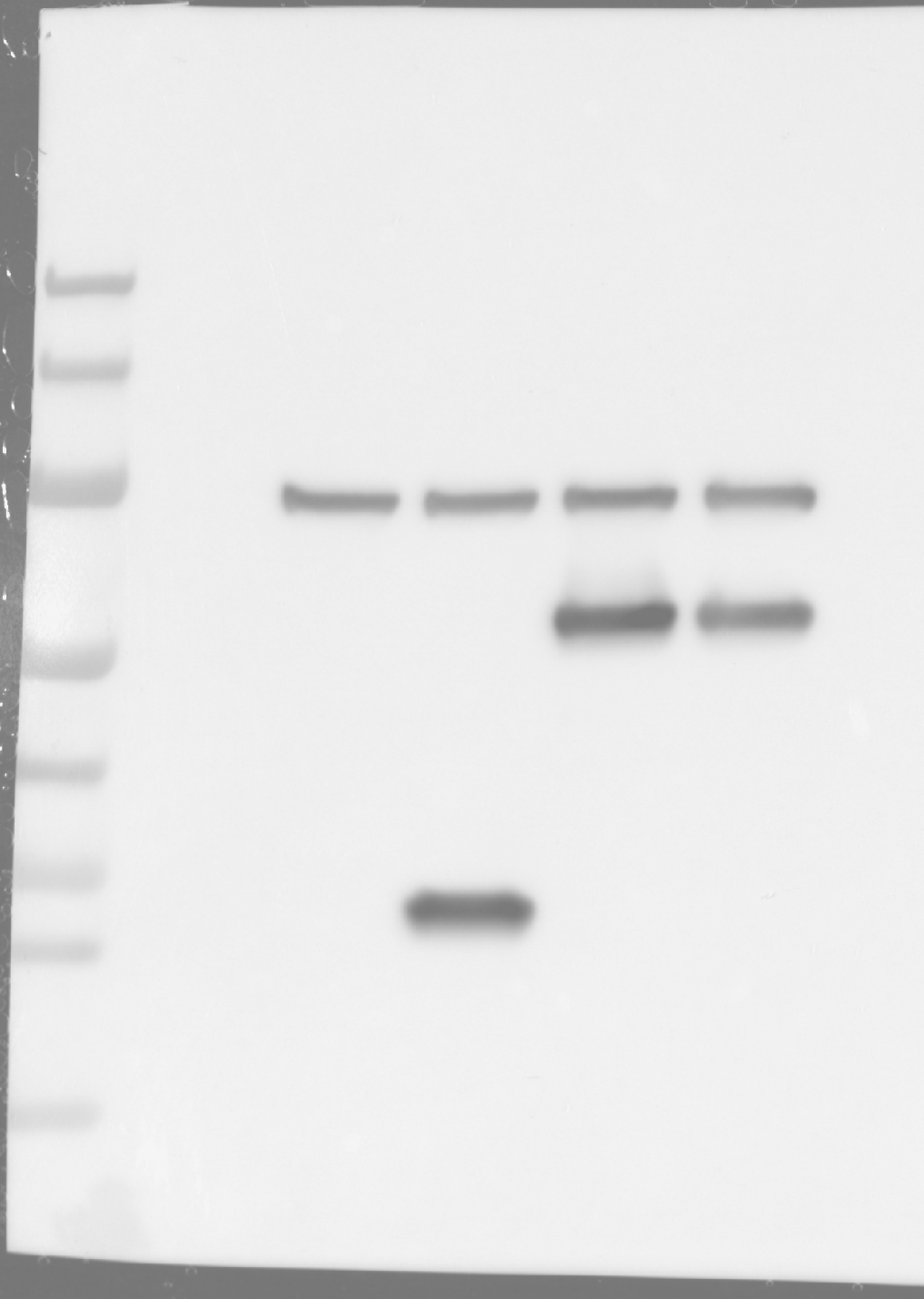

Supplement: Figure 1—figure supplement 5—source data 1. — The tiff files correspond to uncropped pictures of the chemiluminescent signal acquired on a BioRad Chemidoc. Two different immunoblotting of the same extracts were used for this figure (respectively labeled upper and lower). The regions used to generate the figure are highlighted for each immunoblot by back squares in the jpg files, which also contain at the bottom an overlay with a picture of the membrane to locate the protein ladder positions. [file elife-73913-fig1-figsupp5-data1.zip › Figure 1-figure supplement 5-source data 1/Fig.1-S5A-upper-Merge1.tif]

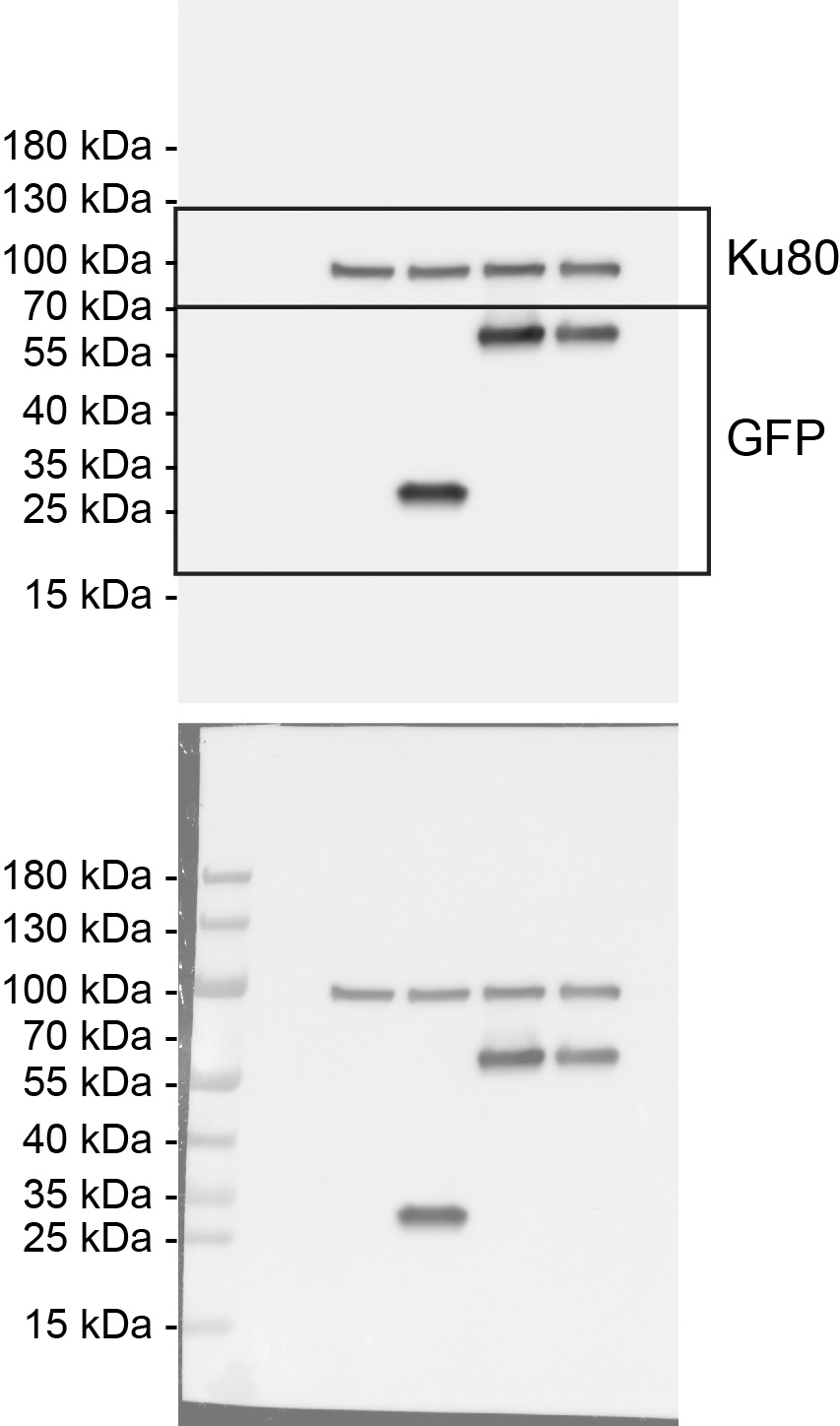

Supplement: Figure 1—figure supplement 5—source data 1. — The tiff files correspond to uncropped pictures of the chemiluminescent signal acquired on a BioRad Chemidoc. Two different immunoblotting of the same extracts were used for this figure (respectively labeled upper and lower). The regions used to generate the figure are highlighted for each immunoblot by back squares in the jpg files, which also contain at the bottom an overlay with a picture of the membrane to locate the protein ladder positions. [file elife-73913-fig1-figsupp5-data1.zip › Figure 1-figure supplement 5-source data 1/Fig.1-S5A-upper.jpg]

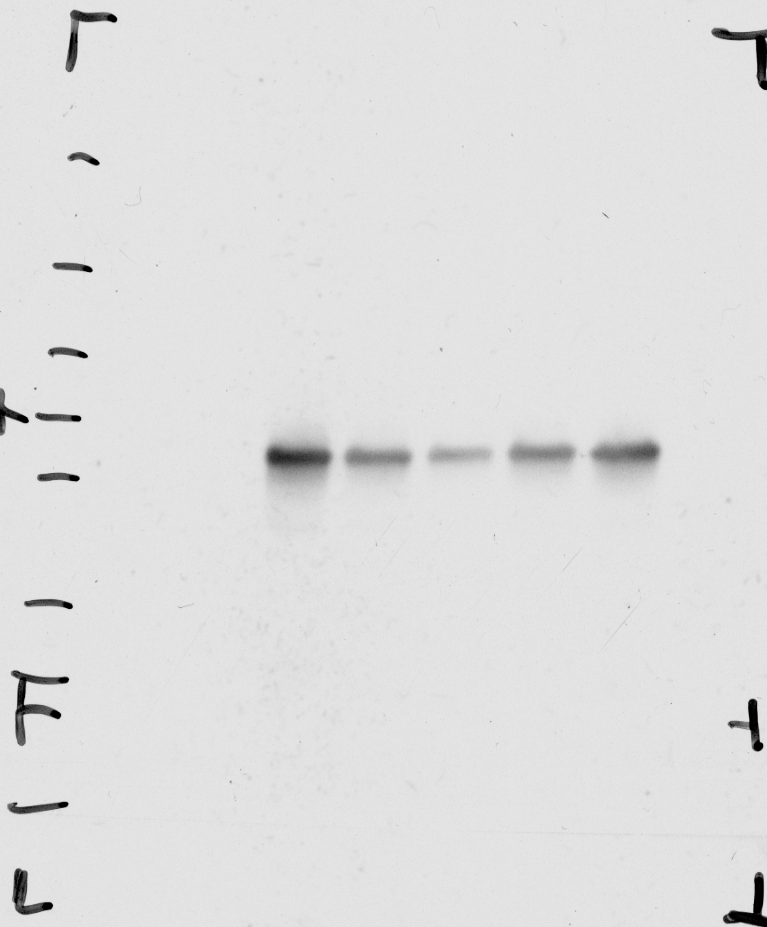

Supplement: Figure 1—figure supplement 5—source data 2. — The tiff files correspond to uncropped pictures of the chemiluminescent signal acquired using autoradiographic films. The regions used to generate the figure are highlighted for each immunoblot by back squares in the jpg files. [file elife-73913-fig1-figsupp5-data2.zip › Figure 1-figure supplement 5-source data 2/Fig.1-S5C-GFP.tif]

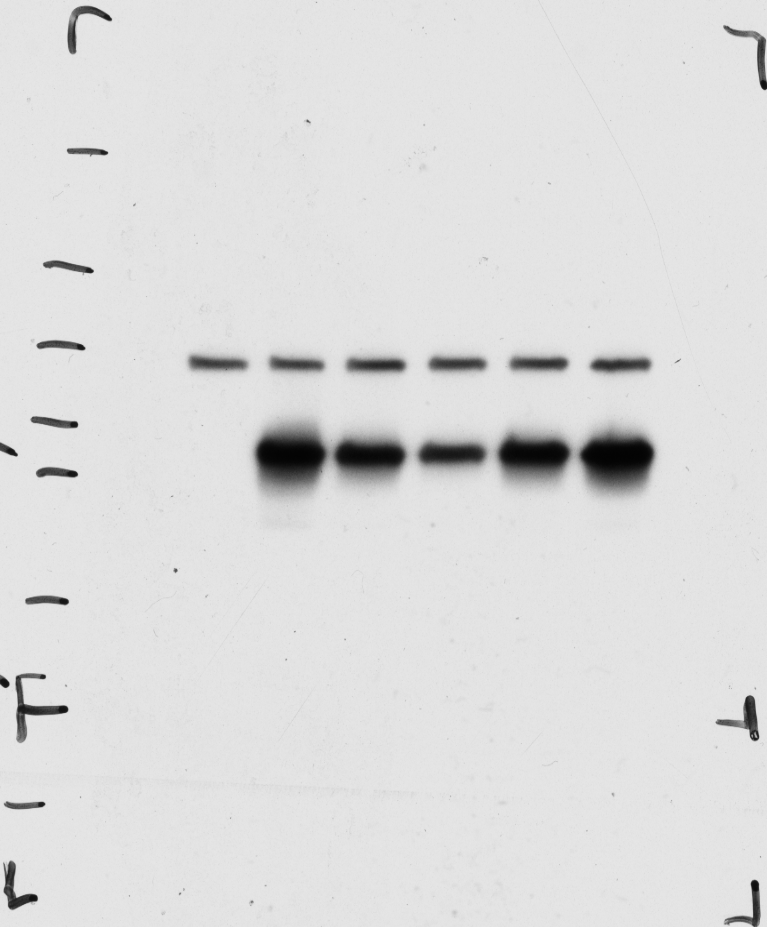

Supplement: Figure 1—figure supplement 5—source data 2. — The tiff files correspond to uncropped pictures of the chemiluminescent signal acquired using autoradiographic films. The regions used to generate the figure are highlighted for each immunoblot by back squares in the jpg files. [file elife-73913-fig1-figsupp5-data2.zip › Figure 1-figure supplement 5-source data 2/Fig.1-S5C-Ku80.tif]

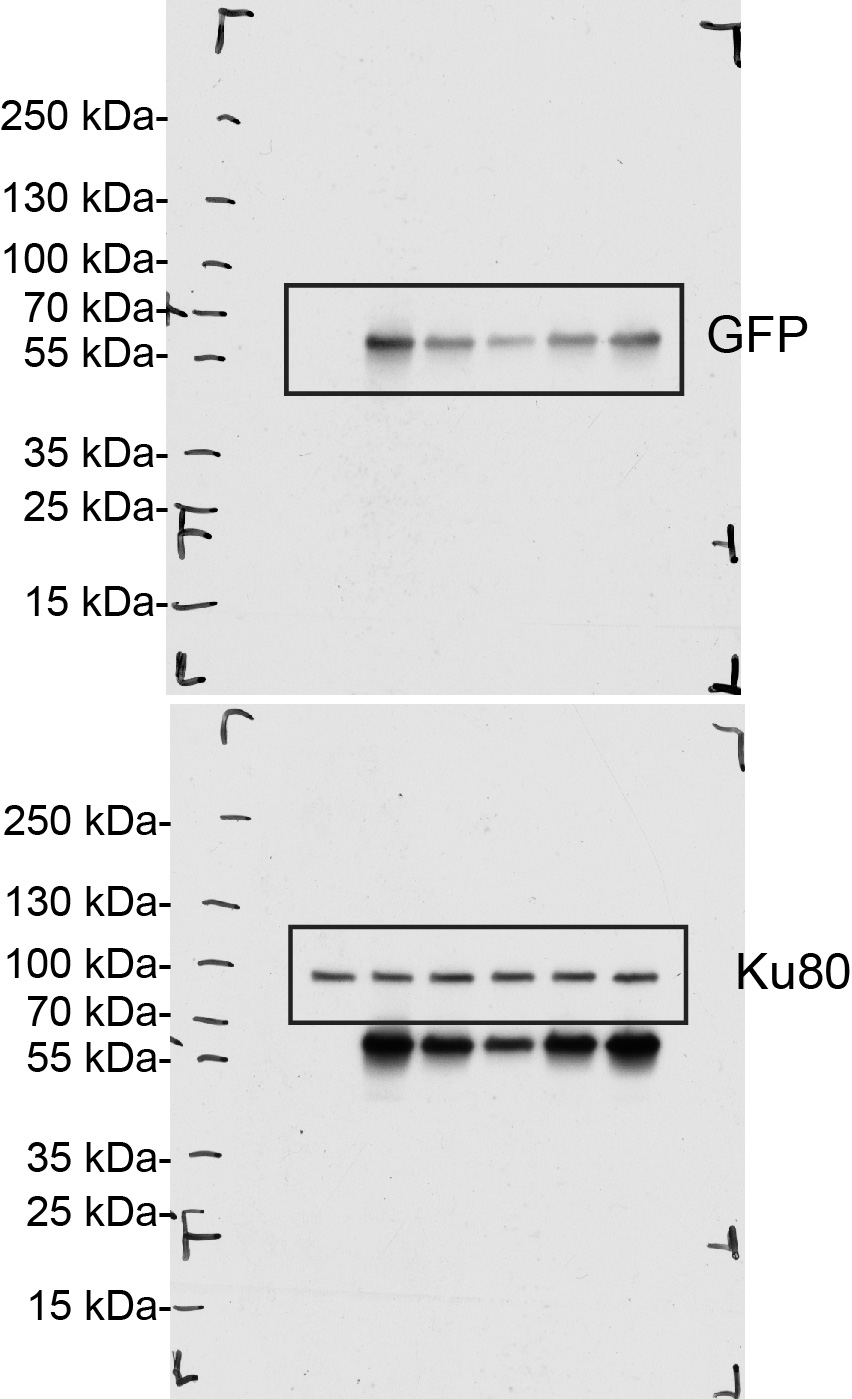

Supplement: Figure 1—figure supplement 5—source data 2. — The tiff files correspond to uncropped pictures of the chemiluminescent signal acquired using autoradiographic films. The regions used to generate the figure are highlighted for each immunoblot by back squares in the jpg files. [file elife-73913-fig1-figsupp5-data2.zip › Figure 1-figure supplement 5-source data 2/Fig.1-S5C.jpg]

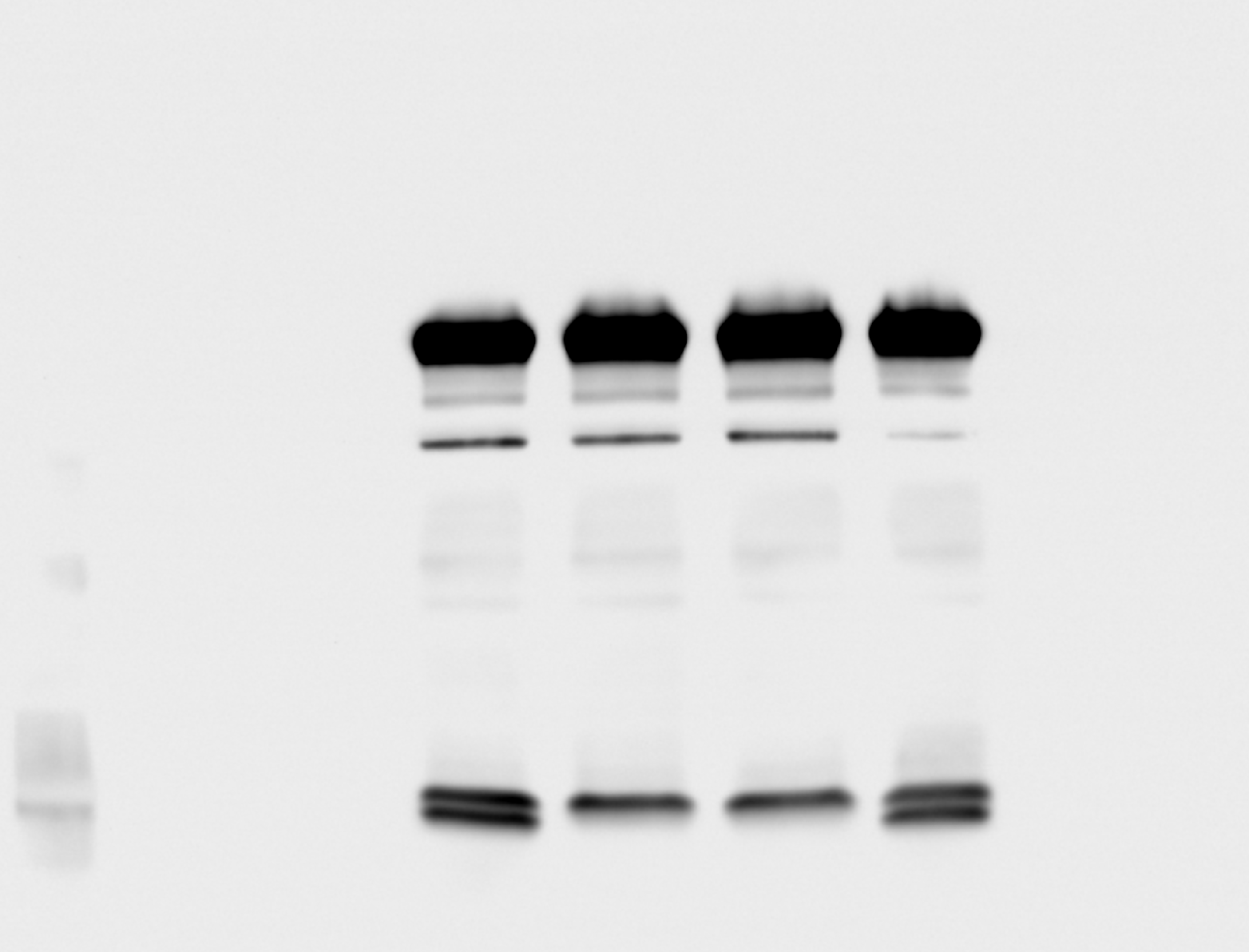

Supplement: Figure 1—figure supplement 5—source data 3. — The tiff files correspond to uncropped pictures of the chemiluminescent signal acquired on a BioRad Chemidoc. The regions used to generate the figure are highlighted by back squares in the jpg file, which also contains at the bottom an overlay with a picture of the membrane to locate the protein ladder positions. [file elife-73913-fig1-figsupp5-data3.zip › Figure 1-figure supplement 5-source data 3/Fig.1-S5E-HSD17B11.tif]

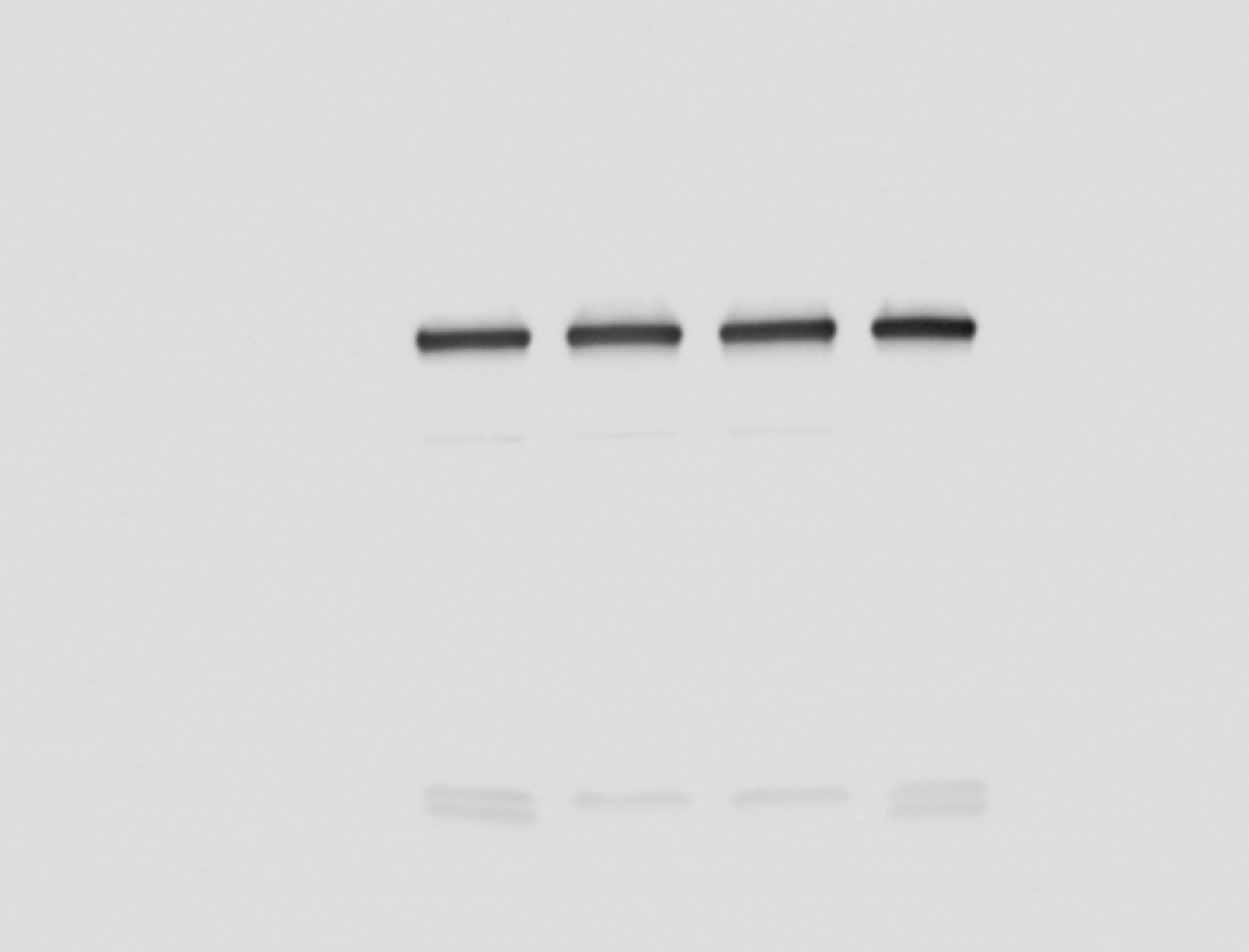

Supplement: Figure 1—figure supplement 5—source data 3. — The tiff files correspond to uncropped pictures of the chemiluminescent signal acquired on a BioRad Chemidoc. The regions used to generate the figure are highlighted by back squares in the jpg file, which also contains at the bottom an overlay with a picture of the membrane to locate the protein ladder positions. [file elife-73913-fig1-figsupp5-data3.zip › Figure 1-figure supplement 5-source data 3/Fig.1-S5E-SAFA.tif]

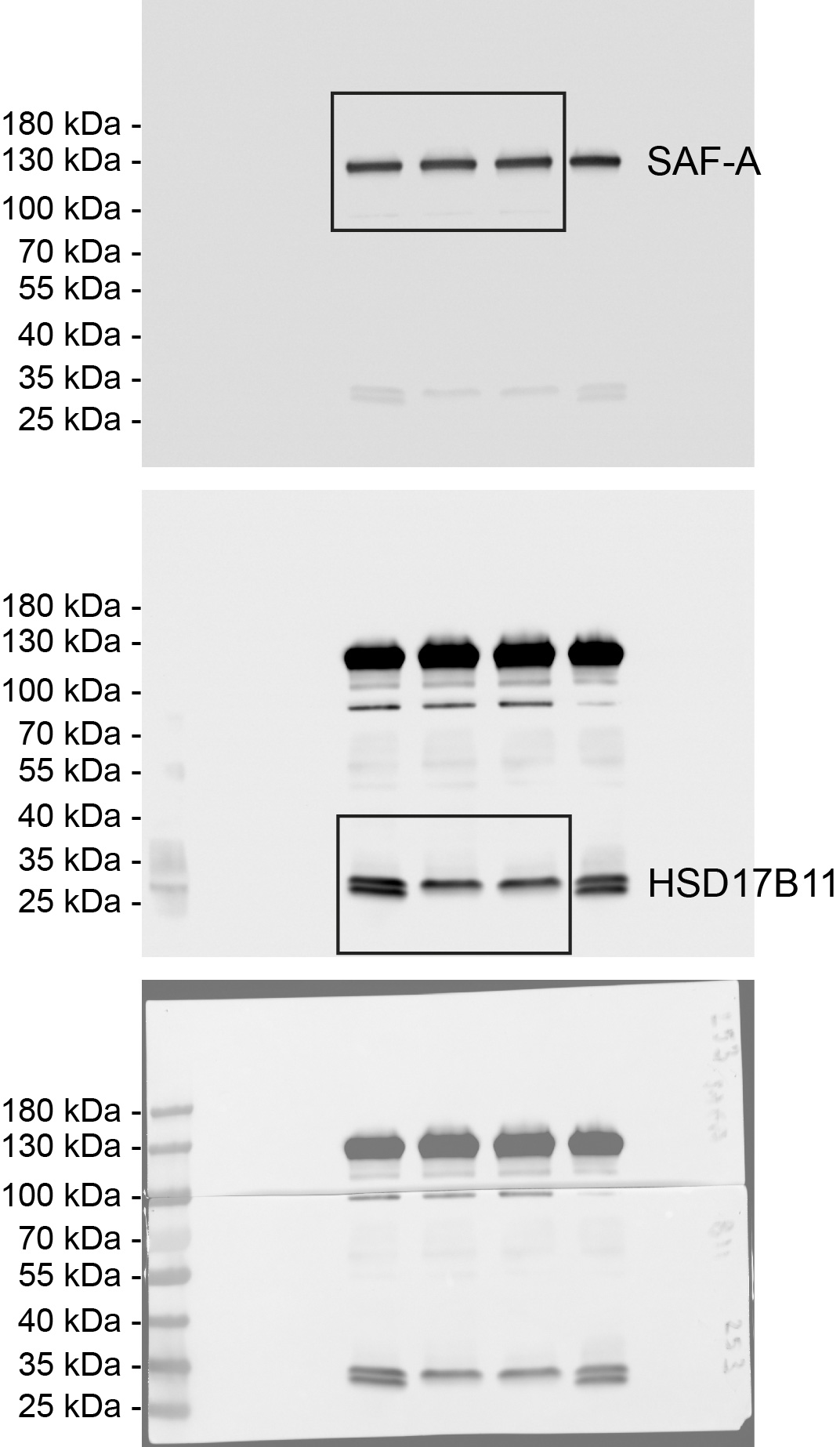

Supplement: Figure 1—figure supplement 5—source data 3. — The tiff files correspond to uncropped pictures of the chemiluminescent signal acquired on a BioRad Chemidoc. The regions used to generate the figure are highlighted by back squares in the jpg file, which also contains at the bottom an overlay with a picture of the membrane to locate the protein ladder positions. [file elife-73913-fig1-figsupp5-data3.zip › Figure 1-figure supplement 5-source data 3/Fig.1-S5E.jpg]

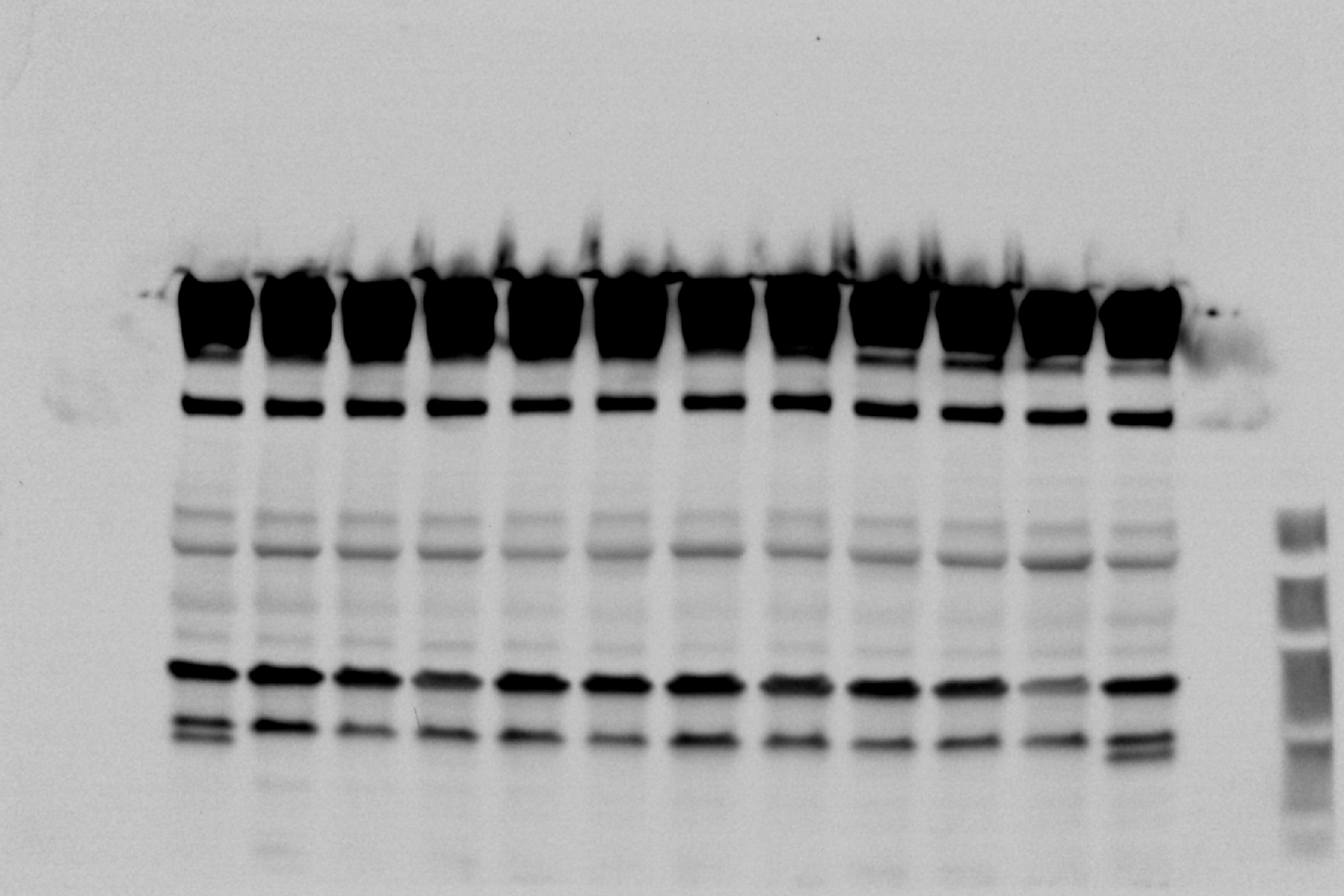

Supplement: Figure 1—figure supplement 5—source data 4. — The tiff files correspond to uncropped pictures of the chemiluminescent signal acquired on a BioRad Chemidoc. The regions used to generate the figure are highlighted by back squares in the jpg file, which also contains at the bottom an overlay with a picture of the membrane to locate the protein ladder positions. [file elife-73913-fig1-figsupp5-data4.zip › Figure 1-figure supplement 5-source data 4/Fig.1-S5G-HSD17B11.tif]

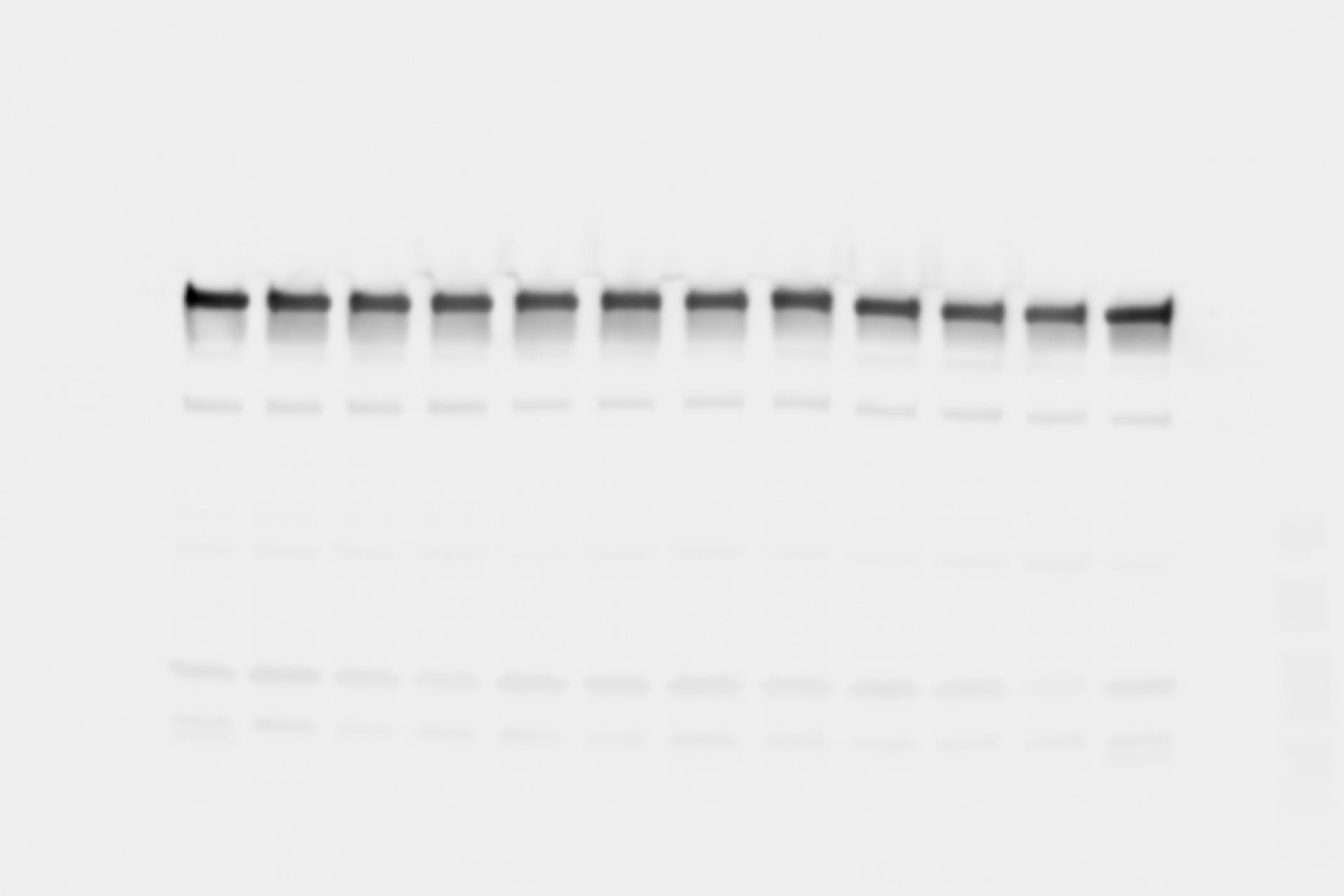

Supplement: Figure 1—figure supplement 5—source data 4. — The tiff files correspond to uncropped pictures of the chemiluminescent signal acquired on a BioRad Chemidoc. The regions used to generate the figure are highlighted by back squares in the jpg file, which also contains at the bottom an overlay with a picture of the membrane to locate the protein ladder positions. [file elife-73913-fig1-figsupp5-data4.zip › Figure 1-figure supplement 5-source data 4/Fig.1-S5G-SAFA.tif]

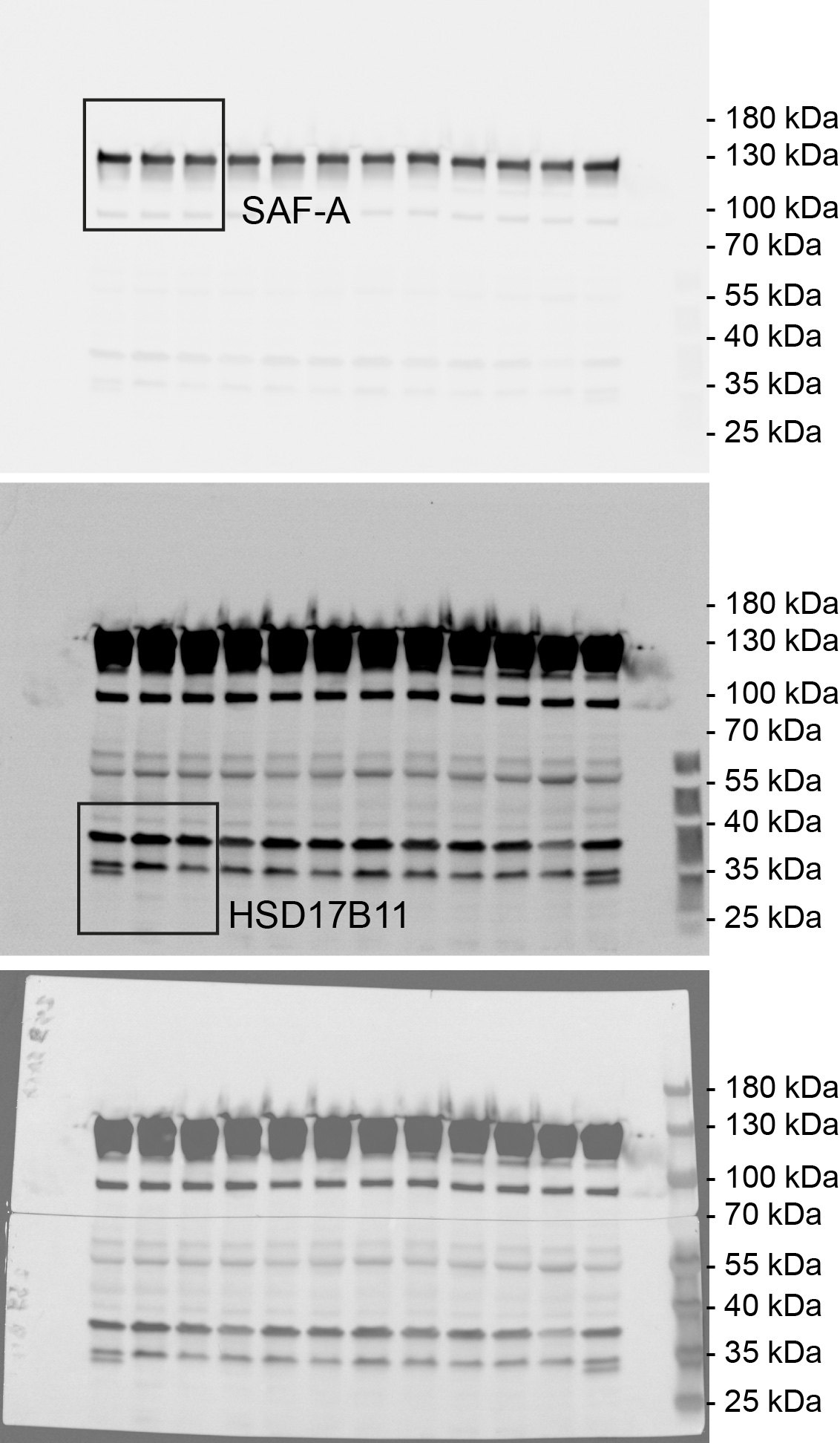

Supplement: Figure 1—figure supplement 5—source data 4. — The tiff files correspond to uncropped pictures of the chemiluminescent signal acquired on a BioRad Chemidoc. The regions used to generate the figure are highlighted by back squares in the jpg file, which also contains at the bottom an overlay with a picture of the membrane to locate the protein ladder positions. [file elife-73913-fig1-figsupp5-data4.zip › Figure 1-figure supplement 5-source data 4/Fig.1-S5G.jpg]

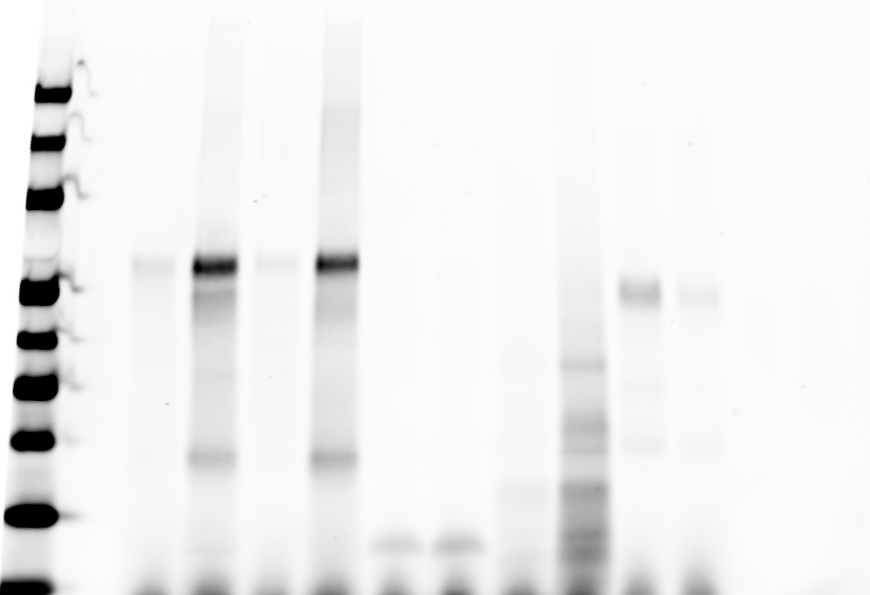

Supplement: Figure 2—source data 1. — The tiff files correspond to an uncropped picture of the AlexaFluor647 fluorescence signal, acquired on an Odyssey LI-COR, and of a scan of the membrane stained with Ponceau S. The jpg file combines both pictures and can be used to locate the protein ladders. [file elife-73913-fig2-data1.zip › Figure 2-source data 1/Fig.2D-Click-AF647.tif]

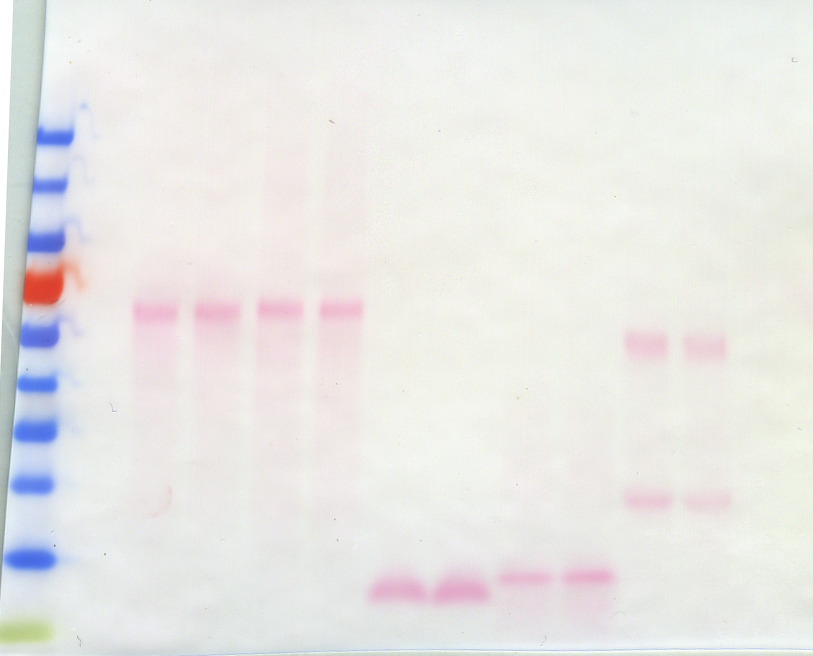

Supplement: Figure 2—source data 1. — The tiff files correspond to an uncropped picture of the AlexaFluor647 fluorescence signal, acquired on an Odyssey LI-COR, and of a scan of the membrane stained with Ponceau S. The jpg file combines both pictures and can be used to locate the protein ladders. [file elife-73913-fig2-data1.zip › Figure 2-source data 1/Fig.2D-Ponceau.tif]

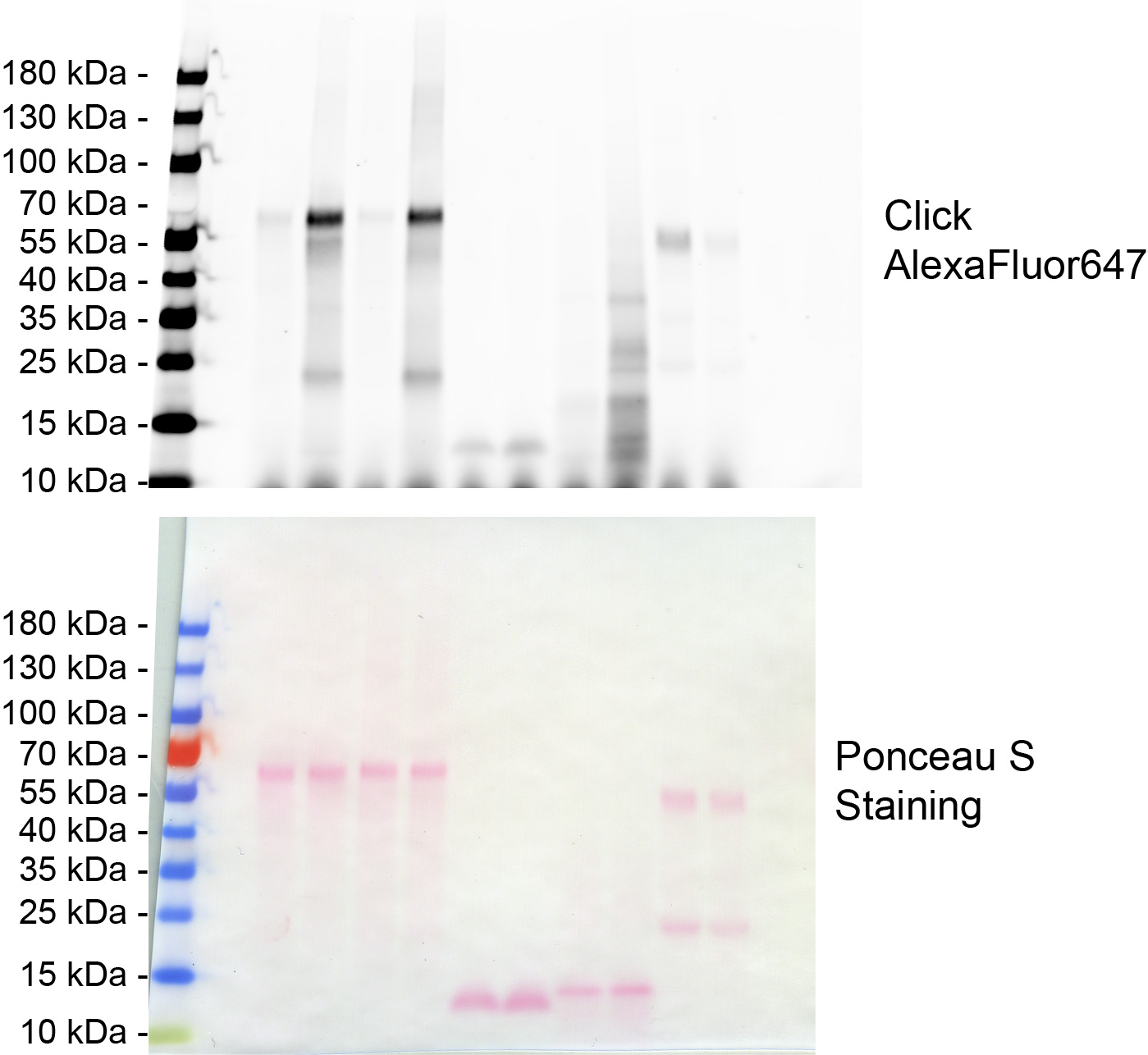

Supplement: Figure 2—source data 1. — The tiff files correspond to an uncropped picture of the AlexaFluor647 fluorescence signal, acquired on an Odyssey LI-COR, and of a scan of the membrane stained with Ponceau S. The jpg file combines both pictures and can be used to locate the protein ladders. [file elife-73913-fig2-data1.zip › Figure 2-source data 1/Fig.2D.jpg]

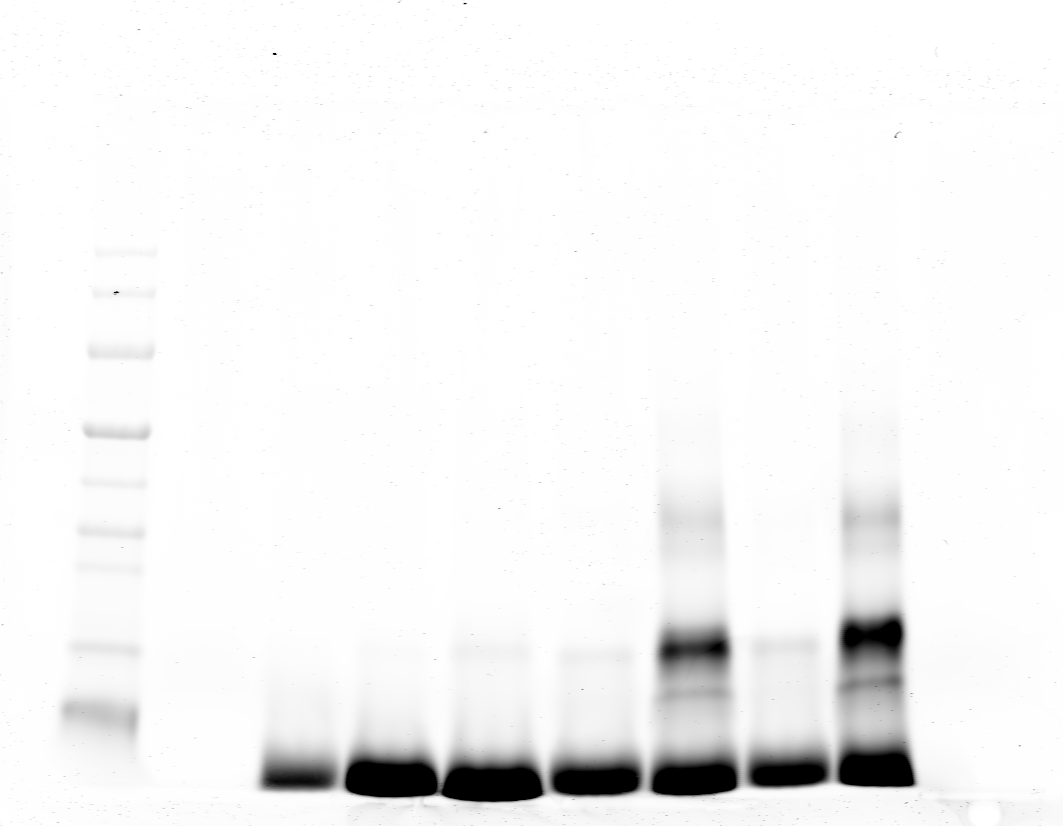

Supplement: Figure 2—source data 2. — The tiff files correspond to uncropped pictures of the AlexaFluor647 fluorescence, acquired in gel on an Odyssey LI-COR, and of the gels after staining with Coomassie blue scanned with the BioRad Chemidoc. Two different gels were used (respectively labeled upper and lower). Each jpg file combines the two pictures used to generate upper and lower parts of the figure. [file elife-73913-fig2-data2.zip › Figure 2-source data 2/Fig.2E-lower-Click-AF647.tif]

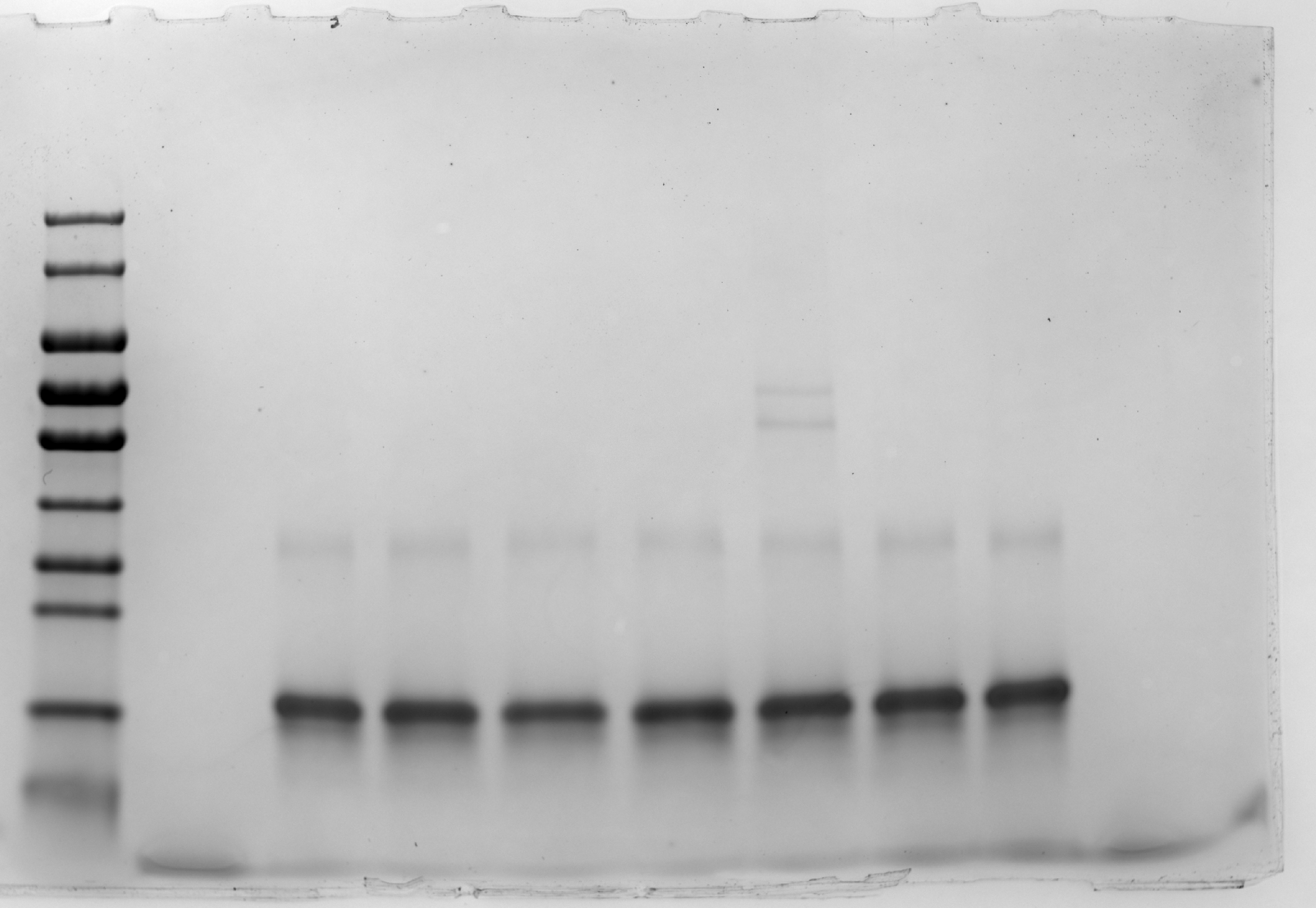

Supplement: Figure 2—source data 2. — The tiff files correspond to uncropped pictures of the AlexaFluor647 fluorescence, acquired in gel on an Odyssey LI-COR, and of the gels after staining with Coomassie blue scanned with the BioRad Chemidoc. Two different gels were used (respectively labeled upper and lower). Each jpg file combines the two pictures used to generate upper and lower parts of the figure. [file elife-73913-fig2-data2.zip › Figure 2-source data 2/Fig.2E-lower-Coomassie.tif]

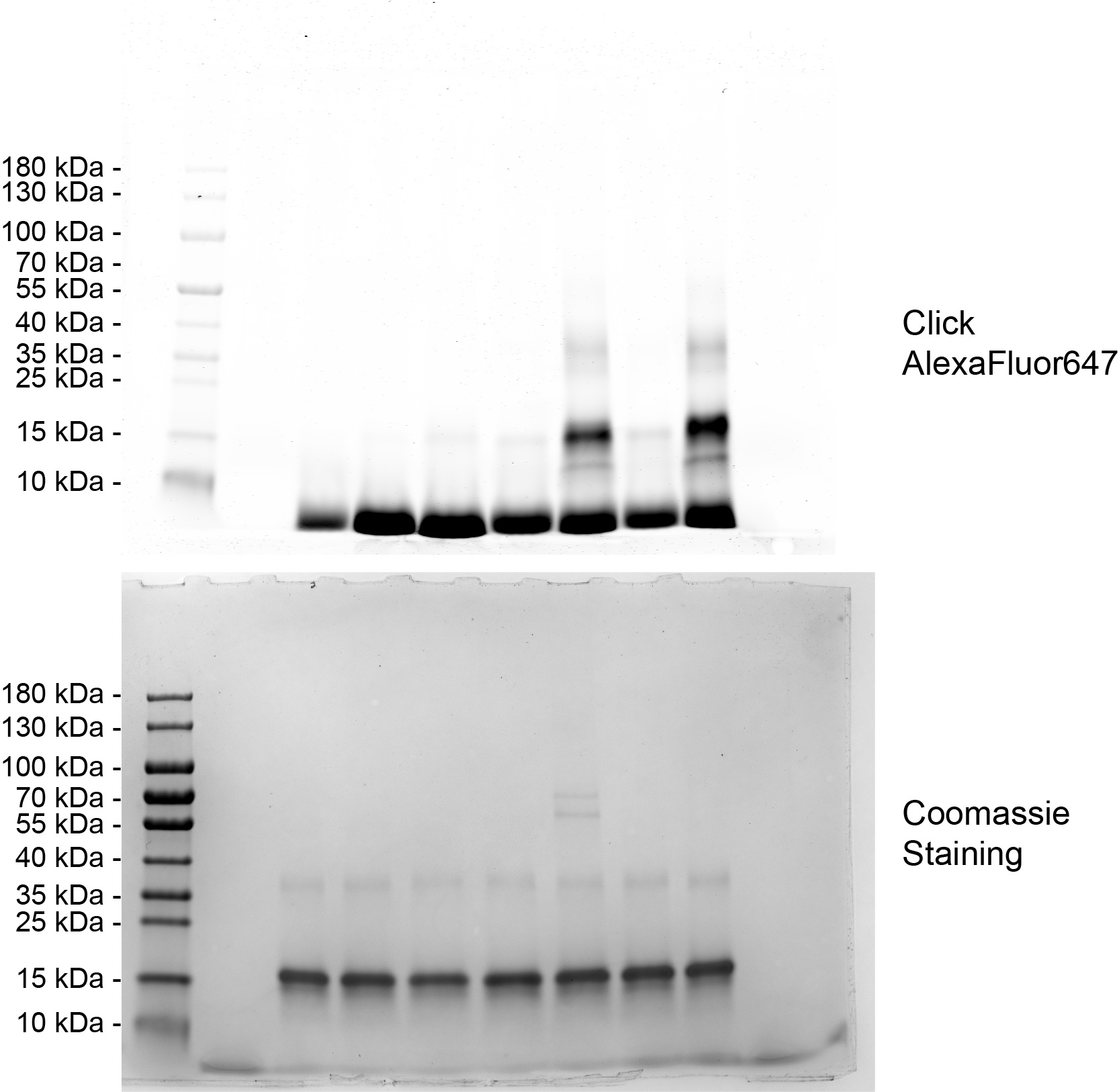

Supplement: Figure 2—source data 2. — The tiff files correspond to uncropped pictures of the AlexaFluor647 fluorescence, acquired in gel on an Odyssey LI-COR, and of the gels after staining with Coomassie blue scanned with the BioRad Chemidoc. Two different gels were used (respectively labeled upper and lower). Each jpg file combines the two pictures used to generate upper and lower parts of the figure. [file elife-73913-fig2-data2.zip › Figure 2-source data 2/Fig.2E-lower.jpg]

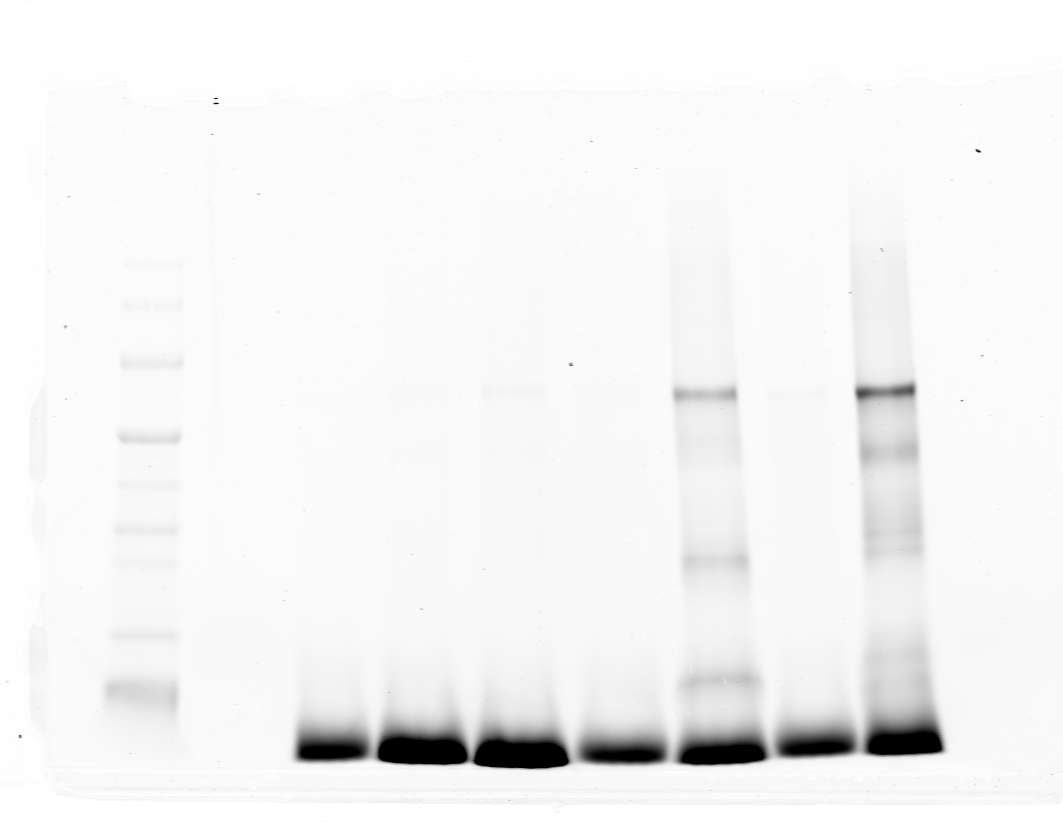

Supplement: Figure 2—source data 2. — The tiff files correspond to uncropped pictures of the AlexaFluor647 fluorescence, acquired in gel on an Odyssey LI-COR, and of the gels after staining with Coomassie blue scanned with the BioRad Chemidoc. Two different gels were used (respectively labeled upper and lower). Each jpg file combines the two pictures used to generate upper and lower parts of the figure. [file elife-73913-fig2-data2.zip › Figure 2-source data 2/Fig.2E-upper-Click-AF647.tif]

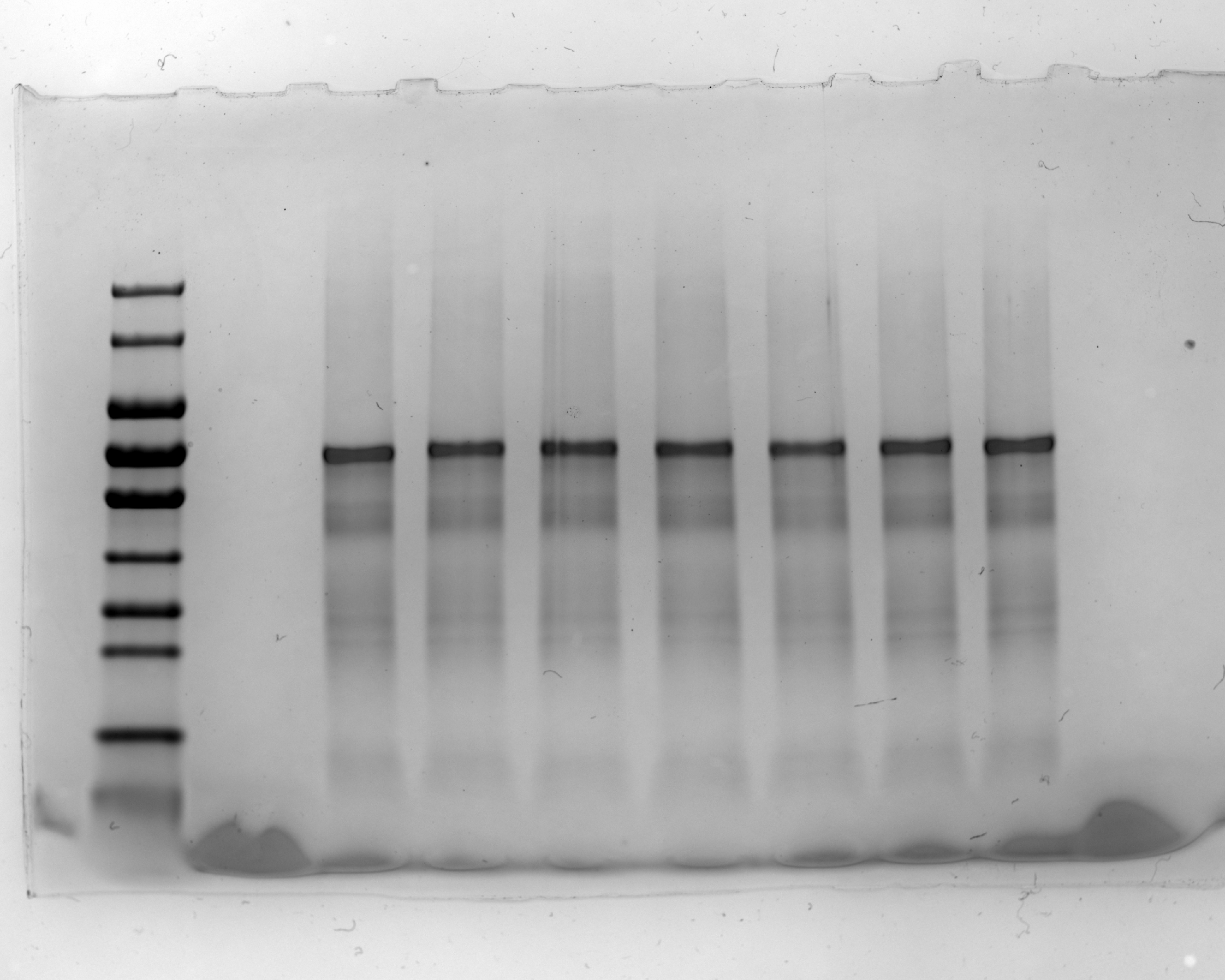

Supplement: Figure 2—source data 2. — The tiff files correspond to uncropped pictures of the AlexaFluor647 fluorescence, acquired in gel on an Odyssey LI-COR, and of the gels after staining with Coomassie blue scanned with the BioRad Chemidoc. Two different gels were used (respectively labeled upper and lower). Each jpg file combines the two pictures used to generate upper and lower parts of the figure. [file elife-73913-fig2-data2.zip › Figure 2-source data 2/Fig.2E-upper-Coomassie.tif]

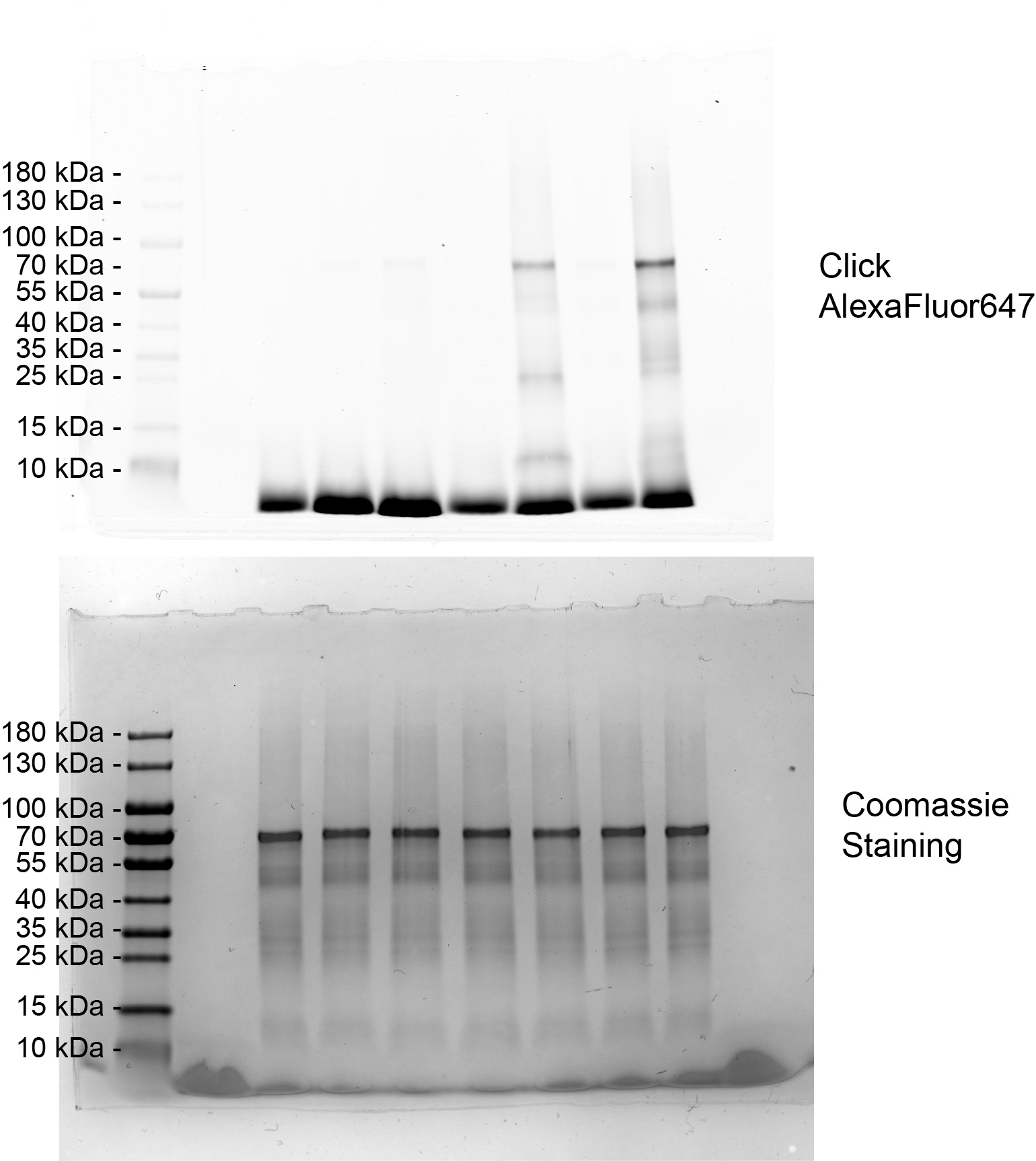

Supplement: Figure 2—source data 2. — The tiff files correspond to uncropped pictures of the AlexaFluor647 fluorescence, acquired in gel on an Odyssey LI-COR, and of the gels after staining with Coomassie blue scanned with the BioRad Chemidoc. Two different gels were used (respectively labeled upper and lower). Each jpg file combines the two pictures used to generate upper and lower parts of the figure. [file elife-73913-fig2-data2.zip › Figure 2-source data 2/Fig.2E-upper.jpg]

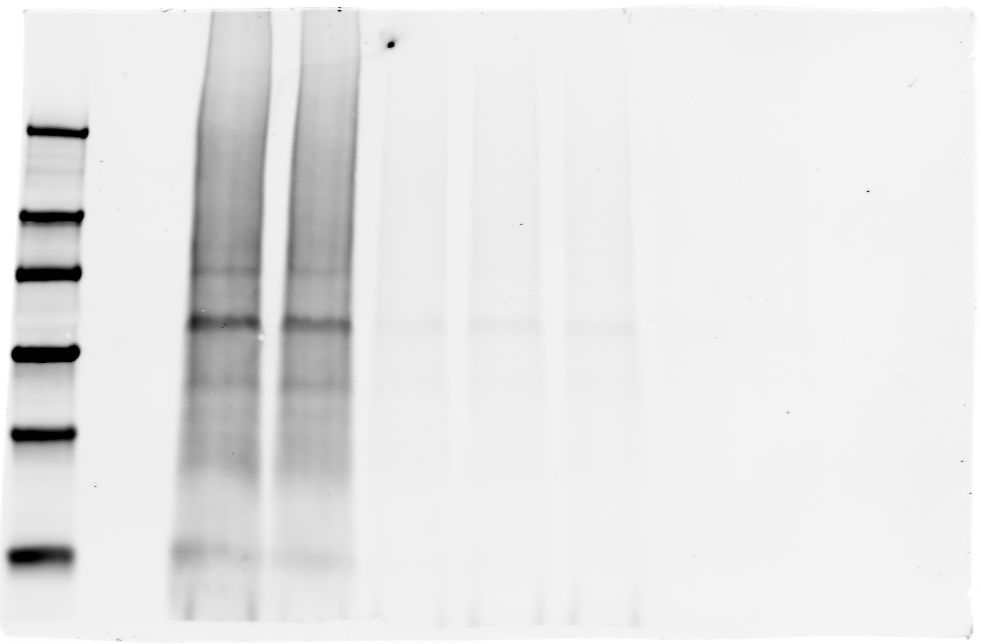

Supplement: Figure 2—source data 3. — The tiff files correspond to uncropped pictures of the AlexaFluor647 fluorescence signal, acquired on an Odyssey LI-COR, of a scan of the membrane stained with Ponceau S, for the upper part (supernatant), and of the chemiluminescent signal acquired using autoradiographic films for the lower part (beads). The jpg file combines the pictures used to generate the figure and can be used to locate the protein ladders. [file elife-73913-fig2-data3.zip › Figure 2-source data 3/Fig.2F-lower-Click-AF647.tif]

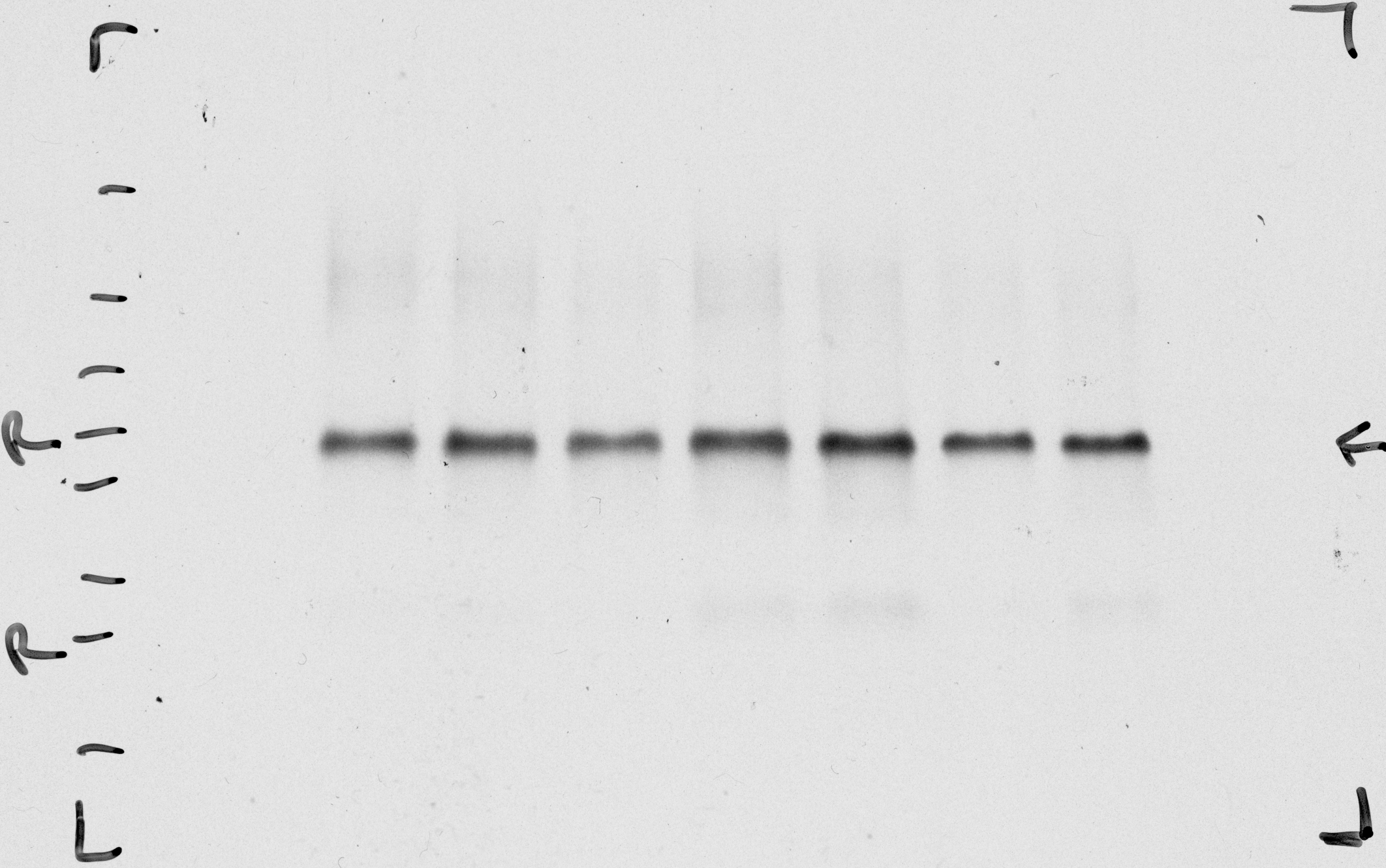

Supplement: Figure 2—source data 3. — The tiff files correspond to uncropped pictures of the AlexaFluor647 fluorescence signal, acquired on an Odyssey LI-COR, of a scan of the membrane stained with Ponceau S, for the upper part (supernatant), and of the chemiluminescent signal acquired using autoradiographic films for the lower part (beads). The jpg file combines the pictures used to generate the figure and can be used to locate the protein ladders. [file elife-73913-fig2-data3.zip › Figure 2-source data 3/Fig.2F-lower-GFP.tif]

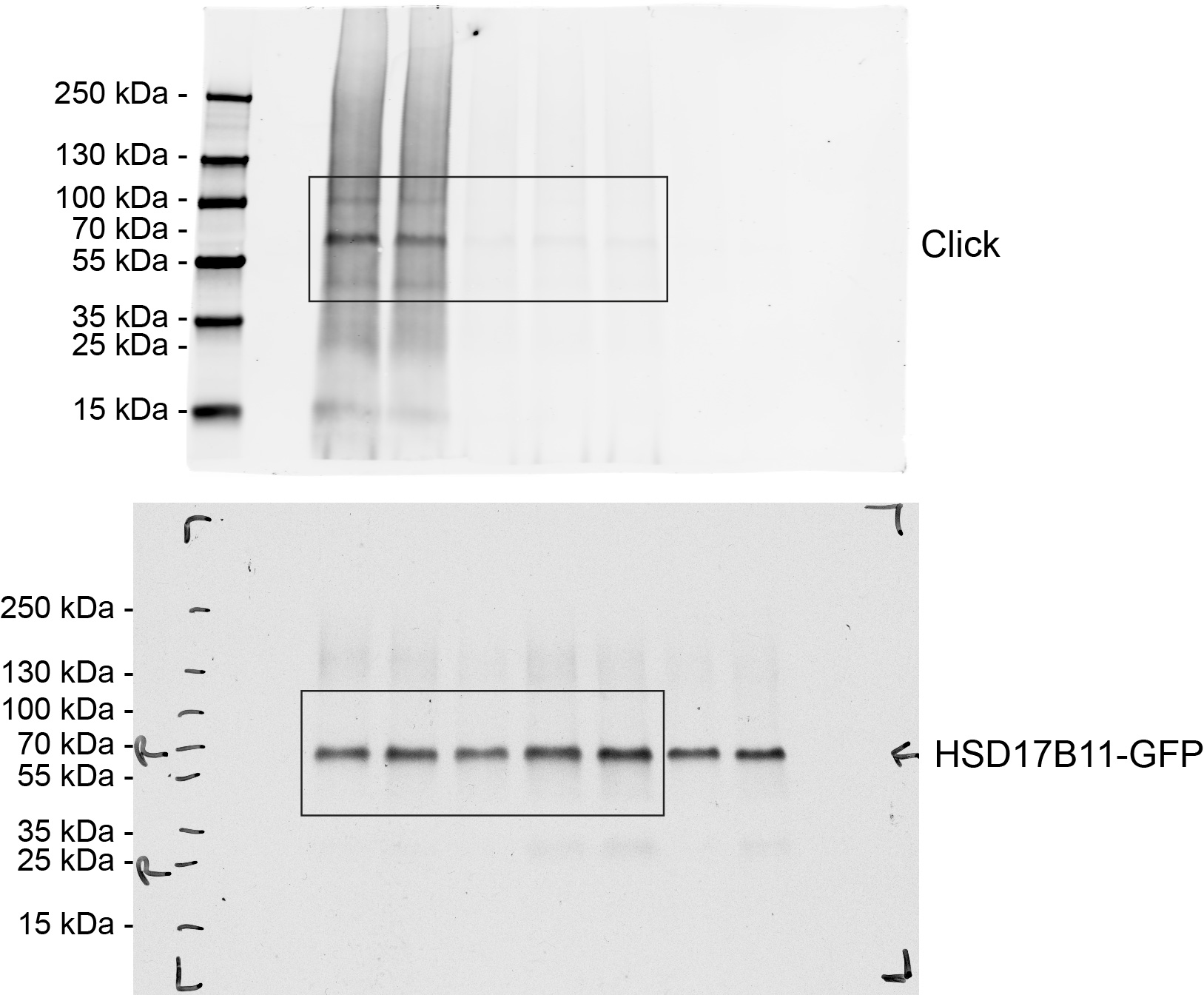

Supplement: Figure 2—source data 3. — The tiff files correspond to uncropped pictures of the AlexaFluor647 fluorescence signal, acquired on an Odyssey LI-COR, of a scan of the membrane stained with Ponceau S, for the upper part (supernatant), and of the chemiluminescent signal acquired using autoradiographic films for the lower part (beads). The jpg file combines the pictures used to generate the figure and can be used to locate the protein ladders. [file elife-73913-fig2-data3.zip › Figure 2-source data 3/Fig.2F-lower.jpg]

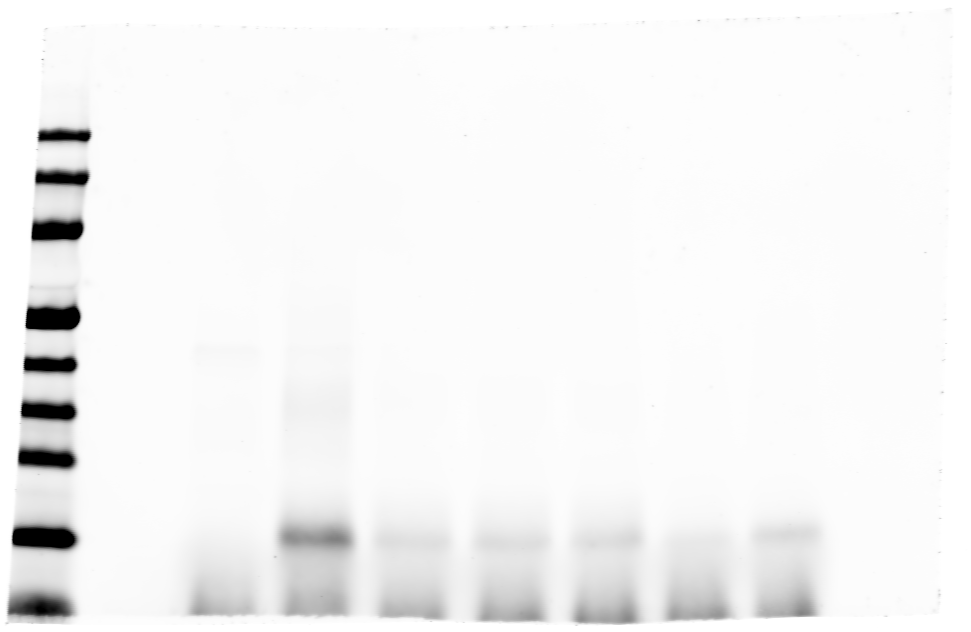

Supplement: Figure 2—source data 3. — The tiff files correspond to uncropped pictures of the AlexaFluor647 fluorescence signal, acquired on an Odyssey LI-COR, of a scan of the membrane stained with Ponceau S, for the upper part (supernatant), and of the chemiluminescent signal acquired using autoradiographic films for the lower part (beads). The jpg file combines the pictures used to generate the figure and can be used to locate the protein ladders. [file elife-73913-fig2-data3.zip › Figure 2-source data 3/Fig.2F-upper-Click-AF647.tif]

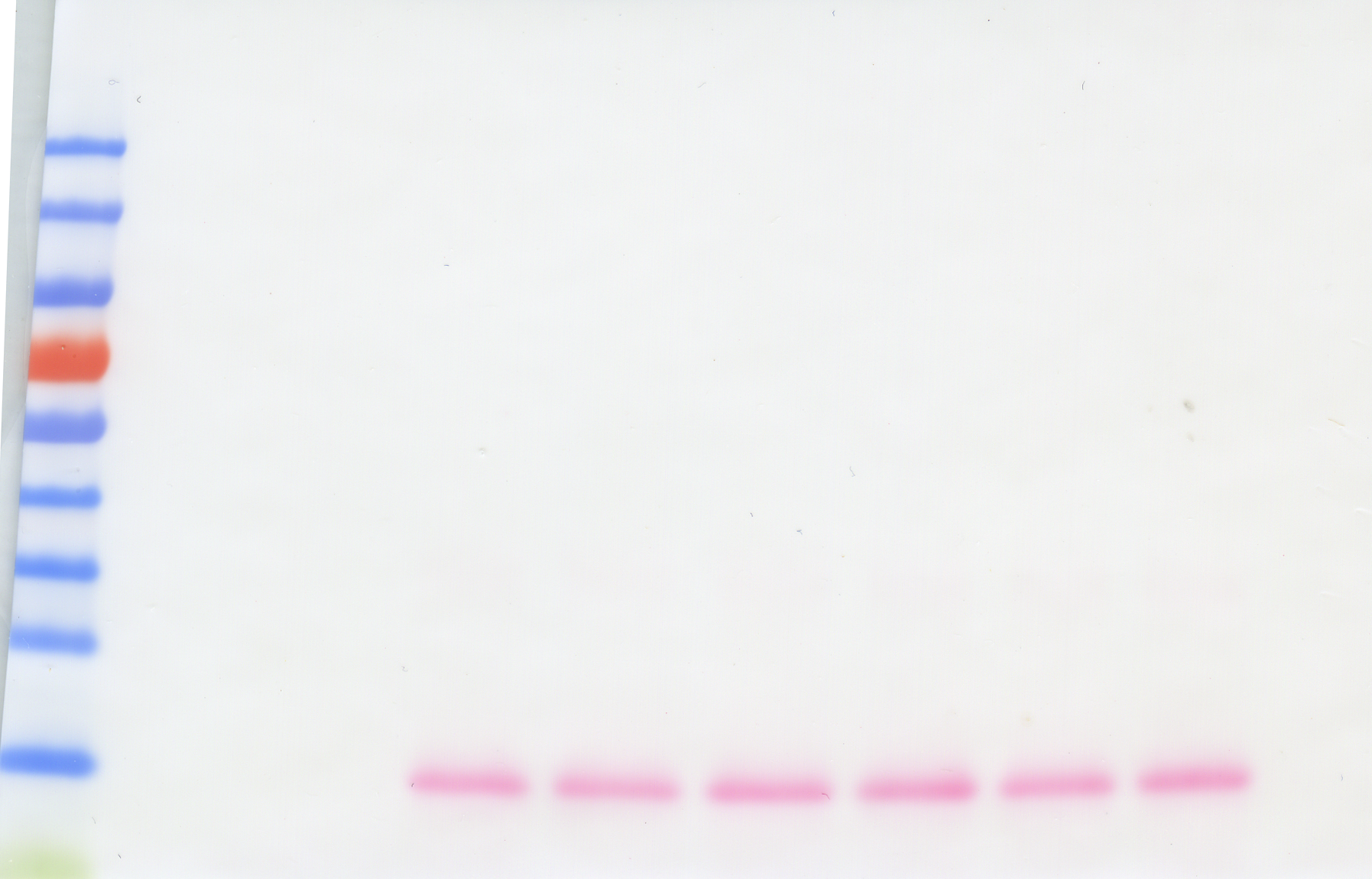

Supplement: Figure 2—source data 3. — The tiff files correspond to uncropped pictures of the AlexaFluor647 fluorescence signal, acquired on an Odyssey LI-COR, of a scan of the membrane stained with Ponceau S, for the upper part (supernatant), and of the chemiluminescent signal acquired using autoradiographic films for the lower part (beads). The jpg file combines the pictures used to generate the figure and can be used to locate the protein ladders. [file elife-73913-fig2-data3.zip › Figure 2-source data 3/Fig.2F-upper-Ponceau.tif]

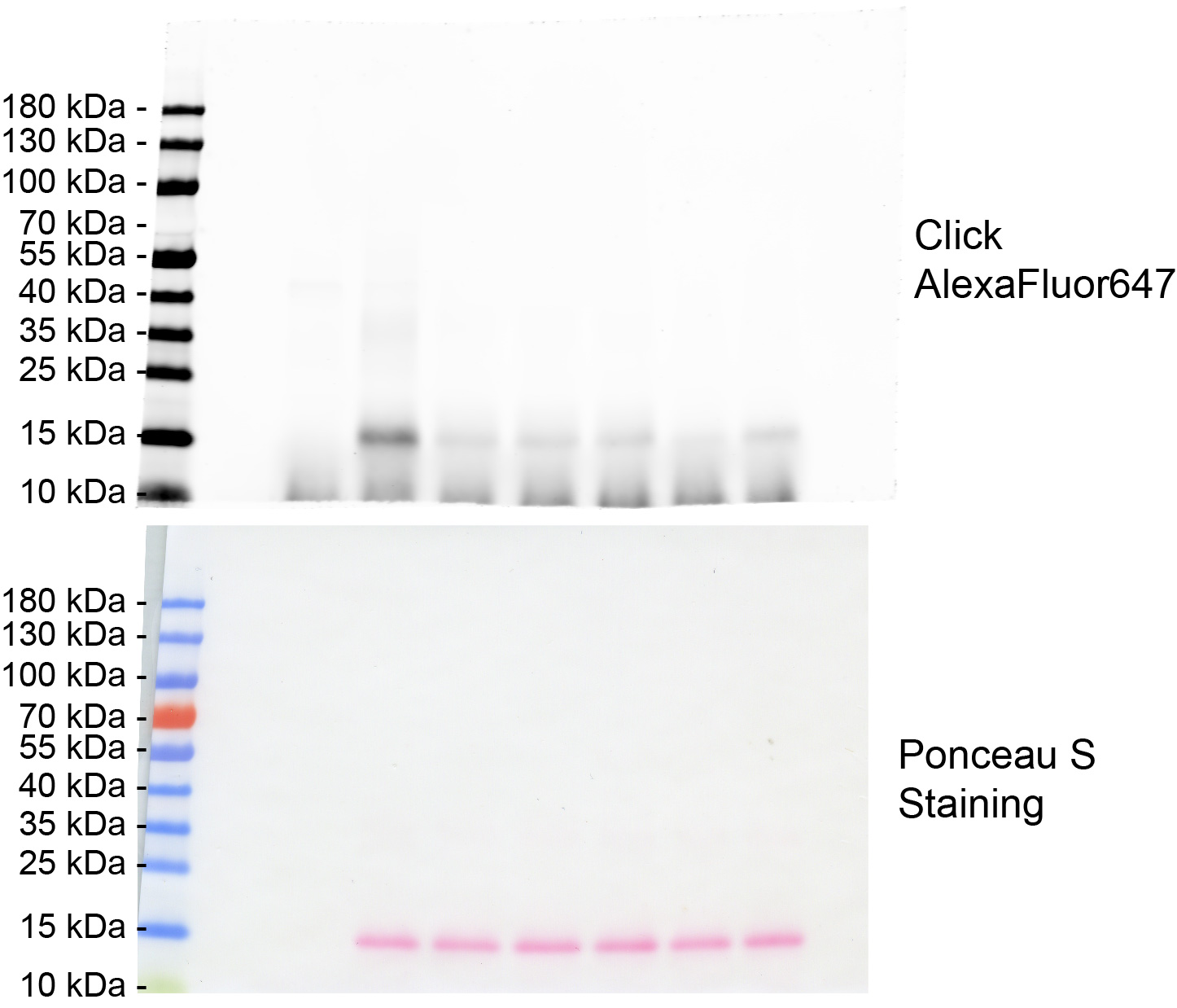

Supplement: Figure 2—source data 3. — The tiff files correspond to uncropped pictures of the AlexaFluor647 fluorescence signal, acquired on an Odyssey LI-COR, of a scan of the membrane stained with Ponceau S, for the upper part (supernatant), and of the chemiluminescent signal acquired using autoradiographic films for the lower part (beads). The jpg file combines the pictures used to generate the figure and can be used to locate the protein ladders. [file elife-73913-fig2-data3.zip › Figure 2-source data 3/Fig.2F-upper.jpg]

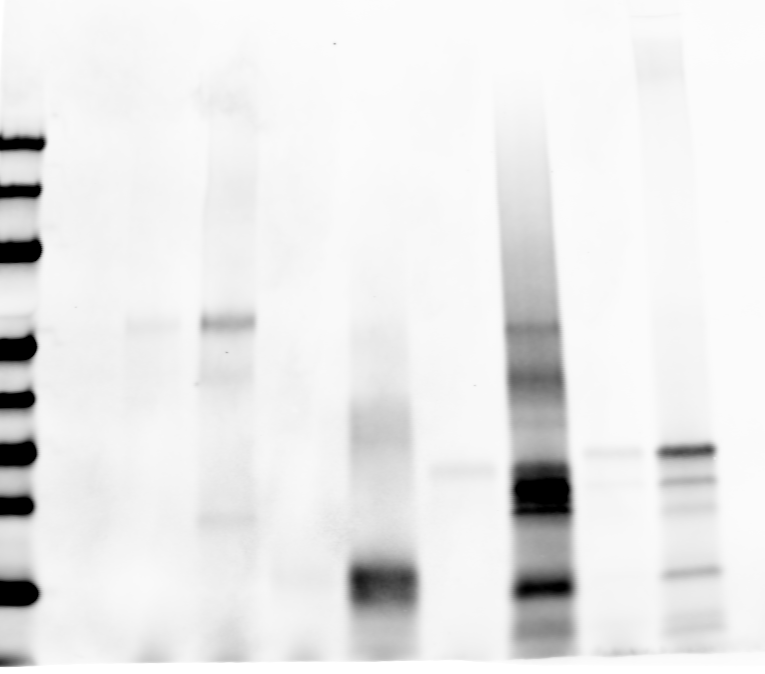

Supplement: Figure 2—figure supplement 1—source data 1. — The tiff files correspond to an uncropped picture of the AlexaFluor647 fluorescence signal, acquired on an Odyssey LI-COR, and of a scan of the membrane stained with Ponceau S. The jpg file combines the pictures used to generate and can be used to locate the protein ladders. [file elife-73913-fig2-figsupp1-data1.zip › Figure 2-figure supplement 1-source data 1/Fig.2-S1A-Click-AF647.tif]

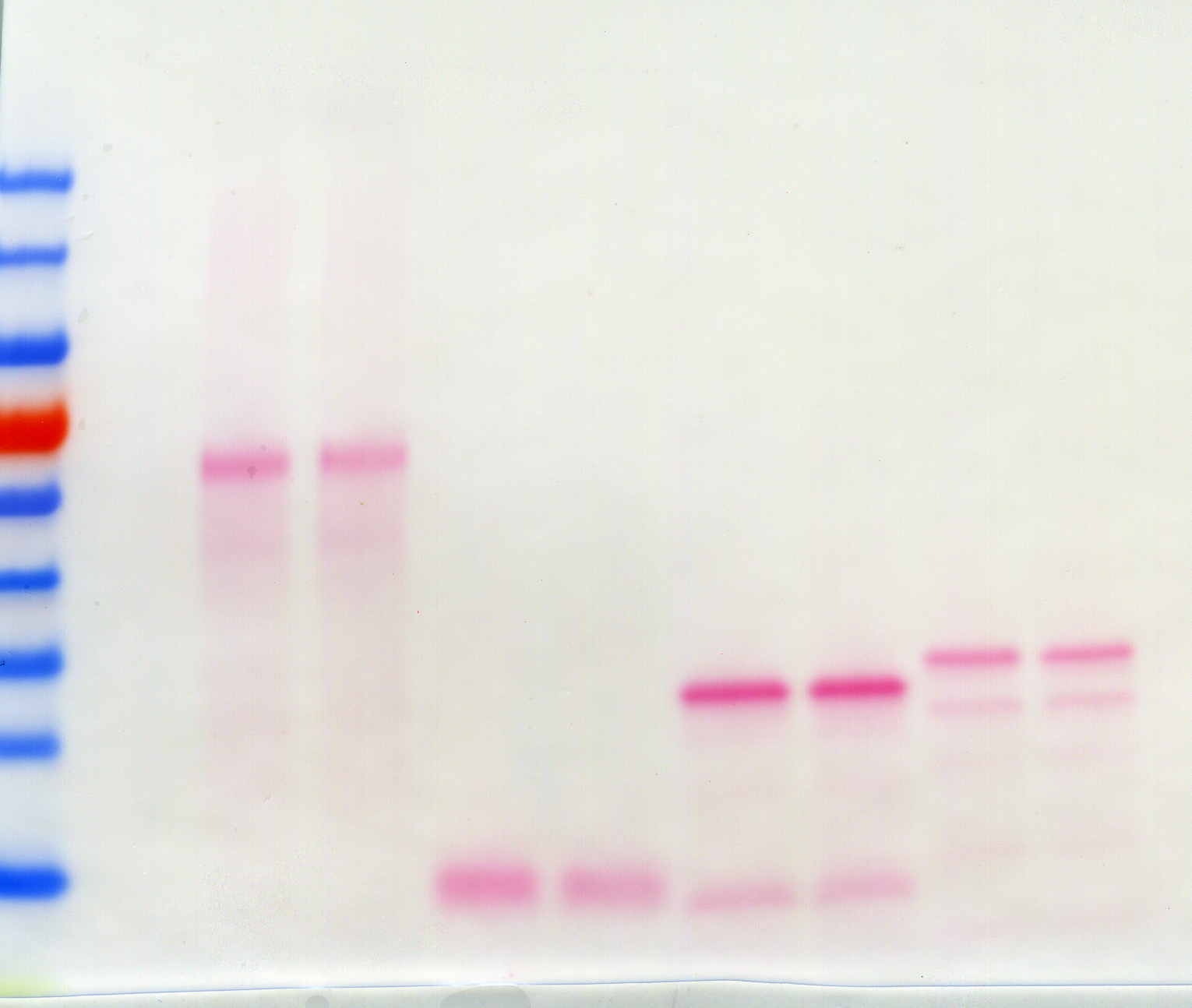

Supplement: Figure 2—figure supplement 1—source data 1. — The tiff files correspond to an uncropped picture of the AlexaFluor647 fluorescence signal, acquired on an Odyssey LI-COR, and of a scan of the membrane stained with Ponceau S. The jpg file combines the pictures used to generate and can be used to locate the protein ladders. [file elife-73913-fig2-figsupp1-data1.zip › Figure 2-figure supplement 1-source data 1/Fig.2-S1A-Ponceau.tif]

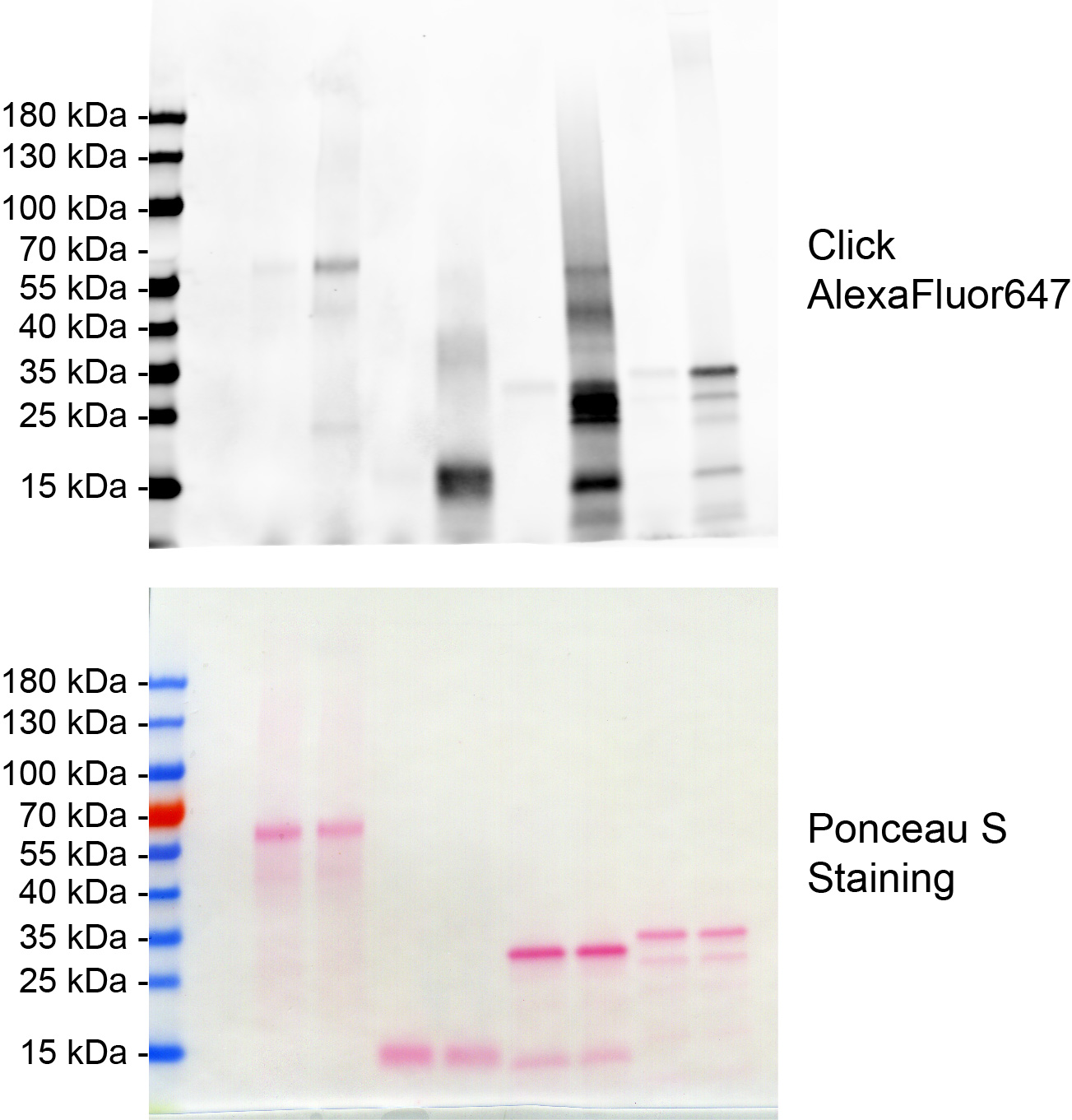

Supplement: Figure 2—figure supplement 1—source data 1. — The tiff files correspond to an uncropped picture of the AlexaFluor647 fluorescence signal, acquired on an Odyssey LI-COR, and of a scan of the membrane stained with Ponceau S. The jpg file combines the pictures used to generate and can be used to locate the protein ladders. [file elife-73913-fig2-figsupp1-data1.zip › Figure 2-figure supplement 1-source data 1/Fig.2-S1A.jpg]

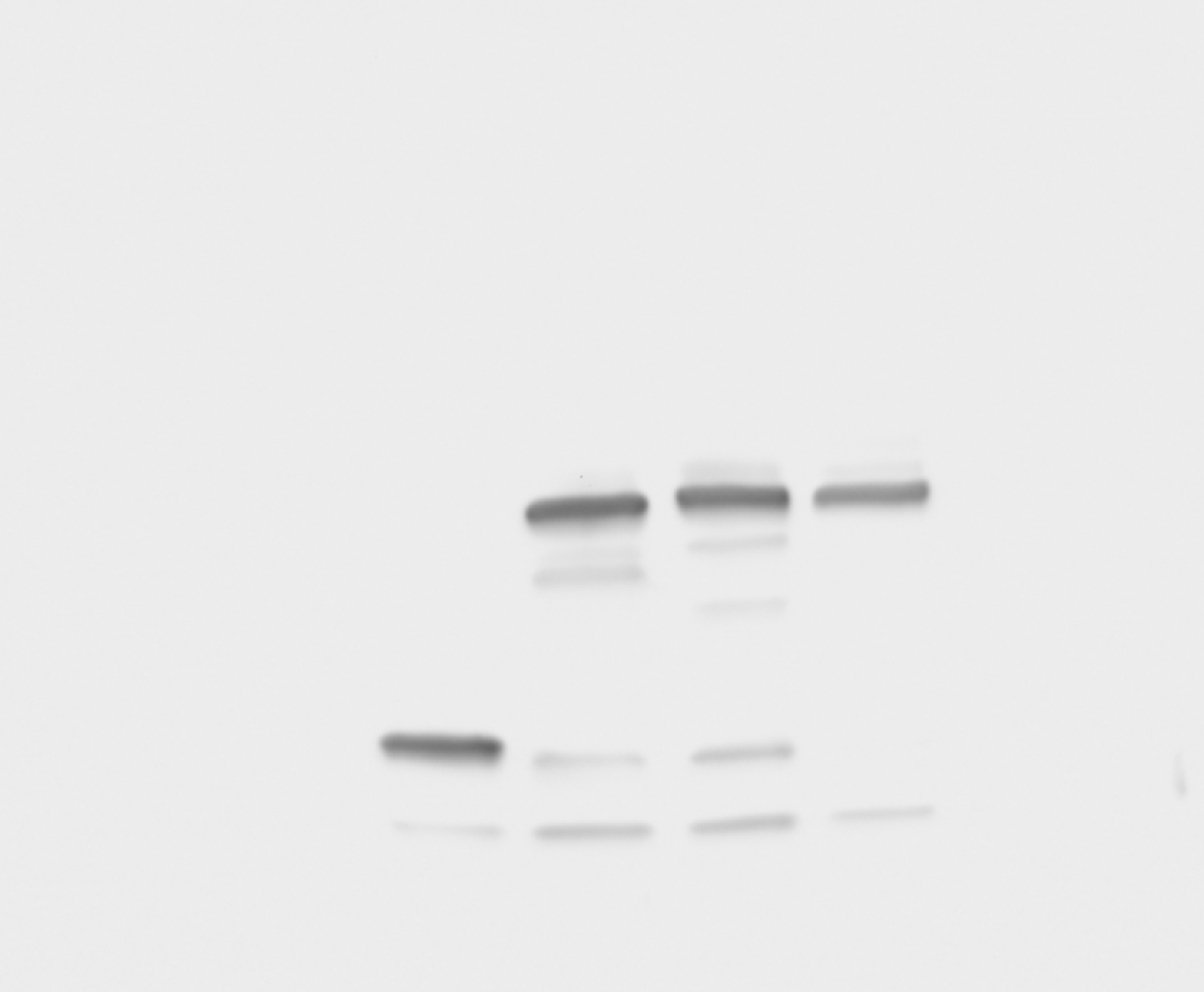

Supplement: Figure 2—figure supplement 2—source data 1. — The tiff files correspond to uncropped pictures the chemiluminescent signal acquired on a BioRad Chemidoc. The regions used to generate the figure are highlighted for each immunoblot by back squares in the jpg file, which also contains at the bottom an overlay with a picture of the membrane to locate the protein ladder positions. [file elife-73913-fig2-figsupp2-data1.zip › Figure 2-figure supplement 2-source data 1/Fig.2-S2G-GFP.tif]

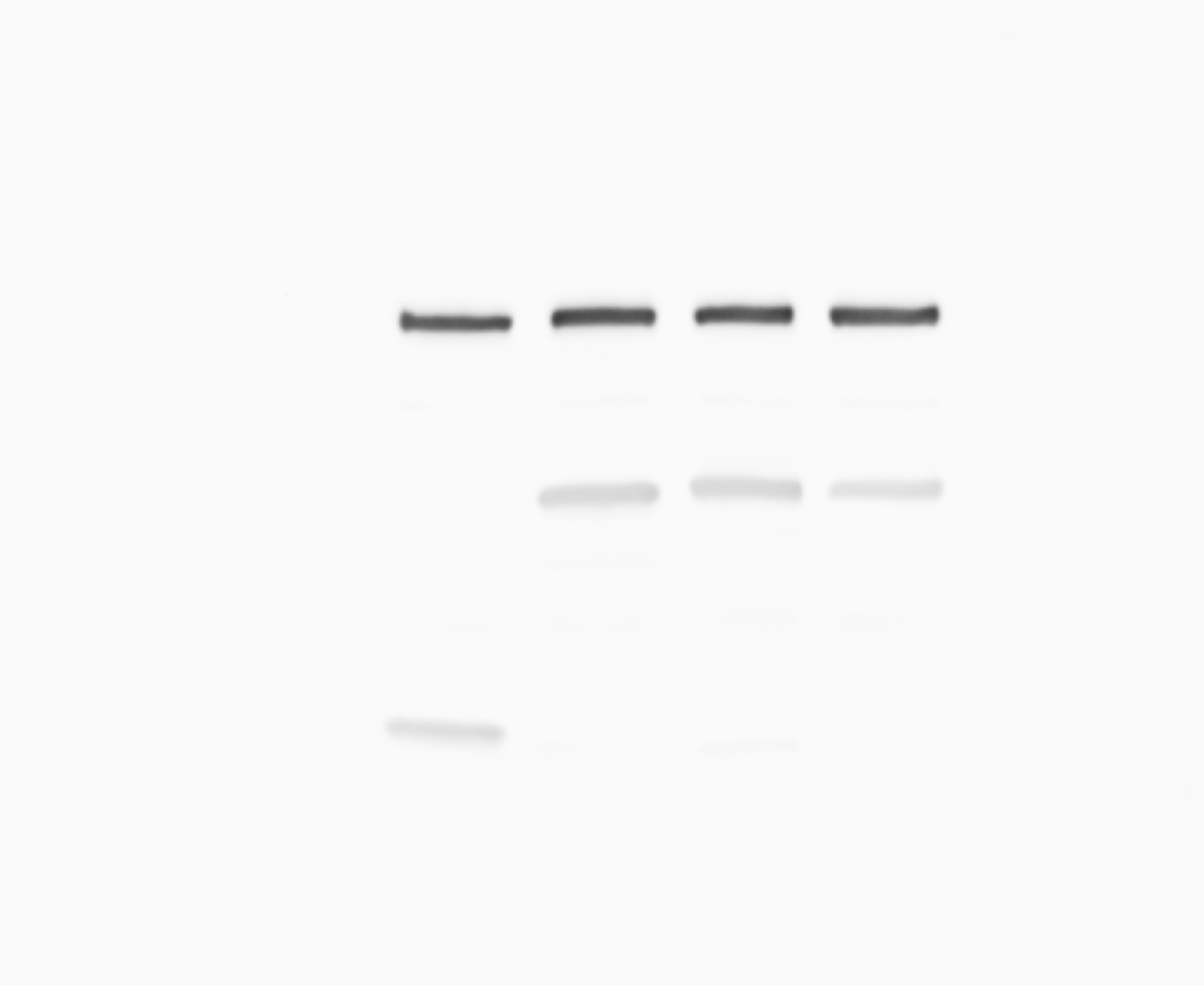

Supplement: Figure 2—figure supplement 2—source data 1. — The tiff files correspond to uncropped pictures the chemiluminescent signal acquired on a BioRad Chemidoc. The regions used to generate the figure are highlighted for each immunoblot by back squares in the jpg file, which also contains at the bottom an overlay with a picture of the membrane to locate the protein ladder positions. [file elife-73913-fig2-figsupp2-data1.zip › Figure 2-figure supplement 2-source data 1/Fig.2-S2G-SAF-A.tif]

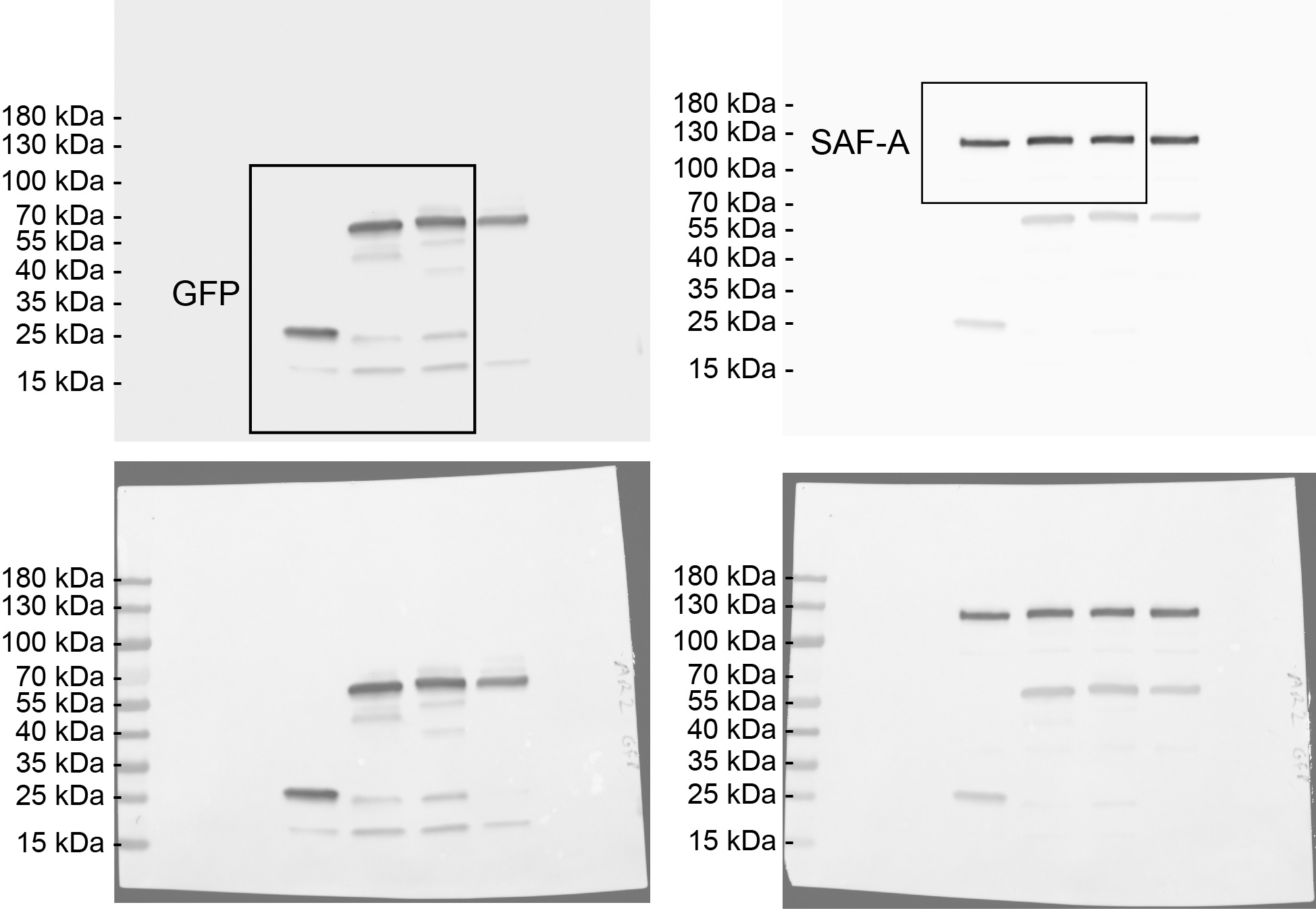

Supplement: Figure 2—figure supplement 2—source data 1. — The tiff files correspond to uncropped pictures the chemiluminescent signal acquired on a BioRad Chemidoc. The regions used to generate the figure are highlighted for each immunoblot by back squares in the jpg file, which also contains at the bottom an overlay with a picture of the membrane to locate the protein ladder positions. [file elife-73913-fig2-figsupp2-data1.zip › Figure 2-figure supplement 2-source data 1/Fig.2-S2G.jpg]

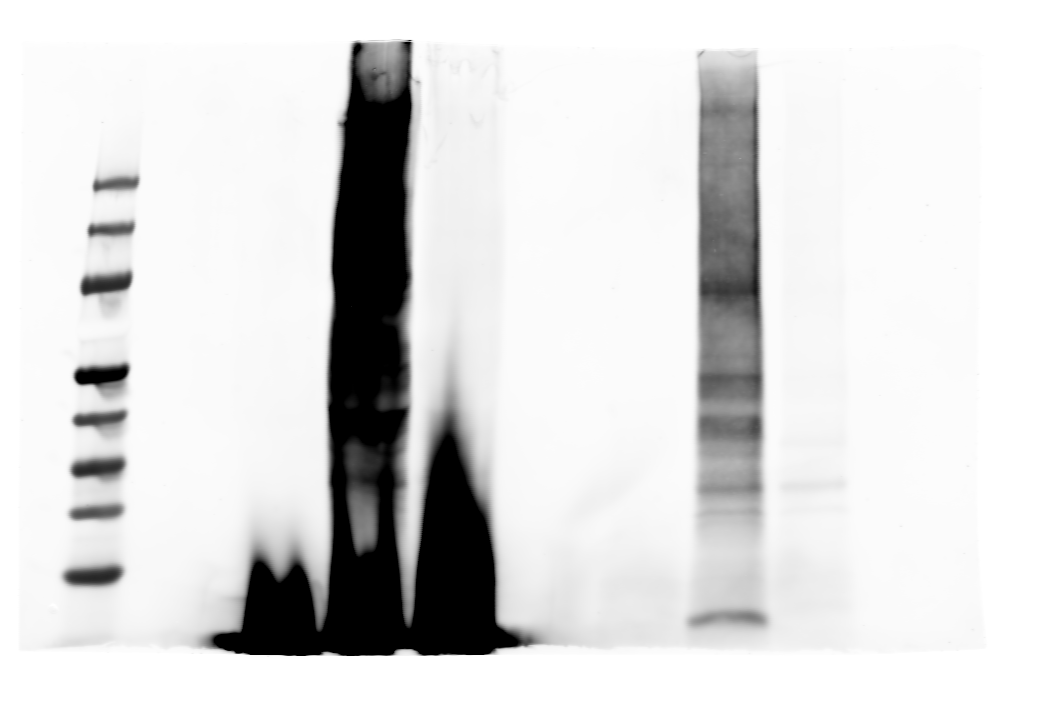

Supplement: Figure 3—source data 1. — The tiff files correspond to an uncropped picture of the AlexaFluor647 fluorescence signal, acquired on an Odyssey LI-COR, and of a scan of the membrane stained with Ponceau S. The regions used to generate the figure are highlighted by back squares in the jpg file. [file elife-73913-fig3-data1.zip › Figure 3-source data 1/Fig.3A-Click-AF647.tif]

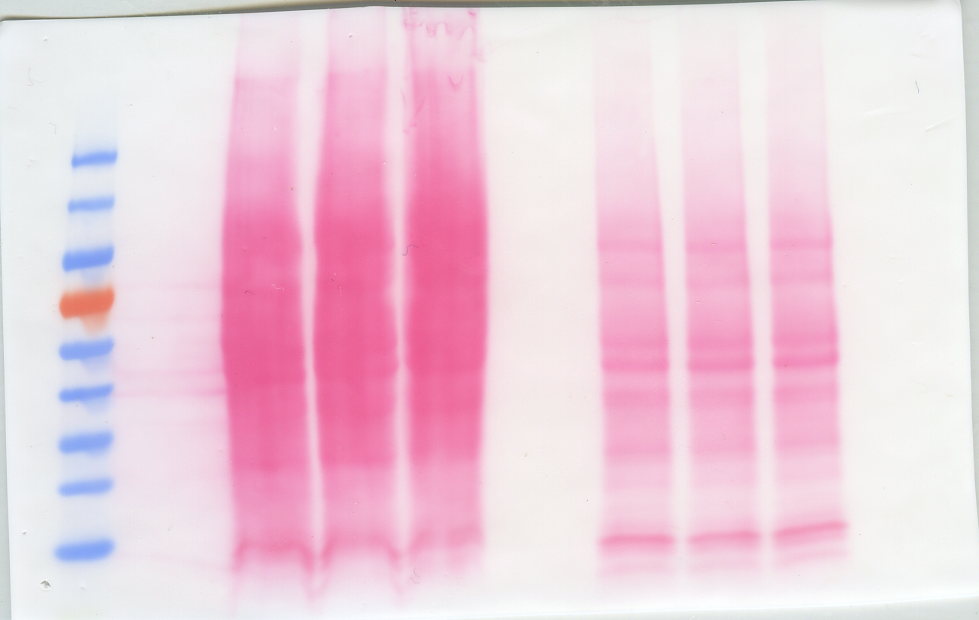

Supplement: Figure 3—source data 1. — The tiff files correspond to an uncropped picture of the AlexaFluor647 fluorescence signal, acquired on an Odyssey LI-COR, and of a scan of the membrane stained with Ponceau S. The regions used to generate the figure are highlighted by back squares in the jpg file. [file elife-73913-fig3-data1.zip › Figure 3-source data 1/Fig.3A-Ponceau.tif]

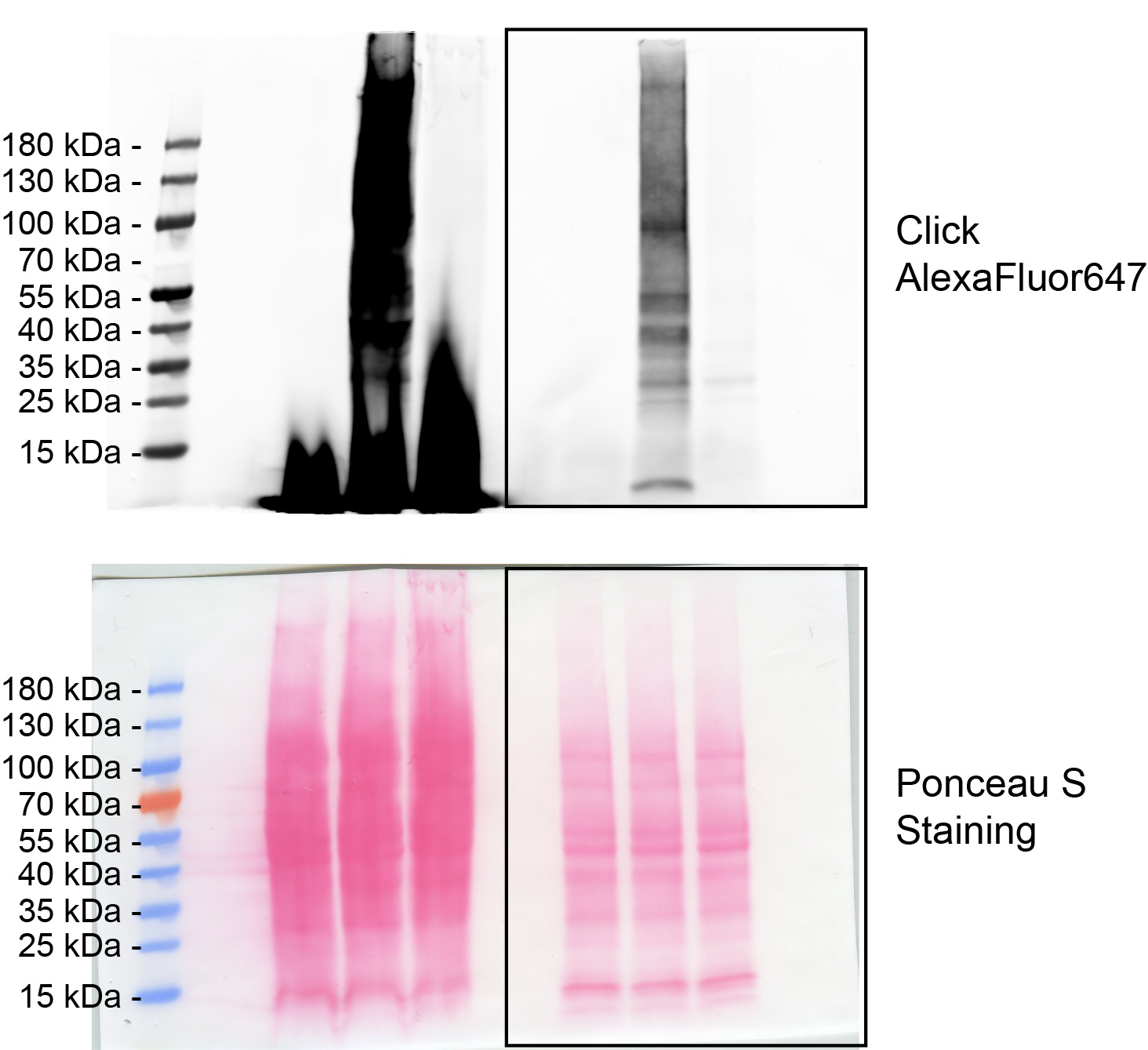

Supplement: Figure 3—source data 1. — The tiff files correspond to an uncropped picture of the AlexaFluor647 fluorescence signal, acquired on an Odyssey LI-COR, and of a scan of the membrane stained with Ponceau S. The regions used to generate the figure are highlighted by back squares in the jpg file. [file elife-73913-fig3-data1.zip › Figure 3-source data 1/Fig.3A.jpg]

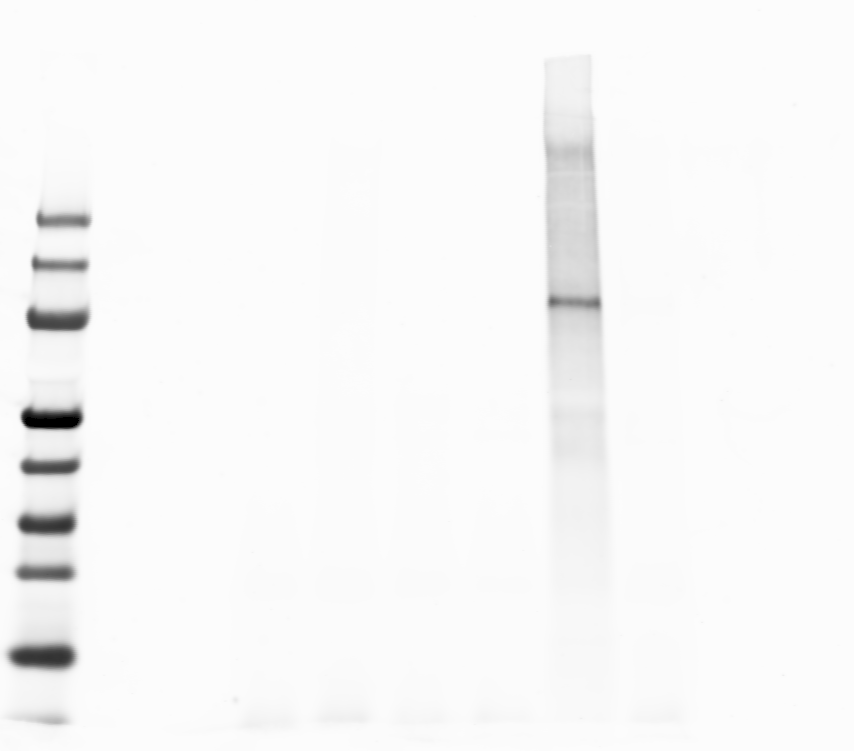

Supplement: Figure 3—source data 2. — The tiff files correspond to uncropped pictures of the AlexaFluor647 and PSMD2 fluorescence signal, both detected on a LI-COR Odyssey. The jpg file combines both pictures and can be used to locate the protein ladders. [file elife-73913-fig3-data2.zip › Figure 3-source data 2/Fig.3C-Click-AF647.tif]

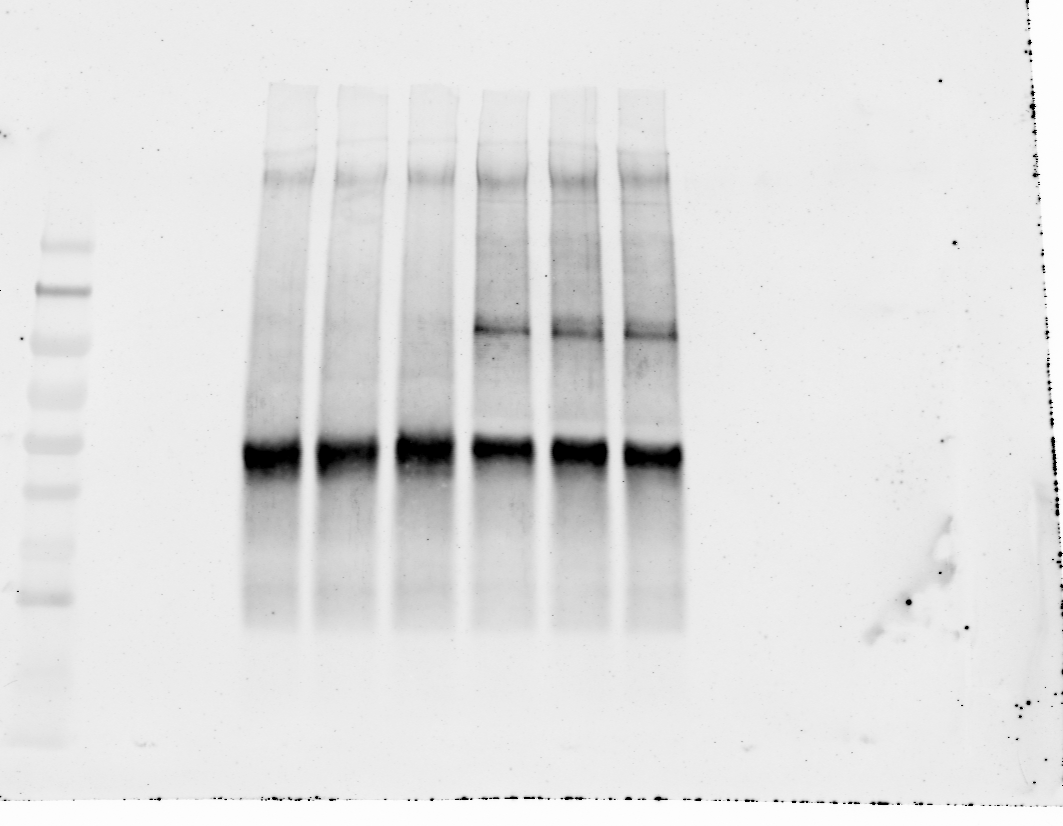

Supplement: Figure 3—source data 2. — The tiff files correspond to uncropped pictures of the AlexaFluor647 and PSMD2 fluorescence signal, both detected on a LI-COR Odyssey. The jpg file combines both pictures and can be used to locate the protein ladders. [file elife-73913-fig3-data2.zip › Figure 3-source data 2/Fig.3C-PSMD2.tif]

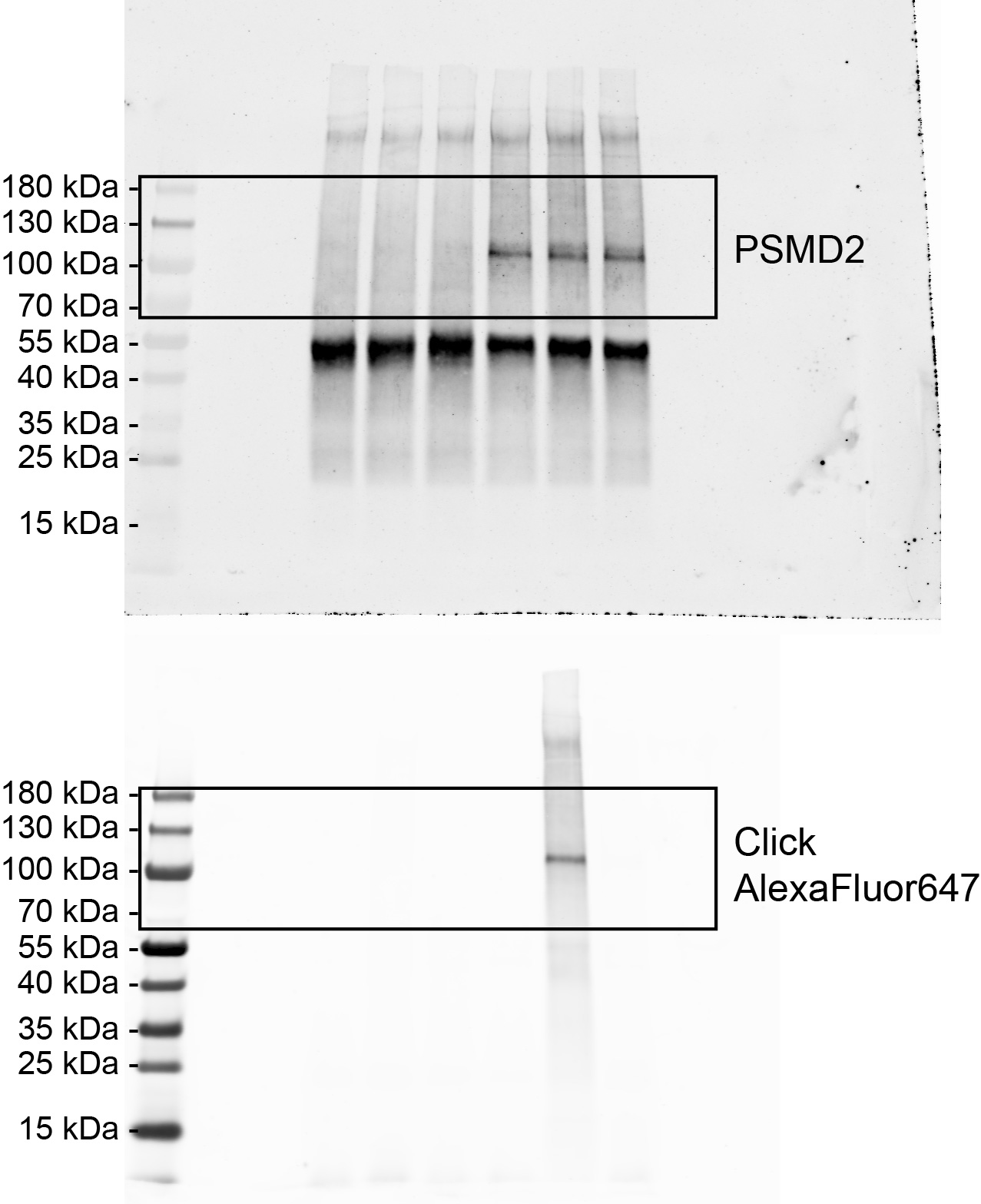

Supplement: Figure 3—source data 2. — The tiff files correspond to uncropped pictures of the AlexaFluor647 and PSMD2 fluorescence signal, both detected on a LI-COR Odyssey. The jpg file combines both pictures and can be used to locate the protein ladders. [file elife-73913-fig3-data2.zip › Figure 3-source data 2/Fig.3C.jpg]

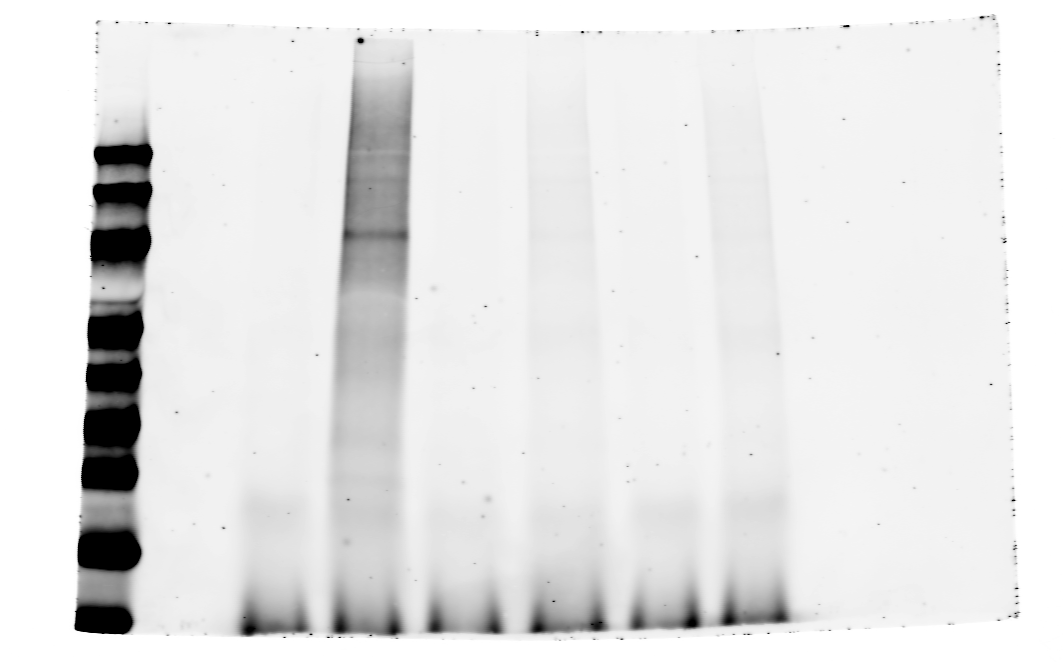

Supplement: Figure 3—source data 3. — The tiff files correspond to uncropped pictures of the AlexaFluor647 and PSMD2 fluorescence signal, both detected on a LI-COR Odyssey. The jpg file combines both pictures and can be used to locate the protein ladders. [file elife-73913-fig3-data3.zip › Figure 3-source data 3/Fig.3D-Click-AF647.tif]

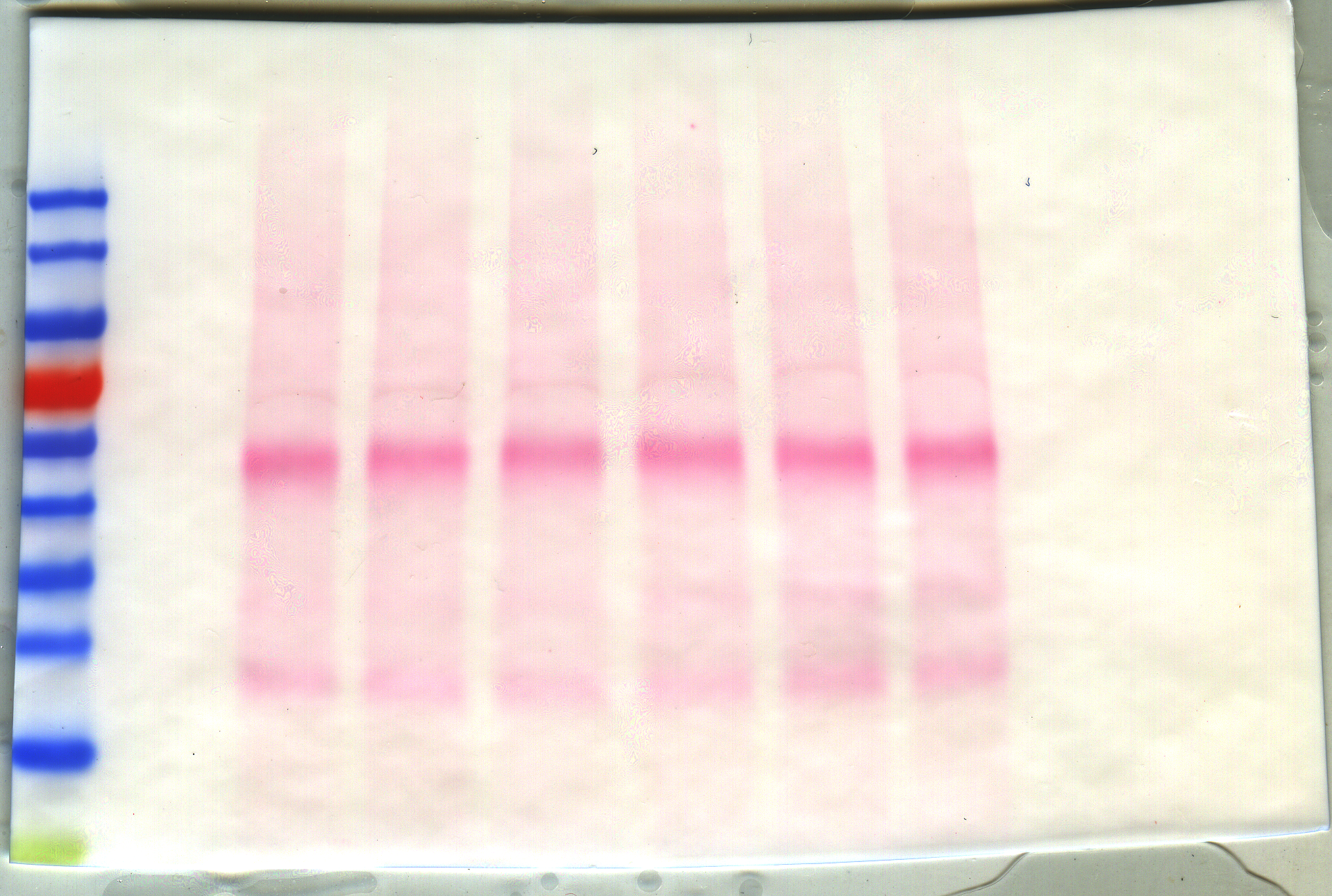

Supplement: Figure 3—source data 3. — The tiff files correspond to uncropped pictures of the AlexaFluor647 and PSMD2 fluorescence signal, both detected on a LI-COR Odyssey. The jpg file combines both pictures and can be used to locate the protein ladders. [file elife-73913-fig3-data3.zip › Figure 3-source data 3/Fig.3D-Ponceau.tif]

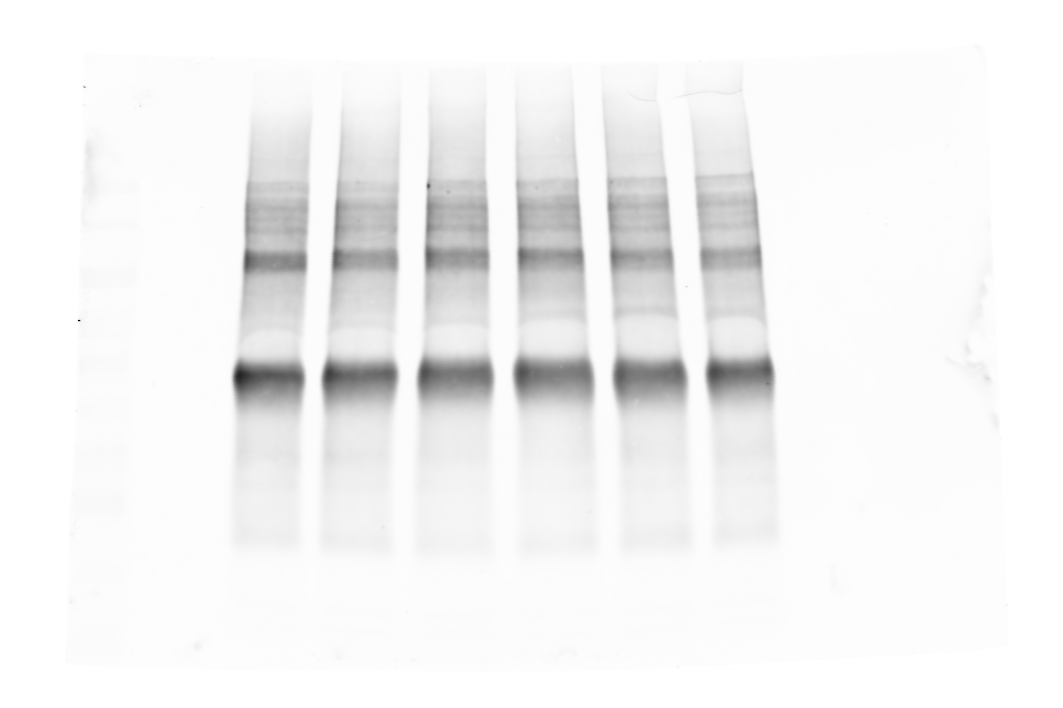

Supplement: Figure 3—source data 3. — The tiff files correspond to uncropped pictures of the AlexaFluor647 and PSMD2 fluorescence signal, both detected on a LI-COR Odyssey. The jpg file combines both pictures and can be used to locate the protein ladders. [file elife-73913-fig3-data3.zip › Figure 3-source data 3/Fig.3D-PSMD2.tif]

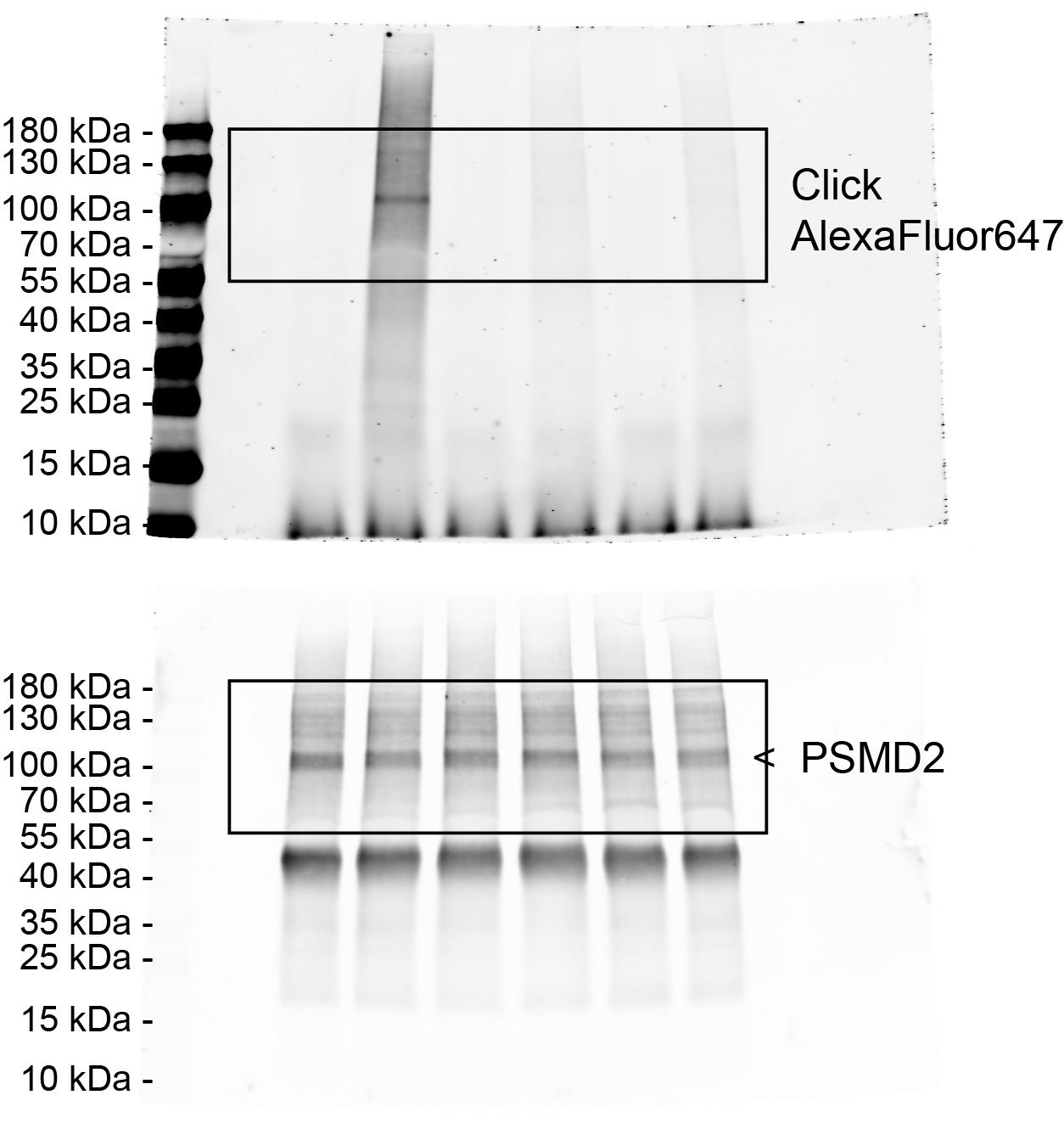

Supplement: Figure 3—source data 3. — The tiff files correspond to uncropped pictures of the AlexaFluor647 and PSMD2 fluorescence signal, both detected on a LI-COR Odyssey. The jpg file combines both pictures and can be used to locate the protein ladders. [file elife-73913-fig3-data3.zip › Figure 3-source data 3/Fig.3D.jpg]

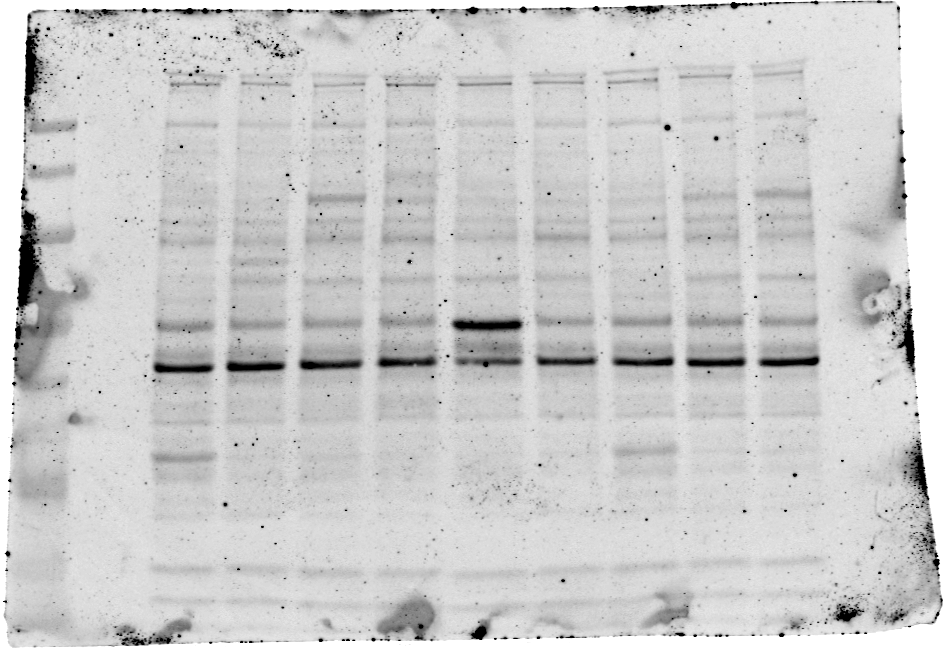

Supplement: Figure 3—figure supplement 2—source data 1. — The tiff files correspond to uncropped pictures of the IRDye800CW fluorescence signal acquired on a LI-COR Odyssey. The regions used to generate the figure are highlighted by back squares in the jpg file. [file elife-73913-fig3-figsupp2-data1.zip › Figure 3-figure supplement 2-source data 1/Fig.3-S2B-beta-actin.tif]

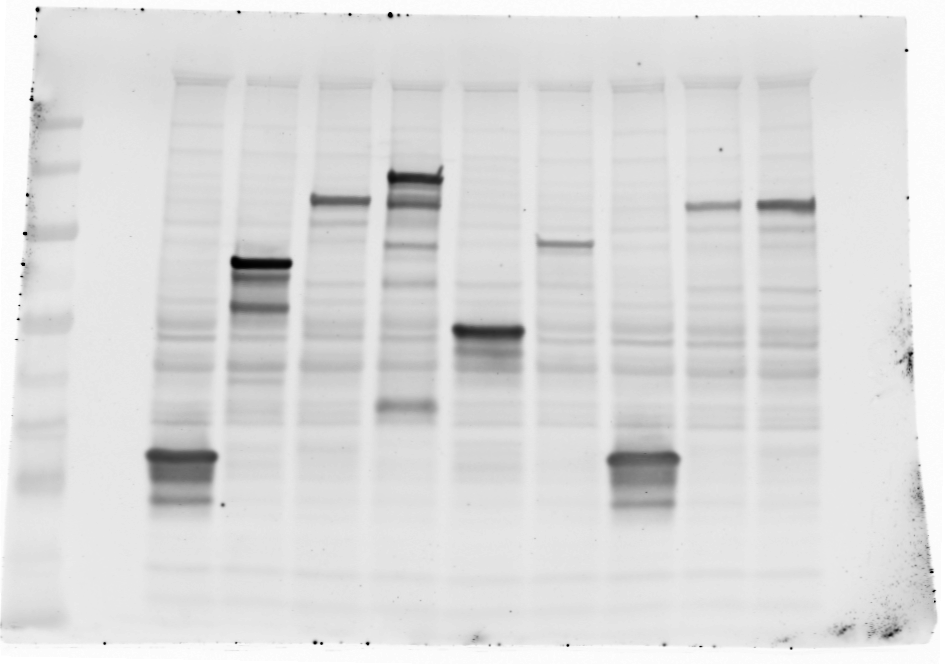

Supplement: Figure 3—figure supplement 2—source data 1. — The tiff files correspond to uncropped pictures of the IRDye800CW fluorescence signal acquired on a LI-COR Odyssey. The regions used to generate the figure are highlighted by back squares in the jpg file. [file elife-73913-fig3-figsupp2-data1.zip › Figure 3-figure supplement 2-source data 1/Fig.3-S2B-GFP.tif]

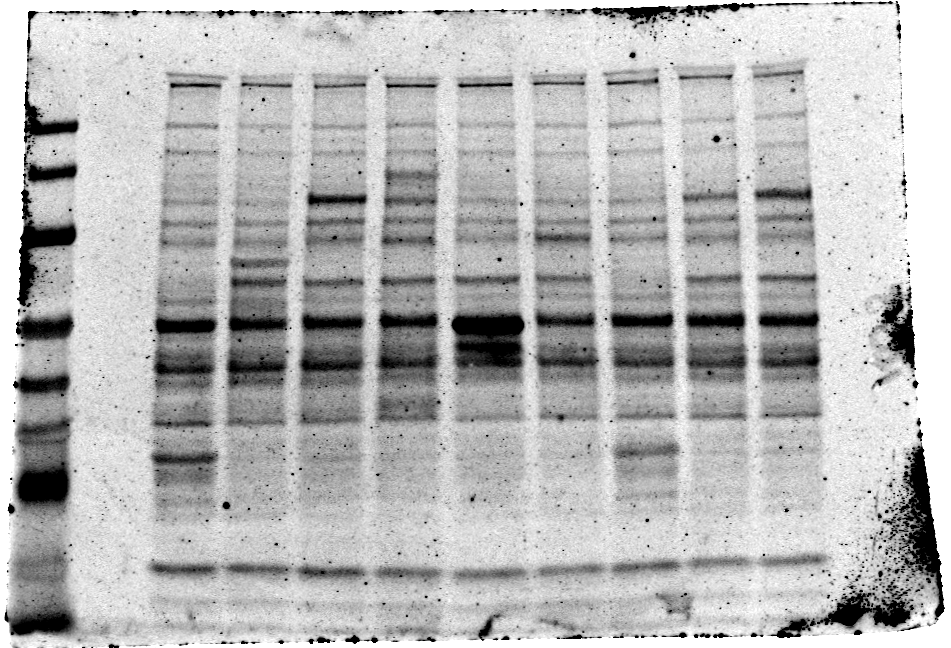

Supplement: Figure 3—figure supplement 2—source data 1. — The tiff files correspond to uncropped pictures of the IRDye800CW fluorescence signal acquired on a LI-COR Odyssey. The regions used to generate the figure are highlighted by back squares in the jpg file. [file elife-73913-fig3-figsupp2-data1.zip › Figure 3-figure supplement 2-source data 1/Fig.3-S2B-H2AX.tif]

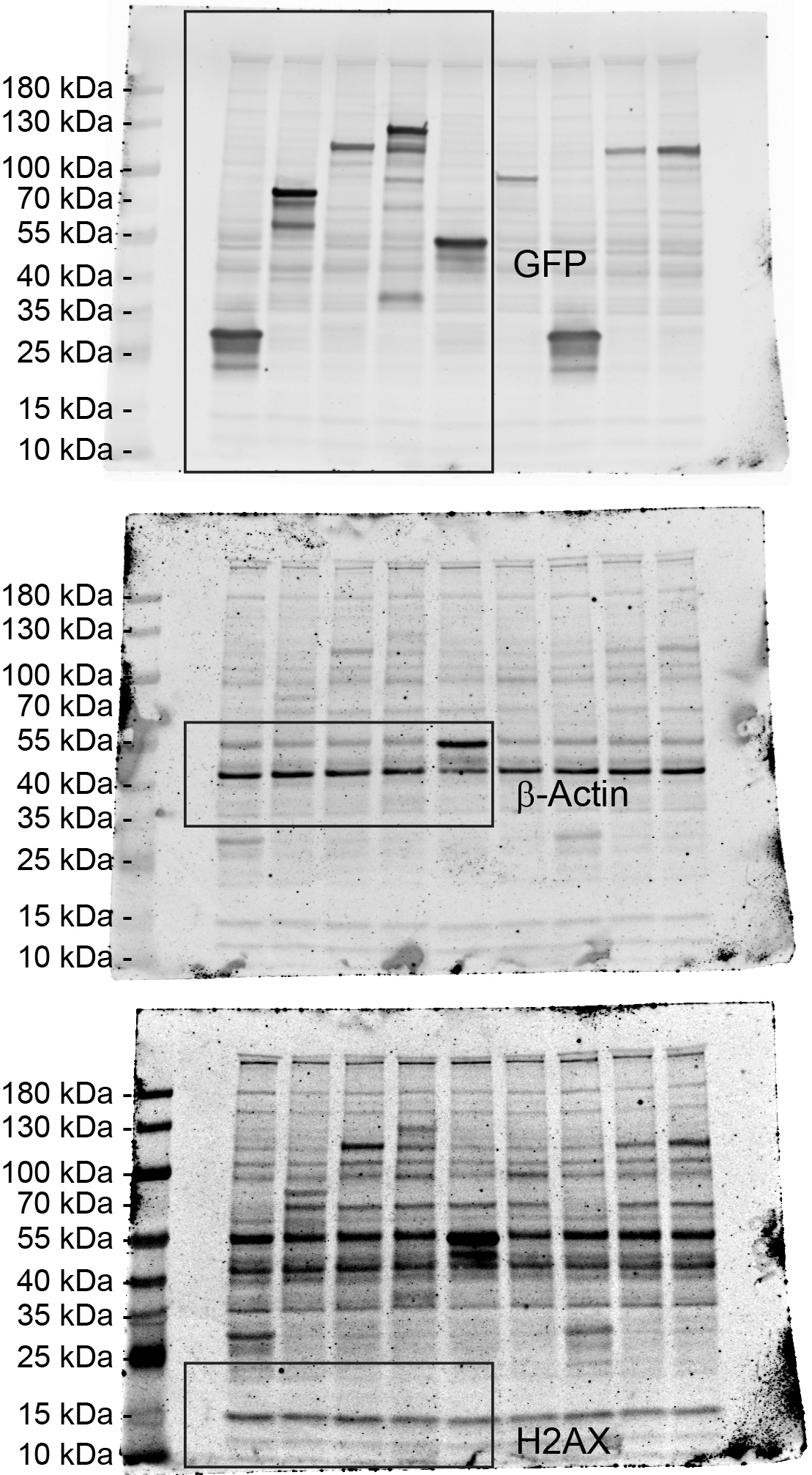

Supplement: Figure 3—figure supplement 2—source data 1. — The tiff files correspond to uncropped pictures of the IRDye800CW fluorescence signal acquired on a LI-COR Odyssey. The regions used to generate the figure are highlighted by back squares in the jpg file. [file elife-73913-fig3-figsupp2-data1.zip › Figure 3-figure supplement 2-source data 1/Fig.3-S2B.jpg]

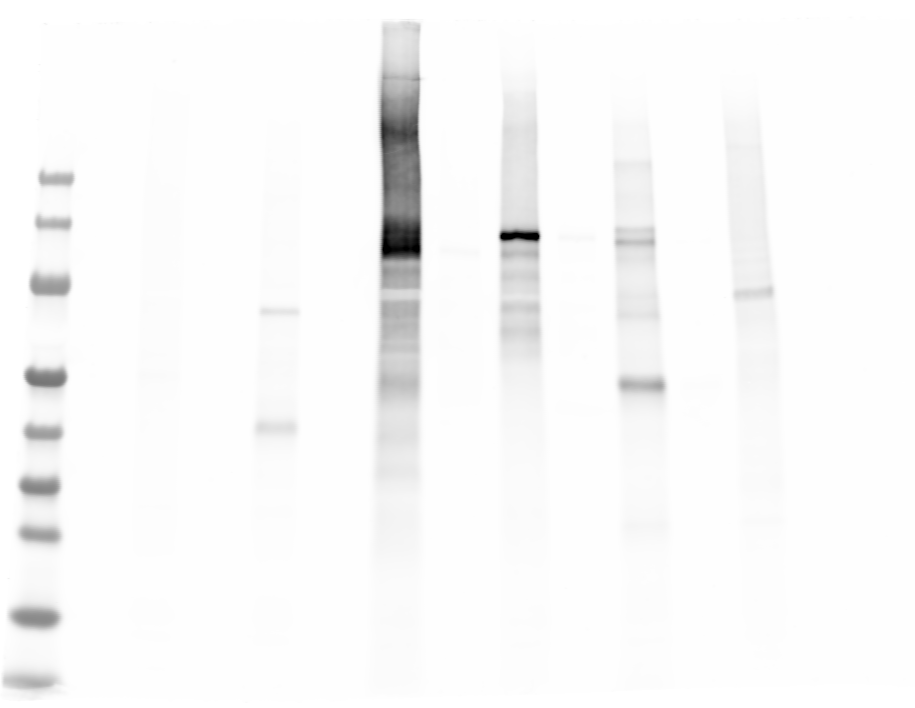

Supplement: Figure 3—figure supplement 2—source data 2. — The tiff files correspond to uncropped pictures of the AlexaFluor647 fluorescence signal and of the IRDye800CW signal (GFP), both acquired on an Odyssey LI-COR, and a scan of the membrane stained with Ponceau S. The jpg file combines all pictures and can be used to locate the protein ladders. [file elife-73913-fig3-figsupp2-data2.zip › Figure 3-figure supplement 2-source data 2/Fig.3-S2C-Click-AF647.tif]

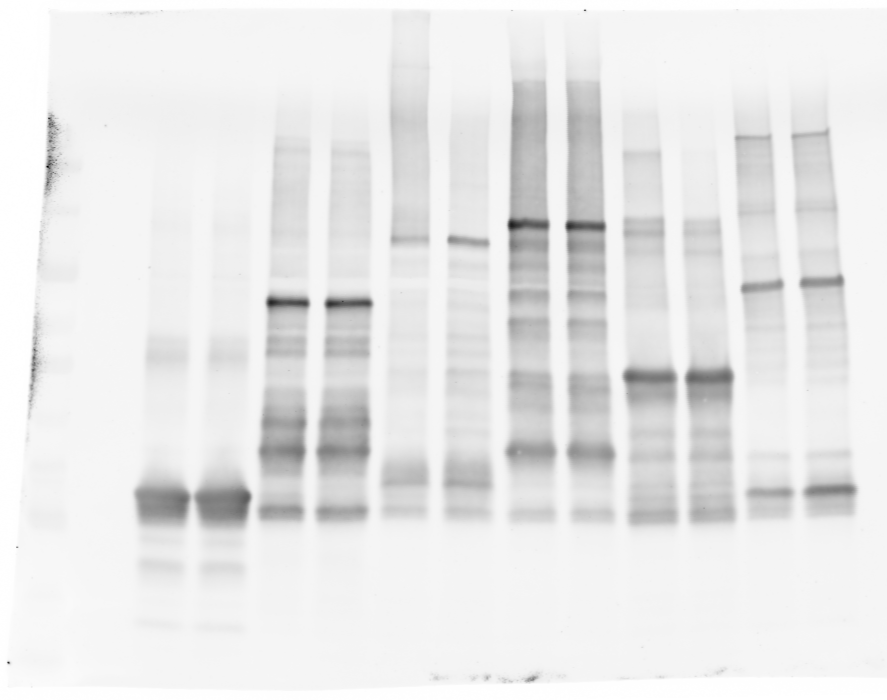

Supplement: Figure 3—figure supplement 2—source data 2. — The tiff files correspond to uncropped pictures of the AlexaFluor647 fluorescence signal and of the IRDye800CW signal (GFP), both acquired on an Odyssey LI-COR, and a scan of the membrane stained with Ponceau S. The jpg file combines all pictures and can be used to locate the protein ladders. [file elife-73913-fig3-figsupp2-data2.zip › Figure 3-figure supplement 2-source data 2/Fig.3-S2C-GFP.tif]

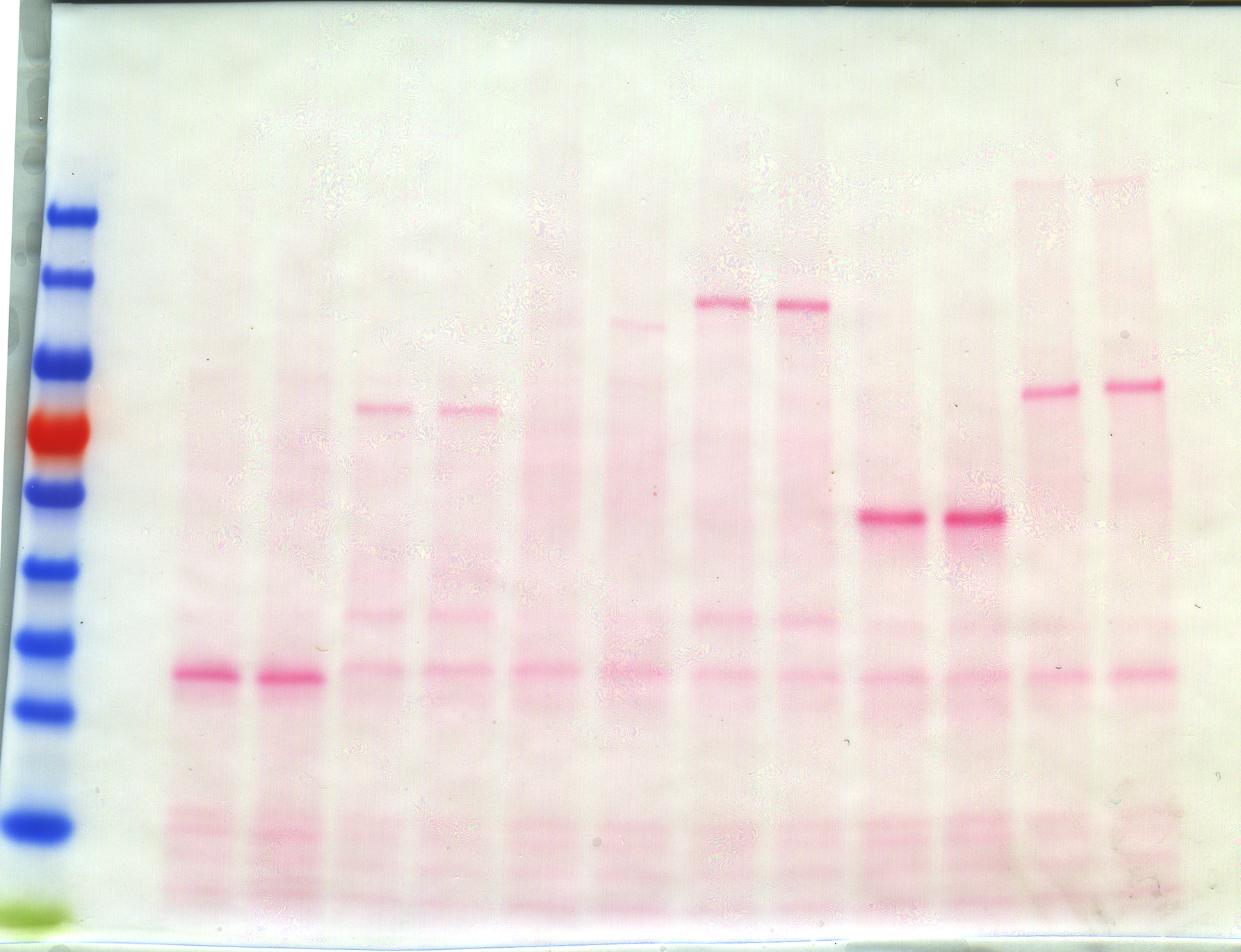

Supplement: Figure 3—figure supplement 2—source data 2. — The tiff files correspond to uncropped pictures of the AlexaFluor647 fluorescence signal and of the IRDye800CW signal (GFP), both acquired on an Odyssey LI-COR, and a scan of the membrane stained with Ponceau S. The jpg file combines all pictures and can be used to locate the protein ladders. [file elife-73913-fig3-figsupp2-data2.zip › Figure 3-figure supplement 2-source data 2/Fig.3-S2C-Ponceau.tif]

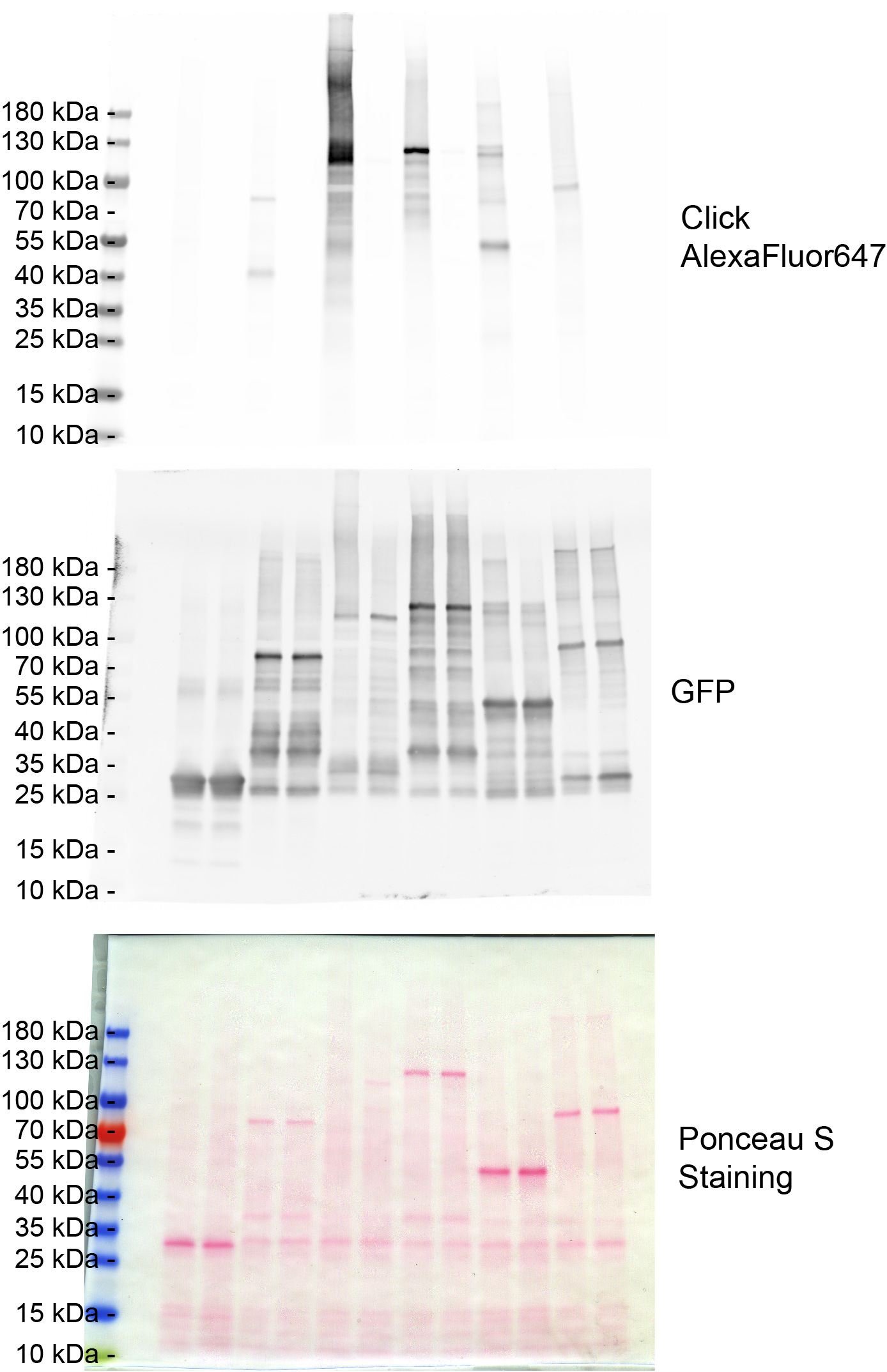

Supplement: Figure 3—figure supplement 2—source data 2. — The tiff files correspond to uncropped pictures of the AlexaFluor647 fluorescence signal and of the IRDye800CW signal (GFP), both acquired on an Odyssey LI-COR, and a scan of the membrane stained with Ponceau S. The jpg file combines all pictures and can be used to locate the protein ladders. [file elife-73913-fig3-figsupp2-data2.zip › Figure 3-figure supplement 2-source data 2/Fig.3-S2C.jpg]

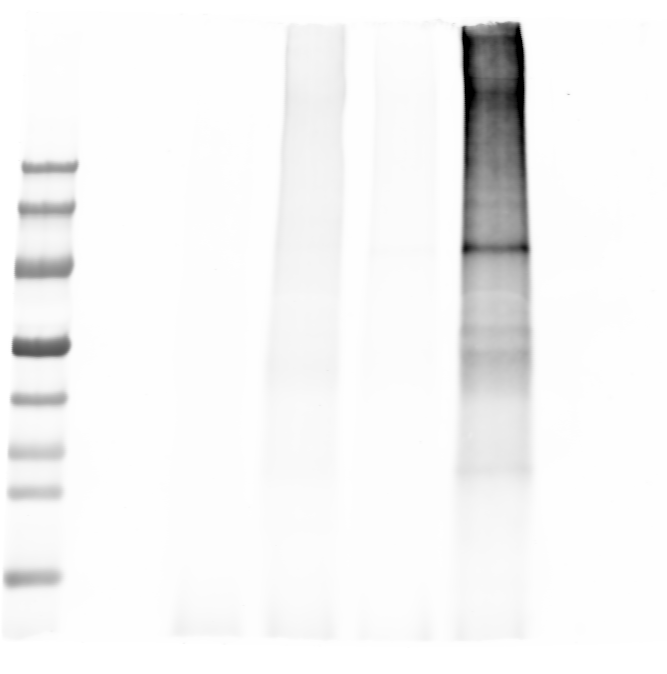

Supplement: Figure 3—figure supplement 2—source data 3. — The tiff files correspond to uncropped pictures of the AlexaFluor647 and PSMD2 fluorescence signal, both detected on a LI-COR Odyssey. The jpg file combines both pictures and can be used to locate the protein ladders. [file elife-73913-fig3-figsupp2-data3.zip › Figure 3-figure supplement 2-source data 3/Fig.3-S2D-Click-AF647.tif]

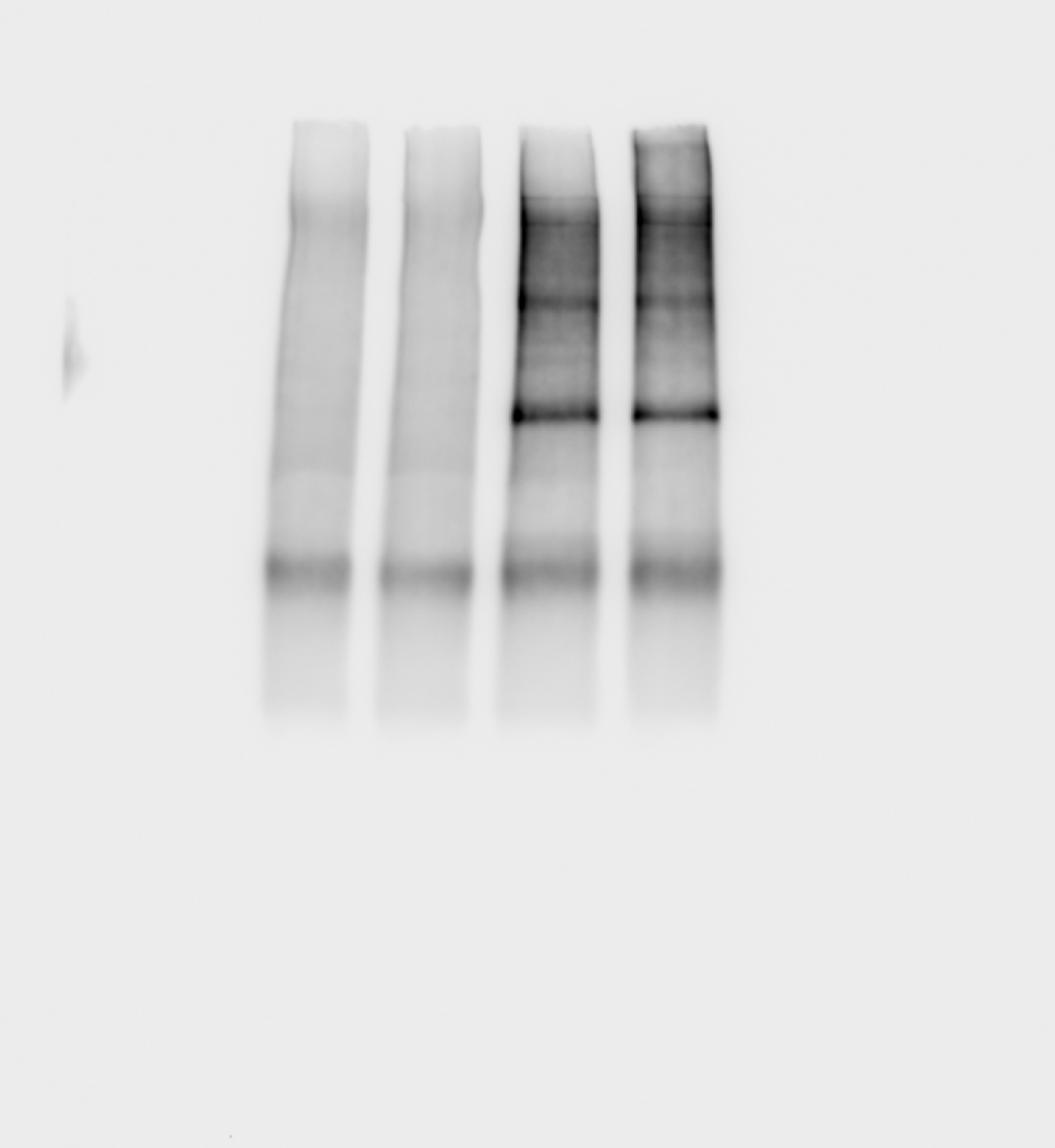

Supplement: Figure 3—figure supplement 2—source data 3. — The tiff files correspond to uncropped pictures of the AlexaFluor647 and PSMD2 fluorescence signal, both detected on a LI-COR Odyssey. The jpg file combines both pictures and can be used to locate the protein ladders. [file elife-73913-fig3-figsupp2-data3.zip › Figure 3-figure supplement 2-source data 3/Fig.3-S2D-PSMD.tif]

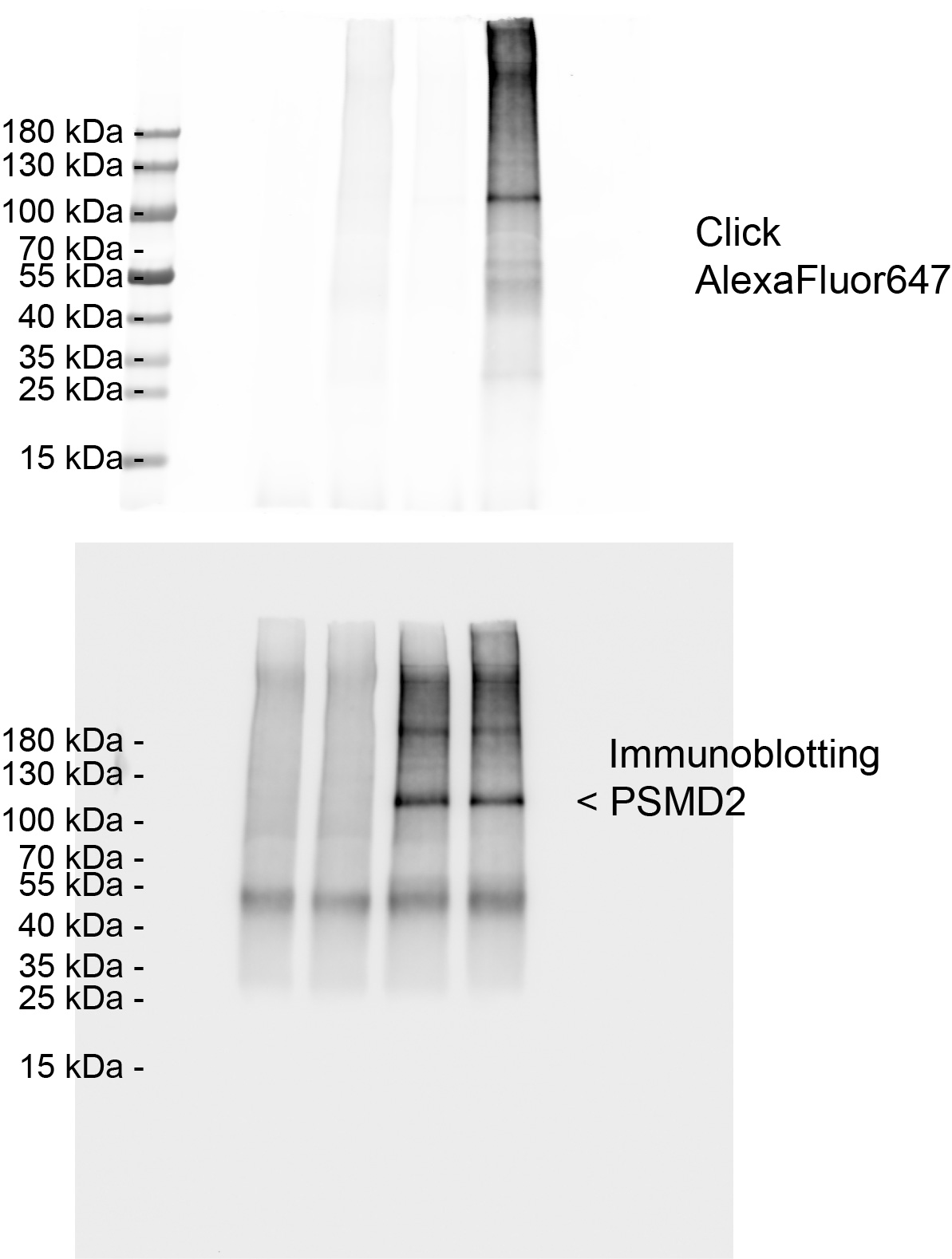

Supplement: Figure 3—figure supplement 2—source data 3. — The tiff files correspond to uncropped pictures of the AlexaFluor647 and PSMD2 fluorescence signal, both detected on a LI-COR Odyssey. The jpg file combines both pictures and can be used to locate the protein ladders. [file elife-73913-fig3-figsupp2-data3.zip › Figure 3-figure supplement 2-source data 3/Fig.3-S2D.jpg]

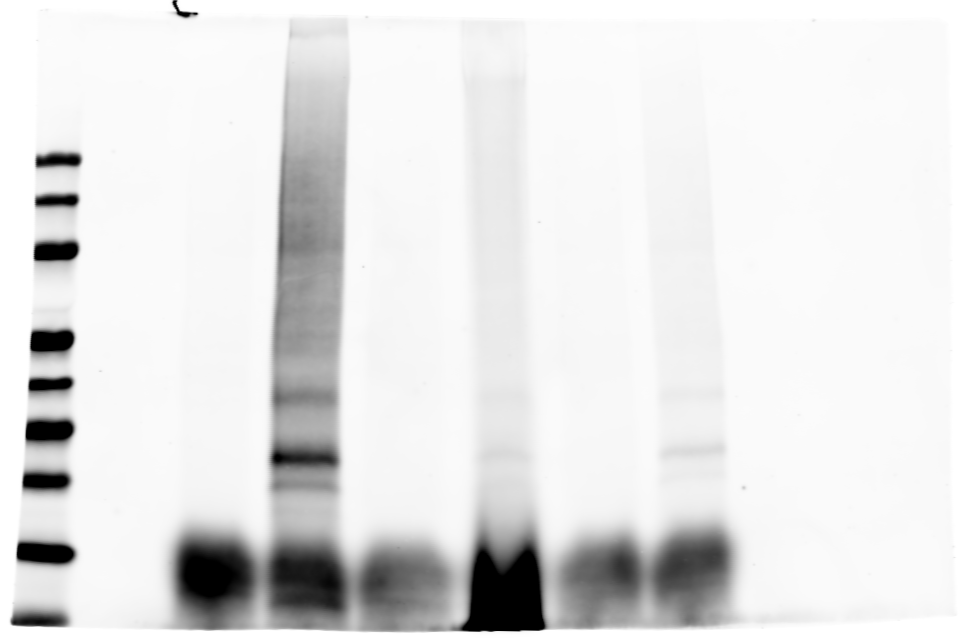

Supplement: Figure 3—figure supplement 2—source data 4. — The tiff files correspond to an uncropped picture of the AlexaFluor647 fluorescence signal, acquired on an Odyssey LI-COR, and of a scan of the membrane stained with Ponceau S. The jpg file combines both pictures and can be used to locate the protein ladders. [file elife-73913-fig3-figsupp2-data4.zip › Figure 3-figure supplement 2-source data 4/Fig.3-S2E-Click-AF647.tif]

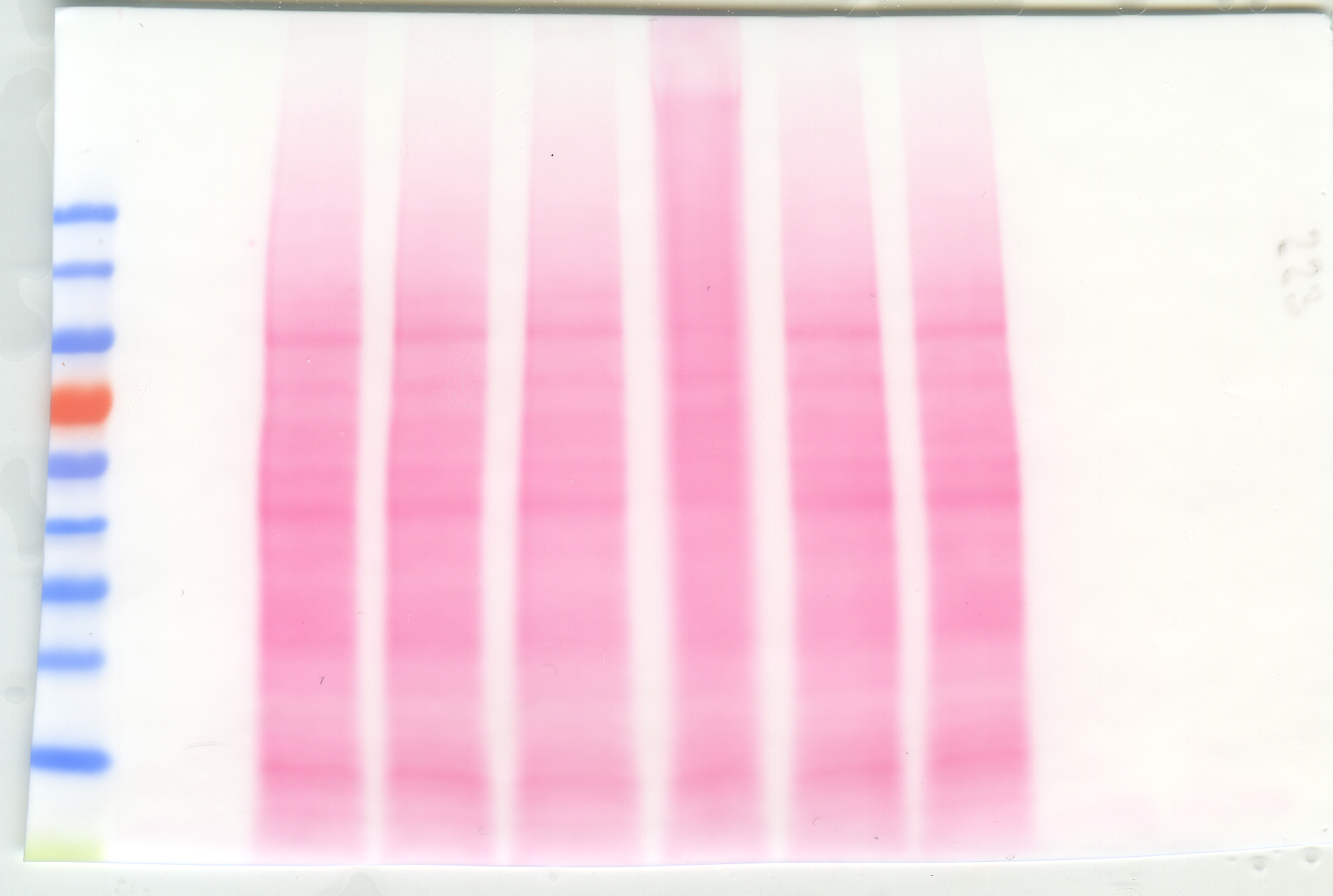

Supplement: Figure 3—figure supplement 2—source data 4. — The tiff files correspond to an uncropped picture of the AlexaFluor647 fluorescence signal, acquired on an Odyssey LI-COR, and of a scan of the membrane stained with Ponceau S. The jpg file combines both pictures and can be used to locate the protein ladders. [file elife-73913-fig3-figsupp2-data4.zip › Figure 3-figure supplement 2-source data 4/Fig.3-S2E-Ponceau.tif]

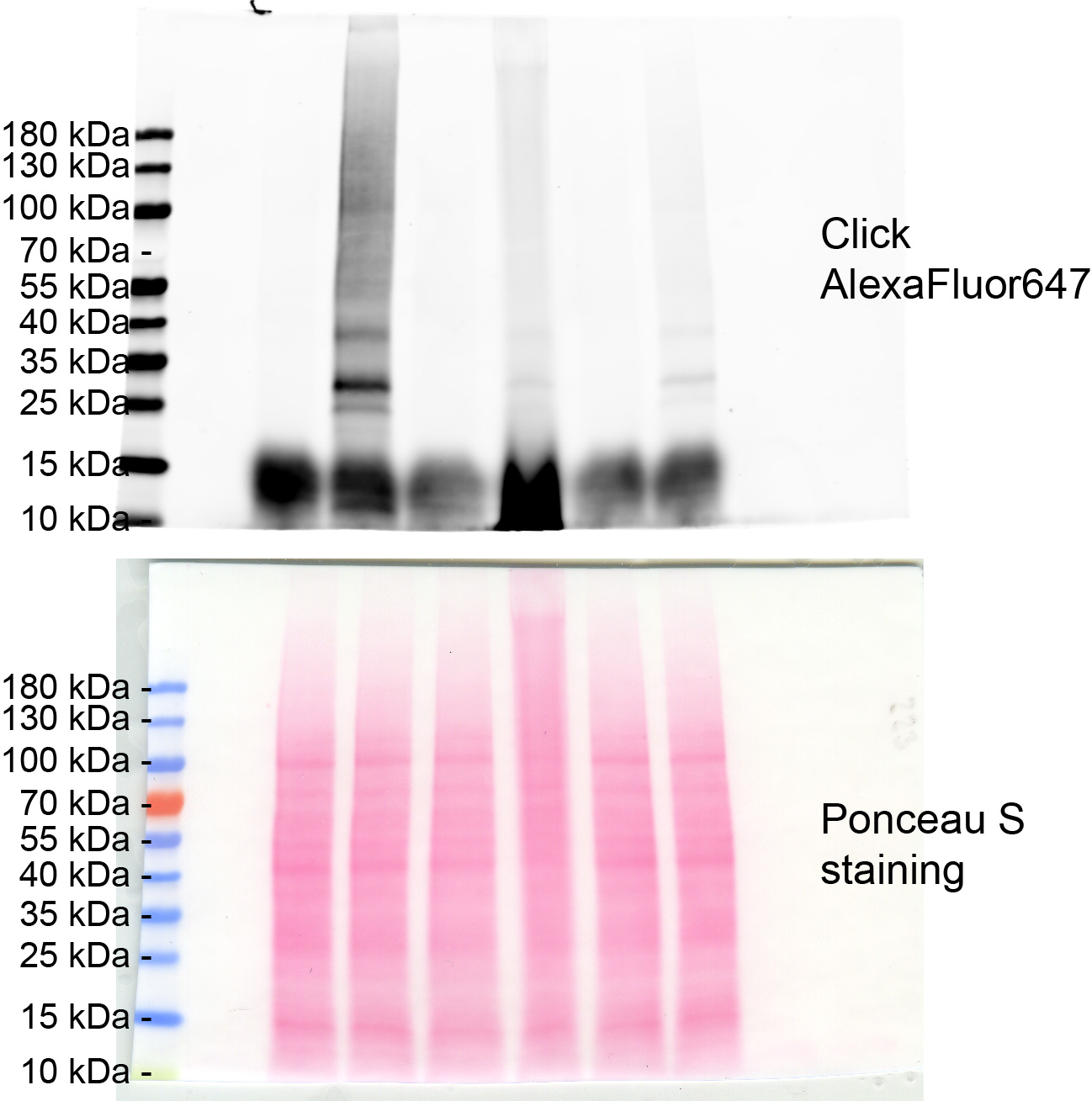

Supplement: Figure 3—figure supplement 2—source data 4. — The tiff files correspond to an uncropped picture of the AlexaFluor647 fluorescence signal, acquired on an Odyssey LI-COR, and of a scan of the membrane stained with Ponceau S. The jpg file combines both pictures and can be used to locate the protein ladders. [file elife-73913-fig3-figsupp2-data4.zip › Figure 3-figure supplement 2-source data 4/Fig.3-S2E.jpg]

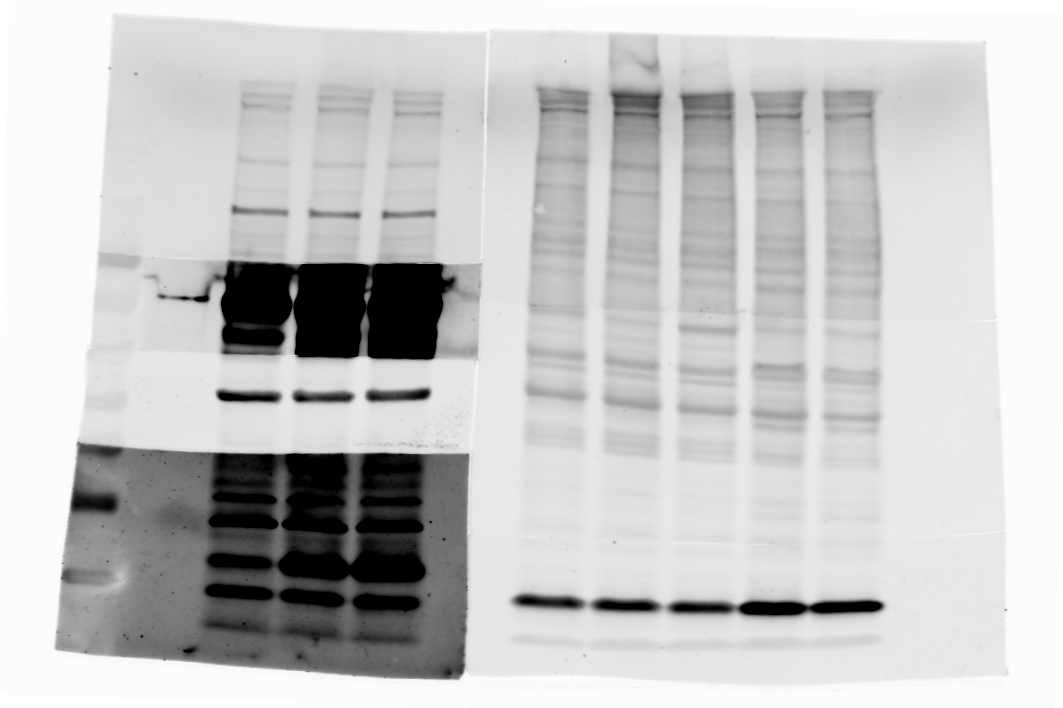

Supplement: Figure 4—source data 1. — The tiff files correspond to uncropped pictures of the IRDye800CW fluorescence signal acquired on a LI-COR Odyssey. The regions used to generate the figure are highlighted by back squares in the jpg file. [file elife-73913-fig4-data1.zip › Figure 4-source data 1/Fig.4C-beta-Actin.tif]

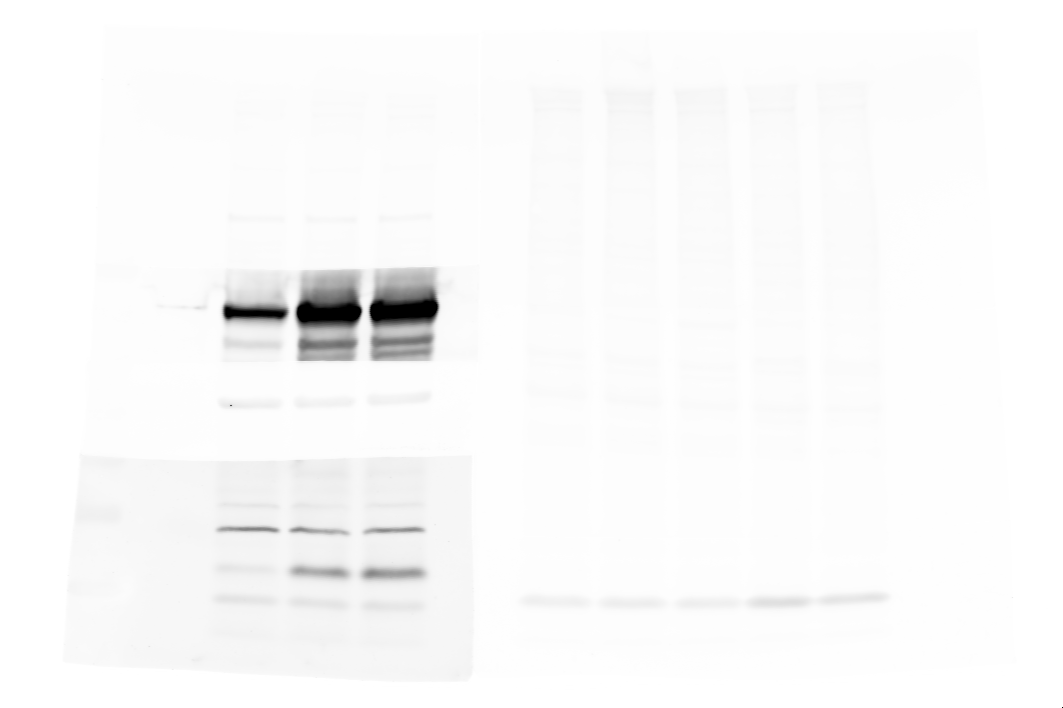

Supplement: Figure 4—source data 1. — The tiff files correspond to uncropped pictures of the IRDye800CW fluorescence signal acquired on a LI-COR Odyssey. The regions used to generate the figure are highlighted by back squares in the jpg file. [file elife-73913-fig4-data1.zip › Figure 4-source data 1/Fig.4C-HSP70.tif]

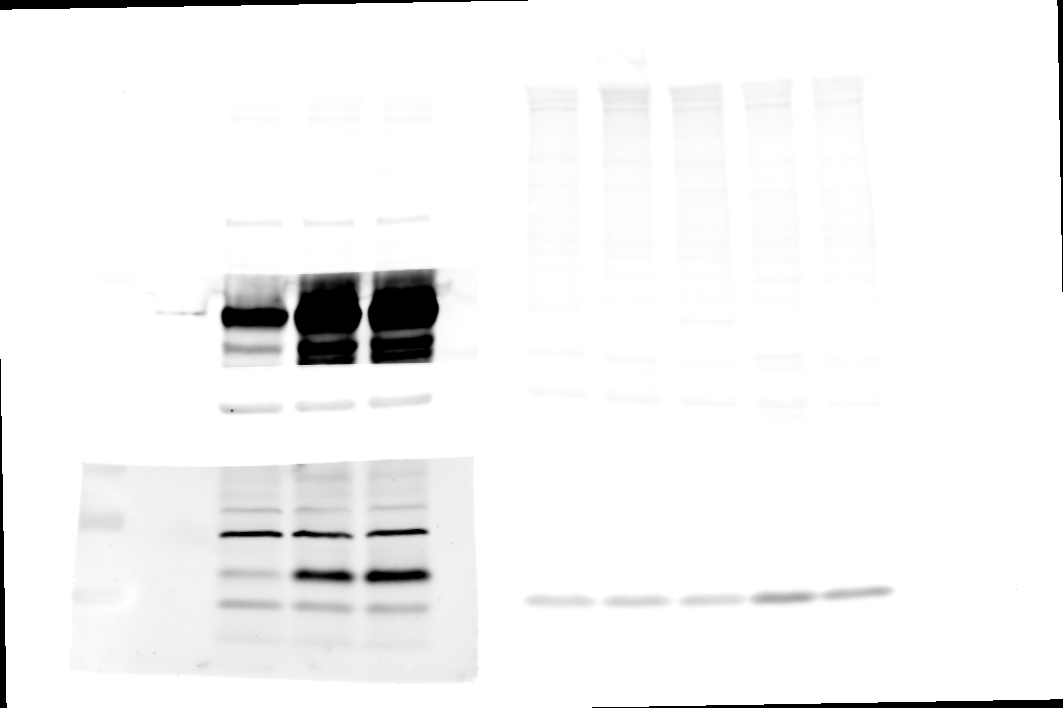

Supplement: Figure 4—source data 1. — The tiff files correspond to uncropped pictures of the IRDye800CW fluorescence signal acquired on a LI-COR Odyssey. The regions used to generate the figure are highlighted by back squares in the jpg file. [file elife-73913-fig4-data1.zip › Figure 4-source data 1/Fig.4C-p21.tif]

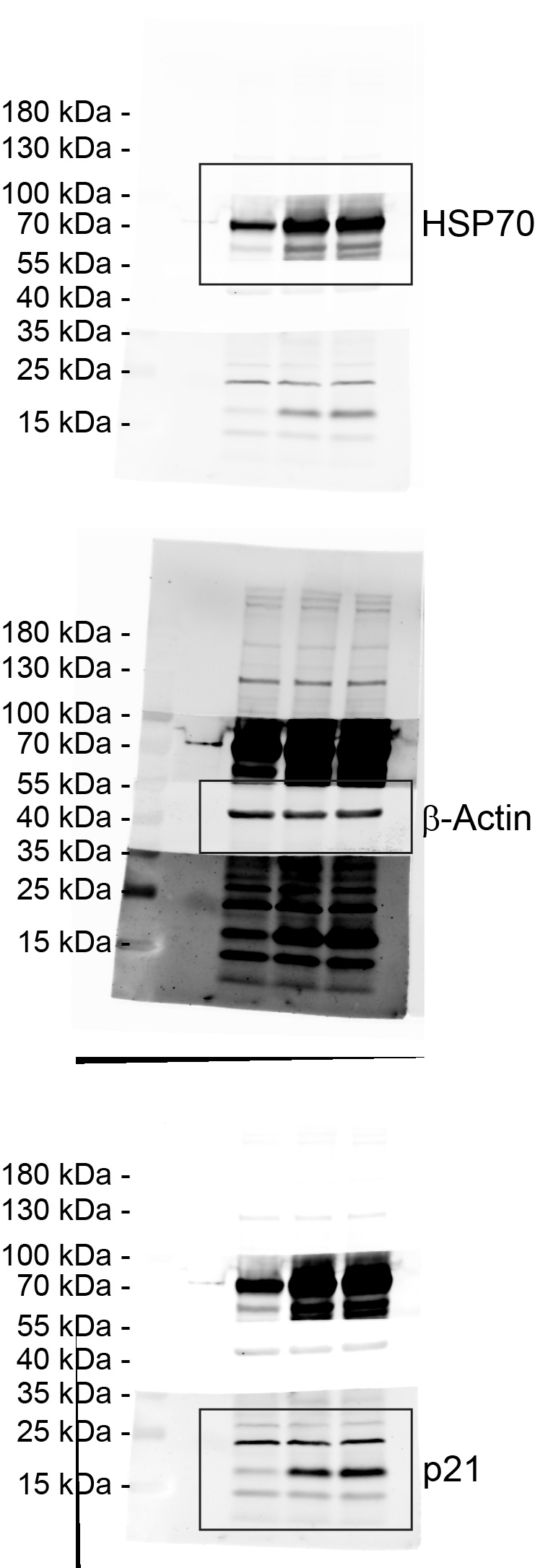

Supplement: Figure 4—source data 1. — The tiff files correspond to uncropped pictures of the IRDye800CW fluorescence signal acquired on a LI-COR Odyssey. The regions used to generate the figure are highlighted by back squares in the jpg file. [file elife-73913-fig4-data1.zip › Figure 4-source data 1/Fig.4C.jpg]

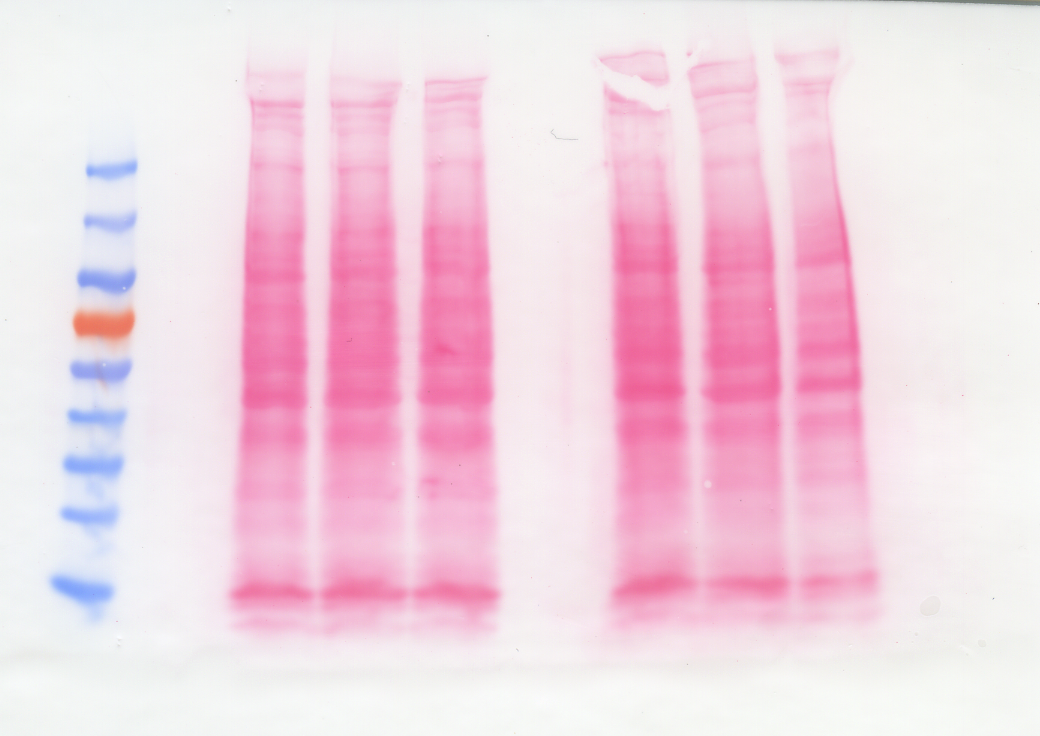

Supplement: Figure 4—source data 2. — The tiff files correspond to an uncropped picture of the IRDye800CW fluorescence signal, acquired on an Odyssey LI-COR, and of a scan of the membrane stained with Ponceau S. The regions used to generate the figure are highlighted by back squares in the jpg file. [file elife-73913-fig4-data2.zip › Figure 4-source data 2/Fig.4D-Ponceau.tif]

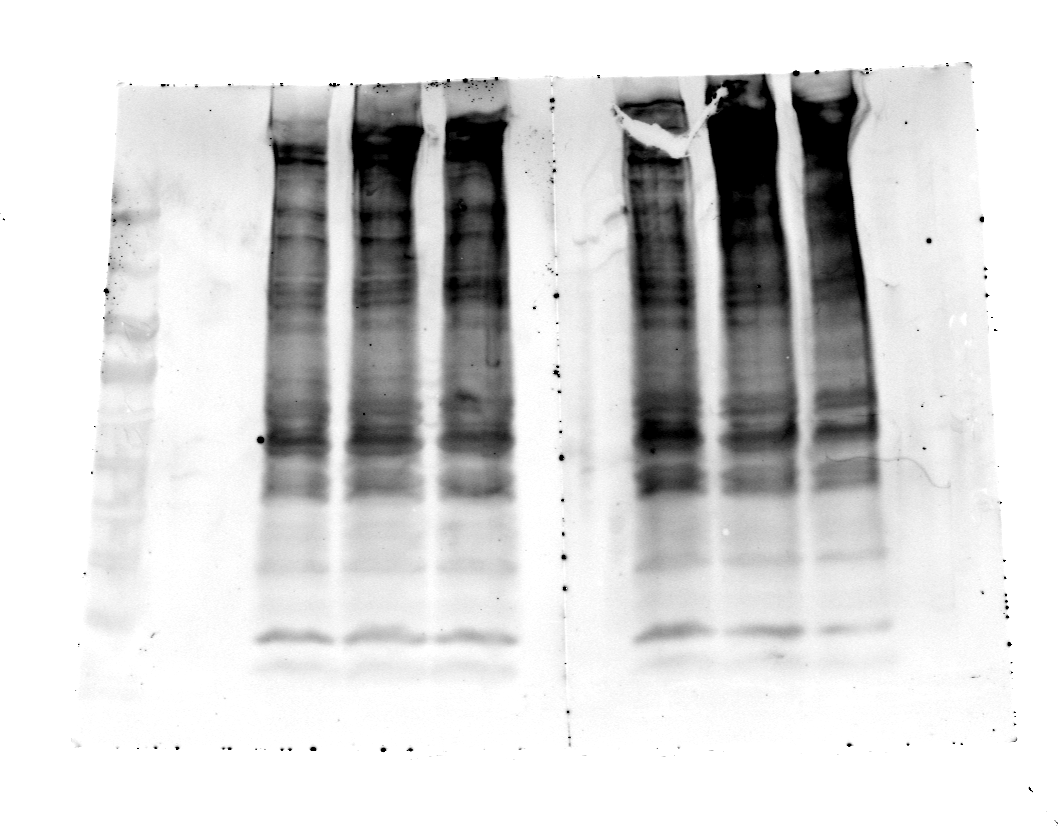

Supplement: Figure 4—source data 2. — The tiff files correspond to an uncropped picture of the IRDye800CW fluorescence signal, acquired on an Odyssey LI-COR, and of a scan of the membrane stained with Ponceau S. The regions used to generate the figure are highlighted by back squares in the jpg file. [file elife-73913-fig4-data2.zip › Figure 4-source data 2/Fig.4D-Ubiquitin.tif]

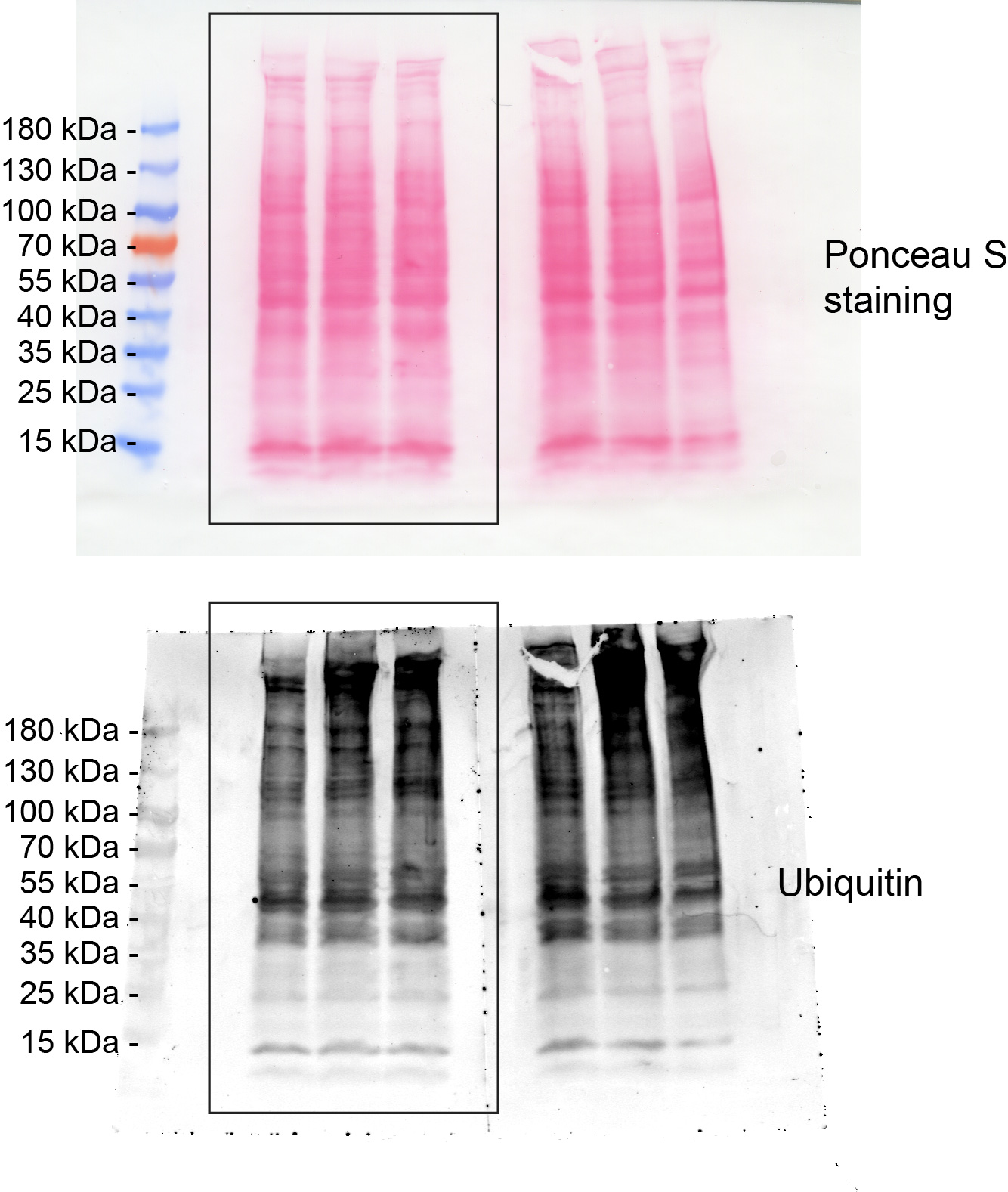

Supplement: Figure 4—source data 2. — The tiff files correspond to an uncropped picture of the IRDye800CW fluorescence signal, acquired on an Odyssey LI-COR, and of a scan of the membrane stained with Ponceau S. The regions used to generate the figure are highlighted by back squares in the jpg file. [file elife-73913-fig4-data2.zip › Figure 4-source data 2/Fig.4D.jpg]

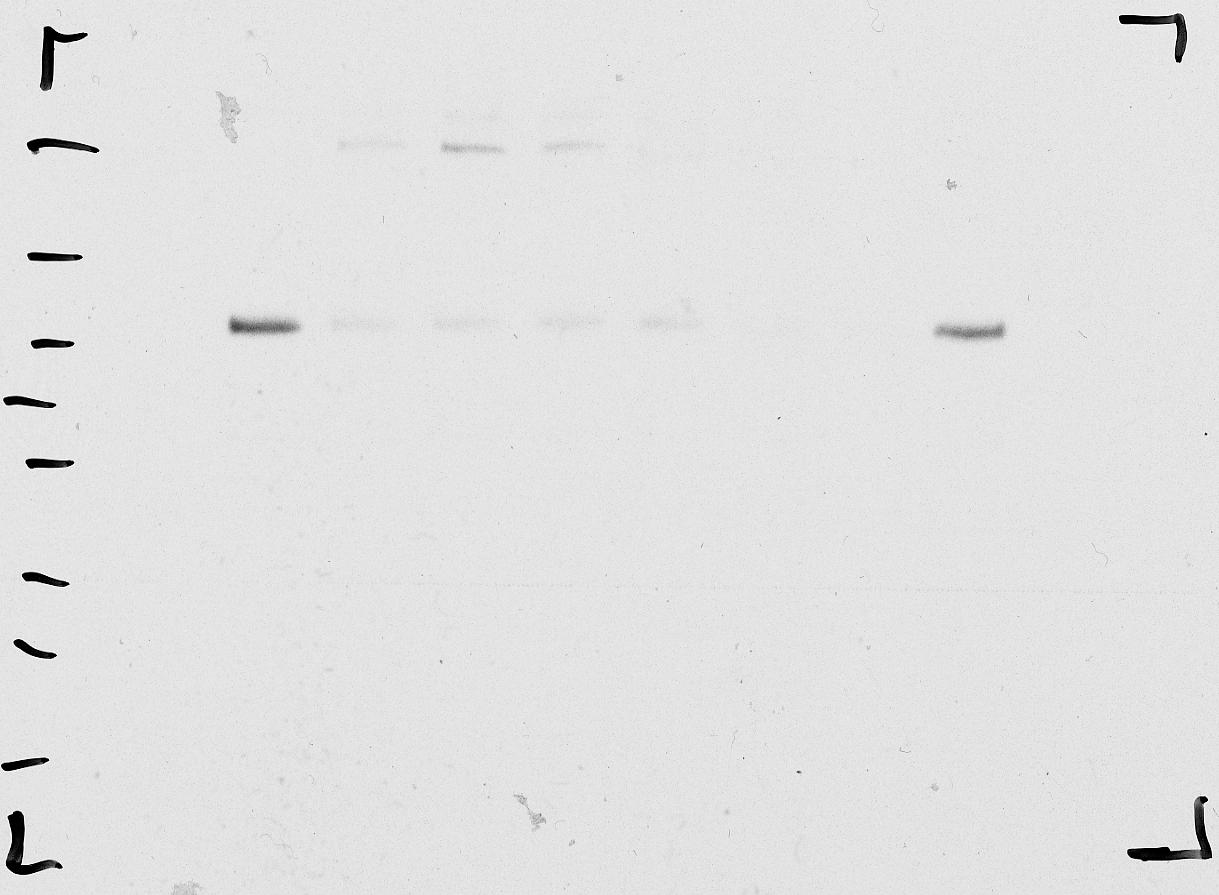

Supplement: Figure 4—source data 3. — The tiff files correspond to uncropped pictures of the chemiluminescent signal acquired on a BioRad Chemidoc, of the IRDye800CW fluorescence signal acquired on a LI-COR Odyssey or of the chemiluminescent signal acquired using autoradiographic films. Several immunoblotting, labeled from a to f, of the same extracts were used to generate this figure, all with a loading control. The regions used to generate the figure are highlighted for each immunoblot by back squares in the jpg files. [file elife-73913-fig4-data3.zip › Figure 4-source data 3/Fig.4Ga-ATF6.tif]

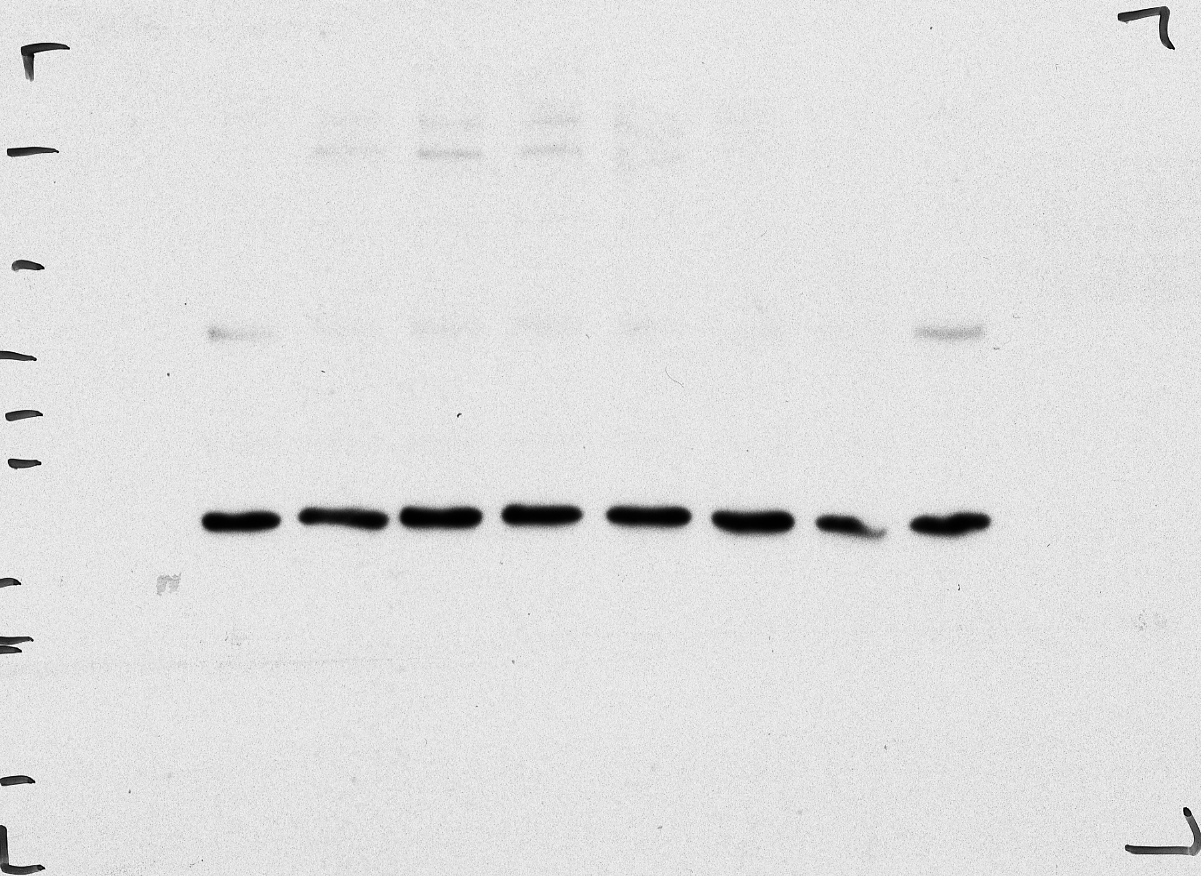

Supplement: Figure 4—source data 3. — The tiff files correspond to uncropped pictures of the chemiluminescent signal acquired on a BioRad Chemidoc, of the IRDye800CW fluorescence signal acquired on a LI-COR Odyssey or of the chemiluminescent signal acquired using autoradiographic films. Several immunoblotting, labeled from a to f, of the same extracts were used to generate this figure, all with a loading control. The regions used to generate the figure are highlighted for each immunoblot by back squares in the jpg files. [file elife-73913-fig4-data3.zip › Figure 4-source data 3/Fig.4Ga-b-ACTIN.tif]

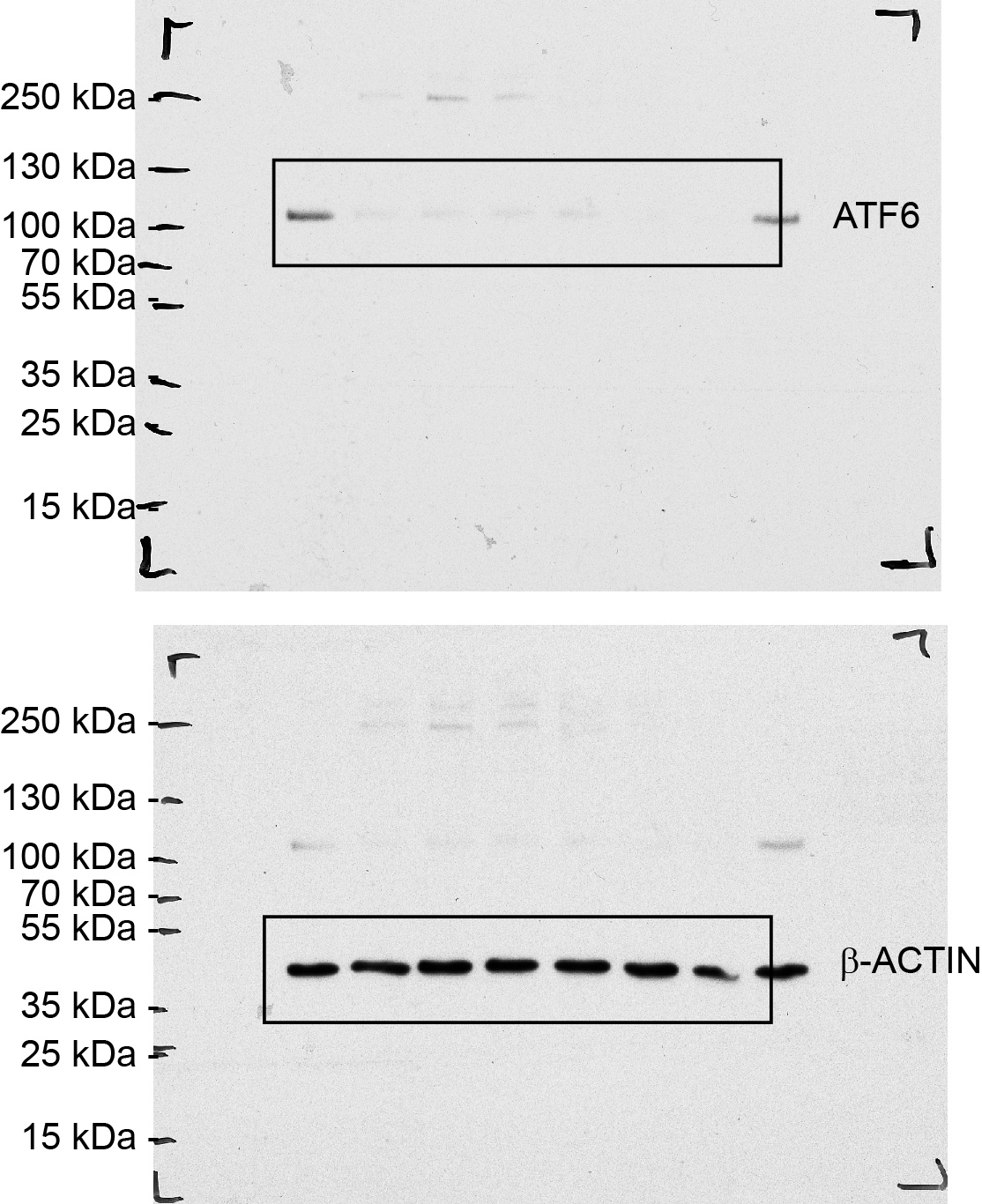

Supplement: Figure 4—source data 3. — The tiff files correspond to uncropped pictures of the chemiluminescent signal acquired on a BioRad Chemidoc, of the IRDye800CW fluorescence signal acquired on a LI-COR Odyssey or of the chemiluminescent signal acquired using autoradiographic films. Several immunoblotting, labeled from a to f, of the same extracts were used to generate this figure, all with a loading control. The regions used to generate the figure are highlighted for each immunoblot by back squares in the jpg files. [file elife-73913-fig4-data3.zip › Figure 4-source data 3/Fig.4Ga.jpg]

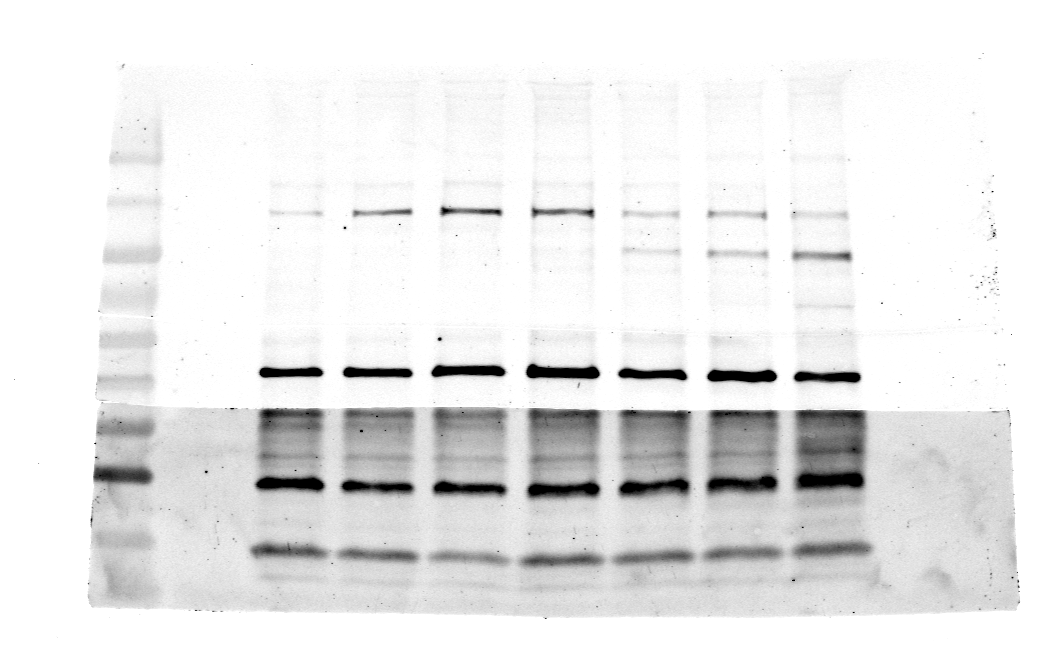

Supplement: Figure 4—source data 3. — The tiff files correspond to uncropped pictures of the chemiluminescent signal acquired on a BioRad Chemidoc, of the IRDye800CW fluorescence signal acquired on a LI-COR Odyssey or of the chemiluminescent signal acquired using autoradiographic films. Several immunoblotting, labeled from a to f, of the same extracts were used to generate this figure, all with a loading control. The regions used to generate the figure are highlighted for each immunoblot by back squares in the jpg files. [file elife-73913-fig4-data3.zip › Figure 4-source data 3/Fig.4Gb-H2AX.tif]

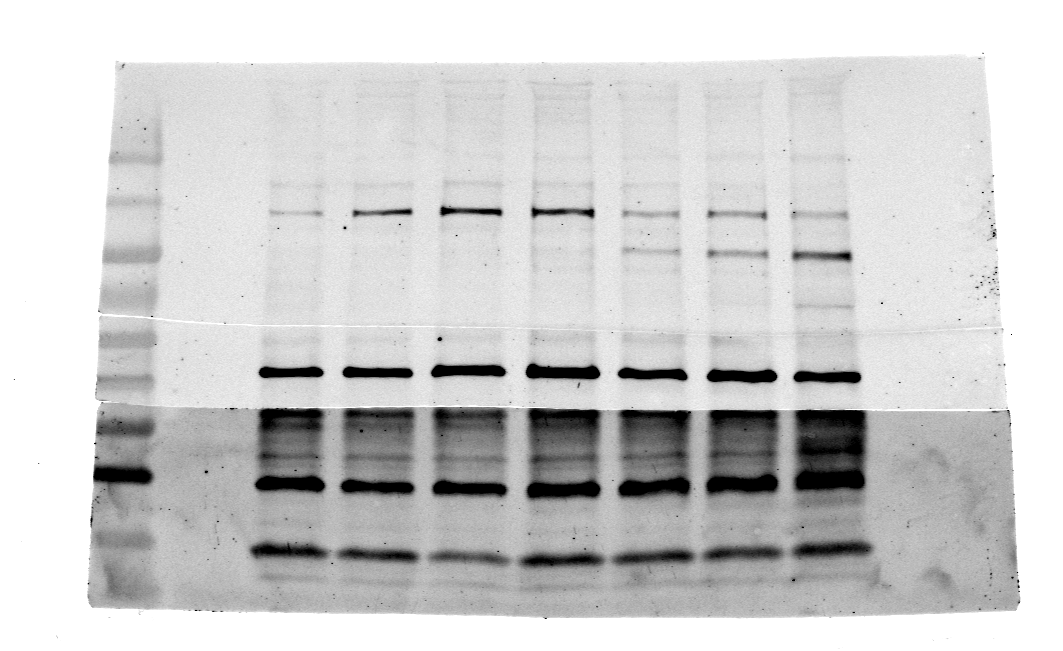

Supplement: Figure 4—source data 3. — The tiff files correspond to uncropped pictures of the chemiluminescent signal acquired on a BioRad Chemidoc, of the IRDye800CW fluorescence signal acquired on a LI-COR Odyssey or of the chemiluminescent signal acquired using autoradiographic films. Several immunoblotting, labeled from a to f, of the same extracts were used to generate this figure, all with a loading control. The regions used to generate the figure are highlighted for each immunoblot by back squares in the jpg files. [file elife-73913-fig4-data3.zip › Figure 4-source data 3/Fig.4Gb-PARP1.tif]

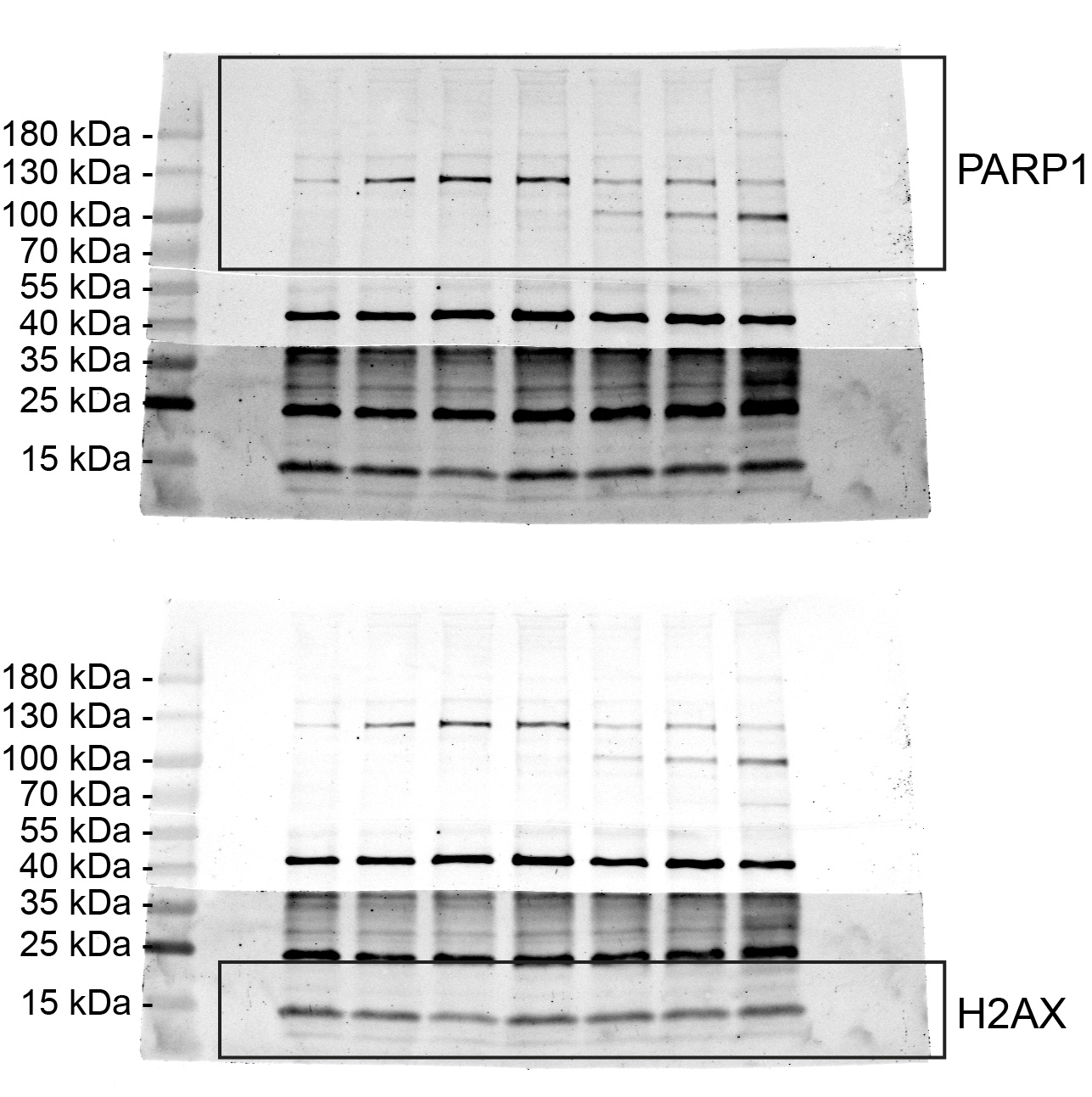

Supplement: Figure 4—source data 3. — The tiff files correspond to uncropped pictures of the chemiluminescent signal acquired on a BioRad Chemidoc, of the IRDye800CW fluorescence signal acquired on a LI-COR Odyssey or of the chemiluminescent signal acquired using autoradiographic films. Several immunoblotting, labeled from a to f, of the same extracts were used to generate this figure, all with a loading control. The regions used to generate the figure are highlighted for each immunoblot by back squares in the jpg files. [file elife-73913-fig4-data3.zip › Figure 4-source data 3/Fig.4Gb.jpg]

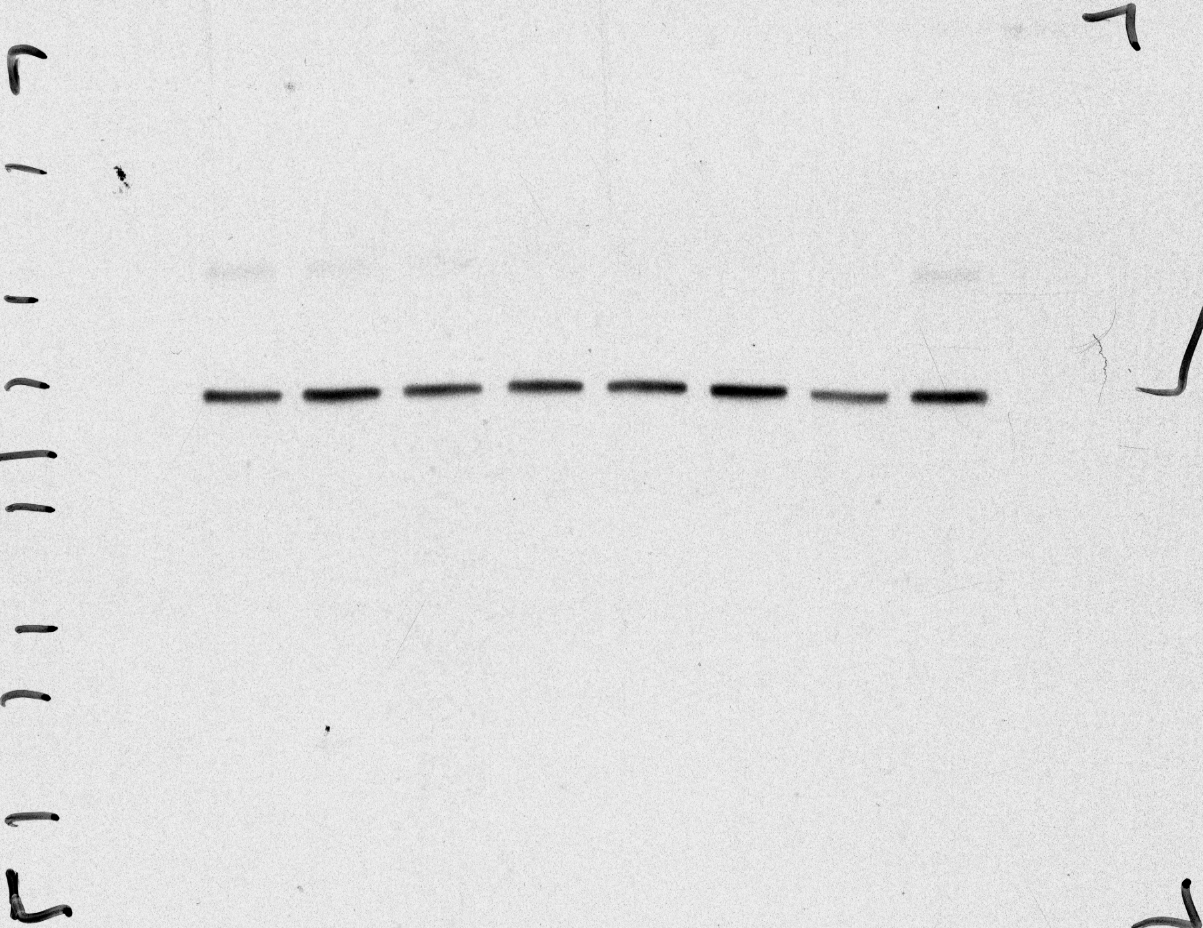

Supplement: Figure 4—source data 3. — The tiff files correspond to uncropped pictures of the chemiluminescent signal acquired on a BioRad Chemidoc, of the IRDye800CW fluorescence signal acquired on a LI-COR Odyssey or of the chemiluminescent signal acquired using autoradiographic films. Several immunoblotting, labeled from a to f, of the same extracts were used to generate this figure, all with a loading control. The regions used to generate the figure are highlighted for each immunoblot by back squares in the jpg files. [file elife-73913-fig4-data3.zip › Figure 4-source data 3/Fig.4Gc-Ku80.tif]

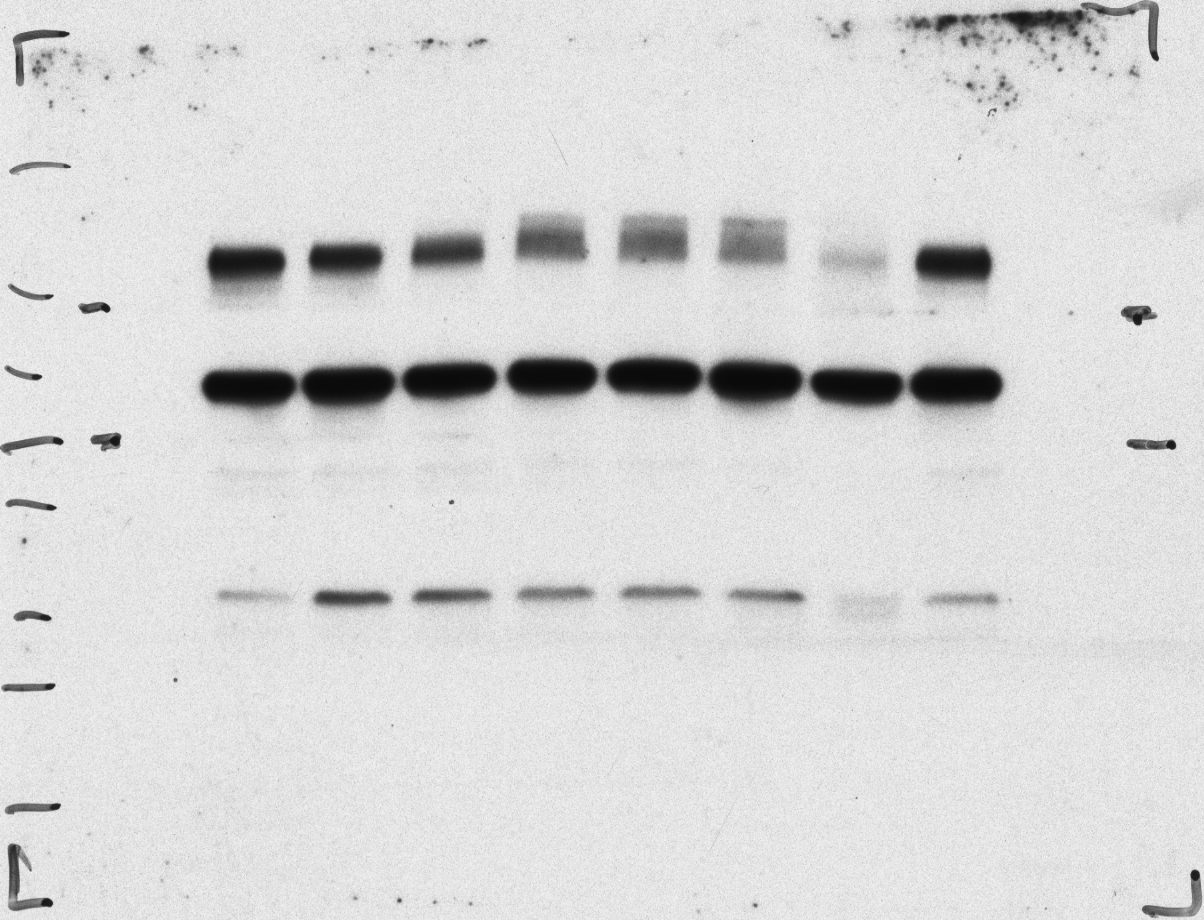

Supplement: Figure 4—source data 3. — The tiff files correspond to uncropped pictures of the chemiluminescent signal acquired on a BioRad Chemidoc, of the IRDye800CW fluorescence signal acquired on a LI-COR Odyssey or of the chemiluminescent signal acquired using autoradiographic films. Several immunoblotting, labeled from a to f, of the same extracts were used to generate this figure, all with a loading control. The regions used to generate the figure are highlighted for each immunoblot by back squares in the jpg files. [file elife-73913-fig4-data3.zip › Figure 4-source data 3/Fig.4Gc-PERK-PheIF2A.tif]

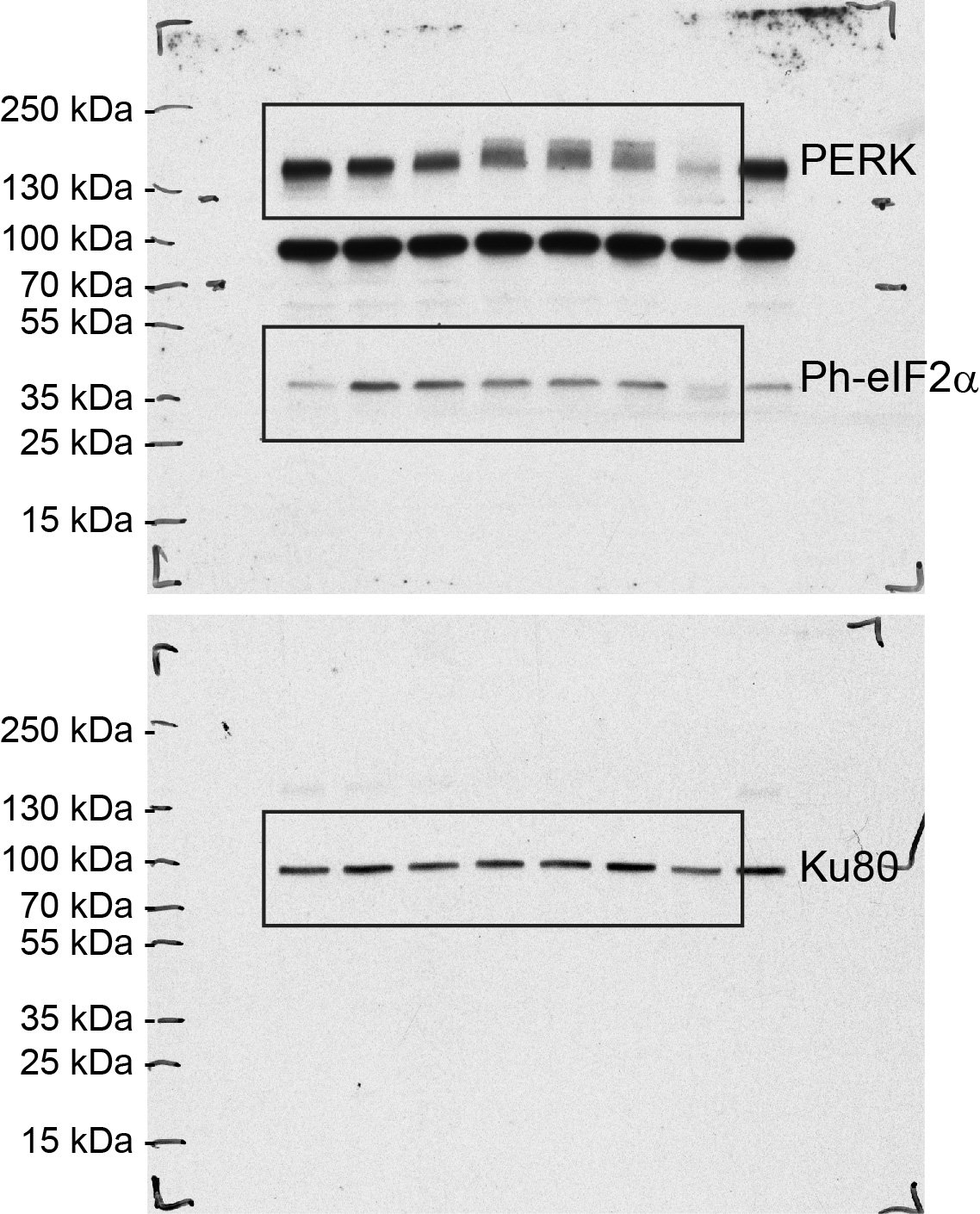

Supplement: Figure 4—source data 3. — The tiff files correspond to uncropped pictures of the chemiluminescent signal acquired on a BioRad Chemidoc, of the IRDye800CW fluorescence signal acquired on a LI-COR Odyssey or of the chemiluminescent signal acquired using autoradiographic films. Several immunoblotting, labeled from a to f, of the same extracts were used to generate this figure, all with a loading control. The regions used to generate the figure are highlighted for each immunoblot by back squares in the jpg files. [file elife-73913-fig4-data3.zip › Figure 4-source data 3/Fig.4Gc.jpg]

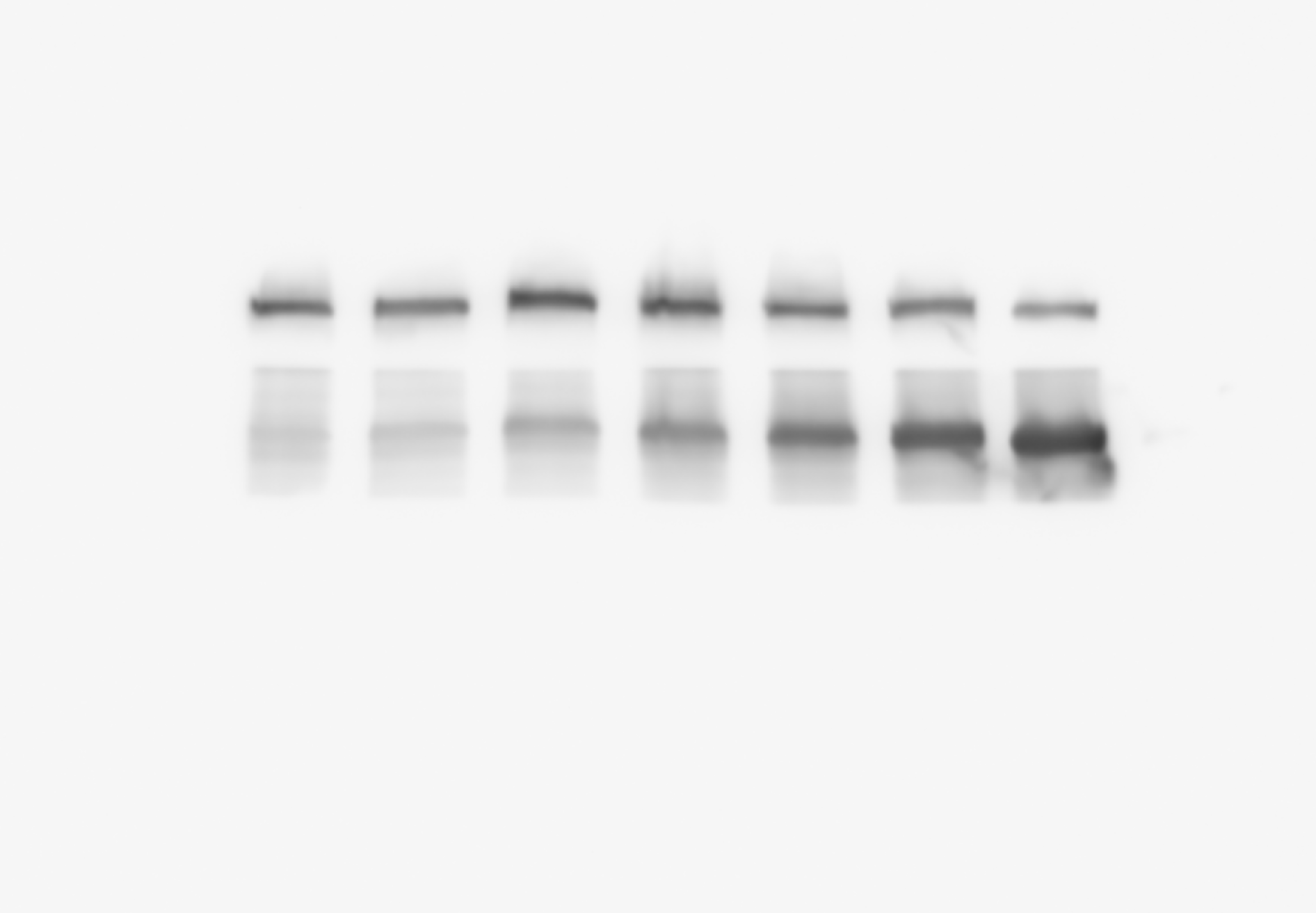

Supplement: Figure 4—source data 3. — The tiff files correspond to uncropped pictures of the chemiluminescent signal acquired on a BioRad Chemidoc, of the IRDye800CW fluorescence signal acquired on a LI-COR Odyssey or of the chemiluminescent signal acquired using autoradiographic films. Several immunoblotting, labeled from a to f, of the same extracts were used to generate this figure, all with a loading control. The regions used to generate the figure are highlighted for each immunoblot by back squares in the jpg files. [file elife-73913-fig4-data3.zip › Figure 4-source data 3/Fig.4Gd-HSP70-SAFA.tif]

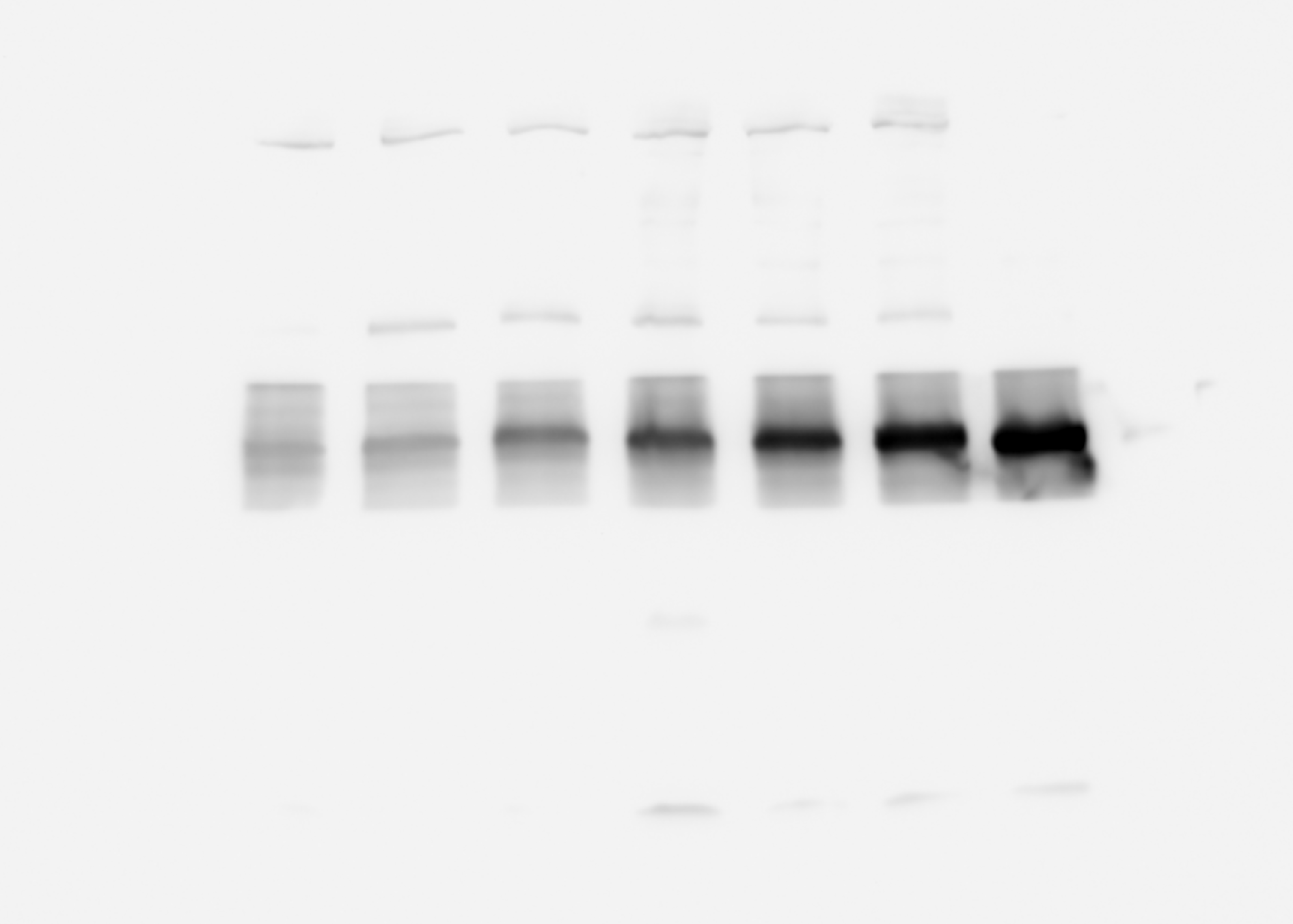

Supplement: Figure 4—source data 3. — The tiff files correspond to uncropped pictures of the chemiluminescent signal acquired on a BioRad Chemidoc, of the IRDye800CW fluorescence signal acquired on a LI-COR Odyssey or of the chemiluminescent signal acquired using autoradiographic films. Several immunoblotting, labeled from a to f, of the same extracts were used to generate this figure, all with a loading control. The regions used to generate the figure are highlighted for each immunoblot by back squares in the jpg files. [file elife-73913-fig4-data3.zip › Figure 4-source data 3/Fig.4Gd-HSP70.tif]

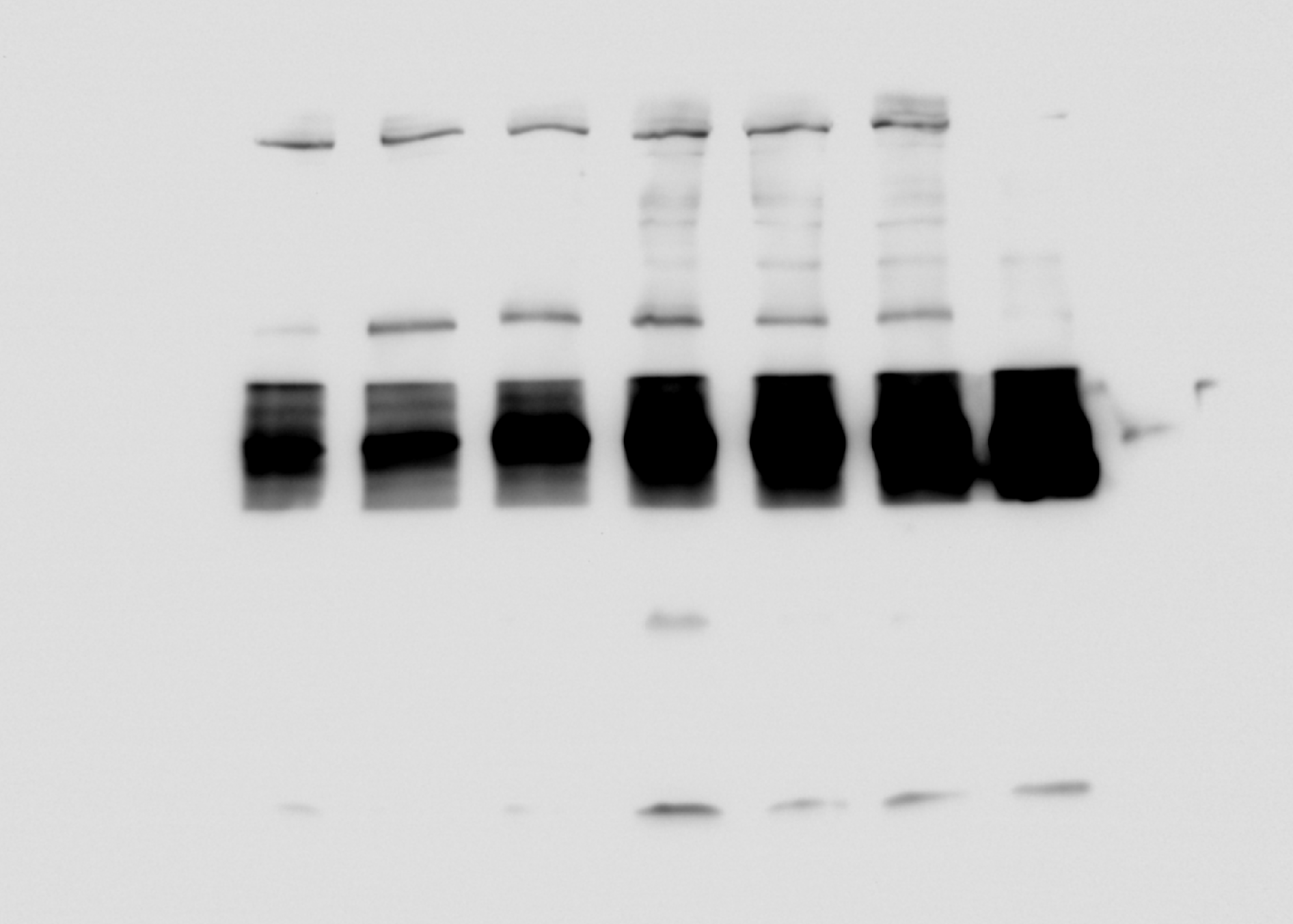

Supplement: Figure 4—source data 3. — The tiff files correspond to uncropped pictures of the chemiluminescent signal acquired on a BioRad Chemidoc, of the IRDye800CW fluorescence signal acquired on a LI-COR Odyssey or of the chemiluminescent signal acquired using autoradiographic films. Several immunoblotting, labeled from a to f, of the same extracts were used to generate this figure, all with a loading control. The regions used to generate the figure are highlighted for each immunoblot by back squares in the jpg files. [file elife-73913-fig4-data3.zip › Figure 4-source data 3/Fig.4Gd-PhIRE1a.tif]

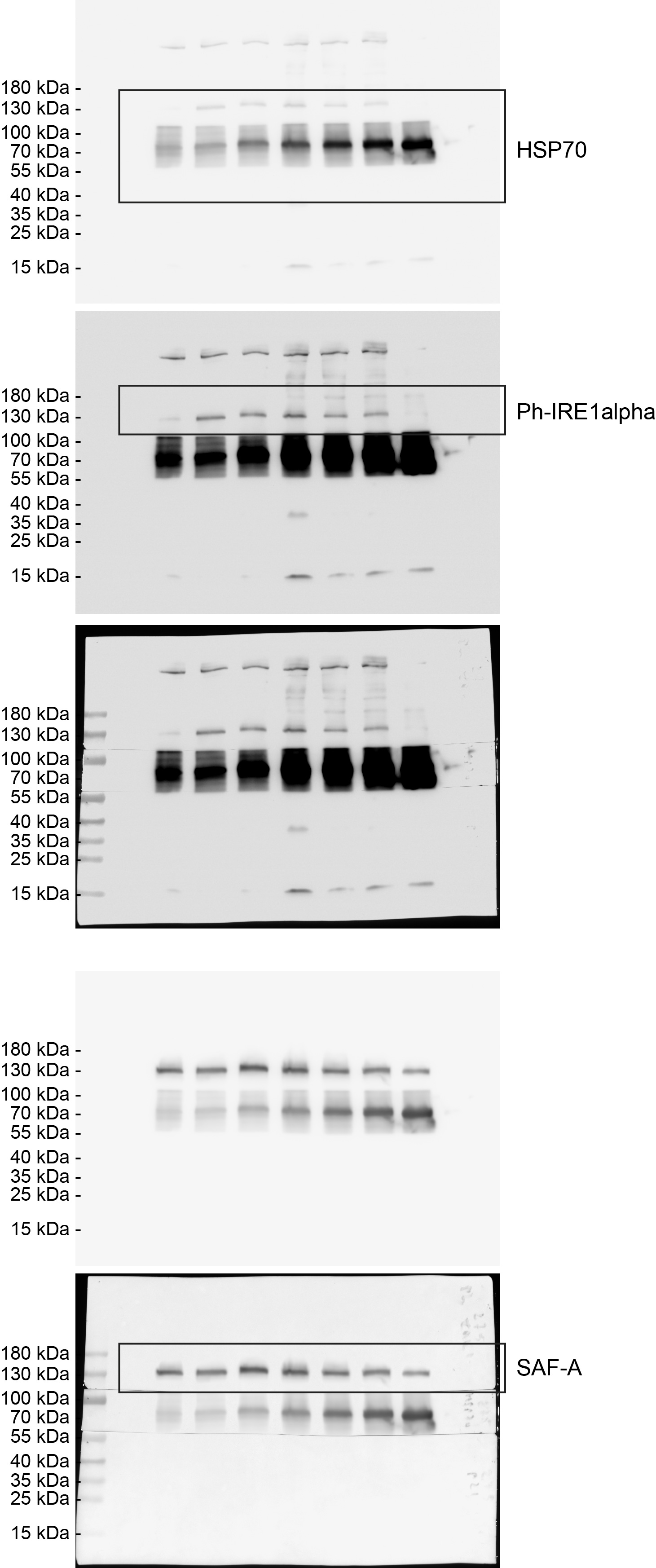

Supplement: Figure 4—source data 3. — The tiff files correspond to uncropped pictures of the chemiluminescent signal acquired on a BioRad Chemidoc, of the IRDye800CW fluorescence signal acquired on a LI-COR Odyssey or of the chemiluminescent signal acquired using autoradiographic films. Several immunoblotting, labeled from a to f, of the same extracts were used to generate this figure, all with a loading control. The regions used to generate the figure are highlighted for each immunoblot by back squares in the jpg files. [file elife-73913-fig4-data3.zip › Figure 4-source data 3/Fig.4Gd.jpg]

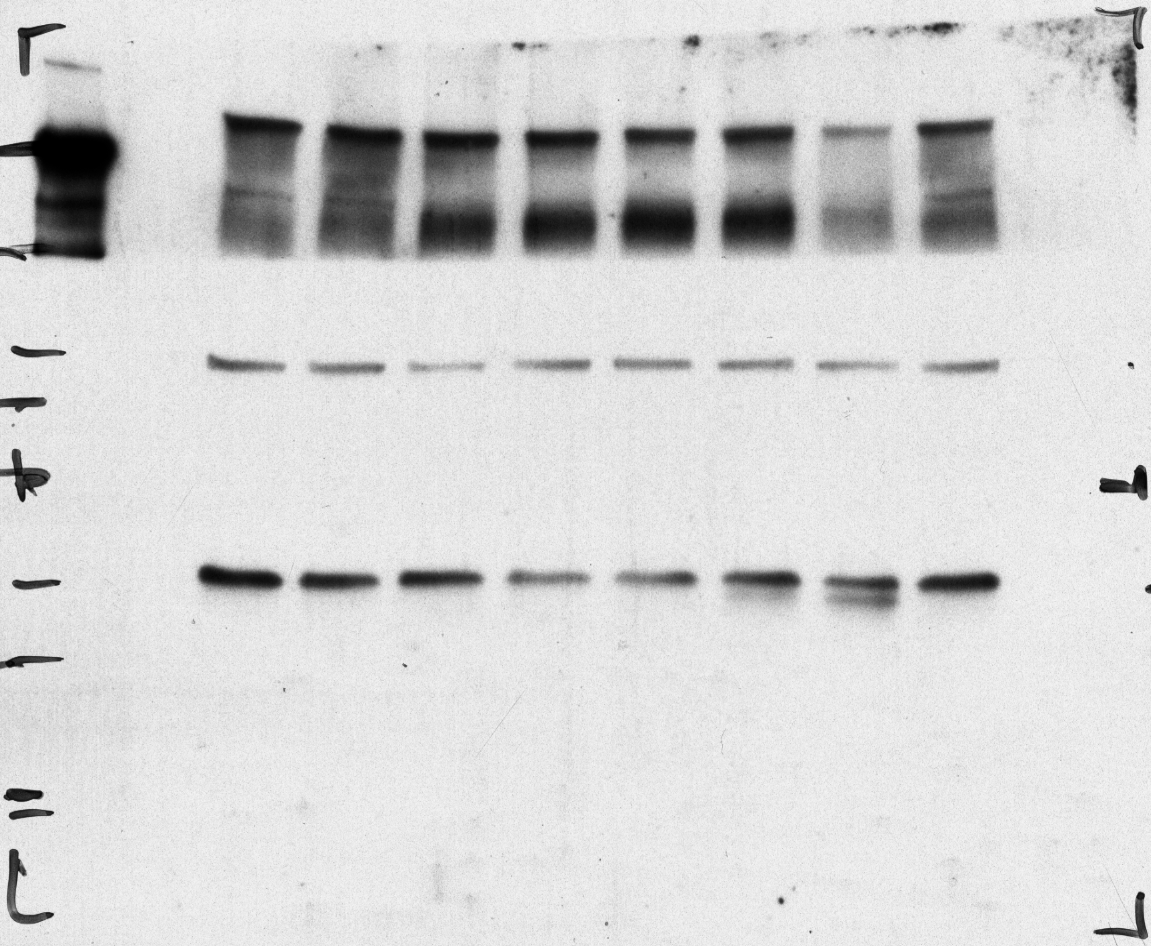

Supplement: Figure 4—source data 3. — The tiff files correspond to uncropped pictures of the chemiluminescent signal acquired on a BioRad Chemidoc, of the IRDye800CW fluorescence signal acquired on a LI-COR Odyssey or of the chemiluminescent signal acquired using autoradiographic films. Several immunoblotting, labeled from a to f, of the same extracts were used to generate this figure, all with a loading control. The regions used to generate the figure are highlighted for each immunoblot by back squares in the jpg files. [file elife-73913-fig4-data3.zip › Figure 4-source data 3/Fig.4Ge-eIF2a.tif]

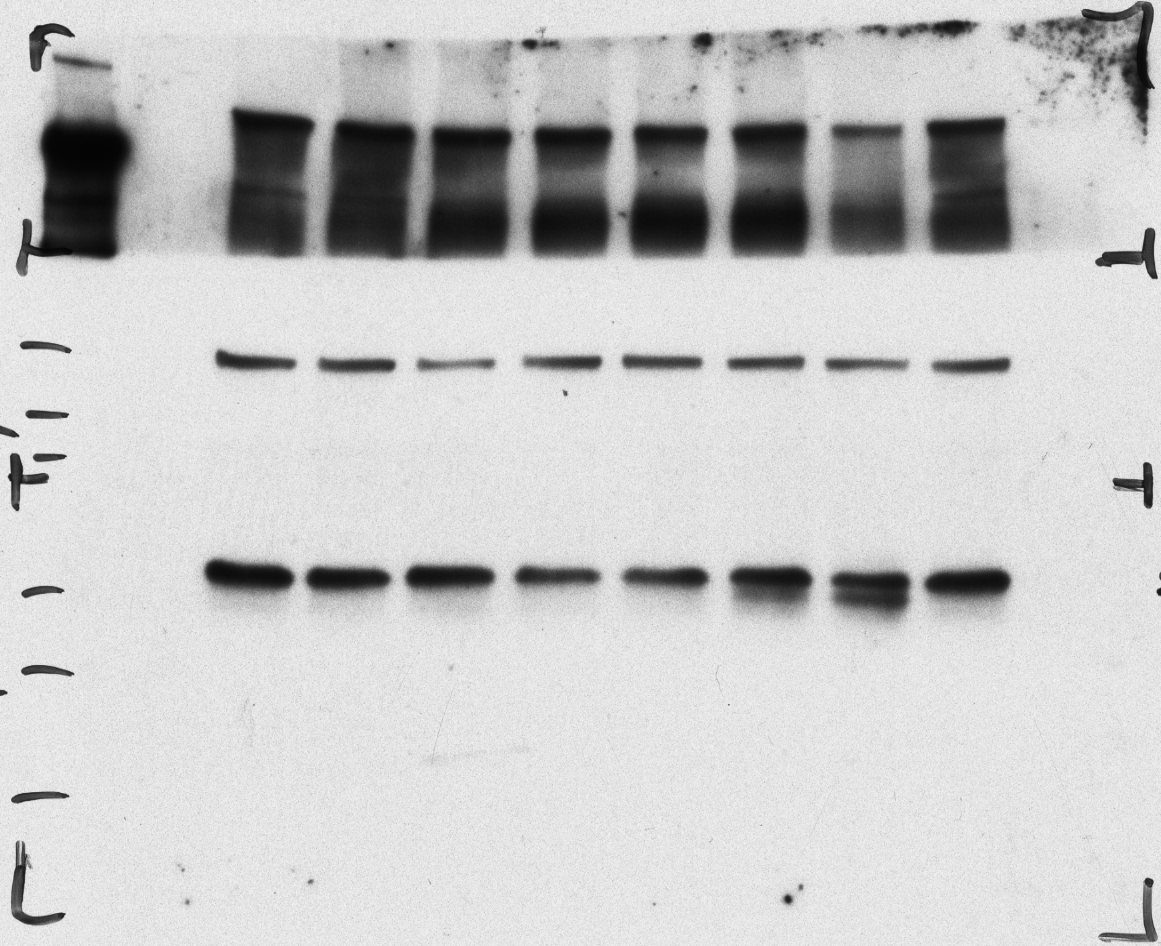

Supplement: Figure 4—source data 3. — The tiff files correspond to uncropped pictures of the chemiluminescent signal acquired on a BioRad Chemidoc, of the IRDye800CW fluorescence signal acquired on a LI-COR Odyssey or of the chemiluminescent signal acquired using autoradiographic films. Several immunoblotting, labeled from a to f, of the same extracts were used to generate this figure, all with a loading control. The regions used to generate the figure are highlighted for each immunoblot by back squares in the jpg files. [file elife-73913-fig4-data3.zip › Figure 4-source data 3/Fig.4Ge-Ku80.tif]

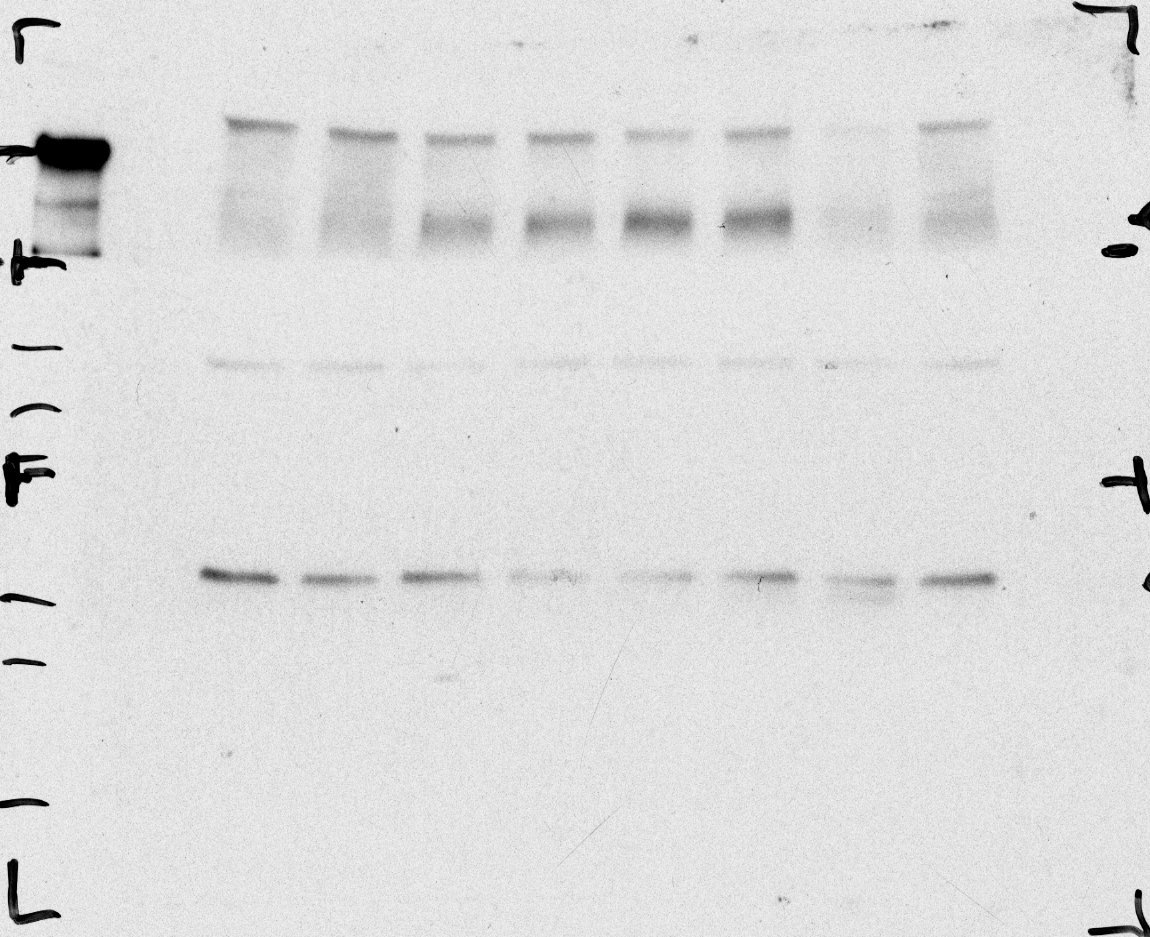

Supplement: Figure 4—source data 3. — The tiff files correspond to uncropped pictures of the chemiluminescent signal acquired on a BioRad Chemidoc, of the IRDye800CW fluorescence signal acquired on a LI-COR Odyssey or of the chemiluminescent signal acquired using autoradiographic films. Several immunoblotting, labeled from a to f, of the same extracts were used to generate this figure, all with a loading control. The regions used to generate the figure are highlighted for each immunoblot by back squares in the jpg files. [file elife-73913-fig4-data3.zip › Figure 4-source data 3/Fig.4Ge-PhPERK.tif]

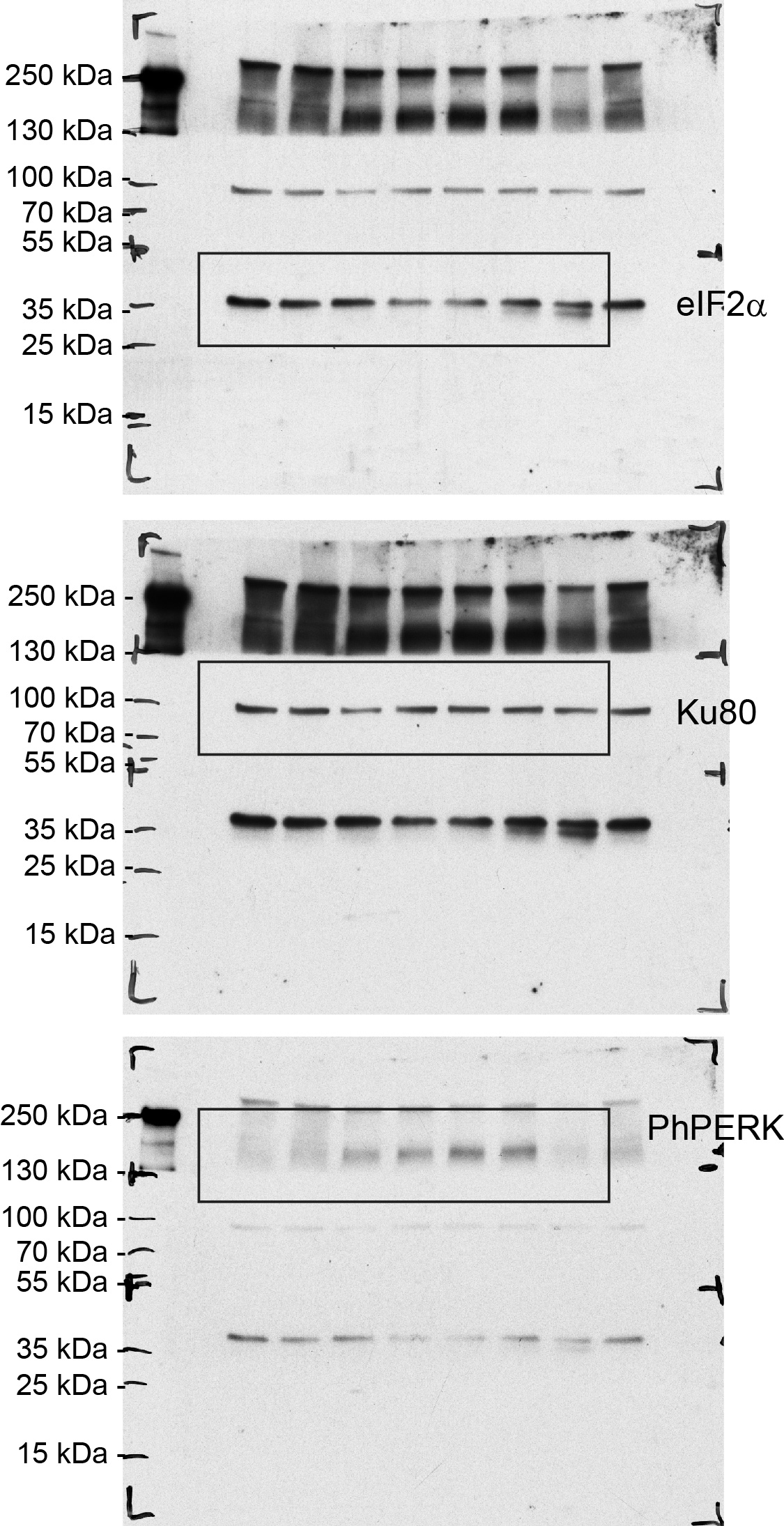

Supplement: Figure 4—source data 3. — The tiff files correspond to uncropped pictures of the chemiluminescent signal acquired on a BioRad Chemidoc, of the IRDye800CW fluorescence signal acquired on a LI-COR Odyssey or of the chemiluminescent signal acquired using autoradiographic films. Several immunoblotting, labeled from a to f, of the same extracts were used to generate this figure, all with a loading control. The regions used to generate the figure are highlighted for each immunoblot by back squares in the jpg files. [file elife-73913-fig4-data3.zip › Figure 4-source data 3/Fig.4Ge.jpg]

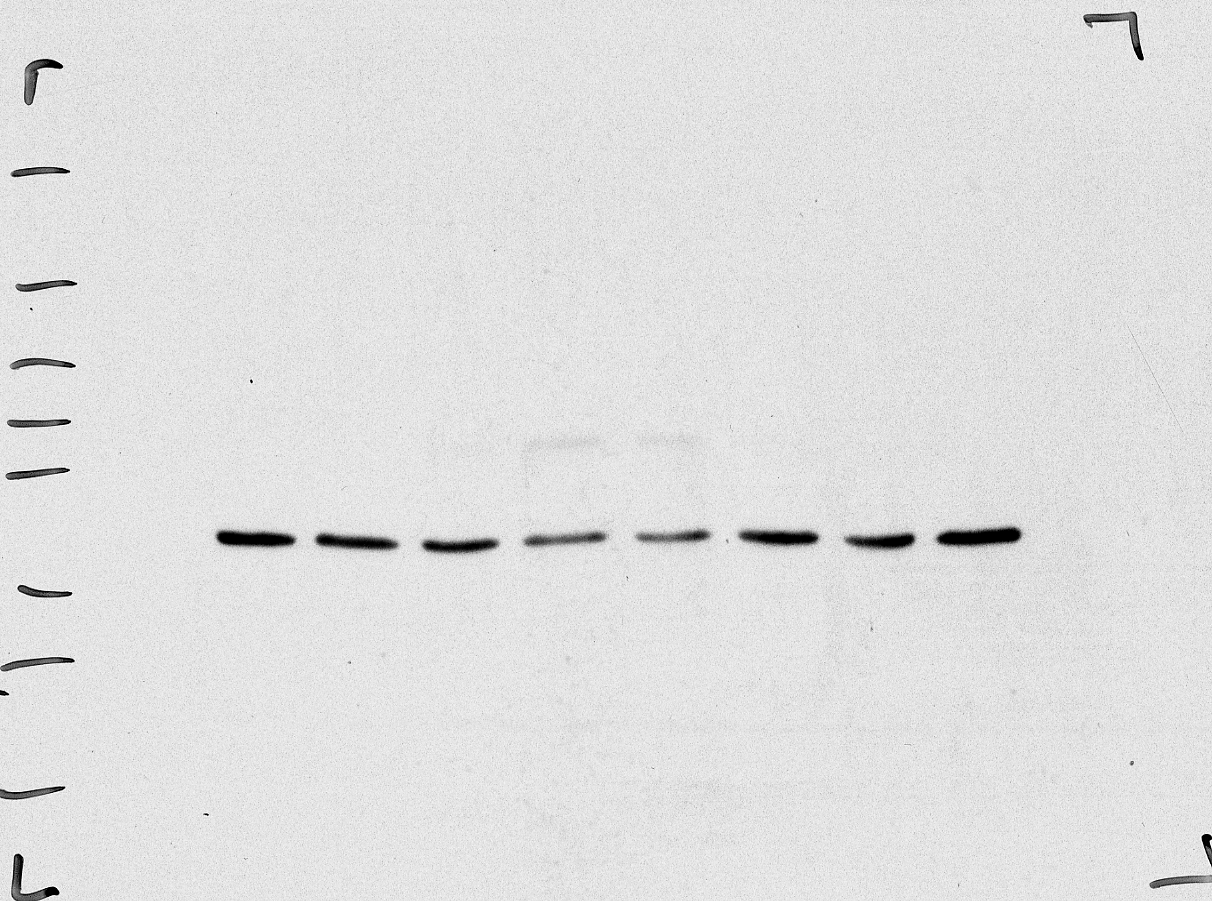

Supplement: Figure 4—source data 3. — The tiff files correspond to uncropped pictures of the chemiluminescent signal acquired on a BioRad Chemidoc, of the IRDye800CW fluorescence signal acquired on a LI-COR Odyssey or of the chemiluminescent signal acquired using autoradiographic films. Several immunoblotting, labeled from a to f, of the same extracts were used to generate this figure, all with a loading control. The regions used to generate the figure are highlighted for each immunoblot by back squares in the jpg files. [file elife-73913-fig4-data3.zip › Figure 4-source data 3/Fig.4Gf-b-ACTIN.tif]

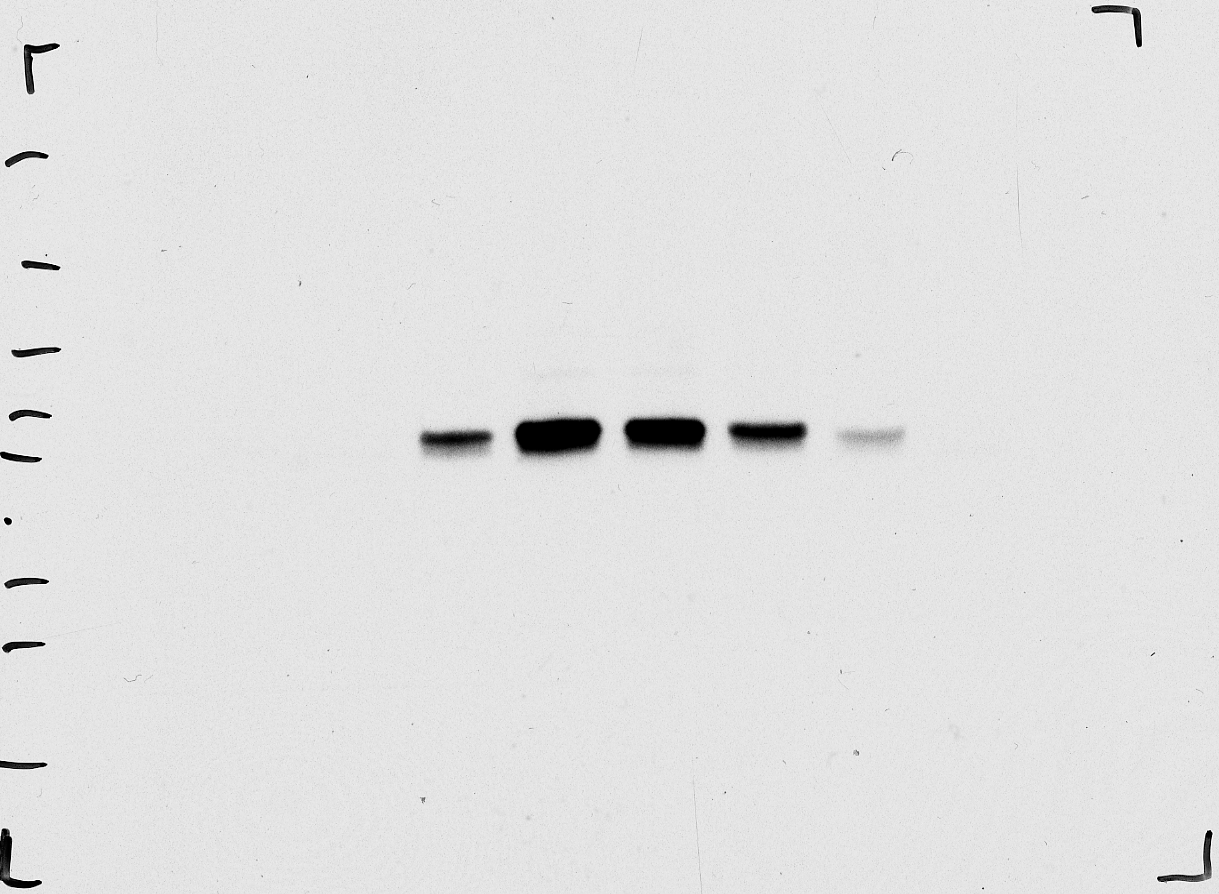

Supplement: Figure 4—source data 3. — The tiff files correspond to uncropped pictures of the chemiluminescent signal acquired on a BioRad Chemidoc, of the IRDye800CW fluorescence signal acquired on a LI-COR Odyssey or of the chemiluminescent signal acquired using autoradiographic films. Several immunoblotting, labeled from a to f, of the same extracts were used to generate this figure, all with a loading control. The regions used to generate the figure are highlighted for each immunoblot by back squares in the jpg files. [file elife-73913-fig4-data3.zip › Figure 4-source data 3/Fig.4Gf-XBP-1s.tif]

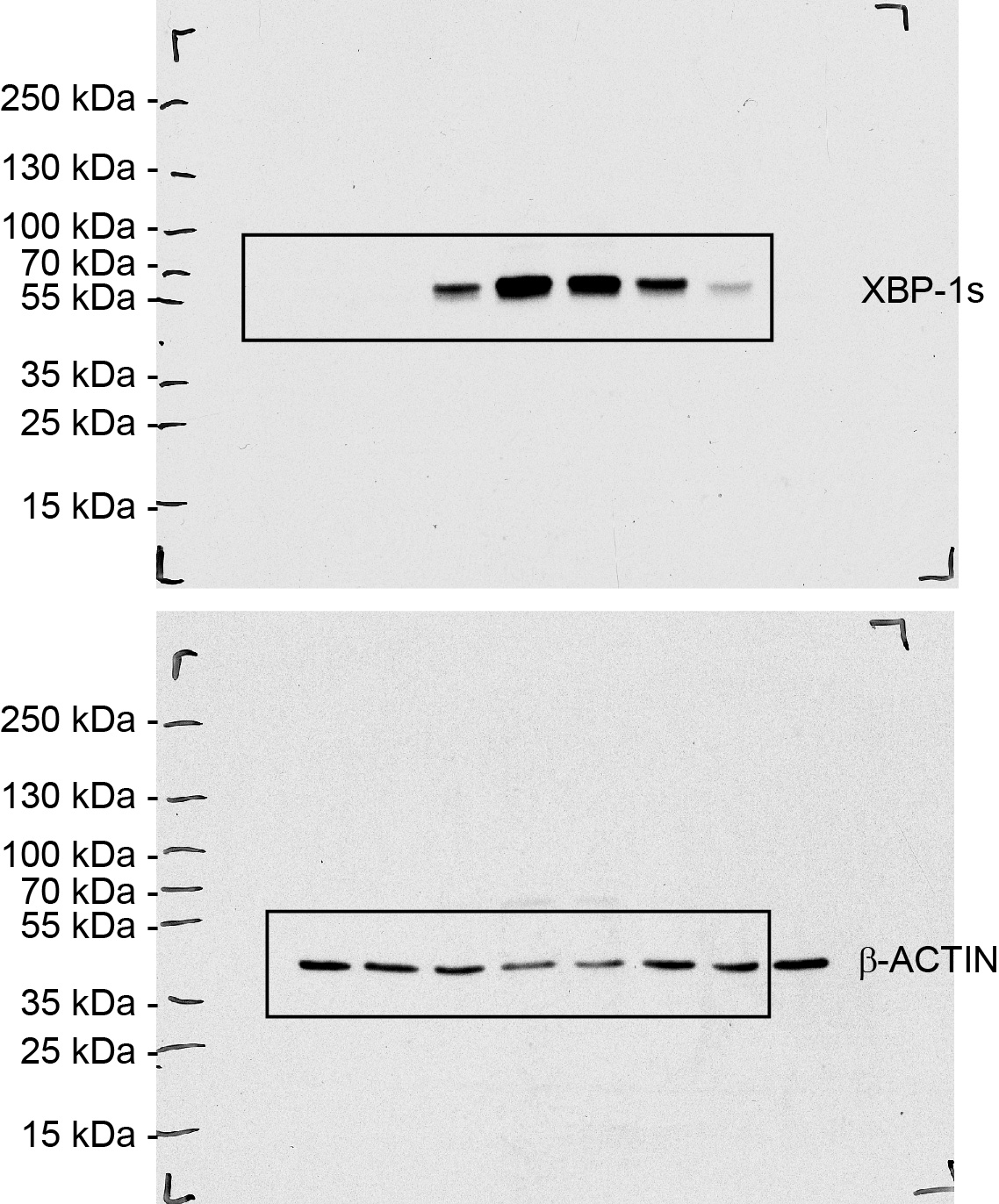

Supplement: Figure 4—source data 3. — The tiff files correspond to uncropped pictures of the chemiluminescent signal acquired on a BioRad Chemidoc, of the IRDye800CW fluorescence signal acquired on a LI-COR Odyssey or of the chemiluminescent signal acquired using autoradiographic films. Several immunoblotting, labeled from a to f, of the same extracts were used to generate this figure, all with a loading control. The regions used to generate the figure are highlighted for each immunoblot by back squares in the jpg files. [file elife-73913-fig4-data3.zip › Figure 4-source data 3/Fig.4Gf.jpg]

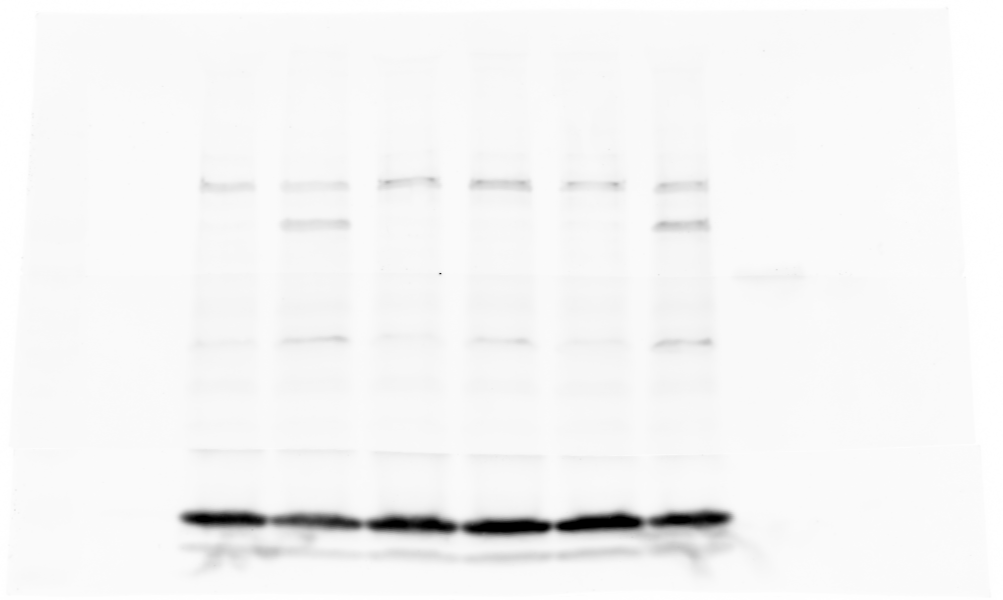

Supplement: Figure 4—source data 4. — The tiff files correspond to uncropped pictures of the IRDye800CW fluorescence signal acquired on a LI-COR Odyssey. The regions used to generate the figure are highlighted by back squares in the jpg file. [file elife-73913-fig4-data4.zip › Figure 4-source data 4/Fig.4H-H2AX.tif]

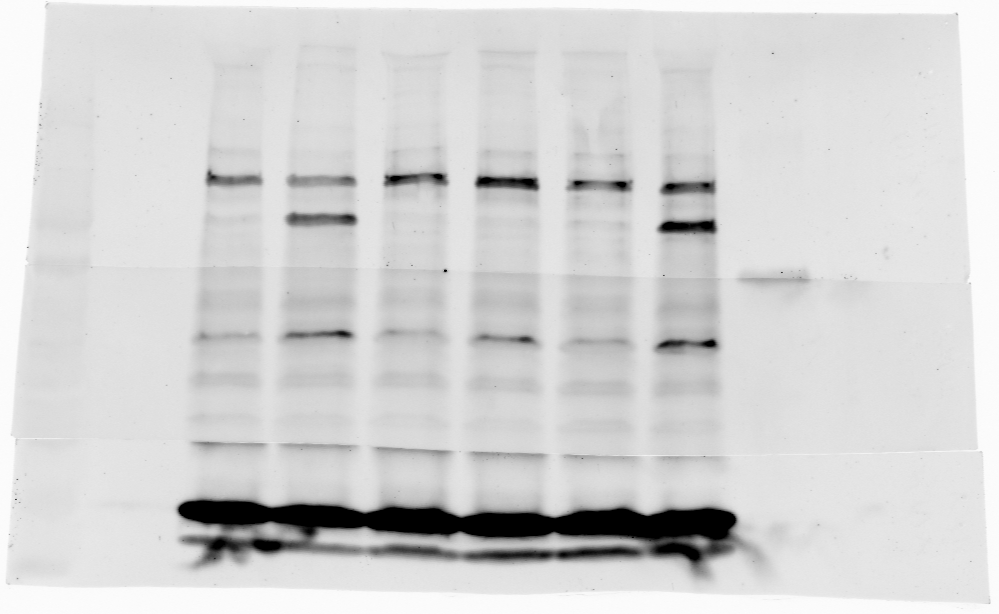

Supplement: Figure 4—source data 4. — The tiff files correspond to uncropped pictures of the IRDye800CW fluorescence signal acquired on a LI-COR Odyssey. The regions used to generate the figure are highlighted by back squares in the jpg file. [file elife-73913-fig4-data4.zip › Figure 4-source data 4/Fig.4H-PARP1.tif]

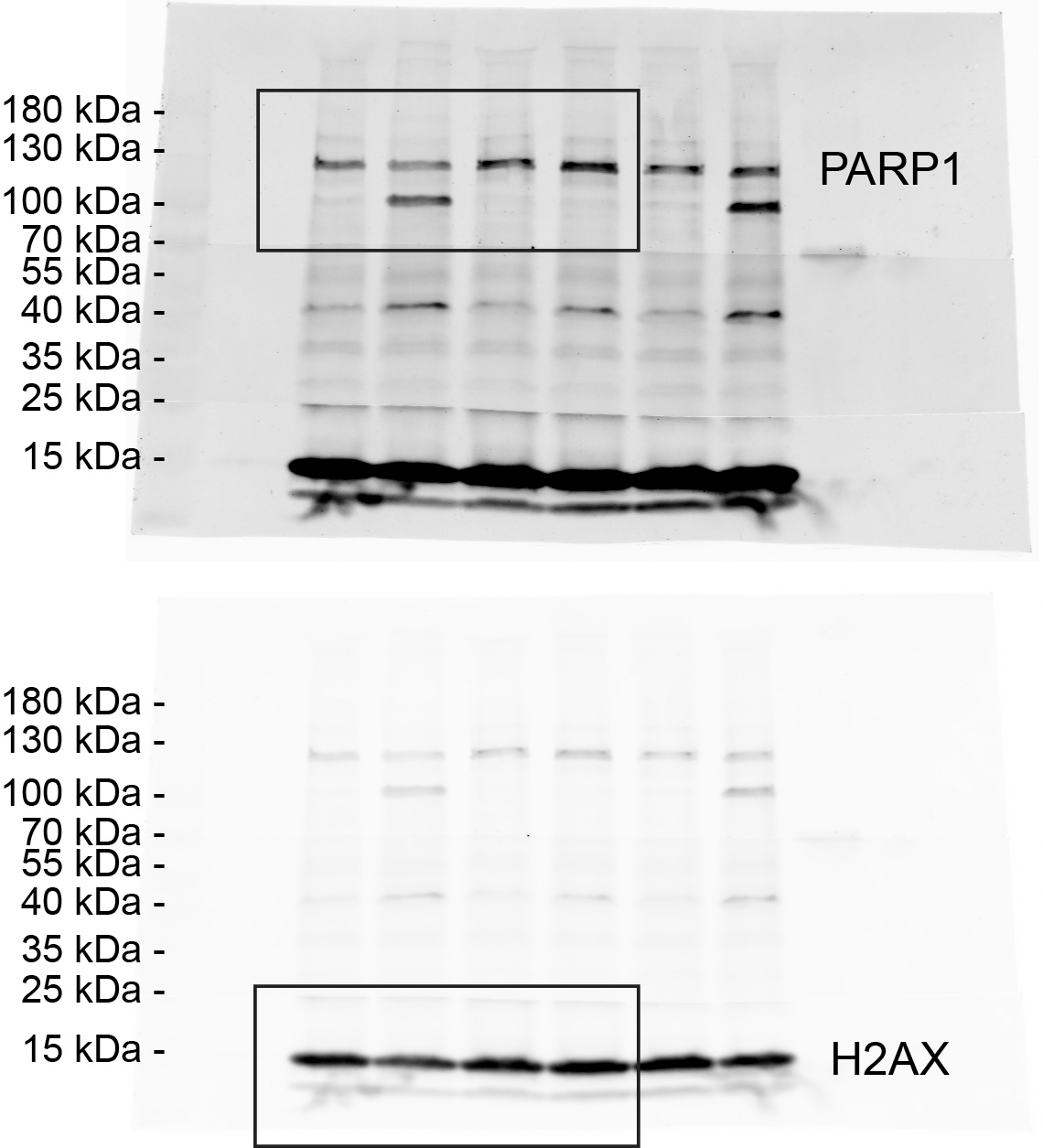

Supplement: Figure 4—source data 4. — The tiff files correspond to uncropped pictures of the IRDye800CW fluorescence signal acquired on a LI-COR Odyssey. The regions used to generate the figure are highlighted by back squares in the jpg file. [file elife-73913-fig4-data4.zip › Figure 4-source data 4/Fig.4H.jpg]

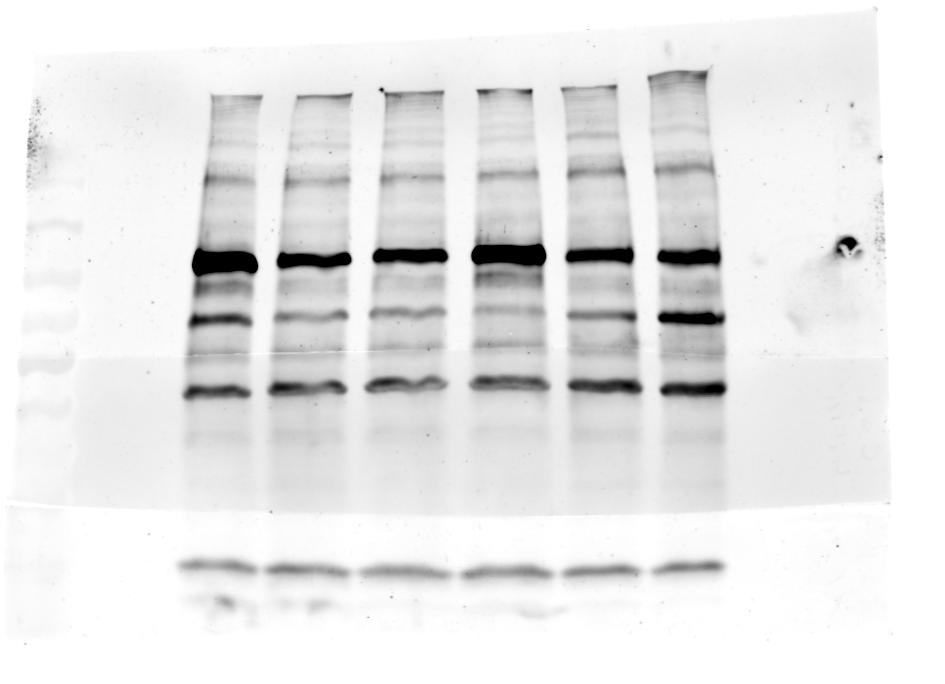

Supplement: Figure 4—figure supplement 1—source data 1. — The tiff files correspond to uncropped pictures of the IRDye800CW fluorescence signal acquired on a LI-COR Odyssey. The regions used to generate the figure are highlighted by back squares in the jpg file. [file elife-73913-fig4-figsupp1-data1.zip › Figure 4-figure supplement 1-source data 1/Fig.4-S1C-beta-Actin.tif]
