# Supplementary figures and images for: SDR enzymes oxidize specific lipidic alkynylcarbinols into cytotoxic protein-reactive species (part 2 of 2)
Source: eLife. 2022 May 10;11:e73913. doi: 10.7554/eLife.73913 (PMC9090334; doi:10.7554/eLife.73913)

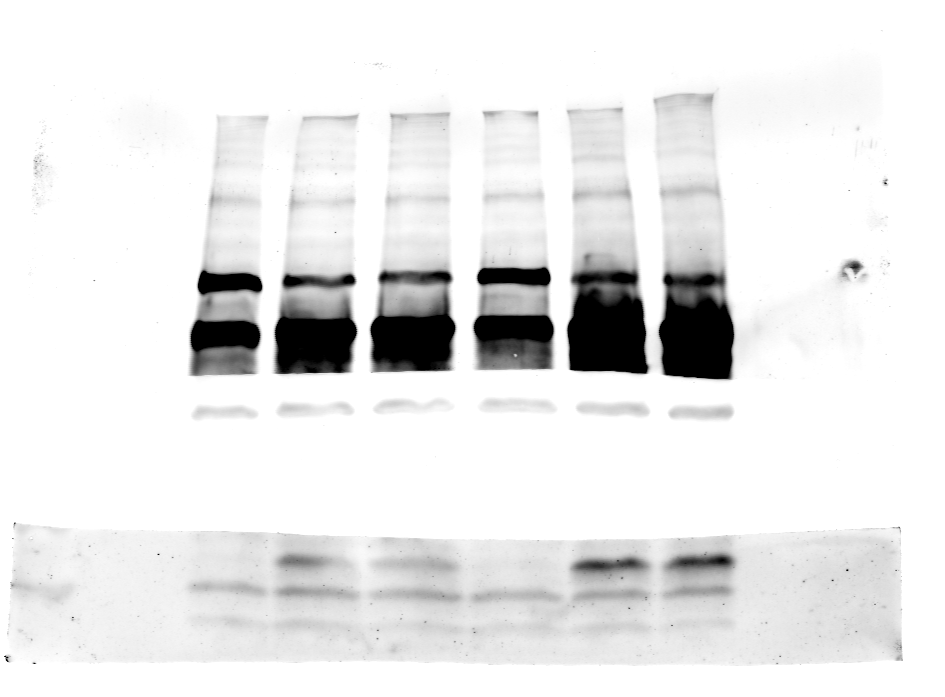

Supplement: Figure 4—figure supplement 1—source data 1. — The tiff files correspond to uncropped pictures of the IRDye800CW fluorescence signal acquired on a LI-COR Odyssey. The regions used to generate the figure are highlighted by back squares in the jpg file. [file elife-73913-fig4-figsupp1-data1.zip › Figure 4-figure supplement 1-source data 1/Fig.4-S1C-p21.tif]

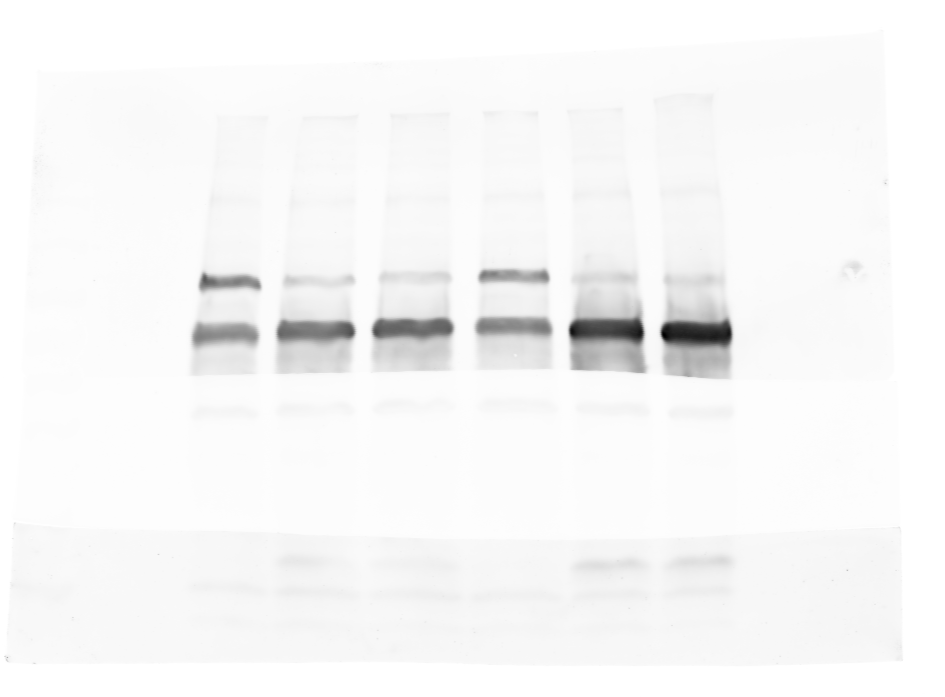

Supplement: Figure 4—figure supplement 1—source data 1. — The tiff files correspond to uncropped pictures of the IRDye800CW fluorescence signal acquired on a LI-COR Odyssey. The regions used to generate the figure are highlighted by back squares in the jpg file. [file elife-73913-fig4-figsupp1-data1.zip › Figure 4-figure supplement 1-source data 1/Fig.4-S1C-PSMD2-HSP70.tif]

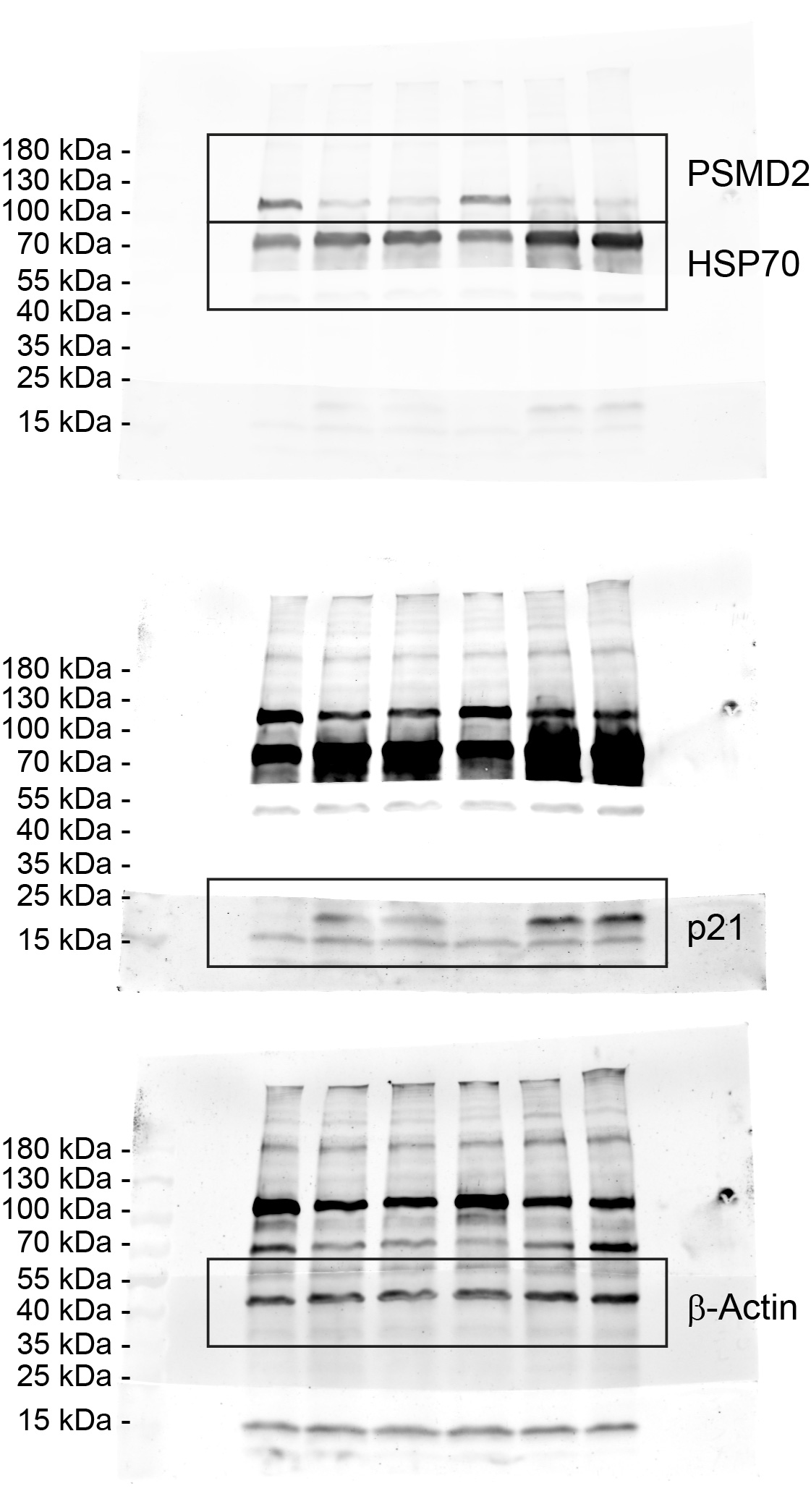

Supplement: Figure 4—figure supplement 1—source data 1. — The tiff files correspond to uncropped pictures of the IRDye800CW fluorescence signal acquired on a LI-COR Odyssey. The regions used to generate the figure are highlighted by back squares in the jpg file. [file elife-73913-fig4-figsupp1-data1.zip › Figure 4-figure supplement 1-source data 1/Fig.4-S1C.jpg]

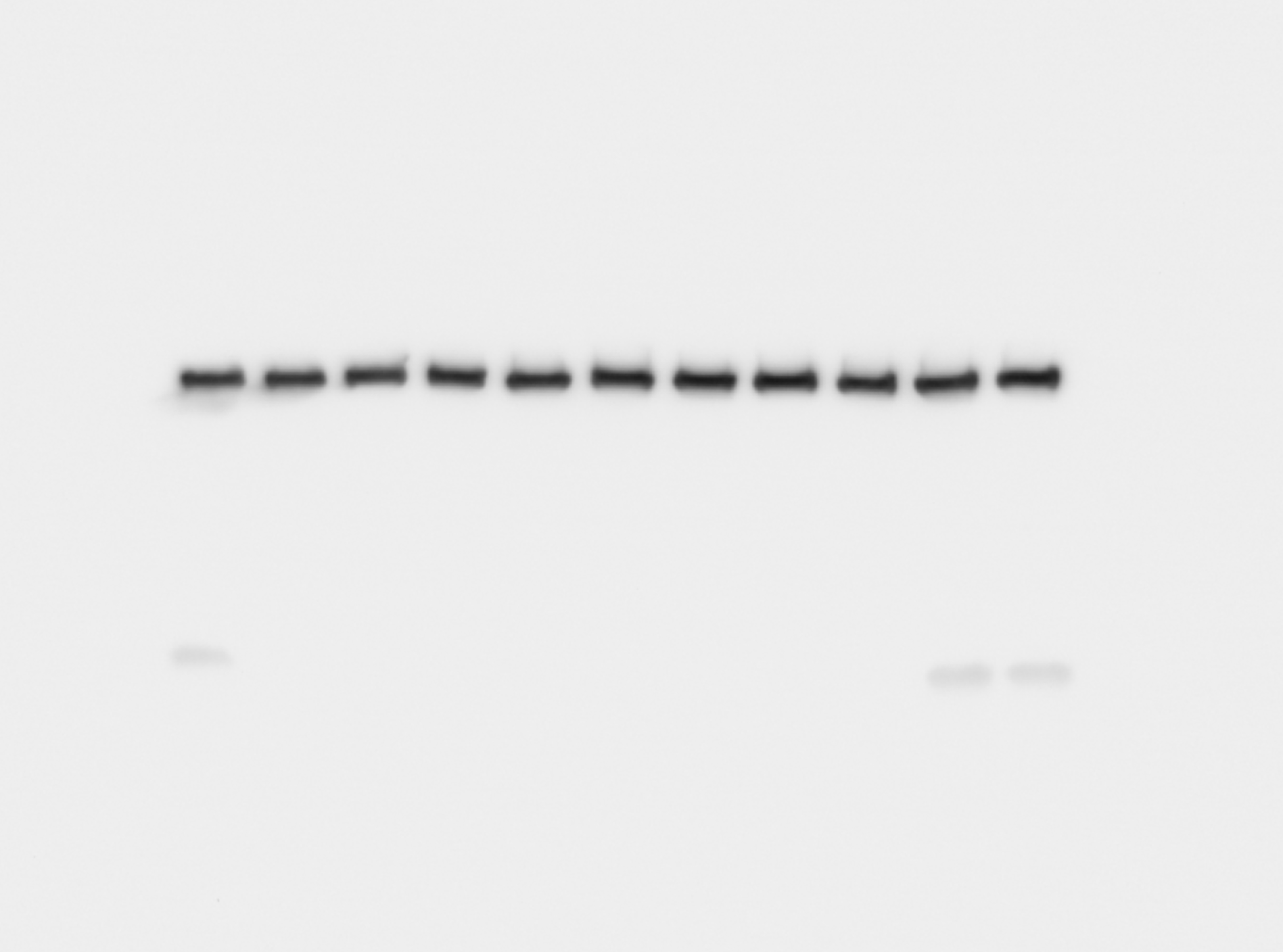

Supplement: Figure 5—source data 1. — The tiff files correspond to uncropped pictures of the chemiluminescent signal acquired on a BioRad Chemidoc. The regions used to generate the figure are highlighted by back squares in the jpg file, which also contains at the bottom an overlay with a picture of the membrane to locate the protein ladder positions. [file elife-73913-fig5-data1.zip › Figure 5-source data 1/Fig.5E-Ku80.tif]

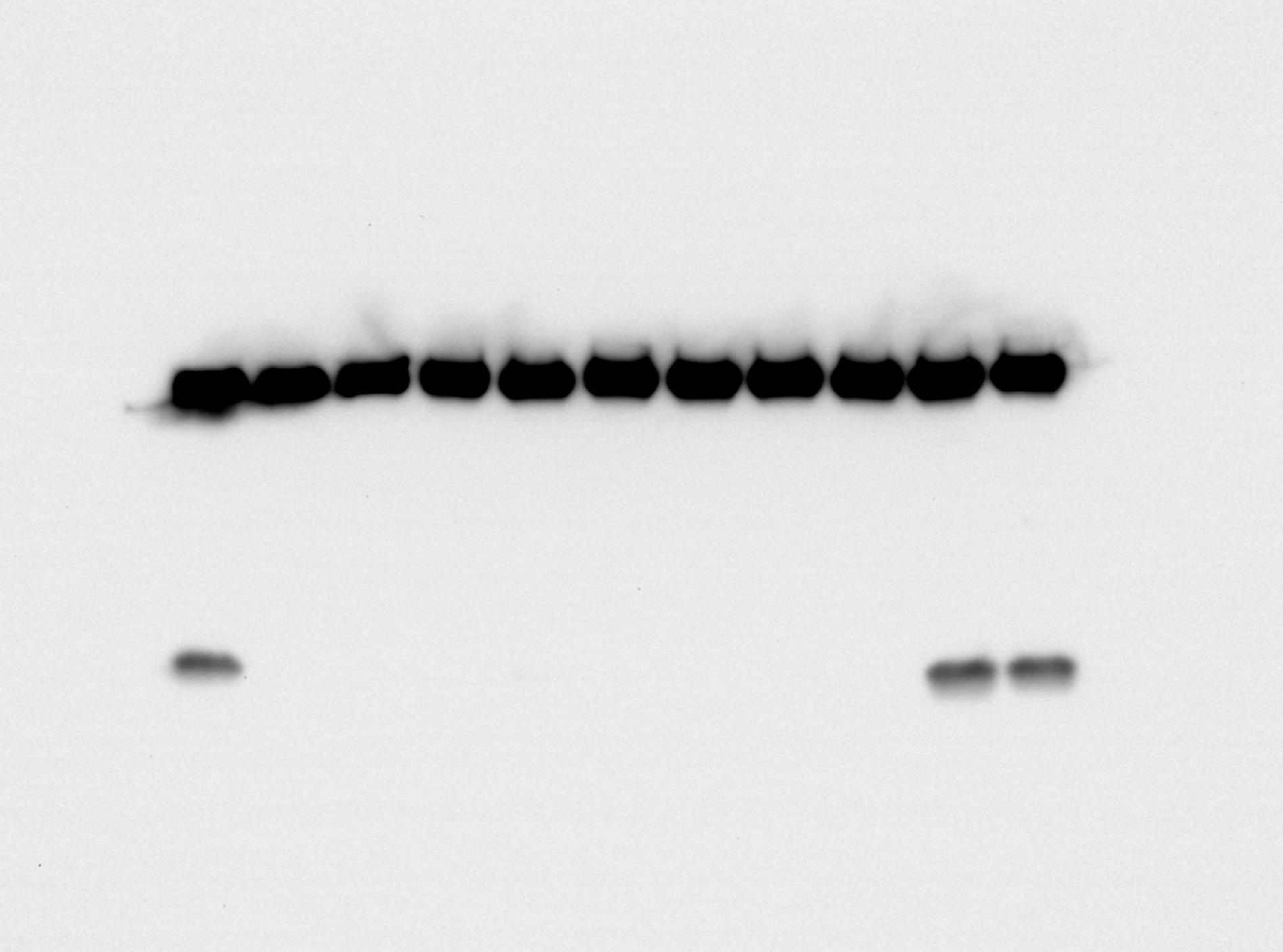

Supplement: Figure 5—source data 1. — The tiff files correspond to uncropped pictures of the chemiluminescent signal acquired on a BioRad Chemidoc. The regions used to generate the figure are highlighted by back squares in the jpg file, which also contains at the bottom an overlay with a picture of the membrane to locate the protein ladder positions. [file elife-73913-fig5-data1.zip › Figure 5-source data 1/Fig.5E-RDH11.tif]

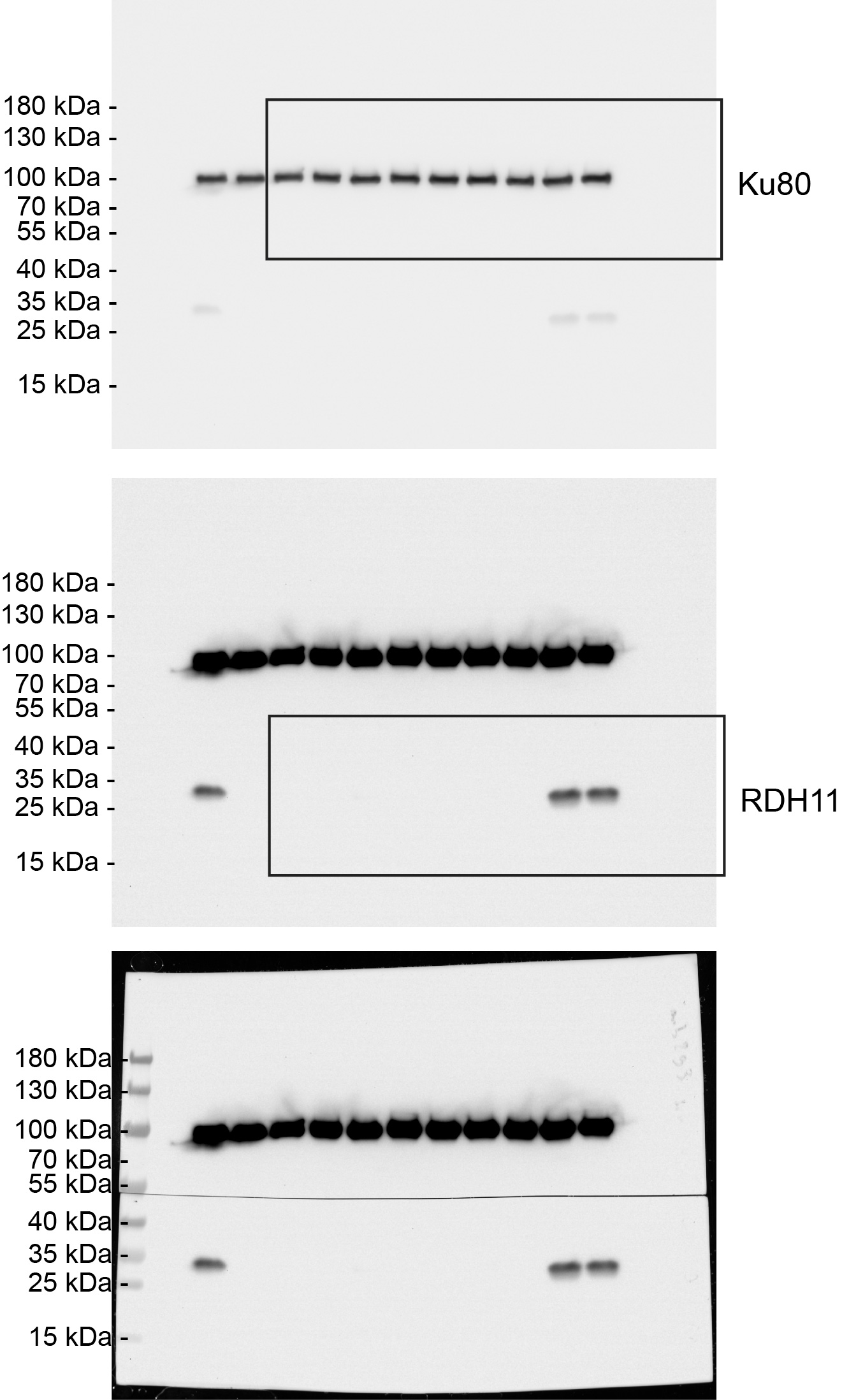

Supplement: Figure 5—source data 1. — The tiff files correspond to uncropped pictures of the chemiluminescent signal acquired on a BioRad Chemidoc. The regions used to generate the figure are highlighted by back squares in the jpg file, which also contains at the bottom an overlay with a picture of the membrane to locate the protein ladder positions. [file elife-73913-fig5-data1.zip › Figure 5-source data 1/Fig.5E.jpg]

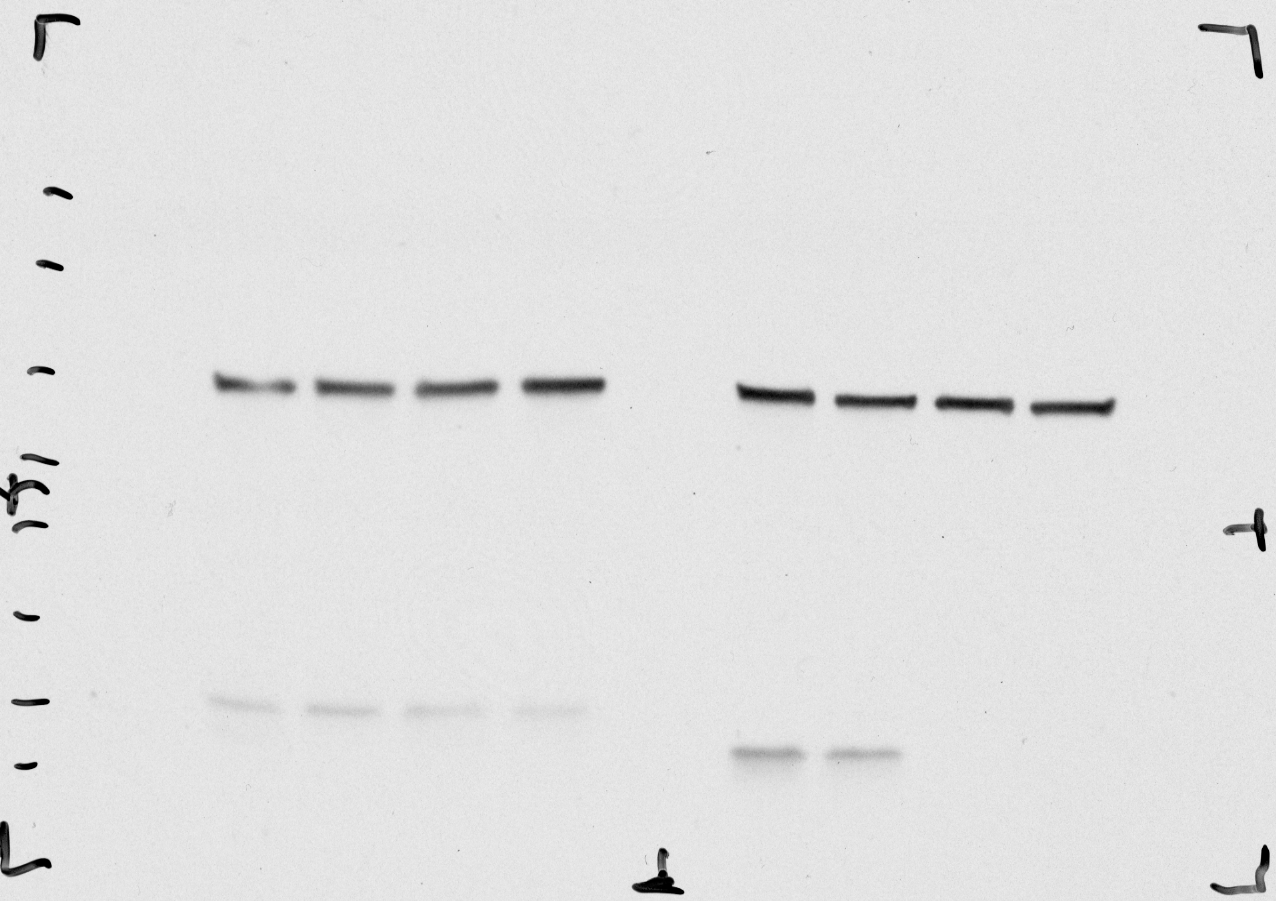

Supplement: Figure 5—source data 2. — The tiff files correspond to uncropped pictures of the chemiluminescent signal acquired using autoradiographic films. Two different immunoblotting of the same extracts were used for this figure (respectively labeled upper and lower). The regions used to generate the figure are highlighted for each immunoblot by back squares in the jpg files. [file elife-73913-fig5-data2.zip › Figure 5-source data 2/Fig.5F-Lower-Ku80.tif]

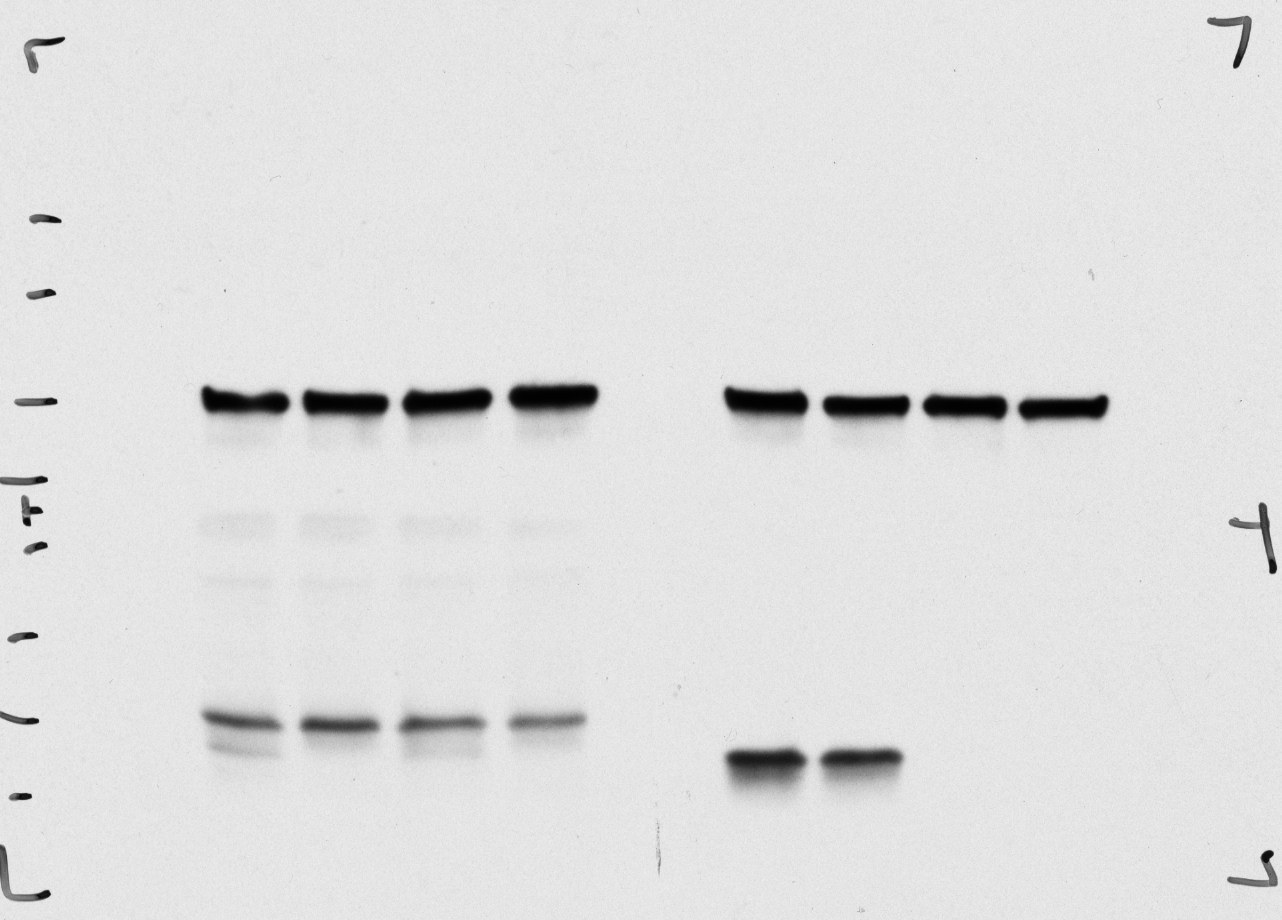

Supplement: Figure 5—source data 2. — The tiff files correspond to uncropped pictures of the chemiluminescent signal acquired using autoradiographic films. Two different immunoblotting of the same extracts were used for this figure (respectively labeled upper and lower). The regions used to generate the figure are highlighted for each immunoblot by back squares in the jpg files. [file elife-73913-fig5-data2.zip › Figure 5-source data 2/Fig.5F-Lower-RDH11.tif]

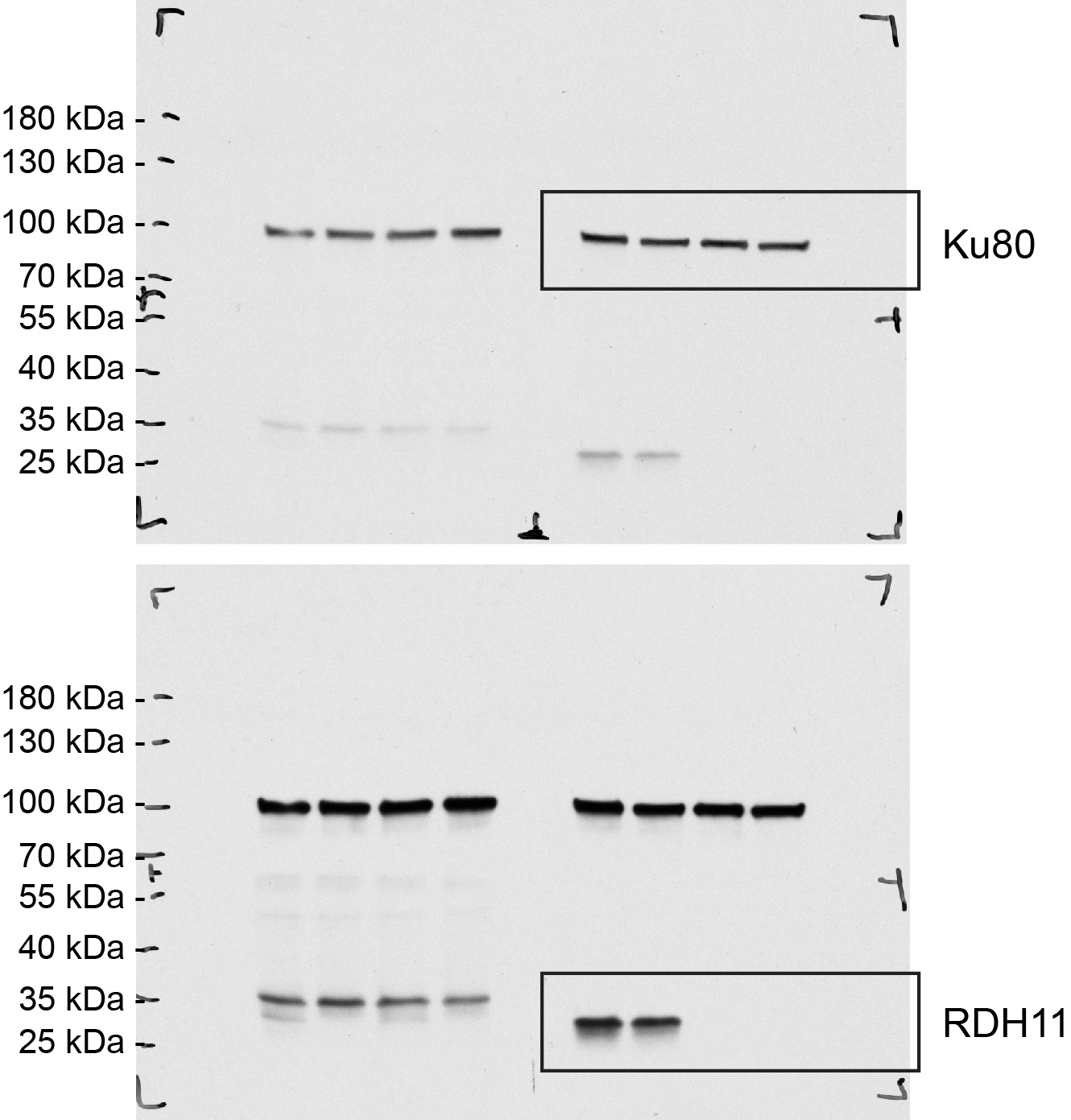

Supplement: Figure 5—source data 2. — The tiff files correspond to uncropped pictures of the chemiluminescent signal acquired using autoradiographic films. Two different immunoblotting of the same extracts were used for this figure (respectively labeled upper and lower). The regions used to generate the figure are highlighted for each immunoblot by back squares in the jpg files. [file elife-73913-fig5-data2.zip › Figure 5-source data 2/Fig.5F-Lower.jpg]

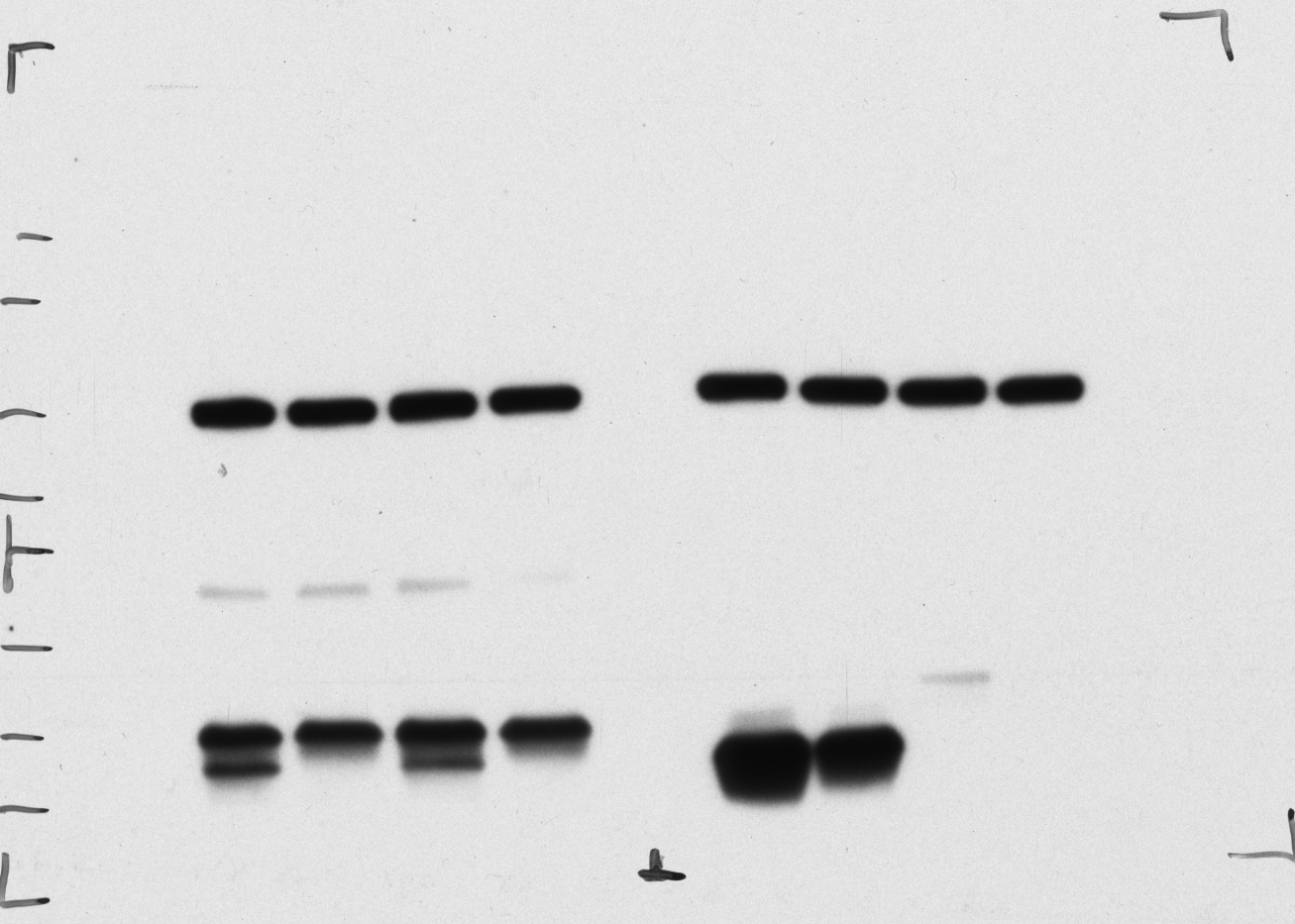

Supplement: Figure 5—source data 2. — The tiff files correspond to uncropped pictures of the chemiluminescent signal acquired using autoradiographic films. Two different immunoblotting of the same extracts were used for this figure (respectively labeled upper and lower). The regions used to generate the figure are highlighted for each immunoblot by back squares in the jpg files. [file elife-73913-fig5-data2.zip › Figure 5-source data 2/Fig.5F-Upper-HSD17B11.tif]

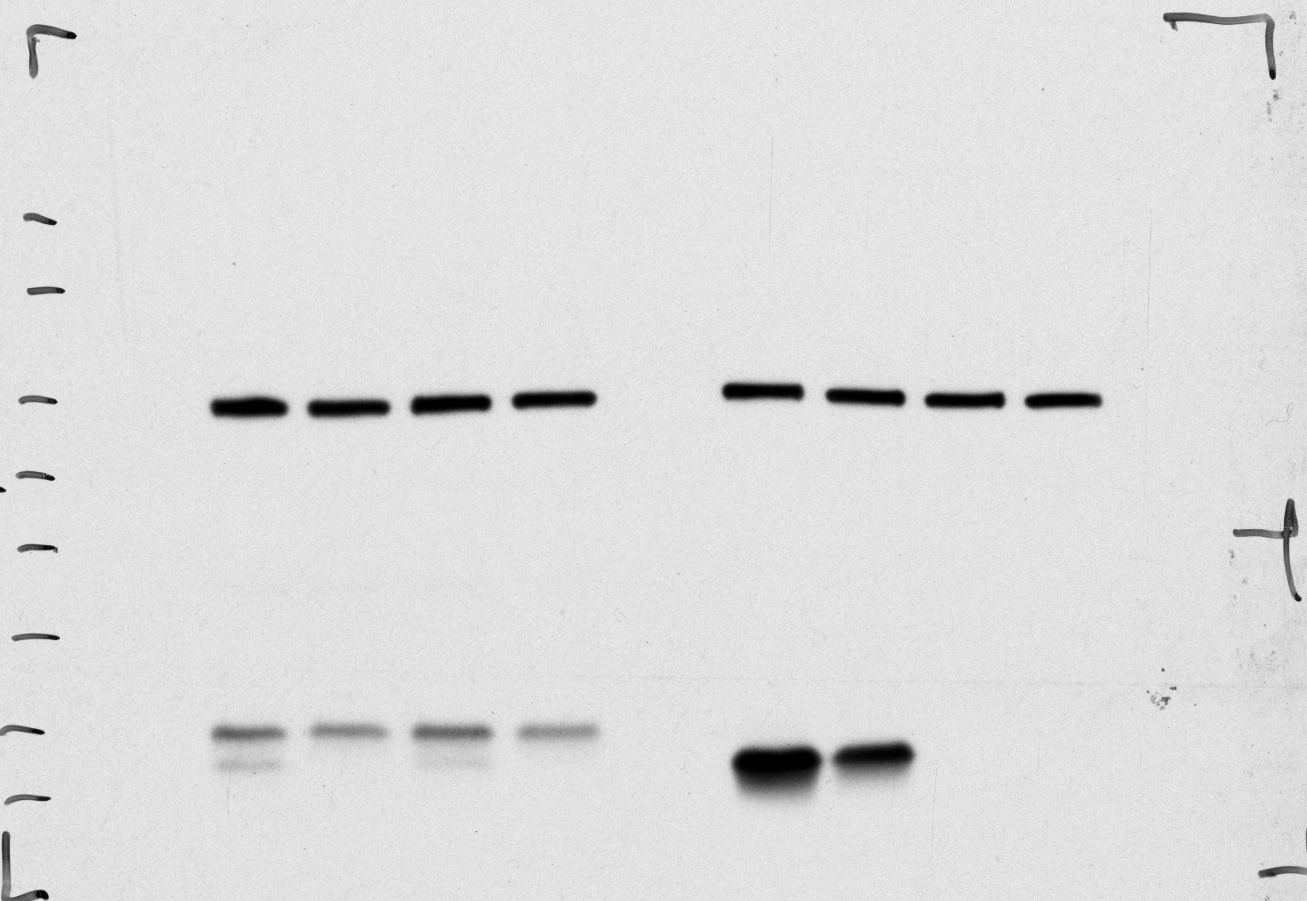

Supplement: Figure 5—source data 2. — The tiff files correspond to uncropped pictures of the chemiluminescent signal acquired using autoradiographic films. Two different immunoblotting of the same extracts were used for this figure (respectively labeled upper and lower). The regions used to generate the figure are highlighted for each immunoblot by back squares in the jpg files. [file elife-73913-fig5-data2.zip › Figure 5-source data 2/Fig.5F-Upper-Ku80.tif]

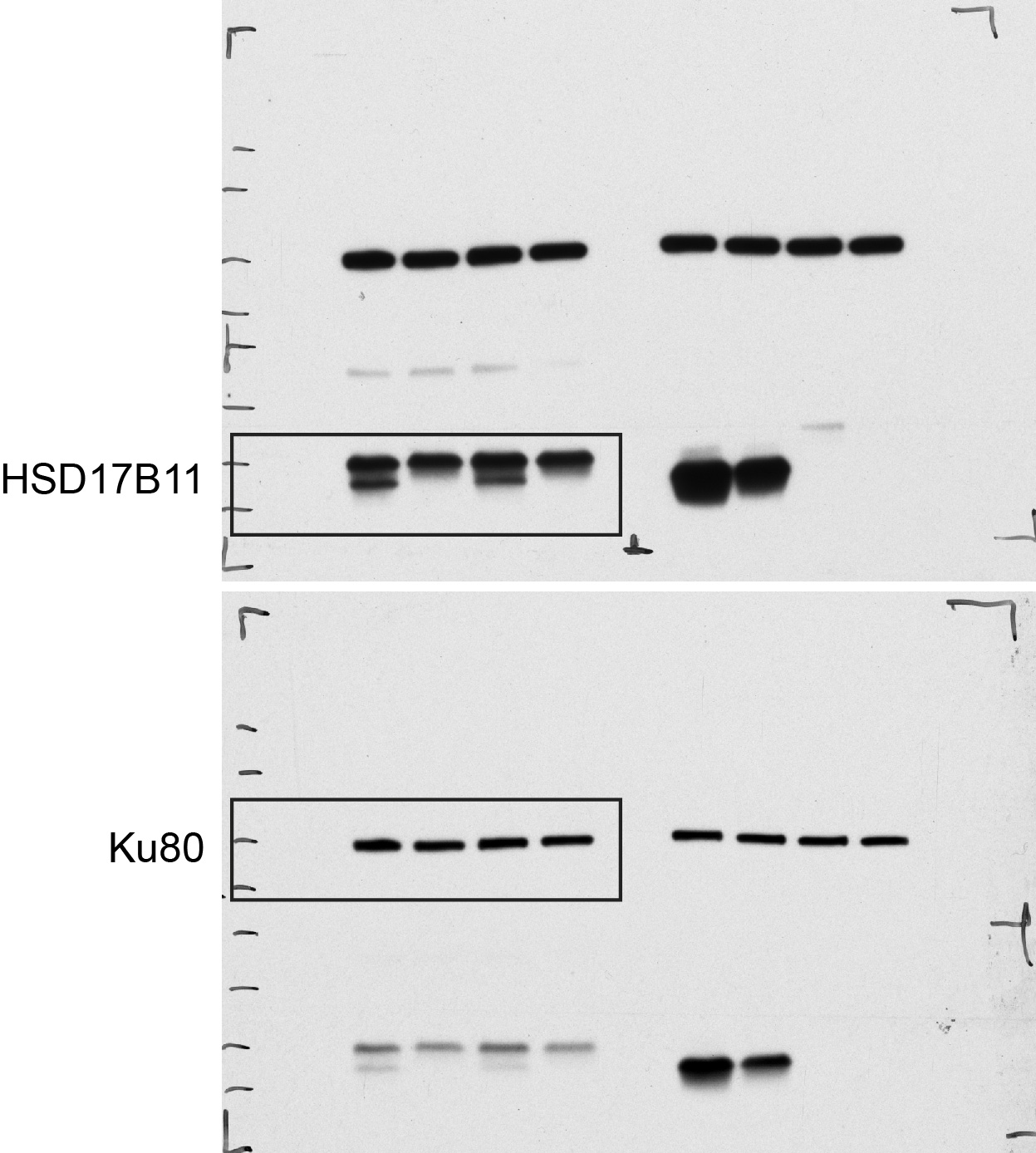

Supplement: Figure 5—source data 2. — The tiff files correspond to uncropped pictures of the chemiluminescent signal acquired using autoradiographic films. Two different immunoblotting of the same extracts were used for this figure (respectively labeled upper and lower). The regions used to generate the figure are highlighted for each immunoblot by back squares in the jpg files. [file elife-73913-fig5-data2.zip › Figure 5-source data 2/Fig.5F-Upper.jpg]

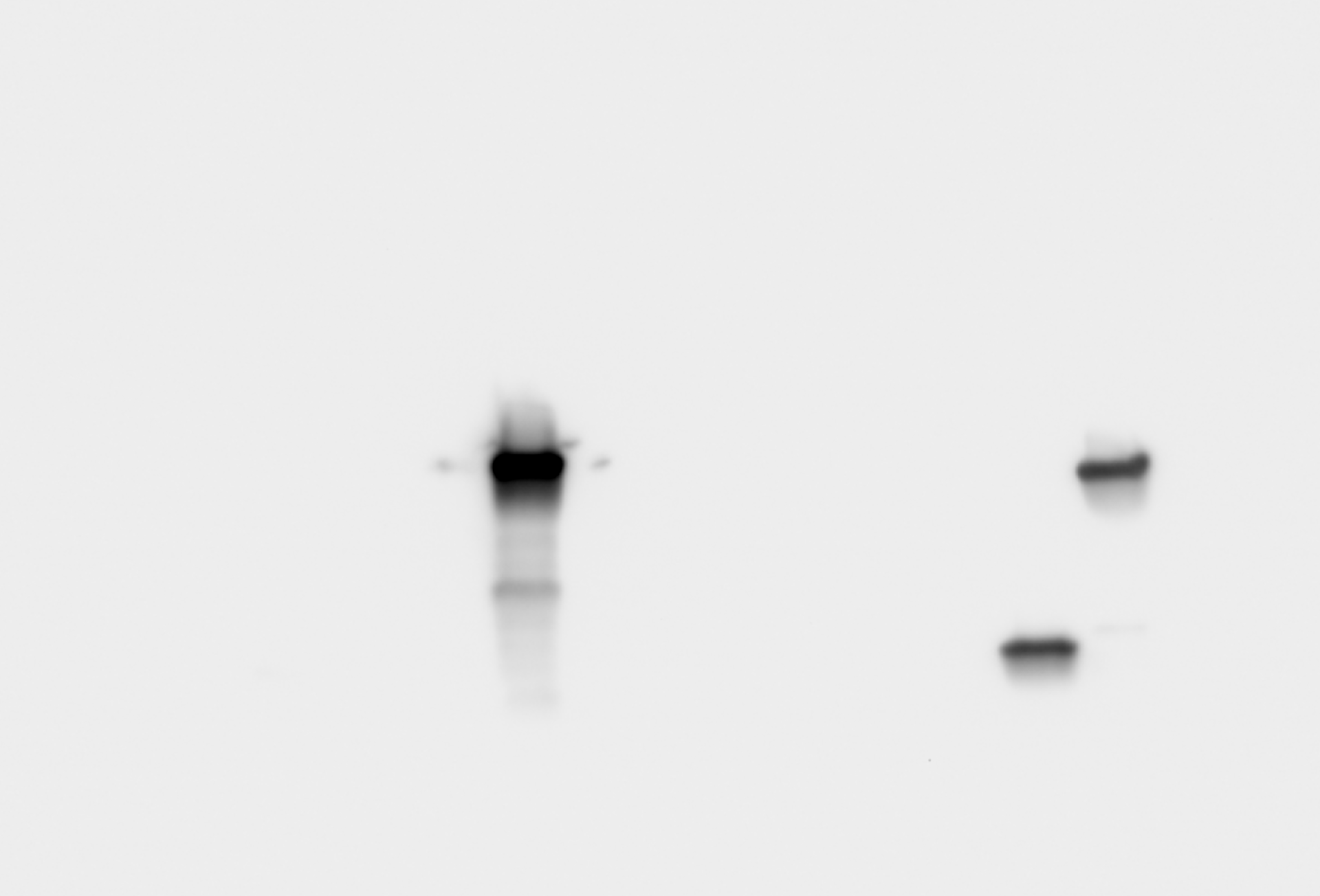

Supplement: Figure 5—source data 3. — The tiff files correspond to uncropped pictures of the chemiluminescent signal acquired on a BioRad Chemidoc. The same extracts were loaded twice (left and right part of the membrane) and the membrane sliced to simultaneously probe GFP and HPGD. The regions used to generate the figure are highlighted by back squares in the jpg file, which also contains overlays of the chemiluminescent signal with a picture of the membrane to locate the protein ladder positions. [file elife-73913-fig5-data3.zip › Figure 5-source data 3/Fig.5I-GFP-GFP.tif]

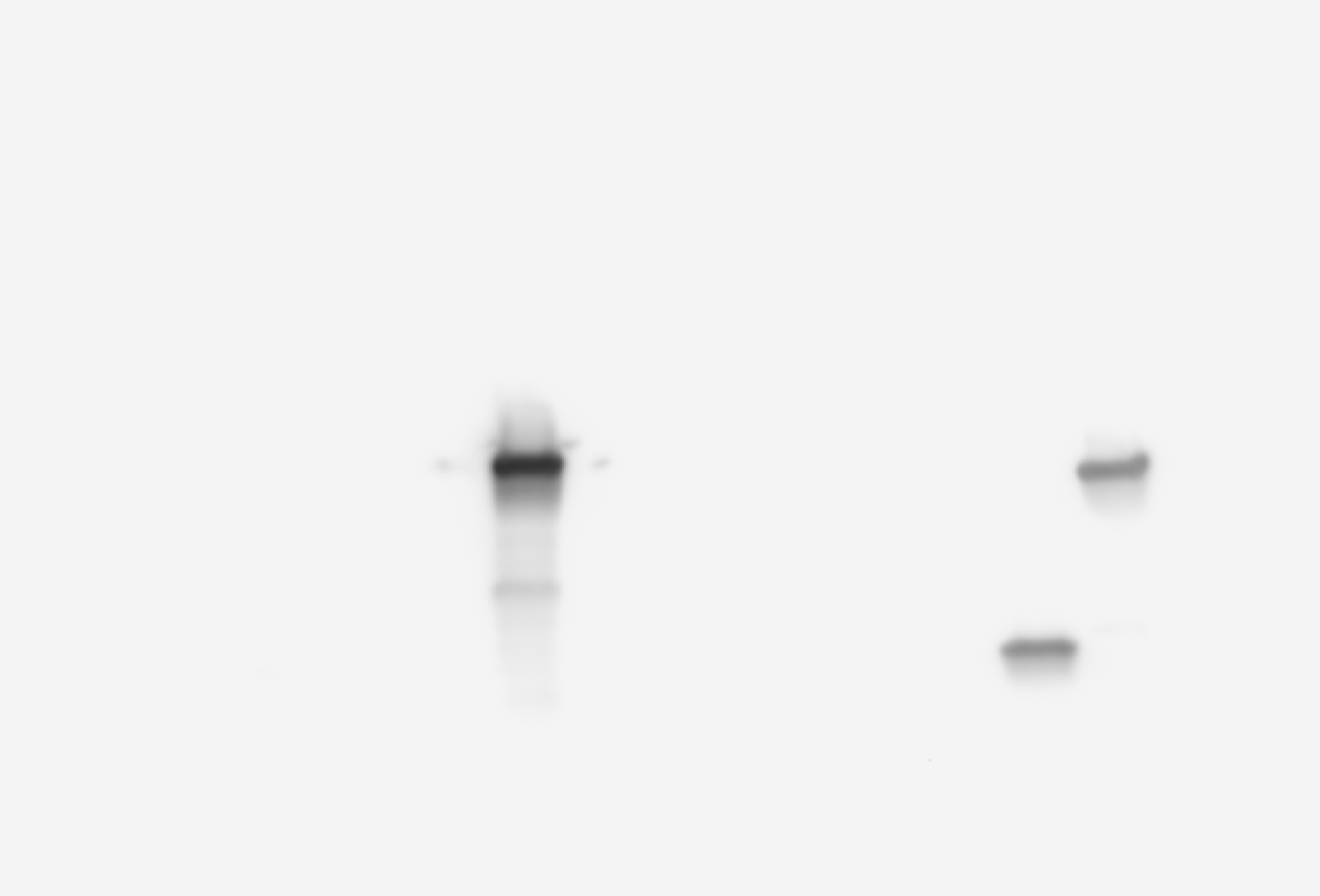

Supplement: Figure 5—source data 3. — The tiff files correspond to uncropped pictures of the chemiluminescent signal acquired on a BioRad Chemidoc. The same extracts were loaded twice (left and right part of the membrane) and the membrane sliced to simultaneously probe GFP and HPGD. The regions used to generate the figure are highlighted by back squares in the jpg file, which also contains overlays of the chemiluminescent signal with a picture of the membrane to locate the protein ladder positions. [file elife-73913-fig5-data3.zip › Figure 5-source data 3/Fig.5I-GFP-HPGD.tif]

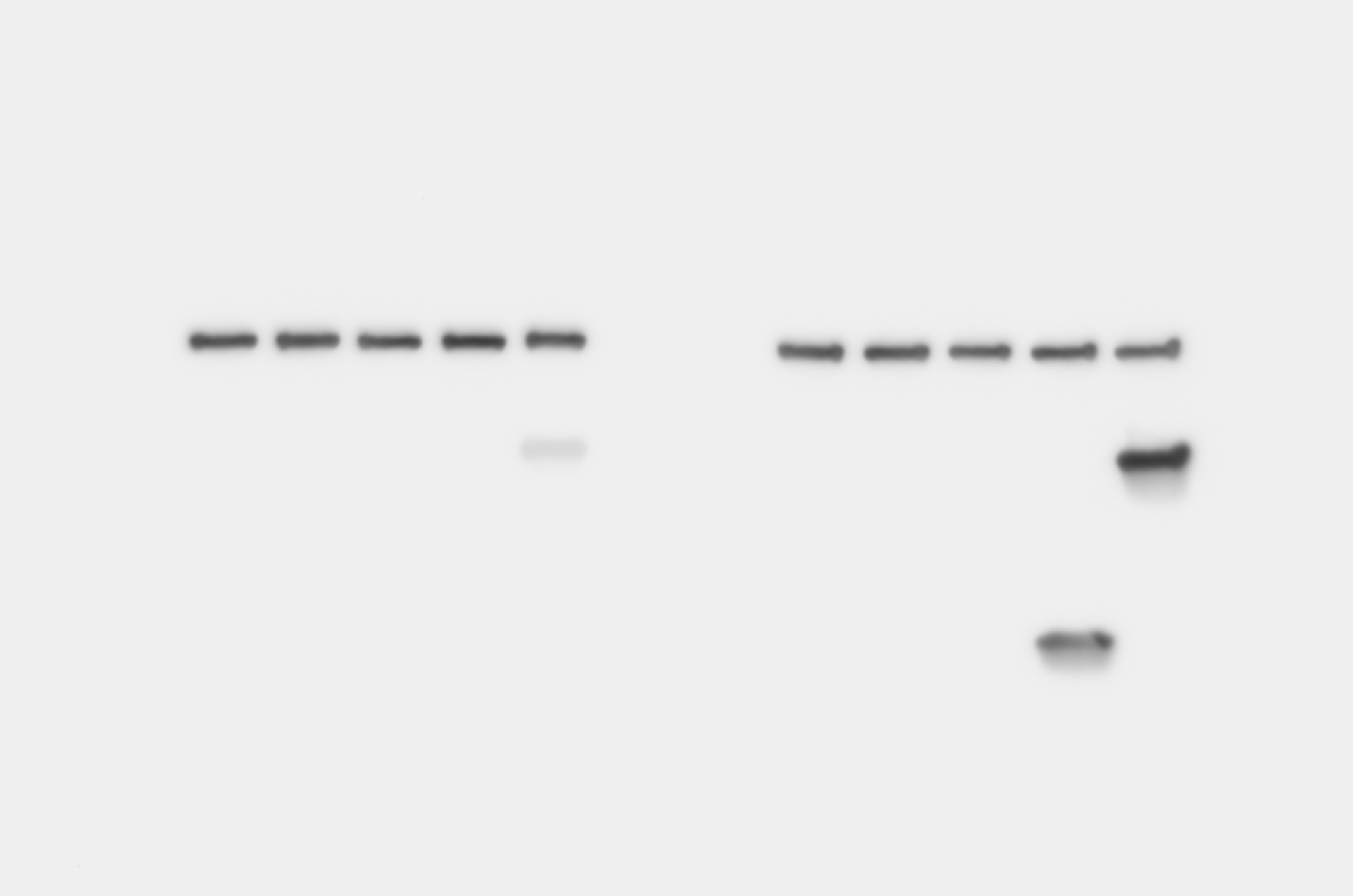

Supplement: Figure 5—source data 3. — The tiff files correspond to uncropped pictures of the chemiluminescent signal acquired on a BioRad Chemidoc. The same extracts were loaded twice (left and right part of the membrane) and the membrane sliced to simultaneously probe GFP and HPGD. The regions used to generate the figure are highlighted by back squares in the jpg file, which also contains overlays of the chemiluminescent signal with a picture of the membrane to locate the protein ladder positions. [file elife-73913-fig5-data3.zip › Figure 5-source data 3/Fig.5I-Ku80.tif]

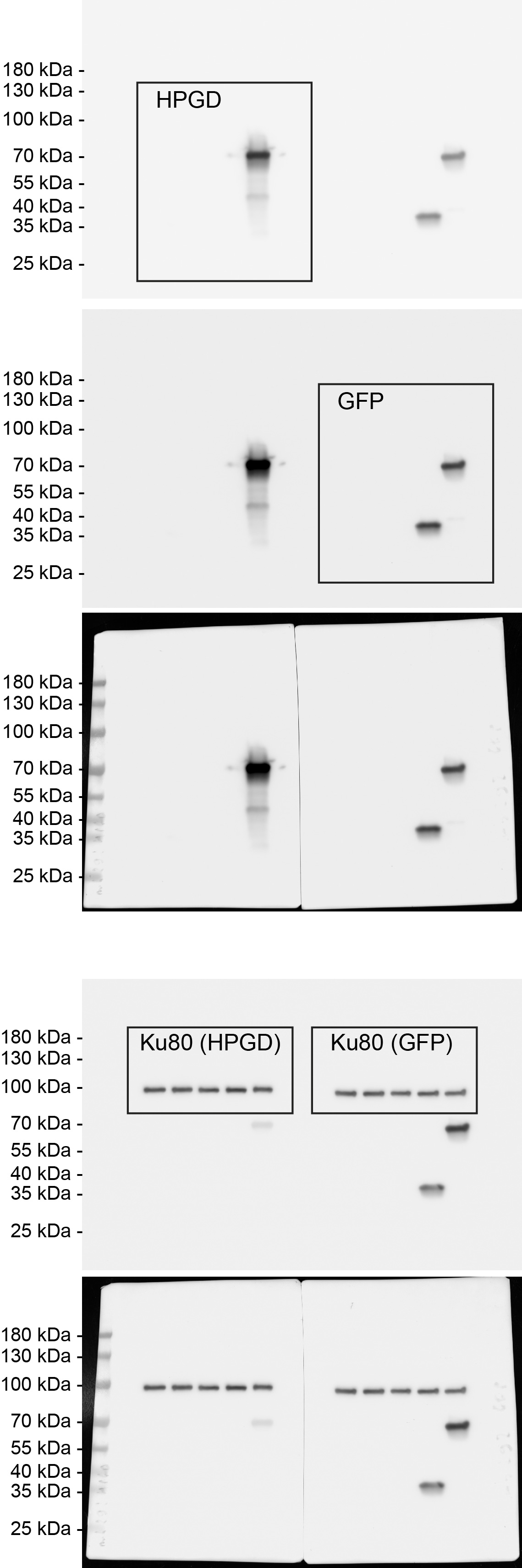

Supplement: Figure 5—source data 3. — The tiff files correspond to uncropped pictures of the chemiluminescent signal acquired on a BioRad Chemidoc. The same extracts were loaded twice (left and right part of the membrane) and the membrane sliced to simultaneously probe GFP and HPGD. The regions used to generate the figure are highlighted by back squares in the jpg file, which also contains overlays of the chemiluminescent signal with a picture of the membrane to locate the protein ladder positions. [file elife-73913-fig5-data3.zip › Figure 5-source data 3/Fig.5I.jpg]

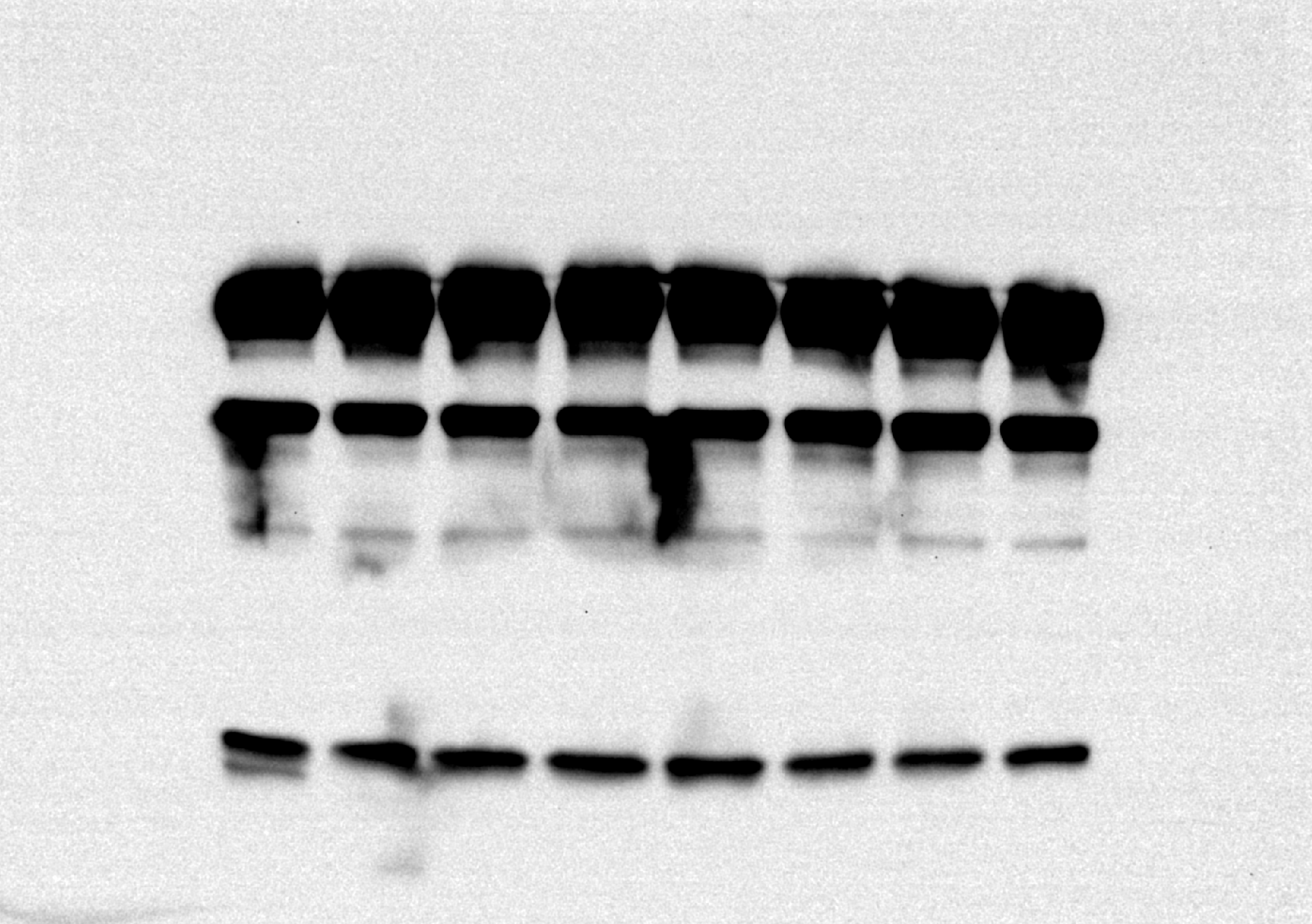

Supplement: Figure 5—figure supplement 2—source data 1. — The tiff files correspond to uncropped pictures of the chemiluminescent signal acquired on a BioRad Chemidoc. Two different immunoblotting of the same extracts were used for this figure (respectively labeled upper and lower). The regions used to generate are highlighted for each immunoblot by back squares in the jpg files, which also contain at the bottom an overlay with a picture of the membrane to locate the protein ladder positions. [file elife-73913-fig5-figsupp2-data1.zip › Figure 5-figure supplement 2-source data 1/Fig.5-S2A-Lower-HSD17B11.tif]

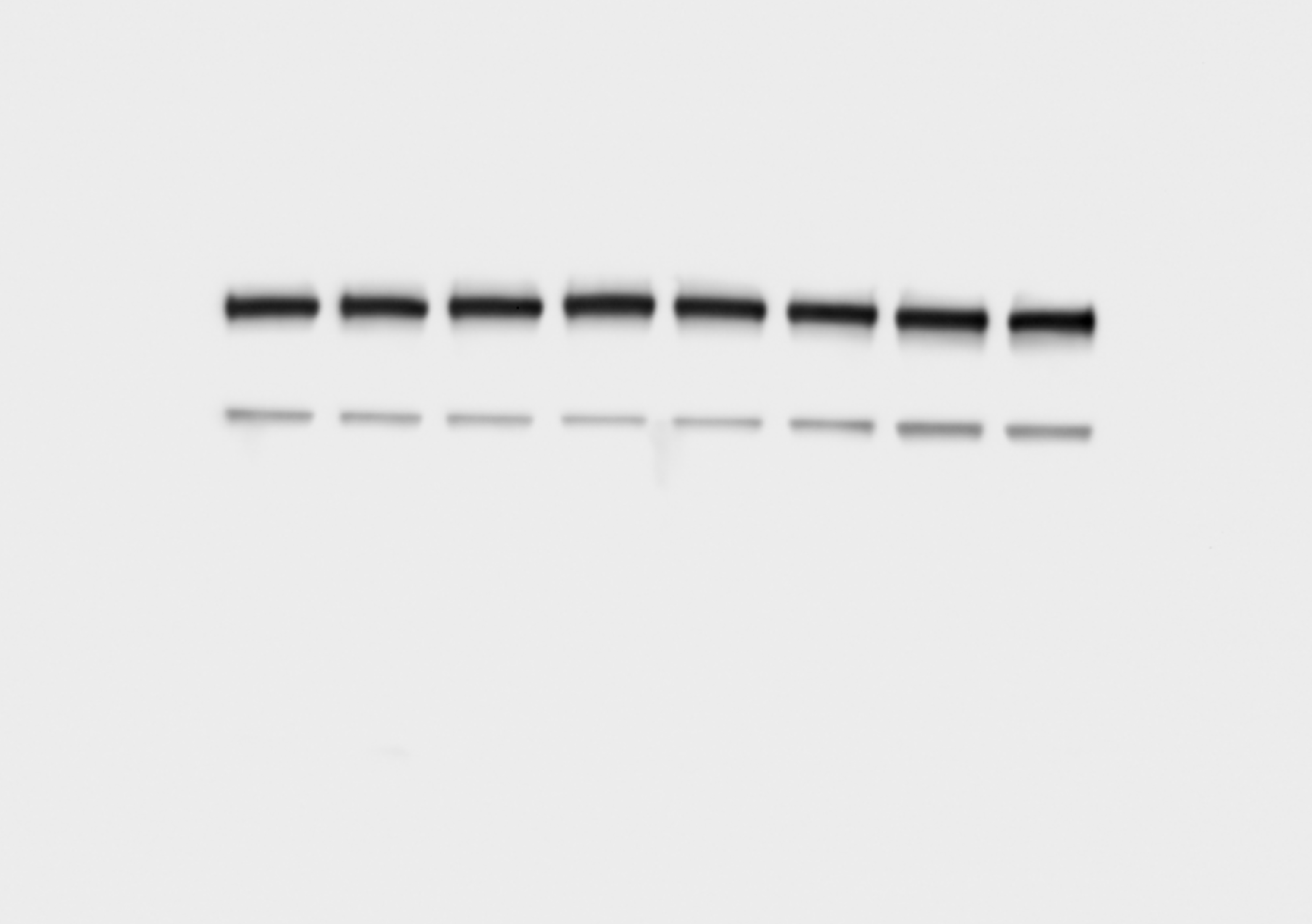

Supplement: Figure 5—figure supplement 2—source data 1. — The tiff files correspond to uncropped pictures of the chemiluminescent signal acquired on a BioRad Chemidoc. Two different immunoblotting of the same extracts were used for this figure (respectively labeled upper and lower). The regions used to generate are highlighted for each immunoblot by back squares in the jpg files, which also contain at the bottom an overlay with a picture of the membrane to locate the protein ladder positions. [file elife-73913-fig5-figsupp2-data1.zip › Figure 5-figure supplement 2-source data 1/Fig.5-S2A-Lower-SAFA.tif]

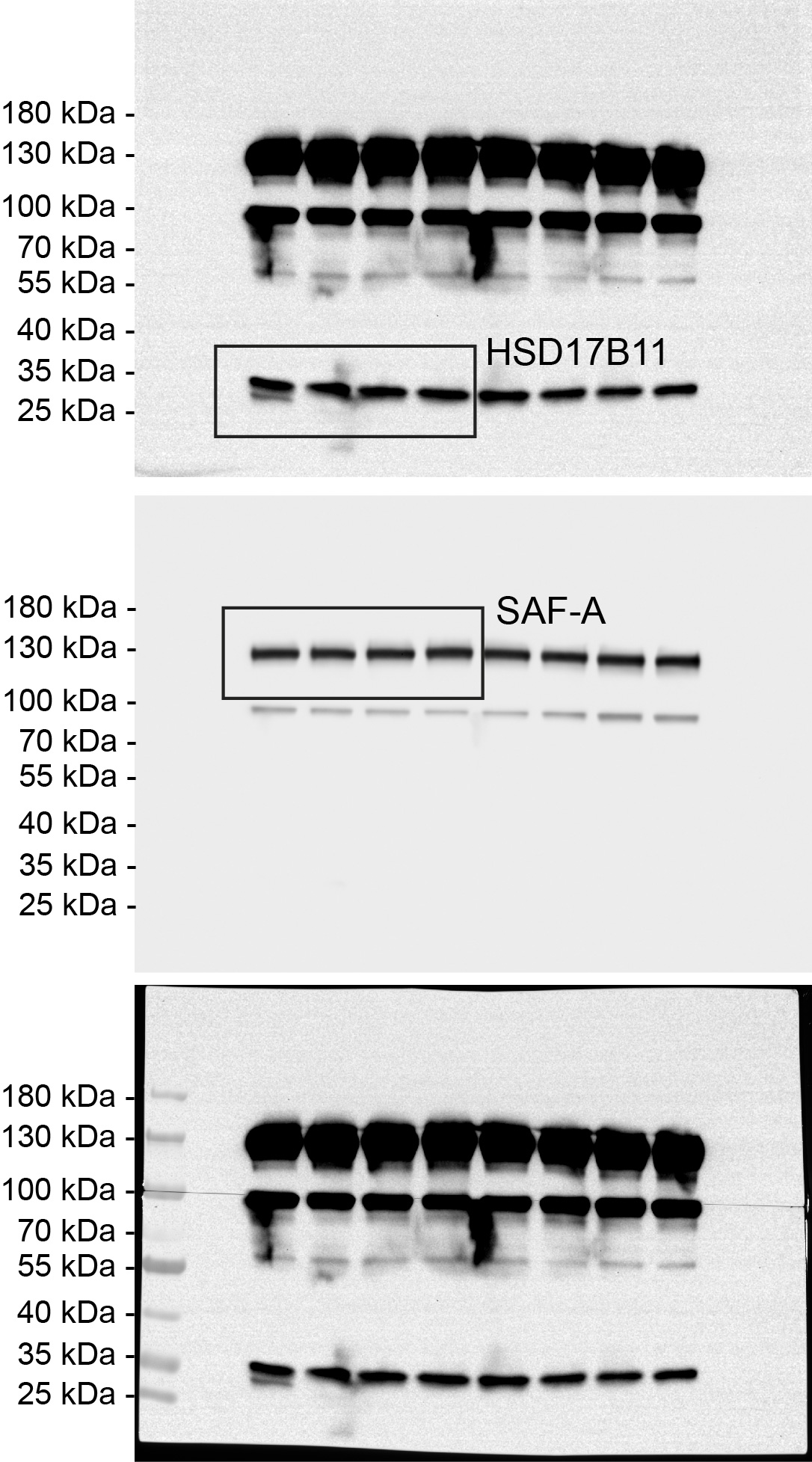

Supplement: Figure 5—figure supplement 2—source data 1. — The tiff files correspond to uncropped pictures of the chemiluminescent signal acquired on a BioRad Chemidoc. Two different immunoblotting of the same extracts were used for this figure (respectively labeled upper and lower). The regions used to generate are highlighted for each immunoblot by back squares in the jpg files, which also contain at the bottom an overlay with a picture of the membrane to locate the protein ladder positions. [file elife-73913-fig5-figsupp2-data1.zip › Figure 5-figure supplement 2-source data 1/Fig.5-S2A-Lower.jpg]

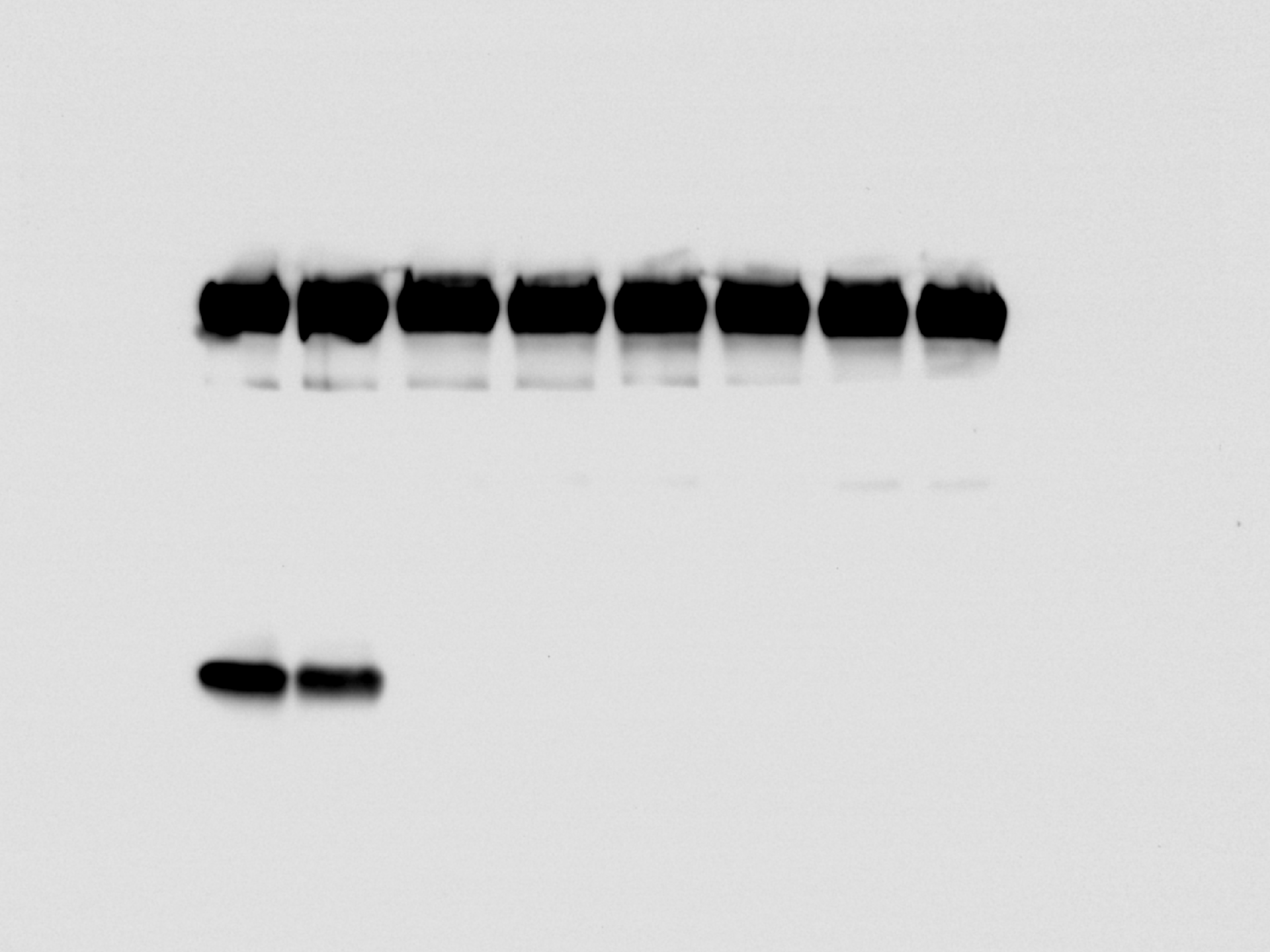

Supplement: Figure 5—figure supplement 2—source data 1. — The tiff files correspond to uncropped pictures of the chemiluminescent signal acquired on a BioRad Chemidoc. Two different immunoblotting of the same extracts were used for this figure (respectively labeled upper and lower). The regions used to generate are highlighted for each immunoblot by back squares in the jpg files, which also contain at the bottom an overlay with a picture of the membrane to locate the protein ladder positions. [file elife-73913-fig5-figsupp2-data1.zip › Figure 5-figure supplement 2-source data 1/Fig.5-S2A-Upper-RDH11.tif]

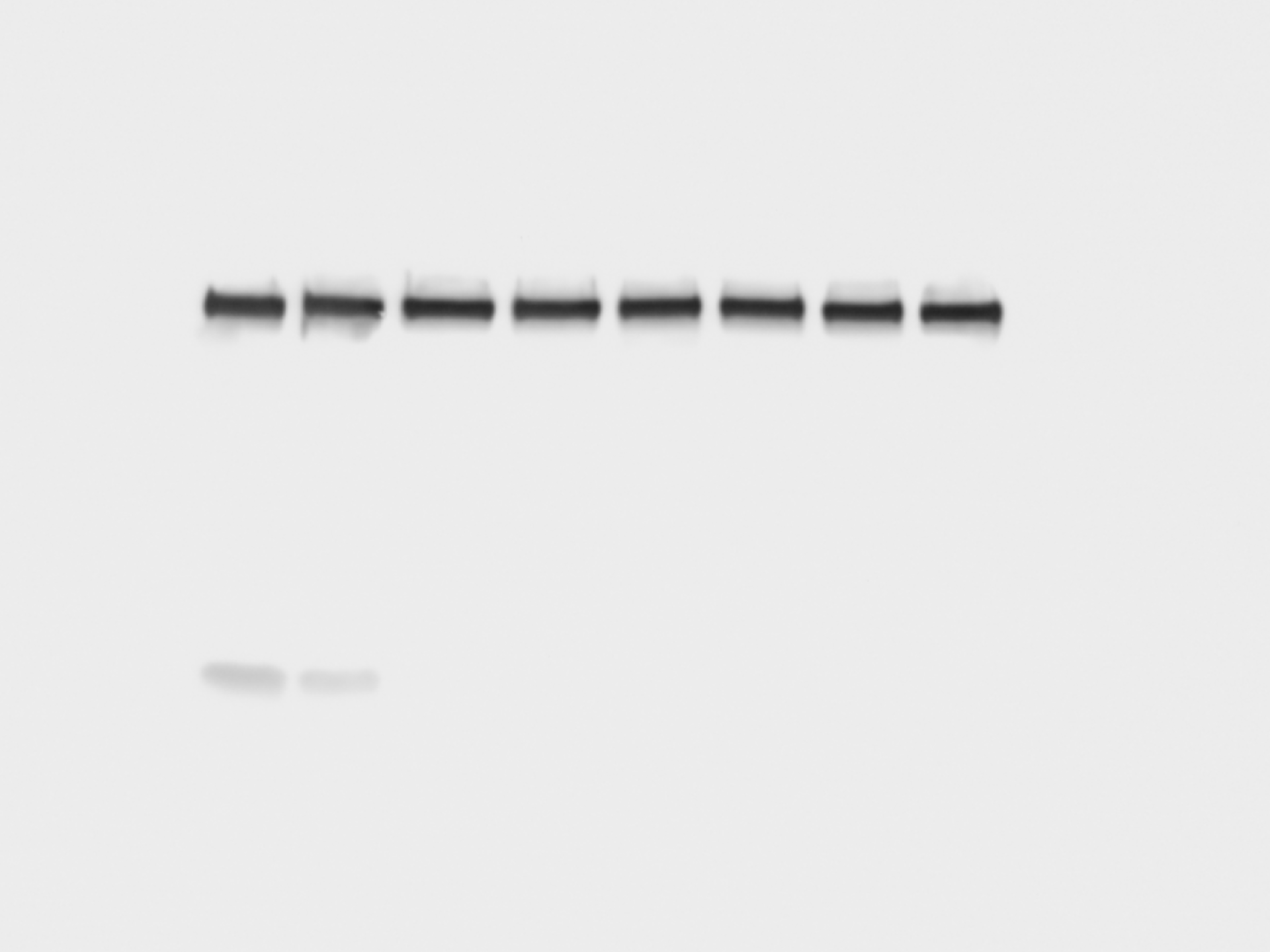

Supplement: Figure 5—figure supplement 2—source data 1. — The tiff files correspond to uncropped pictures of the chemiluminescent signal acquired on a BioRad Chemidoc. Two different immunoblotting of the same extracts were used for this figure (respectively labeled upper and lower). The regions used to generate are highlighted for each immunoblot by back squares in the jpg files, which also contain at the bottom an overlay with a picture of the membrane to locate the protein ladder positions. [file elife-73913-fig5-figsupp2-data1.zip › Figure 5-figure supplement 2-source data 1/Fig.5-S2A-Upper-SAFA.tif]

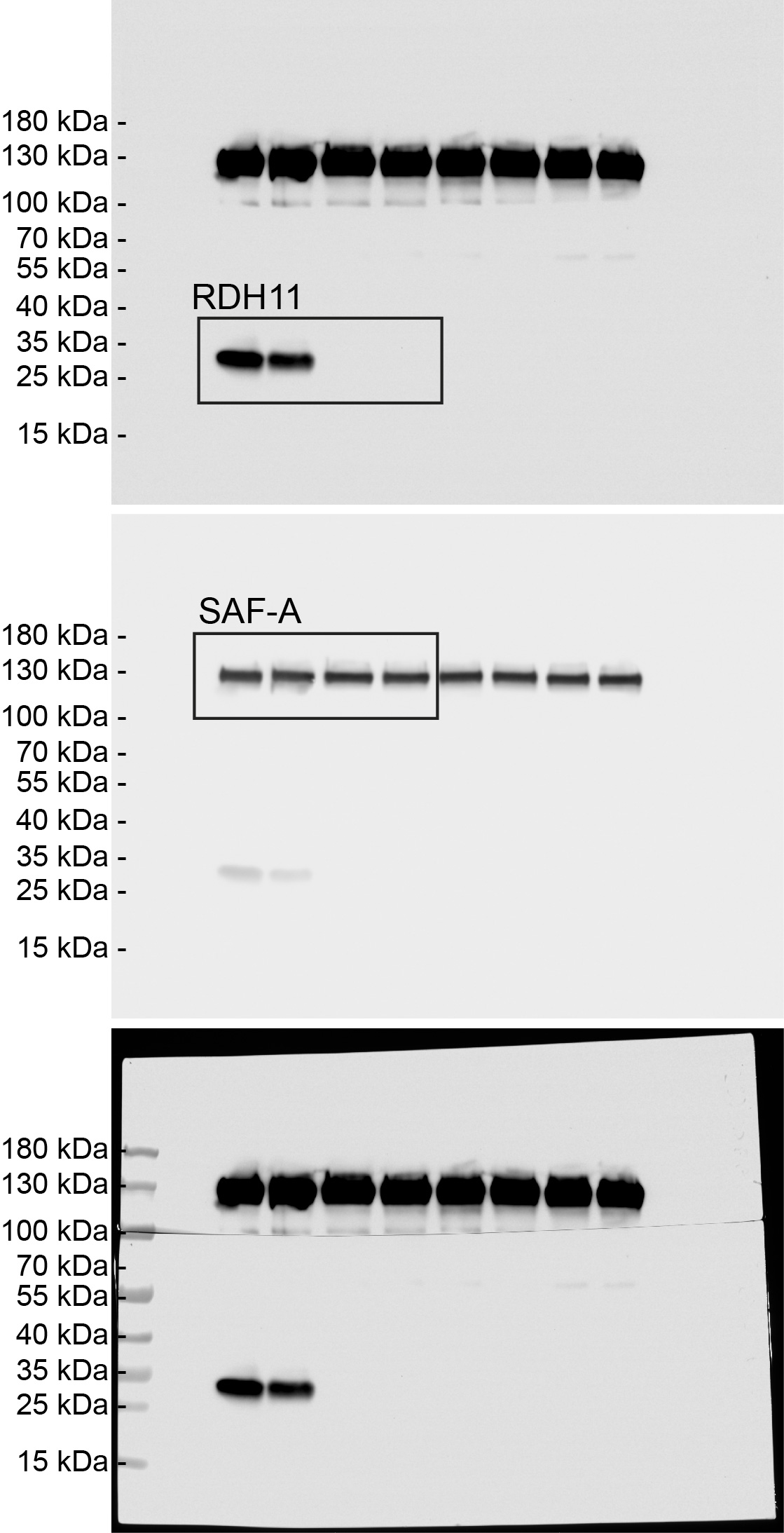

Supplement: Figure 5—figure supplement 2—source data 1. — The tiff files correspond to uncropped pictures of the chemiluminescent signal acquired on a BioRad Chemidoc. Two different immunoblotting of the same extracts were used for this figure (respectively labeled upper and lower). The regions used to generate are highlighted for each immunoblot by back squares in the jpg files, which also contain at the bottom an overlay with a picture of the membrane to locate the protein ladder positions. [file elife-73913-fig5-figsupp2-data1.zip › Figure 5-figure supplement 2-source data 1/Fig.5-S2A-Upper.jpg]

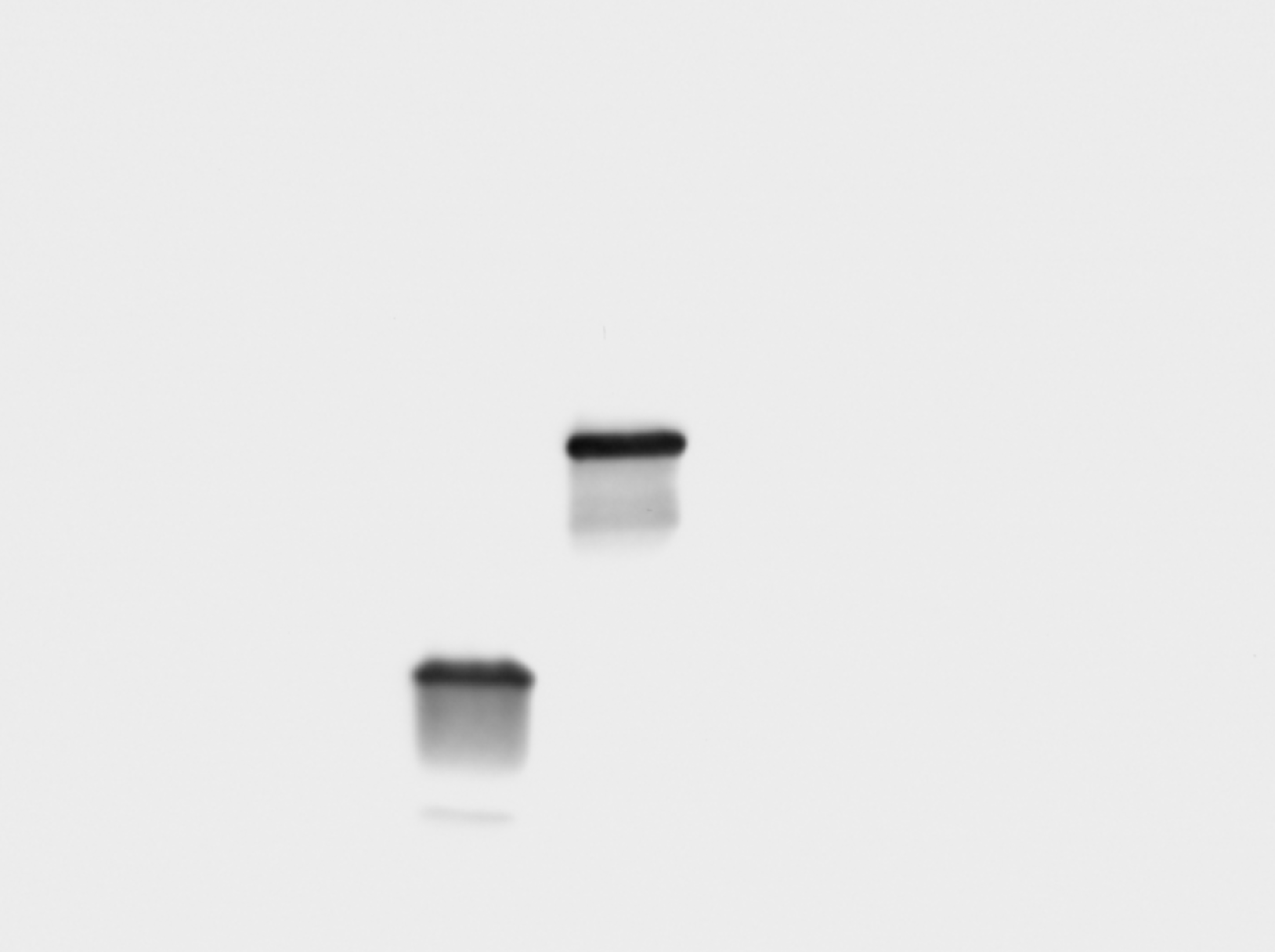

Supplement: Figure 5—figure supplement 2—source data 2. — The tiff files correspond to uncropped pictures of the chemiluminescent signal acquired on a BioRad Chemidoc. Two different immunoblotting of the same extracts were used for this figure (respectively labeled upper and lower). The regions used to generate the figure are highlighted for each immunoblot by back squares in the jpg files, which also contain at the bottom an overlay with a picture of the membrane to locate the protein ladder positions. [file elife-73913-fig5-figsupp2-data2.zip › Figure 5-figure supplement 2-source data 2/Fig.5-S2C-lower-GFP.tif]

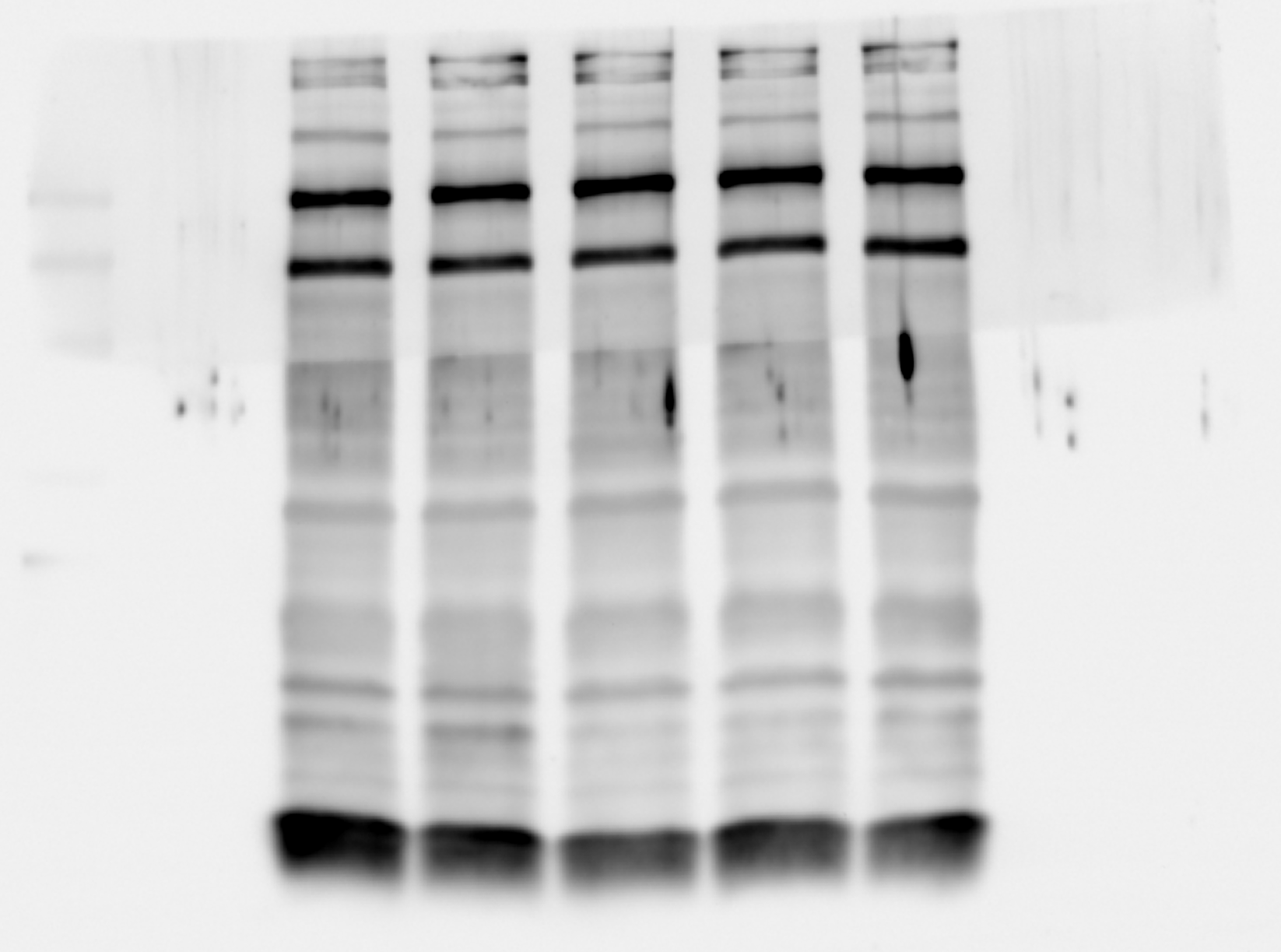

Supplement: Figure 5—figure supplement 2—source data 2. — The tiff files correspond to uncropped pictures of the chemiluminescent signal acquired on a BioRad Chemidoc. Two different immunoblotting of the same extracts were used for this figure (respectively labeled upper and lower). The regions used to generate the figure are highlighted for each immunoblot by back squares in the jpg files, which also contain at the bottom an overlay with a picture of the membrane to locate the protein ladder positions. [file elife-73913-fig5-figsupp2-data2.zip › Figure 5-figure supplement 2-source data 2/Fig.5-S2C-lower-H2AX.tif]

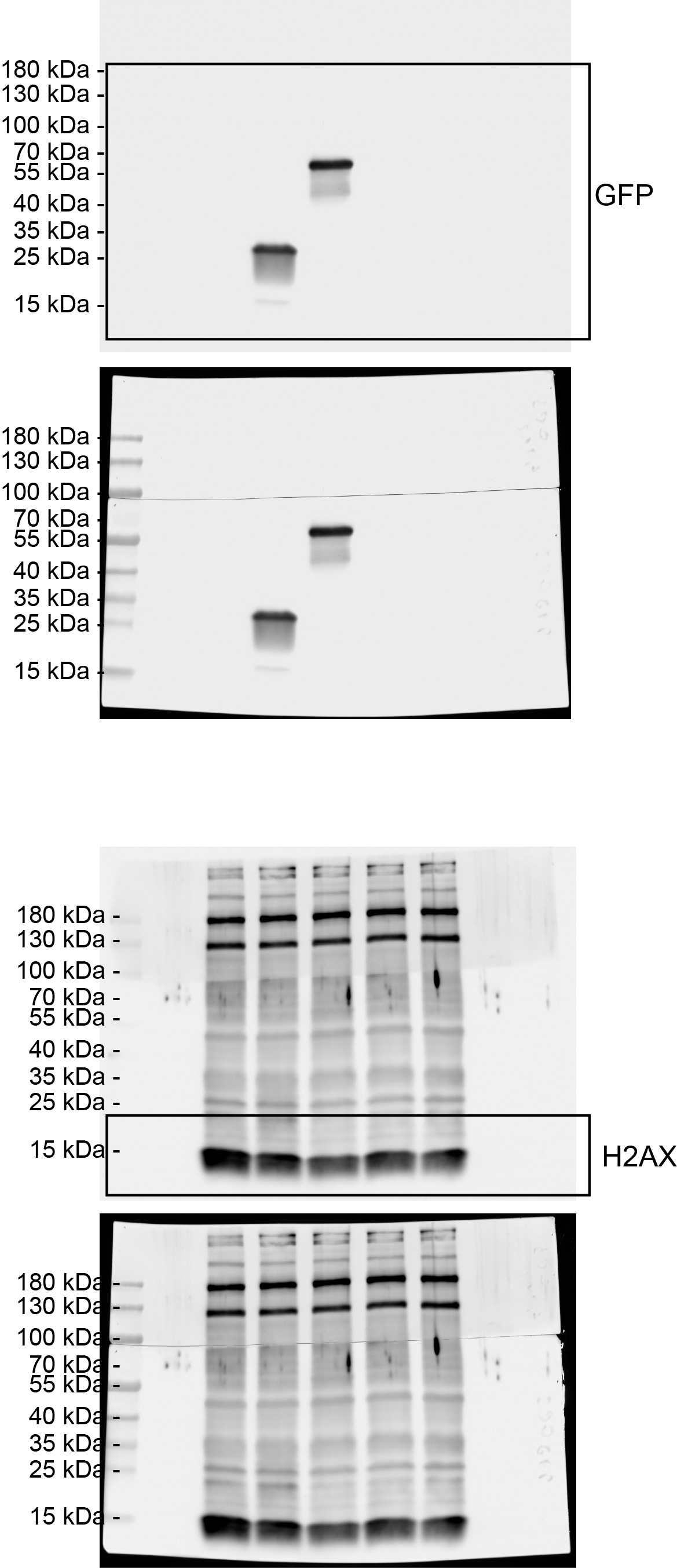

Supplement: Figure 5—figure supplement 2—source data 2. — The tiff files correspond to uncropped pictures of the chemiluminescent signal acquired on a BioRad Chemidoc. Two different immunoblotting of the same extracts were used for this figure (respectively labeled upper and lower). The regions used to generate the figure are highlighted for each immunoblot by back squares in the jpg files, which also contain at the bottom an overlay with a picture of the membrane to locate the protein ladder positions. [file elife-73913-fig5-figsupp2-data2.zip › Figure 5-figure supplement 2-source data 2/Fig.5-S2C-lower.jpg]

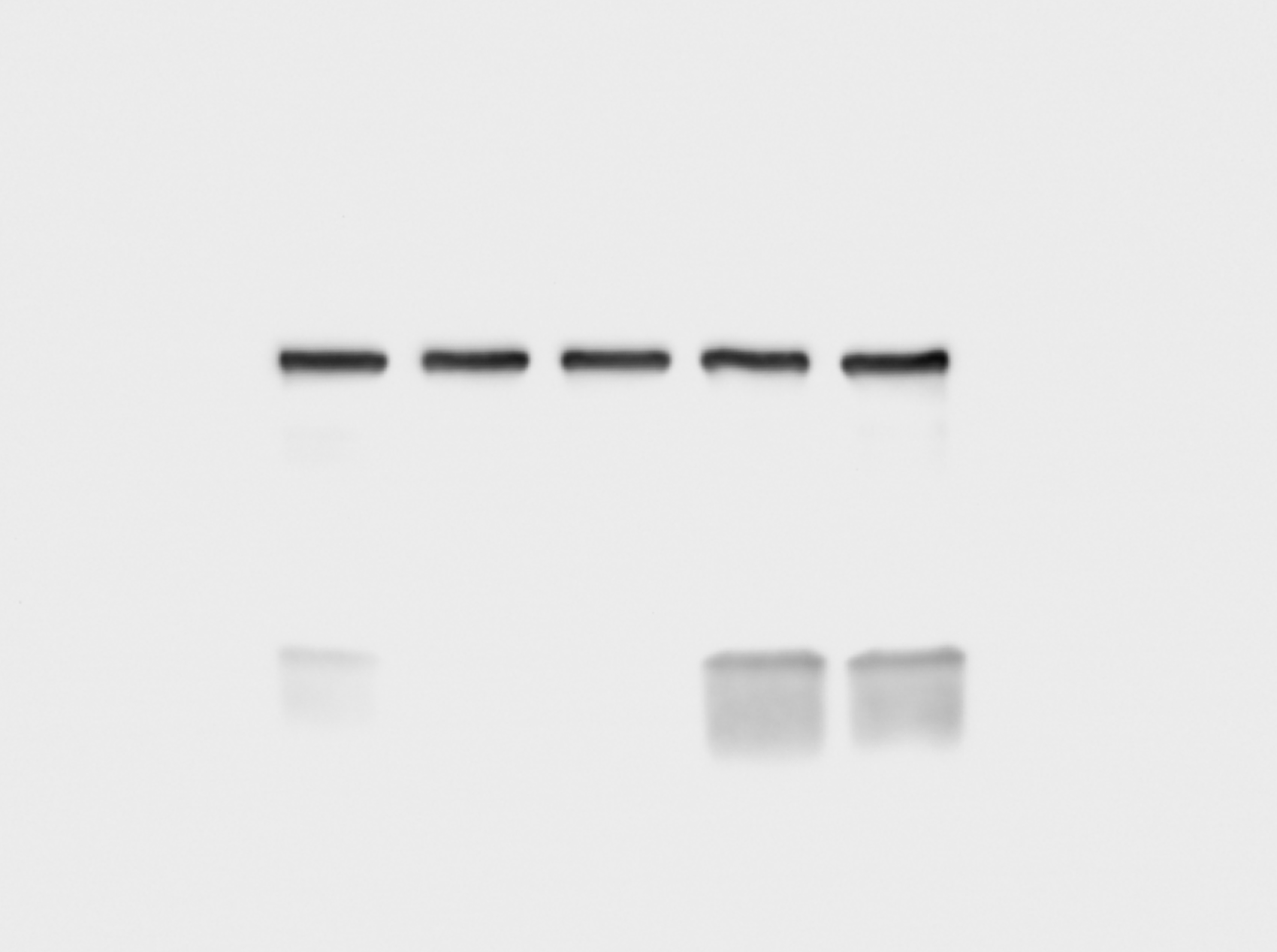

Supplement: Figure 5—figure supplement 2—source data 2. — The tiff files correspond to uncropped pictures of the chemiluminescent signal acquired on a BioRad Chemidoc. Two different immunoblotting of the same extracts were used for this figure (respectively labeled upper and lower). The regions used to generate the figure are highlighted for each immunoblot by back squares in the jpg files, which also contain at the bottom an overlay with a picture of the membrane to locate the protein ladder positions. [file elife-73913-fig5-figsupp2-data2.zip › Figure 5-figure supplement 2-source data 2/Fig.5-S2C-upper-Ku80.tif]

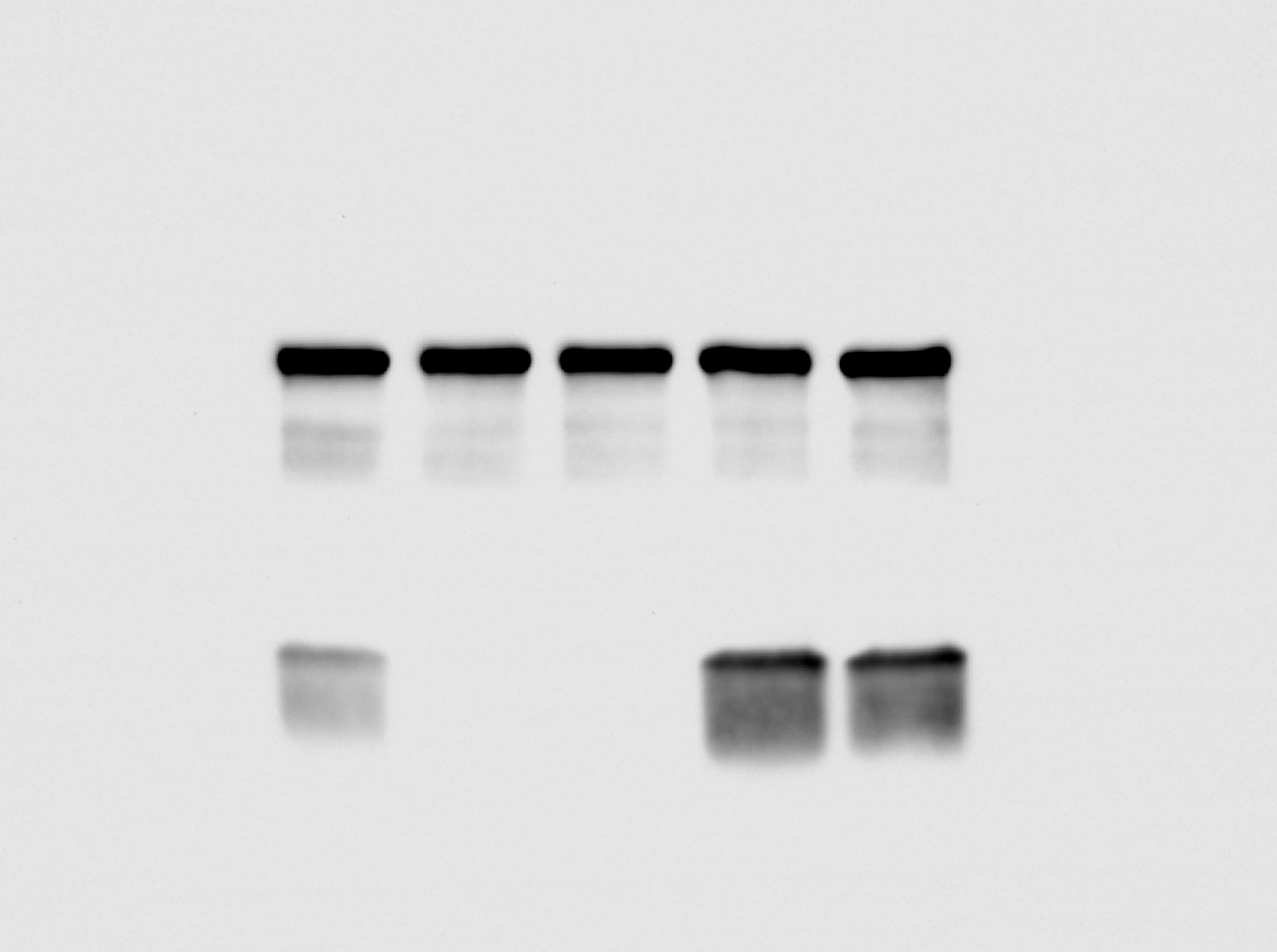

Supplement: Figure 5—figure supplement 2—source data 2. — The tiff files correspond to uncropped pictures of the chemiluminescent signal acquired on a BioRad Chemidoc. Two different immunoblotting of the same extracts were used for this figure (respectively labeled upper and lower). The regions used to generate the figure are highlighted for each immunoblot by back squares in the jpg files, which also contain at the bottom an overlay with a picture of the membrane to locate the protein ladder positions. [file elife-73913-fig5-figsupp2-data2.zip › Figure 5-figure supplement 2-source data 2/Fig.5-S2C-upper-RDH11.tif]

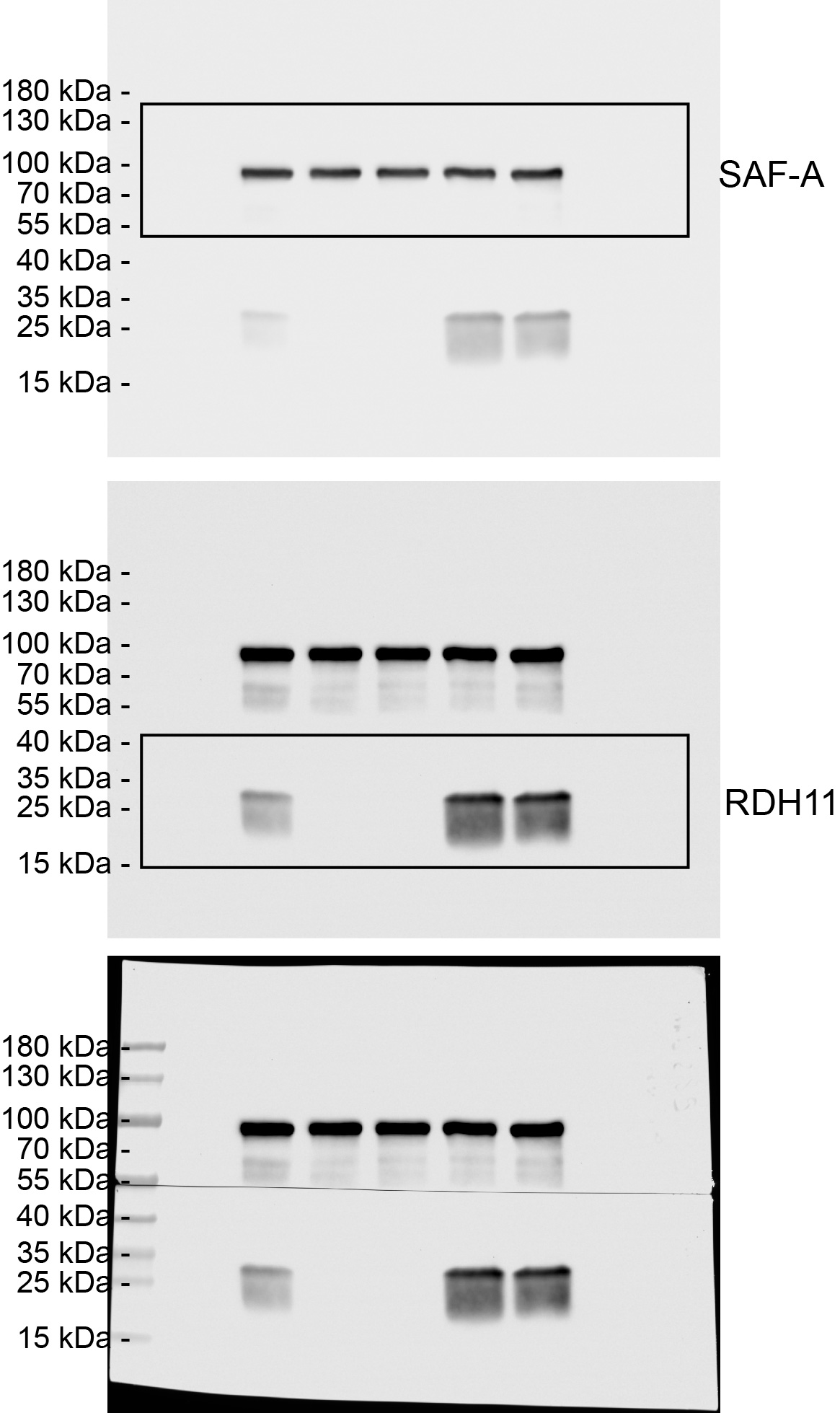

Supplement: Figure 5—figure supplement 2—source data 2. — The tiff files correspond to uncropped pictures of the chemiluminescent signal acquired on a BioRad Chemidoc. Two different immunoblotting of the same extracts were used for this figure (respectively labeled upper and lower). The regions used to generate the figure are highlighted for each immunoblot by back squares in the jpg files, which also contain at the bottom an overlay with a picture of the membrane to locate the protein ladder positions. [file elife-73913-fig5-figsupp2-data2.zip › Figure 5-figure supplement 2-source data 2/Fig.5-S2C-Upper.jpg]
